# Therapeutic Drug Monitoring for Dose Optimization of Infliximab in Patients With Inflammatory Bowel Disease: An Analysis of Canadian Real-World Data

**DOI:** 10.1155/cjgh/5713315

**Published:** 2025-02-06

**Authors:** David C. Sealey, Kai Fai Ho, Z. Christina Zhou, Michael Clark, Brian G. Feagan, Remo Panaccione, A. Hillary Steinhart, Elena Bolshtyansky, Martin Williamson, Waqqas Afif

**Affiliations:** ^1^Johnson & Johnson, Toronto, Ontario, Canada; ^2^STAT-TU Inc., Elora, Ontario, Canada; ^3^Johnson & Johnson, New Brunswick, New Jersey, USA; ^4^Departments of Medicine and Epidemiology and Biostatistics, Western University, London, Ontario, Canada; ^5^Alimentiv, London, Ontario, Canada; ^6^Division of Gastroenterology and Hepatology, University of Calgary, Calgary, Alberta, Canada; ^7^Zane Cohen Centre for Digestive Diseases, Mount Sinai Hospital, Toronto, Ontario, Canada; ^8^Division of Gastroenterology & Hepatology, Temerty Faculty of Medicine, University of Toronto, Toronto, Ontario, Canada; ^9^Division of Gastroenterology, McGill University Health Center, Montreal, Québec, Canada

**Keywords:** Crohn's disease, inflammatory bowel disease, infliximab, patient support program, real-world evidence, therapeutic drug monitoring, ulcerative colitis

## Abstract

**Background:** Although it is generally believed that infliximab dose optimization in patients with inflammatory bowel disease with low serum infliximab concentration at trough results in increased treatment persistence, empirical data to support this notion are lacking. This study evaluated the association of infliximab therapeutic drug monitoring (TDM) and TDM-associated dose optimization with persistence in real-world practice.

**Methods:** Data from adults with Crohn's disease (CD) or ulcerative colitis (UC) who participated in a national patient support program (PSP) in Canada were analyzed. Participants who had a first TDM evaluation (with a recorded infliximab trough concentration) in the maintenance phase of treatment were assessed (excluding those who underwent prior dose optimization). Persistence was evaluated using time-dependent Cox proportional hazards models.

**Results:** In the overall population of patients with CD or UC, TDM was not associated with longer persistence (*n* = 13,203). In patients with no prior dose optimization (*n* = 2729) who had a serum infliximab concentration of < 3 μg/mL, dose optimization within 9 weeks of TDM was associated with significantly longer persistence (HR: 0.36; 95% CI: 0.26, 0.50 for CD [*n* = 711] and HR: 0.30, 95% CI: 0.21, 0.43 for UC [*n* = 501]). Sensitivity analyses yielded similar results when using a threshold concentration of < 5 μg/mL. In an analysis excluding patients who received no further treatment after TDM, the association between dose optimization and longer persistence was not confirmed in patients with CD, and mostly confirmed in patients with UC at a threshold concentration of < 3 μg/mL.

**Conclusion:** TDM-associated dose optimization in patients with UC with low serum infliximab concentrations was associated with longer persistence. This association was not confirmed in patients with CD. This study demonstrated that real-world data from a PSP-generated cohort can be evaluated to inform clinical practice and that this approach may be complementary to other types of cohort studies.

## 1. Introduction

Over the past 20 years, the introduction of tumor necrosis factor (TNF) antagonist therapy has revolutionized the medical management of Crohn's disease (CD) and ulcerative colitis (UC) [1–3]. Infliximab (IFX; REMICADE, Janssen Pharmaceutical Companies of Johnson & Johnson), the first available TNF antagonist, has been widely adopted in clinical practice as a standard therapy for moderate to severe CD and UC [4–7]. Notwithstanding the superior efficacy of IFX compared to conventional therapies, some patients fail to respond to the initial induction regimen. Moreover, a substantial number of responders ultimately lose response and discontinue treatment [7–9]. Inadequate drug exposure due to the development of immunogenicity or patient level differences in drug clearance is the best-understood cause of these problems. To overcome these limitations, clinicians have adopted both therapeutic drug monitoring (TDM)–guided and empirical dose intensification strategies. TDM-guided dose optimization is the practice of measuring serum drug concentrations to inform changes in dosing with the goal of achieving a target drug concentration above a specified therapeutic threshold [10]. Furthermore, TDM can be classified as either “reactive” (defined by use in symptomatic patients) or “proactive” (use in asymptomatic patients). Although definitive studies demonstrating the efficacy of either approach are lacking, considerable observational evidence supports the concept of reactive TDM, and its use is endorsed by several national practice guidelines for both CD and UC [5, 10–12].

Notably, these recommendations are based predominantly on data from small single-center retrospective cohort studies or post hoc analyses of randomized controlled trials [13–20]. Prospective, randomized trials of proactive TDM, using an optimal trough concentration of 3 μg/mL during maintenance, have yielded equivocal results [21, 22]. In the Trough Concentration Adapted Infliximab Treatment (TAXIT) trial that studied treatment-experienced patients with either CD or UC, although not a primary endpoint, targeting a trough IFX concentration of 3–7 μg/mL during maintenance therapy was associated with fewer disease flares compared to clinically based dosing [21]. However, in the Tailored Treatment With Infliximab for Active Crohn's Disease (TAILORIX) trial that evaluated biologic-naïve patients with CD, targeting a trough IFX concentration of 3 μg/mL shortly after induction was not superior to dosing based exclusively upon symptoms [22]. Accordingly, additional information regarding the value of IFX TDM and dose optimization is needed.

This study used real-world data from a national Canadian patient support program (PSP) to investigate the use of TDM in adult patients receiving IFX for the treatment of CD or UC. The aim of this study was to determine whether TDM and TDM-associated dose optimization were associated with improved treatment persistence.

## 2. Methods

### 2.1. Study Design and Data Source

This retrospective cohort study used administrative data collected through Janssen BioAdvance, a national PSP in Canada. BioAdvance provides medication infusion/injection and case management services to patients and physicians for Janssen treatments including IFX. Based upon the quantity of product shipped from the manufacturer and the amount of drug administered, it is estimated that over 90% of patients with CD and UC treated with IFX in Canada received treatment and support through BioAdvance during the study period.

The BioAdvance database contains patient-level, longitudinal data including enrollment records, medical orders, treatment records, and laboratory test results. BioAdvance offers clinicians the option of ordering preinfusion blood draws and TDM testing (ELISA); the results of these tests are recorded in the database. Based upon the interpretation of the data available, there were temporal, provincial, lab-level, and practice-level differences with respect to when antibodies to IFX (ATI) were evaluated. Often, evaluation of ATI was performed when the serum IFX concentration was ≤ 0.035 μg/mL. TDM testing may have occurred outside of BioAdvance, in which case those data were not recorded in the database.

Prior to study initiation, an independent data quality and feasibility assessment was performed (Mu Sigma Business Solutions LLC, Northbrook, Illinois). The data were deemed to be of sufficient quality and integrity (in terms of completeness, conformance, and plausibility) for analysis according to the study protocol. The study protocol was approved by the Advarra Institutional Review Board (IRB; registered with the Office of Human Research Protections and the Food and Drug Administration [FDA] under IRB#00000971). The IRB determined that Canadian privacy requirements for a waiver of consent were met. All analyses were performed on deidentified data. Patients with CD and UC were analyzed separately.

### 2.2. Overall Study Population

The overall study population included adult male and female patients with a primary diagnosis of CD or UC (excluding a primary diagnosis of perianal fistulizing CD) who initiated IFX treatment between January 1, 2015 and December 31, 2018. Patients were required to be ≥ 18 to ≤ 90 years of age at the time of first IFX treatment and have a first recorded weight of < 130 kg. The lower boundary of ≥ 18 years of age was selected as the Canadian IFX product monograph only includes a statement related to the consideration of dose adjustments in adults [23]. The upper boundary of ≤ 90 years of age and weight criterion of < 130 kg were selected to minimize the risk of patient reidentification due to the small number of patients that did not meet these criteria. An 8-month lookback period (May–December 31, 2014, limited by lack of data availability prior to May 2014) was used to exclude patients with prior IFX treatment. For analysis of the overall study population, the index date was the date of IFX treatment initiation.

### 2.3. Subgroup Population

In a prespecified analysis, patients were further selected into a subgroup population if they had a first TDM in the maintenance phase of treatment (defined as occurring after Week 9) with a recorded TDM result prior to meeting censoring or discontinuation rules as defined below. The index date was the date of the first TDM. For analysis, patients were further grouped into distinct subpopulations based on whether they received a dose increase (optimization) prior to the first TDM measurement. In each subpopulation, subsets of patients were further defined based upon their first serum IFX concentration being above or below 3 μg/mL, and whether they received a subsequent (post-TDM measurement) dose optimization within 9 weeks. Sensitivity analyses were conducted at a cut-off of 5 μg/mL and a post-TDM period of dose optimization within 17 weeks. Post hoc sensitivity analyses were also conducted including analyses using a cut-off of 10 μg/mL and analyses excluding from the subgroup population patients that did not receive treatment after TDM.

### 2.4. Definitions

#### 2.4.1. Treatment Persistence

Treatment persistence was measured as the time from the index date until the discontinuation rule was met, or the censoring date was reached if the latter occurred first. Patients with a gap of > 20 weeks between treatments, or between a treatment and the censoring date were deemed discontinued as of 8 weeks after the last treatment preceding the gap. This gap served as a grace period to allow for treatment interruptions for reasons such as travel or surgery. The additional 8 weeks represent the length of one treatment cycle based upon the maintenance treatment interval recommended in the IFX product monograph [23]. Patients were right-censored on July 31, 2019, if they reached this date prior to discontinuation. For the overall study population, sensitivity analyses were also conducted using a shorter gap of > 12 weeks.

#### 2.4.2. Dose Optimization

According to the IFX product monograph, the recommended dose for CD and UC is an induction regimen of 5 mg/kg at 0, 2, and 6 weeks, followed by a maintenance regimen of 5 mg/kg every 8 weeks. The product monograph also states that in some adult patients, consideration may be given to adjusting the dose up to 10 mg/kg to sustain clinical response and remission; in addition to the health professional's clinical assessment, TDM results should be taken into account before considering dose adjustment [23].

Instances of dose optimization were identified using predefined low and high thresholds, based on the recommended treatment regimen, to avoid capturing minor differences in treatment interval and/or dose level [23]. Dose optimization meeting low-threshold criteria was defined as a treatment interval decrease of ≥ 1.57 weeks with a posterior interval of ≤ 6.57 weeks, and/or a dose level increase of ≥ 1.5 mg/kg with a posterior dose level of ≥ 7 mg/kg. For example, these criteria would capture a treatment interval decrease from 8 weeks to 6 weeks, or a dose level increase from 5 mg/kg to 7.5 mg/kg. Dose optimization meeting high-threshold criteria was defined as a treatment interval decrease of ≥ 1.57 weeks with a posterior interval of ≤ 5 weeks, and/or a dose level increase of ≥ 1.5 mg/kg with a posterior dose level of ≥ 9 mg/kg. For example, these criteria would capture a treatment interval decrease from 8 to 4 weeks, or a dose level increase from 5 to 10 mg/kg.

### 2.5. Statistical Analyses

Patient demographics and baseline characteristics were summarized using descriptive statistics.

For the overall study population, an unstratified, unadjusted Kaplan–Meier (KM) method was used to estimate persistence over time. To assess the potential effect of TDM on persistence, Cox proportional hazards models were used in which TDM use was included as a time-dependent covariate to avoid immortal-time bias [24, 25]. Analyses were stratified by year of treatment initiation. Age group (18 to < 65, 65–90), gender, first recorded weight (quartiles), and “province/region of treating physician” were included as fixed covariates. For each analysis, four models were developed to evaluate interaction factors and the proportional hazards assumption (Supporting Information Table 10a). For each analysis, the appropriate model was selected for interpretation based on the significance testing of interaction terms and the proportional hazards assumption (Supporting Information Figure 1a). Results are presented as hazard ratios (HRs) with 95% confidence intervals (CIs). An HR < 1 indicates a persistence advantage compared to the reference.

The subgroup population was specifically selected to evaluate the potential association between TDM-associated dose optimization and persistence, if it exists. In these analyses, differences in persistence between patient subsets were compared using Cox proportional hazards models in which TDM-associated dose optimization within the postindex period (9/17 weeks) was included as a time-dependent covariate. Covariate factors were included, and for each analysis, four models were developed to evaluate interaction factors and the proportional hazards assumption (Supporting Information Table 10b). For each analysis, the appropriate model was selected for interpretation based on the significance testing of interaction terms and the proportional hazards assumption (Supporting Information Figure 1b). When the proportional hazards assumption was violated, comparisons were evaluated at two time points for determining a post-TDM dose optimization: at 4 weeks and at 6 weeks. In relation to the 9-week post-TDM period (approximately one cycle of treatment based upon a treatment interval of every 8 weeks), 4 weeks represents a shorter, commonly observed interval, and 6 weeks represents the midpoint between 4 and 8 weeks. Results are presented as HRs with 95% CIs.

Quantitative models were developed to evaluate the potential impact of measured and unmeasured confounders on comparisons between patient subsets [26]. The persistence of each patient in a specified subset was artificially increased or decreased to determine the magnitude of adjustment required to contradict the observed result.

Statistical analyses were performed using SAS version 9.3. The threshold for statistical significance was *p* < 0.05 unless otherwise specified. No adjustments were made for multiple comparisons.

## 3. Results

### 3.1. Overall Study Population

**Figure 1:**
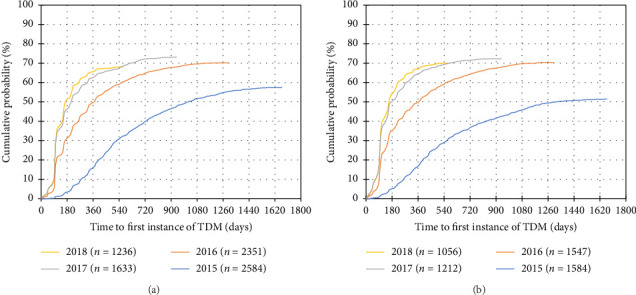
Cumulative distribution of time to the first instance of TDM per cohort year of initiation. TDM: therapeutic drug monitoring. (a) Crohn's disease. (b) Ulcerative colitis.

A total of 7804 patients with CD and 5399 patients with UC were included (Table 1, Supporting Information Figure 2). 5170 (66.2%) patients with CD and 3520 (65.2%) patients with UC had at least one instance of TDM during IFX treatment. In both disease cohorts, the distribution of “province of treating physician” was significantly different between patients who received TDM compared to patients who did not receive TDM. Of the 10 provinces in Canada, the proportion of patients in Saskatchewan and Manitoba who received TDM may be underrepresented due to TDM testing performed outside of BioAdvance in those provinces. A trend towards earlier use of TDM and use of TDM in a higher proportion of patients was observed in more recent years of treatment initiation (Figure 1) (Table 2).

#### 3.1.1. Persistence Estimates and Impact of TDM on Persistence

**Figure 2:**
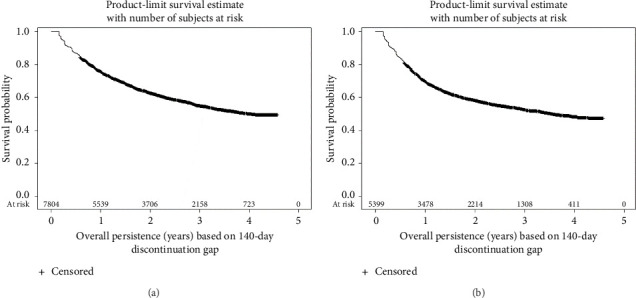
Kaplan–Meier estimates of persistence. (a) Crohn's disease. (b) Ulcerative colitis.

KM survival analyses based upon the 20-week discontinuation rule showed 1-year actuarial persistence rates of 76.0% and 69.9% in patients with CD and UC, respectively (Figure 2). Persistence rates at 4 years were 49.9% for CD and 48.4% for UC. Persistence rates based upon the more stringent 12-week discontinuation rule were approximately 10% lower (Supporting Information Figure 3).

In both diseases, time on treatment after TDM was not associated with longer IFX persistence compared to time on treatment prior to any TDM (Supporting Information Table 1), indicating that TDM was not associated with longer persistence. Factors associated with longer persistence included age < 65 years (*p* ≤ 0.0001) in CD and UC, as well as male gender (*p* ≤ 0.0001) in CD. It is possible that patients who received TDM were subject to selection bias based on disease activity (e.g., severity, nonresponse, and loss of response), treatment history, treatment options, physician clinical practice, or other unmeasured factors. Also, the model did not account for IFX dosing, TDM results, or TDM-associated dosing changes.

### 3.2. Subgroup Population

The targeted subgroup analysis was conducted to evaluate the association between TDM results and TDM-associated dose optimization with persistence. A total of 2219 patients with CD and 1575 patients with UC were included in the subgroup population (see Methods; Table 3). Compared to patients in the overall population who received TDM, patients in the subgroup population in both disease cohorts had similar age and gender distributions, as well as first recorded weights (Tables 1 and 3). A majority of patients in the subgroup population initiated treatment in 2017 or 2018. In both disease cohorts, the subgroup population had higher proportions of patients in Ontario and the Atlantic region, and lower proportions of patients in Alberta, Quebec, and Saskatchewan/Manitoba. Patients in the subgroup population in both disease cohorts had longer mean and median times to first TDM compared to patients in the overall population who received TDM (Table 4).

#### 3.2.1. Impact of TDM-Associated Dose Optimization on Persistence

**Figure 3:**
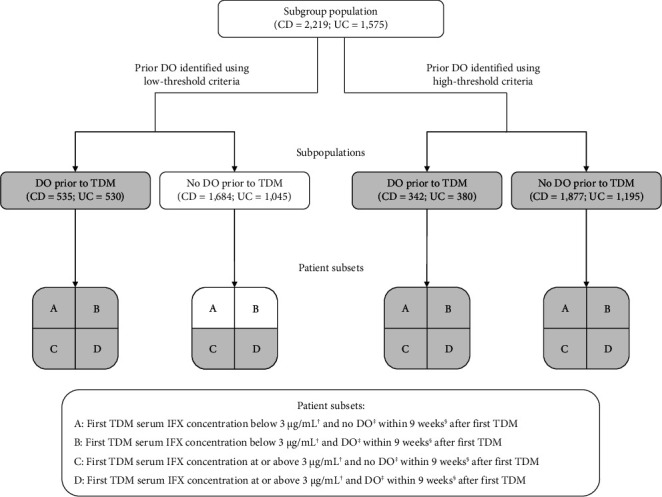
Schematic of subgroup subpopulations and patient subsets. CD: Crohn's disease, DO: dose optimization, IFX: infliximab, TDM: therapeutic drug monitoring, and UC: ulcerative colitis. Results for the patient subsets that are not grayed out are shown in Figure 4. Results for all subpopulations and patient subsets can be found in the Supporting Information. ^†^Sensitivity analyses were conducted with serum IFX 5 and 10 μg/mL. ^‡^Separate analyses were conducted for dose optimization meeting low-threshold and high-threshold criteria after TDM. ^§^Sensitivity analyses were conducted for dose optimization within 17 weeks after TDM.

**Figure 4:**
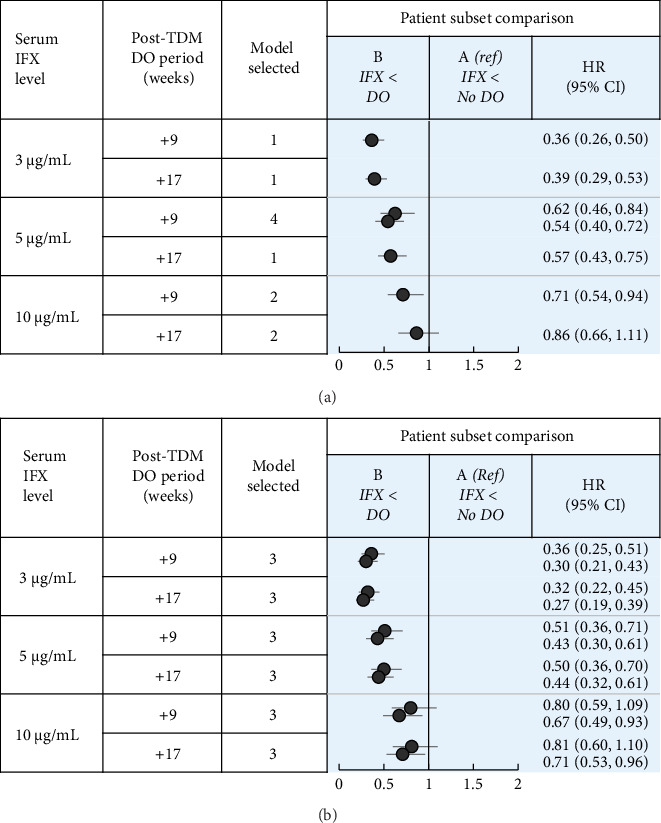
(a) Comparisons of persistence in patients with Crohn's disease who did not receive dose optimization based on low-threshold criteria prior to the first TDM. CI: confidence interval, DO: dose optimization, HR: hazard ratio, IFX: infliximab, Ref: reference, and TDM: therapeutic drug monitoring. Dose optimization thresholds: high: a treatment interval decrease of ≥ 11 days (1.57 weeks) with a posterior interval of ≤ 35 days (5 weeks), and/or a dose level increase of ≥ 1.5 mg/kg with a posterior dose level of ≥ 9 mg/kg. Low: a treatment interval decrease of ≥ 11 days (1.57 weeks) with a posterior interval of ≤ 46 days (6.57 weeks), and/or dose level increase of ≥ 1.5 mg/kg with a posterior dose level of ≥ 7 mg/kg. Dose optimization within post-TDM periods was identified using low-threshold criteria. Model details are described in the Supporting Information Table 10b. Model selection decision tree is shown in Supporting Information Figure 1b. Model results are available in the Supporting Information Table 11. HR (95% CI) displayed to a maximum of 2.0 and shown in bold when *p* < 0.05. Doublet results based on DO at 4 and 6 weeks, respectively. A hazard ratio of < 1 indicates a persistence advantage over the reference. If the CI includes 1, there is no statistically significant difference in persistence. If the CI does not include 1, the difference in persistence is statistically significant. *A*: first TDM serum IFX concentration below level and no dose optimization within the post-TDM period and *B*: first TDM serum IFX concentration below level and dose optimization within the post-TDM period. (b) Comparisons of persistence in patients with ulcerative colitis who did not receive dose optimization based on low-threshold criteria prior to first TDM. CI: confidence interval, DO: dose optimization, HR: hazard ratio, IFX: infliximab, Ref: reference, and TDM: therapeutic drug monitoring. Dose optimization thresholds: high: a treatment interval decrease of ≥ 11 days (1.57 weeks) with a posterior interval of ≤ 35 days (5 weeks), and/or a dose level increase of ≥ 1.5 mg/kg with a posterior dose level of ≥ 9 mg/kg. Low: a treatment interval decrease of ≥ 11 days (1.57 weeks) with a posterior interval of ≤ 46 days (6.57 weeks), and/or dose level increase of ≥ 1.5 mg/kg with a posterior dose level of ≥ 7 mg/kg. Dose optimization within post-TDM periods was identified using low-threshold criteria. Model details are described in Supporting Information Table 10b. The model selection decision tree is shown in Supporting Information Figure 1b. Model results are available in Supporting Information Table 13. HR (95% CI) displayed to a maximum of 2.0 and shown in bold when *p* < 0.05. Doublet results based on DO at 4 weeks and 6 weeks, respectively. A hazard ratio of < 1 indicates a persistence advantage over the reference. If the CI includes 1, there is no statistically significant difference in persistence. If the CI does not include 1, the difference in persistence is statistically significant. *A*: first TDM serum IFX concentration below level and no dose optimization within post-TDM period and *B*: first TDM serum IFX concentration below level and dose optimization within the post-TDM period.

The number of patients included in each subpopulation (i.e., patients who received or did not receive dose optimization meeting low-threshold or high-threshold criteria prior to TDM) is shown in Figure 3. The demographics and baseline characteristics of patients in the defined subsets are summarized in Supporting Information Tables 2–9. The results of the Cox regression analyses demonstrated a strong association between TDM-associated dose optimization and persistence (Figure 4, Supporting Information Figure 4, and Supporting Information Tables 11–18). Results for the subpopulation of patients with no dose optimization meeting low-threshold criteria prior to TDM are described as follows. Additional results for this subpopulation, as well as results for the subpopulation of patients with dose optimization meeting high-threshold criteria prior to TDM, are described in Supporting Information Text 1.

If TDM-guided dose optimization resulted in longer persistence, analysis of the subpopulation of patients without dose optimization meeting low-threshold criteria prior to TDM would have the greatest discriminatory power to observe such an effect. A total of 1684 patients with CD and 1045 patients with UC were included in this subpopulation. In CD, patient subsets differed significantly by mean age, age group, province, year of IFX treatment initiation, and mean time to first TDM (Supporting Information Table 2a; *p* < 0.05). In UC, patient subsets differed significantly by mean age, year of IFX treatment initiation, mean time to first TDM, and year of first TDM (Supporting Information Table 4a; *p* < 0.05).

Results of the main Cox proportional hazards models evaluating persistence are shown in Supporting Information Tables 11a–11f and 13a–13f, with the main subset comparisons of interest summarized in Figures 4(a) and 4(b). The covariate of log (days to TDM) was associated with longer persistence in both CD and UC, indicating that as more time elapsed prior to the first TDM, treatment persistence after TDM was longer. Age < 65 years was associated with longer persistence in UC.

In patients with CD with serum IFX < 3 μg/mL, dose optimization within 9 weeks after the first TDM (Subset B) was associated with longer persistence than no dose optimization (Subset A) (HR: 0.36, 95% CI: 0.26, 0.50; Figure 4(a), B vs. A). Assessment of demographic and baseline characteristic similarities and differences revealed covariates with either confounding (at least partially; year of IFX treatment initiation), competing (time to first TDM), or no expected impact on the differences in persistence observed. To assess the potential impact of measured and unmeasured confounders, models were developed in which the post-TDM persistence of patients who received dose optimization was reduced iteratively (Supporting Information Tables 19–22). After a median post-TDM reduction in persistence of 250 days (equivalent to 4.5 8-week cycles of IFX treatment), the positive association was no longer significant (Supporting Information Table 19a). Therefore, confounding was considered unlikely to account for the positive association observed. A comprehensive analysis of the association of persistence with ATI could not be completed due to missing data (ATI not tested or not reported; see Methods). In Subsets A and B, the proportions of patients who had ATI at first TDM were 45% and 29%, respectively; the proportions with no ATI data were 49% and 61%, respectively.

In patients with UC with serum IFX < 3 μg/mL, dose optimization at 4 weeks (HR: 0.36, 95% CI: 0.25, 0.51) and 6 weeks (HR: 0.30, 95% CI: 0.21, 0.43) after the first TDM (Subset B) was associated with longer persistence than no dose optimization (Subset A) (Figure 4(b), B vs. A). Assessment of demographic and baseline characteristic similarities and differences revealed covariates with either confounding (at least partially; year of IFX treatment initiation), competing (time to first TDM), or no expected impact on the differences in persistence observed. Using modeling as described above, after a median reduction of 175 and 225 days in the post-TDM persistence of patients with dose optimization at 4 and 6 weeks, respectively, the positive association was no longer significant (Supporting Information Table 20a). These reductions are equivalent to 3.1 and 4.0 8-week cycles, respectively, of IFX treatment. Therefore, confounding was considered unlikely to account for the positive association observed. In Subsets A and B, the proportions of patients who had ATI at first TDM were 51% and 24%, respectively; the proportions with no ATI data were 46% and 65%, respectively.

Sensitivity analyses conducted with a serum IFX concentration of 5 μg/mL and/or with a longer post-TDM period for dose optimization similarly found a significant association between post-TDM dose optimization and longer persistence (Figures 4(a) and 4(b), B vs. A). When a serum IFX concentration of 10 μg/mL was used, the results were mixed and depended on the settings of the model. Dose optimization within 17 weeks after the first TDM in patients with CD, or at 4 weeks after the first TDM in patients with UC was not associated with longer persistence.

Considering that discontinuation after TDM may be related to ATI results, clinical status, and other factors, additional sensitivity analyses were performed in an attempt to further isolate the impact of dose optimization on persistence. Patients who received no treatment after TDM were excluded from the subgroup population, and the proportional hazards models were reevaluated. In patients with CD with serum IFX < 3 or < 5 μg/mL, dose optimization within 9 or 17 weeks after the first TDM was not associated with longer persistence compared to no dose optimization (Supporting Information Figure 5 and Supporting Information Table 23). In patients with UC with serum IFX < 3 μg/mL, dose optimization at 6 weeks after the first TDM was associated with longer persistence compared to no dose optimization, whereas dose optimization at 4 weeks after TDM yielded mixed results (Supporting Information Figure 5 and Supporting Information Table 24). In patients with serum IFX < 5 μg/mL, dose optimization at 4 or 6 weeks after the first TDM was not associated with longer persistence compared to no dose optimization. Overall, in patients with CD, the results of the main analyses were not confirmed in the sensitivity analyses. In patients with UC, the results of the main analyses were mostly confirmed in the sensitivity analyses at a serum IFX threshold concentration of < 3 μg/mL.

## 4. Discussion

TDM is widely used by clinicians to optimize therapy in patients with CD and UC receiving treatment with IFX and is established in treatment guidelines. This study provides additional evidence to address how TDM is utilized in a large real-world sample of patients with inflammatory bowel disease (IBD). The analysis was based on administrative data on a cohort of 13,203 patients (2729 patients in the main subpopulation) from a national PSP in Canada over a total exposure period of approximately 4.5 years. The large sample size and availability of high-quality data on dosing, treatment duration, and drug concentrations allowed for extensive sensitivity and bias analyses which are critical to evaluating the validity of real-world evidence. In addition, immortal-time bias (frequently observed in cohort studies) was avoided by using Cox proportional hazards models with TDM use included as a time-dependent covariate [24, 25].

The IFX treatment persistence rates observed in patients with CD and UC are consistent with findings from previous Canadian studies [27–29]. Studies from the United States and Europe demonstrated varying rates of persistence in patients with IBD, with IFX persistence at 1 year ranging from 45% to 80% likely due to differences in methodology as well as an increased susceptibility to referral bias [9, 30, 31]. In contrast, this study captured an inclusive sample given it is estimated that over 90% of patients with IBD treated with IFX in Canada received treatment through BioAdvance during the study period.

Results of previous studies have suggested that IFX TDM is associated with improved treatment outcomes. For example, a positive association between serum IFX concentrations and clinical outcomes has been reported [13, 14, 17], and previous studies have found that the use of proactive TDM significantly increased IFX persistence in patients with IBD [32, 33]. In the analysis of the overall study population, no association between IFX TDM and longer persistence was observed. This result is not surprising given that dose intensification is unlikely to be effective in patients who have adequate drug exposure as a result of adhering to the standard dosing regimen or from empiric dose intensification. In contrast, when serum IFX concentrations and TDM-associated IFX dosing changes were accounted for, in a population selected for the presence of a low serum IFX concentration at first TDM with no prior dose optimization, TDM-associated dose optimization was associated with longer persistence. The association was detected using serum IFX thresholds of both 3 and 5 μg/mL. Based on sensitivity analyses whereby patients who received no treatment after TDM were removed, the positive association was mostly confirmed in UC at the 3 μg/mL level of IFX. In the TAXIT trial, TDM-guided dosing with a target trough IFX concentration of 3–7 μg/mL was associated with a lower risk of relapse in patients with CD and UC [21]. Overall, the results of our study support the recommendation that dose optimization be informed by TDM, consistent with clinical practice guidelines [5, 6, 12]. An important finding of this study was a potential “ceiling effect.” As serum IFX cut-off values were progressively increased, a trend towards a lack of a significant positive association between TDM-associated dose optimization and persistence was observed. It is possible that patients with higher serum IFX levels (below the threshold defined by the model; e.g., from 3 to 10 μg/mL) and no ATI who persisted on treatment could have contributed at least partially to this potential ceiling effect.

There are several limitations to this study. The measurement of treatment outcomes relied on treatment persistence as a surrogate for clinical efficacy, as objective markers of disease activity were not available. Nonetheless, treatment persistence is commonly used as an indirect measure of effectiveness since patients are expected to discontinue therapy if they are no longer achieving adequate response [9, 34–36]. While measured confounders were controlled or otherwise accounted for, unmeasured factors represent potential sources of bias and confounding, including the decision to use TDM which was at the discretion of treating physicians. It is possible that TDM and TDM-related treatment decisions such as dose optimization were used preferentially in patients with more refractory disease, i.e., those patients who had more severe disease or who had already failed other therapies, may have been preferentially selected for an attempt at further IFX dose optimization, possibly leading to a higher failure rate as would be expected in such a population. Also, decisions to continue or discontinue treatment may have depended at least partially on the absence or presence of ATI. We were unable to incorporate ATI results in the analytical models due to the lack of comprehensive testing and a high proportion of incomplete data in the database. Other unmeasured variables include factors related to disease and treatment history (e.g., comorbidities, prior and concomitant therapies, and smoking status) and reasons for discontinuation. The potential impact of different sources of bias was explored through sensitivity analyses and quantitative bias analysis.

## 5. Conclusions

In conclusion, this study provides real-world evidence from a national Canadian cohort of adult patients with CD and UC on the association between TDM-associated IFX dose optimization and persistence in patients with low serum IFX levels. Therapeutic thresholds of 3 and 5 μg/mL appeared to demonstrate utility for TDM-guided clinical decision-making including dose optimization, with a potential ceiling effect at higher thresholds. Based on sensitivity analyses, these findings were mostly confirmed for patients with UC at the 3 μg/mL level, and not confirmed for patients with CD. Future studies to elucidate the association between TDM-associated dose optimization, ATI results, and persistence are warranted. In addition, this study has demonstrated that real-world data from a large manufacturer-supported PSP-generated cohort can be evaluated in an attempt to inform clinical practice.

## Figures and Tables

**Table 1:** Demographics and baseline characteristics of patients with Crohn's disease.

Variable	TDM (*N* = 5170)	No TDM (*N* = 2634)	All patients (*N* = 7804)	*p* value
Age (years)				
*N*^†^	5170	2633	7803	
18–64	4665 (90.2%)	2301 (87.4%)	6966 (89.3%)	0.0792
65–90	505 (9.8%)	332 (12.6%)	837 (10.7%)	
Mean (SD)	42.9 (15.4)	44.4 (16.0)	43.4 (15.6)	0.0607
Gender				
*N*	5170	2634	7804	
Male	2421 (46.8%)	1228 (46.6%)	3649 (46.8%)	0.9378
Female	2749 (53.2%)	1406 (53.4%)	4155 (53.2%)	
First recorded weight during IFX treatment (kg)				
*N*^†^	5167	2629	7796	
Mean (SD)	74.5 (17.9)	74.2 (17.9)	74.4 (17.9)	0.7274
Province/region of treating physician				
*N*^†^	5170	2634	7801	
Alberta	653 (12.6%)	345 (13.1%)	998 (12.8%)	< 0.0001
Atlantic^‡^	635 (12.3%)	280 (10.6%)	915 (11.7%)	
British Columbia	546 (10.6%)	365 (13.9%)	911 (11.7%)	
Ontario	1759 (34.0%)	689 (26.2%)	2448 (31.4%)	
Quebec	1466 (28.4%)	627 (23.8%)	2093 (26.8%)	
Saskatchewan/Manitoba	111 (2.1%)	328 (12.5%)	439 (5.6%)	
Year of initiation of IFX treatment				
*N*^†^	5170	2634	7804	
2015	1485 (28.7%)	1099 (41.7%)	2584 (33.1%)	< 0.0001
2016	1651 (31.9%)	700 (26.6%)	2351 (30.1%)	
2017	1193 (23.1%)	440 (16.7%)	1633 (20.9%)	
2018	841 (16.3%)	395 (15.0%)	1236 (15.8%)	

*Note:* Patients who had at least one instance of TDM during their study period are summarized under TDM. *p* values comparing the TDM and no TDM groups are from a two-sample *t*-test for continuous variables and a chi-square test for categorical variables.

Abbreviations: IFX, infliximab; SD, standard deviation; TDM, therapeutic drug monitoring.

^†^ *N* value may differ from total population size when data on a variable are missing for a proportion of patients.

^‡^Atlantic includes New Brunswick, Nova Scotia, Prince Edward Island, Newfoundland and Labrador.

**Table 2:** Demographics and baseline characteristics of patients with ulcerative colitis.

Variable	TDM (*N* = 3520)	No TDM (*N* = 1879)	All patients (*N* = 5399)	*p* value
Age (years)				
*N*^†^	3514	1878	5392	
18–64	3158 (89.9%)	1639 (87.3%)	4797 (89.0%)	0.1770
65–90	356 (10.1%)	239 (12.7%)	595 (11.0%)	
Mean (SD)	42.2 (15.9)	43.7 (16.6)	42.7 (16.2)	0.1320
Gender				
*N*	3520	1879	5399	
Male	1913 (54.3%)	1028 (54.7%)	2941 (54.5%)	0.9067
Female	1607 (45.7%)	851 (45.3%)	2458 (45.5%)	
First recorded weight during IFX treatment (kg)				
*N*^†^	3517	1877	5394	
Mean (SD)	75.25 (17.4)	75.71 (17.9)	75.41 (17.6)	0.6693
Province/region of treating physician				
*N*	3520	1879	5399	
Alberta	544 (15.5%)	226 (12.0%)	770 (14.3%)	< 0.0001
Atlantic^‡^	307 (8.7%)	136 (7.2%)	443 (8.2%)	
British Columbia	410 (11.6%)	289 (15.4%)	669 (12.9%)	
Ontario	1375 (39.1%)	627 (33.4%)	2002 (37.1%)	
Quebec	807 (22.9%)	392 (20.9%)	1199 (22.2%)	
Saskatchewan/Manitoba	77 (2.2%)	209 (11.1%)	286 (5.3%)	
Year of initiation of IFX treatment				
*N*^†^	3520	1878	5399	
2015	815 (23.2%)	769 (40.9%)	1584 (29.3%)	< 0.0001
2016	1089 (30.9%)	458 (24.4%)	1547 (28.7%)	
2017	876 (24.9%)	336 (17.9%)	1212 (22.4%)	
2018	740 (21.0%)	316 (16.8%)	1056 (19.6%)	

*Note:* Patients who had at least one instance of TDM during their study period are summarized under TDM. *p* values comparing the TDM and no TDM groups are from a two-sample *t*-test for continuous variables and a chi-square test for categorical variables.

Abbreviations: IFX, infliximab; SD, standard deviation; TDM, therapeutic drug monitoring.

^†^ *N* value may differ from total population size when data on a variable are missing for a proportion of patients.

^‡^Atlantic includes New Brunswick, Nova Scotia, Prince Edward Island, Newfoundland and Labrador.

**Table 3:** Demographics and baseline characteristics of patients with Crohn's disease in the subgroup population.

	*N* = 2219
Age (years)	
Mean (SD)	43.0 (15.4)
Median (Q1 and Q3)	42.0 (30, 55)
Age group (years)	
18–64	1994 (89.9%)
65–90	225 (10.1%)
Gender	
Female	1142 (51.5%)
Male	1077 (48.5%)
First recorded weight (kg)	
Mean (SD)	74.3 (17.8)
Missing	3
Province	
Alberta	80 (3.6%)
Atlantic^†^	397 (17.9%)
British Columbia	238 (10.7%)
Ontario	1077 (48.5%)
Quebec	417 (18.8%)
Saskatchewan/Manitoba	10 (0.5%)
Year of initiation of IFX treatment	
2015	375 (16.9%)
2016	446 (20.1%)
2017	751 (33.8%)
2018	647 (29.2%)
Time to first TDM (days)	
Mean (SD)	397.5 (347.3)
Median (Q1 and Q3)	251.0 (108, 610)

Abbreviations: IFX, infliximab; Q, quartile; SD, standard deviation; TDM, therapeutic drug monitoring.

^†^Atlantic includes New Brunswick, Nova Scotia, Prince Edward Island, Newfoundland and Labrador.

**Table 4:** Demographics and baseline characteristics of patients with ulcerative colitis in the subgroup population.

	*N* = 1575
Age (years)	
Mean (SD)	42.3 (16.0)
Median (Q1 and Q3)	40.0 (29, 55)
Missing	2
Age group (years)	
18–64	1414 (89.9%)
65–90	159 (10.1%)
Missing	2
Gender	
Female	738 (46.9%)
Male	837 (53.1%)
First recorded weight (kg)	
Mean (SD)	75.1 (17.4)
Missing	3
Province	
Alberta	90 (5.7%)
Atlantic^†^	198 (12.6%)
British Columbia	195 (12.4%)
Ontario	873 (55.4%)
Quebec	214 (13.6%)
Saskatchewan/Manitoba	5 (0.3%)
Year of initiation of IFX treatment	
2015	199 (12.6%)
2016	298 (18.9%)
2017	538 (34.2%)
2018	540 (34.3%)
Time to first TDM (days)	
Mean (SD)	352.5 (327.5)
Median (Q1 and Q3)	208.0 (102, 492)

Abbreviations: IFX, infliximab; Q, quartile; SD, standard deviation; TDM, therapeutic drug monitoring.

^†^Atlantic includes New Brunswick, Nova Scotia, Prince Edward Island, Newfoundland and Labrador.

## Data Availability

The data sharing policy of Janssen Pharmaceutical Companies of Johnson & Johnson is available at https://www.janssen.com/clinical-trials/transparency. Requests for access to the study data can be submitted through the Yale Open Data Access (YODA) Project site at https://yoda.yale.edu.

## Supporting Information

The Supporting Information section includes the following materials related to the overall study population analysis: Supporting Information Figure 1a shows the overall study population analysis decision tree to select a time-dependent Cox proportional hazards model for interpretation; Supporting Information Figure 2 shows a flowchart of patient selection. Supporting Information Figure 3 shows KM estimates of persistence (sensitivity analysis); Supporting Information Table 1 shows comparisons of persistence after TDM and before any TDM (province interaction); and Supporting Information Table 10a shows descriptions of the overall study population analysis time-dependent Cox proportional hazards models. The Supporting Information section includes the following materials related to the subgroup population analysis: Supporting Information Text 1 includes additional text describing the impact of TDM-associated dose optimization on persistence in subgroup subpopulations of interest; Supporting Information Figure 1b shows the subgroup analysis decision tree to select a time-dependent Cox proportional hazards model for interpretation; Supporting Information Figure 4 shows comparisons of persistence in patients with CD or UC that received or did not receive dose optimization based on low or high-threshold criteria prior to first TDM; Supporting Information Figure 5 shows comparisons of persistence in patients that did not receive dose optimization based on low-threshold criteria prior to first TDM (sensitivity analysis excluding patients with no treatment after TDM); and Supporting Information Table 10b shows descriptions of the subgroup analysis' time-dependent Cox proportional hazards models. In addition, the following materials related to the subgroup population analysis are included: Supporting Information Tables 2–9 show demographic and baseline characteristics; Supporting Information Tables 11–18 show results of time-dependent Cox proportional hazards models; Supporting Information Tables 19–22 show results of quantitative bias analyze;, and Supporting Information Tables 23–24 show results of sensitivity analyses for all subgroup subpopulations and patient subsets evaluated. The Supporting Information section also includes a completed STROBE checklist.
