# Supplementary material for: Therapeutic Drug Monitoring for Dose Optimization of Infliximab in Patients With Inflammatory Bowel Disease: An Analysis of Canadian Real-World Data
Source: Can J Gastroenterol Hepatol. 2025 Feb 6;2025:5713315. doi: 10.1155/cjgh/5713315 (PMC11825194; doi:10.1155/cjgh/5713315)
Supplement: Supporting Information — Additional supporting information can be found online in the Supporting Information section. [file 5713315.f1.docx]

**SUPPLEMENTARY MATERIALS**

**Title:**

Therapeutic drug monitoring for dose optimization of infliximab in patients with inflammatory bowel disease: an analysis of Canadian real-world data

**Authors:**

David C Sealey^1^, Kai Fai Ho^2^, Z Christina Zhou^1^, Michael Clark^3^, Brian G Feagan^4,5^, Remo Panaccione^6^, A Hillary Steinhart^7,8^, Elena Bolshtyansky^3^, Martin Williamson^1^, Waqqas Afif^9,*^

**Author affiliations:**

^1^Johnson & Johnson, Toronto, Ontario, Canada, ^2^STAT-TU Inc., Elora, Ontario, Canada, ^3^Johnson & Johnson, New Brunswick, New Jersey, United States, ^4^Departments of Medicine and Epidemiology and Biostatistics Western University, London, Ontario, Canada, ^5^Alimentiv, London, Ontario, Canada, ^6^Division of Gastroenterology and Hepatology, University of Calgary, Calgary, Alberta, Canada, ^7^Zane Cohen Centre for Digestive Diseases, Mount Sinai Hospital, Toronto, Ontario, Canada, ^8^Division of Gastroenterology & Hepatology, Temerty Faculty of Medicine, University of Toronto, Toronto, Ontario, Canada, ^9^Division of Gastroenterology, McGill University Health Centre, Montreal, Québec, Canada

***Corresponding author:**

Waqqas Afif

**Overall Study Population Analysis**

*Figures*

Overall Study Population Analysis Decision Tree to Select Time-Dependent Cox Proportional
Hazards Model for Interpretation Suppl Figure 1a

Flow Chart of Patient Selection Suppl Figure 2

Kaplan-Meier Estimates of Persistence (Sensitivity Analysis) Suppl Figure 3

*Tables*

Comparison of Persistence After TDM and Before Any TDM (Province Interaction)

in Patients with Crohn’s Disease Suppl Table 1a

in Patients with Ulcerative Colitis Suppl Table 1b

Overall Study Population Analysis Time-Dependent Cox Proportional Hazards Models Suppl Table 10a

**Subgroup Population Analysis**

*Text*

Impact of TDM-Associated Dose Optimization on Persistence in Subgroup Subpopulations of Interest Suppl Text 1

*Figures*

Subgroup Analysis Decision Tree to Select Time-Dependent Cox Proportional
Hazards Model for Interpretation Suppl Figure 1b

Comparisons of Persistence in Patients with:

Crohn’s Disease that did not Receive Dose Optimization Based on
Low Threshold Criteria Prior to First TDM Suppl Figure 4a

Crohn’s Disease that did not Receive Dose Optimization Based on
High Threshold Criteria Prior to First TDM Suppl Figure 4b

Ulcerative Colitis that did not Receive Dose Optimization Based on
Low Threshold Criteria Prior to First TDM Suppl Figure 4c

Ulcerative Colitis that did not Receive Dose Optimization Based on
High Threshold Criteria Prior to First TDM Suppl Figure 4d

Crohn’s Disease that Received Dose Optimization Based on
Low Threshold Criteria Prior to First TDM Suppl Figure 4e

Crohn’s Disease that Received Dose Optimization Based on
High Threshold Criteria Prior to First TDM Suppl Figure 4f

Ulcerative Colitis that Received Dose Optimization Based on
Low Threshold Criteria Prior to First TDM Suppl Figure 4g

Ulcerative Colitis that Received Dose Optimization Based on
High Threshold Criteria Prior to First TDM Suppl Figure 4h

Comparisons of Persistence in Patients that did not Receive Dose Optimization Based on Low Threshold Criteria Prior to First TDM: Sensitivity Analysis Excluding Patients with no Treatment after TDM Suppl Figure 5

*Tables*

Subgroup Analysis Time-Dependent Cox Proportional Hazards Models *Suppl Table 10b*

| Serum IFX  Threshold | Post-Index Period  for DO | Post-TDM DO Criteria | Demographics and Baseline Characteristics | Time-Dependent  Cox Proportional  Hazards Model | Quantitative  Bias Analysis | Sensitivity Analysis |
| --- | --- | --- | --- | --- | --- | --- |
| **Subpopulation: Crohn’s disease; No DO (Based on Low Threshold Criteria) Prior to TDM** | | | | | | |
| 3 μg/mL | +9 weeks | Low Threshold | Suppl Table 2a | Suppl Table 11a | Suppl Table 19 | Suppl Table 23a |
| 3 μg/mL | +17 weeks | Low Threshold | Suppl Table 2b | Suppl Table 11b | - | Suppl Table 23b |
| 5 μg/mL | +9 weeks | Low Threshold | Suppl Table 2c | Suppl Table 11c | - | Suppl Table 23c |
| 5 μg/mL | +17 weeks | Low Threshold | Suppl Table 2d | Suppl Table 11d | - | Suppl Table 23d |
| 10 μg/mL | +9 weeks | Low Threshold | Suppl Table 2e | Suppl Table 11e | - | - |
| 10 μg/mL | +17 weeks | Low Threshold | Suppl Table 2f | Suppl Table 11f | - | - |
| 3 μg/mL | +9 weeks | High Threshold | Suppl Table 2g | Suppl Table 11g | - | - |
| 3 μg/mL | +17 weeks | High Threshold | Suppl Table 2h | Suppl Table 11h | - | - |
| 5 μg/mL | +9 weeks | High Threshold | Suppl Table 2i | Suppl Table 11i | - | - |
| 5 μg/mL | +17 weeks | High Threshold | Suppl Table 2j | Suppl Table 11j | - | - |
| 10 μg/mL | +9 weeks | High Threshold | Suppl Table 2k | Suppl Table 11k | - | - |
| 10 μg/mL | +17 weeks | High Threshold | Suppl Table 2l | Suppl Table 11l | - | - |
| **Subpopulation: Crohn’s disease; No DO (Based on High Threshold Criteria) Prior to TDM** | | | | | | |
| 3 μg/mL | +9 weeks | Low Threshold | Suppl Table 3a | Suppl Table 12a | - | - |
| 3 μg/mL | +17 weeks | Low Threshold | Suppl Table 3b | Suppl Table 12b | - | - |
| 5 μg/mL | +9 weeks | Low Threshold | Suppl Table 3c | Suppl Table 12c | - | - |
| 5 μg/mL | +17 weeks | Low Threshold | Suppl Table 3d | Suppl Table 12d | - | - |
| 10 μg/mL | +9 weeks | Low Threshold | Suppl Table 3e | Suppl Table 12e | - | - |
| 10 μg/mL | +17 weeks | Low Threshold | Suppl Table 3f | Suppl Table 12f | - | - |
| 3 μg/mL | +9 weeks | High Threshold | Suppl Table 3g | Suppl Table 12g | - | - |
| 3 μg/mL | +17 weeks | High Threshold | Suppl Table 3h | Suppl Table 12h | - | - |
| 5 μg/mL | +9 weeks | High Threshold | Suppl Table 3i | Suppl Table 12i | - | - |
| 5 μg/mL | +17 weeks | High Threshold | Suppl Table 3j | Suppl Table 12j | - | - |
| 10 μg/mL | +9 weeks | High Threshold | Suppl Table 3k | Suppl Table 12k | - | - |
| 10 μg/mL | +17 weeks | High Threshold | Suppl Table 3l | Suppl Table 12l | - | - |

DO=dose optimization; IFX=infliximab; TDM=therapeutic drug monitoring.

| Serum IFX  Threshold | Post-Index Period  for DO | Post-TDM DO Criteria | Demographics and Baseline Characteristics | Time-Dependent  Cox Proportional  Hazards Model | Quantitative  Bias Analysis | Sensitivity Analysis |
| --- | --- | --- | --- | --- | --- | --- |
| **Subpopulation: Crohn’s disease; DO (Based on Low Threshold Criteria) Prior to TDM** | | | | | | |
| 3 μg/mL | +9 weeks | Low Threshold | Suppl Table 6a | Suppl Table 15a | - | - |
| 3 μg/mL | +17 weeks | Low Threshold | Suppl Table 6b | Suppl Table 15b | - | - |
| 5 μg/mL | +9 weeks | Low Threshold | Suppl Table 6c | Suppl Table 15c | - | - |
| 5 μg/mL | +17 weeks | Low Threshold | Suppl Table 6d | Suppl Table 15d | - | - |
| 10 μg/mL | +9 weeks | Low Threshold | Suppl Table 6e | Suppl Table 15e | - | - |
| 10 μg/mL | +17 weeks | Low Threshold | Suppl Table 6f | Suppl Table 15f | - | - |
| 3 μg/mL | +9 weeks | High Threshold | Suppl Table 6g | Suppl Table 15g | - | - |
| 3 μg/mL | +17 weeks | High Threshold | Suppl Table 6h | Suppl Table 15h | - | - |
| 5 μg/mL | +9 weeks | High Threshold | Suppl Table 6i | Suppl Table 15i | - | - |
| 5 μg/mL | +17 weeks | High Threshold | Suppl Table 6j | Suppl Table 15j | - | - |
| 10 μg/mL | +9 weeks | High Threshold | Suppl Table 6k | Suppl Table 15k | - | - |
| 10 μg/mL | +17 weeks | High Threshold | Suppl Table 6l | Suppl Table 15l | - | - |
| **Subpopulation: Crohn’s disease; DO (Based on High Threshold Criteria) Prior to TDM** | | | | | | |
| 3 μg/mL | +9 weeks | Low Threshold | Suppl Table 7a | Suppl Table 16a | Suppl Table 21 | - |
| 3 μg/mL | +17 weeks | Low Threshold | Suppl Table 7b | Suppl Table 16b | - | - |
| 5 μg/mL | +9 weeks | Low Threshold | Suppl Table 7c | Suppl Table 16c | - | - |
| 5 μg/mL | +17 weeks | Low Threshold | Suppl Table 7d | Suppl Table 16d | - | - |
| 10 μg/mL | +9 weeks | Low Threshold | Suppl Table 7e | Suppl Table 16e | - | - |
| 10 μg/mL | +17 weeks | Low Threshold | Suppl Table 7f | Suppl Table 16f | - | - |
| 3 μg/mL | +9 weeks | High Threshold | Suppl Table 7g | Suppl Table 16g | - | - |
| 3 μg/mL | +17 weeks | High Threshold | Suppl Table 7h | Suppl Table 16h | - | - |
| 5 μg/mL | +9 weeks | High Threshold | Suppl Table 7i | Suppl Table 16i | - | - |
| 5 μg/mL | +17 weeks | High Threshold | Suppl Table 7j | Suppl Table 16j | - | - |
| 10 μg/mL | +9 weeks | High Threshold | Suppl Table 7k | Suppl Table 16k | - | - |
| 10 μg/mL | +17 weeks | High Threshold | Suppl Table 7l | Suppl Table 16l | - | - |

DO=dose optimization; IFX=infliximab; TDM=therapeutic drug monitoring.

| Serum IFX  Threshold | Post-Index Period  for DO | Post-TDM DO Criteria | Demographics and Baseline Characteristics | Time-Dependent  Cox Proportional  Hazards Model | Quantitative  Bias Analysis | Sensitivity Analysis |
| --- | --- | --- | --- | --- | --- | --- |
| **Subpopulation: Ulcerative Colitis; No DO (Based on Low Threshold Criteria) Prior to TDM** | | | | | | |
| 3 μg/mL | +9 weeks | Low Threshold | Suppl Table 4a | Suppl Table 13a | Suppl Table 20 | Suppl Table 24a |
| 3 μg/mL | +17 weeks | Low Threshold | Suppl Table 4b | Suppl Table 13b | - | Suppl Table 24b |
| 5 μg/mL | +9 weeks | Low Threshold | Suppl Table 4c | Suppl Table 13c | - | Suppl Table 24c |
| 5 μg/mL | +17 weeks | Low Threshold | Suppl Table 4d | Suppl Table 13d | - | Suppl Table 24d |
| 10 μg/mL | +9 weeks | Low Threshold | Suppl Table 4e | Suppl Table 13e | - | - |
| 10 μg/mL | +17 weeks | Low Threshold | Suppl Table 4f | Suppl Table 13f | - | - |
| 3 μg/mL | +9 weeks | High Threshold | Suppl Table 4g | Suppl Table 13g | - | - |
| 3 μg/mL | +17 weeks | High Threshold | Suppl Table 4h | Suppl Table 13h | - | - |
| 5 μg/mL | +9 weeks | High Threshold | Suppl Table 4i | Suppl Table 13i | - | - |
| 5 μg/mL | +17 weeks | High Threshold | Suppl Table 4j | Suppl Table 13j | - | - |
| 10 μg/mL | +9 weeks | High Threshold | Suppl Table 4k | Suppl Table 13k | - | - |
| 10 μg/mL | +17 weeks | High Threshold | Suppl Table 4l | Suppl Table 13l | - | - |
| **Subpopulation: Ulcerative Colitis; No DO (Based on High Threshold Criteria) Prior to TDM** | | | | | | |
| 3 μg/mL | +9 weeks | Low Threshold | Suppl Table 5a | Suppl Table 14a | - | - |
| 3 μg/mL | +17 weeks | Low Threshold | Suppl Table 5b | Suppl Table 14b | - | - |
| 5 μg/mL | +9 weeks | Low Threshold | Suppl Table 5c | Suppl Table 14c | - | - |
| 5 μg/mL | +17 weeks | Low Threshold | Suppl Table 5d | Suppl Table 14d | - | - |
| 10 μg/mL | +9 weeks | Low Threshold | Suppl Table 5e | Suppl Table 14e | - | - |
| 10 μg/mL | +17 weeks | Low Threshold | Suppl Table 5f | Suppl Table 14f | - | - |
| 3 μg/mL | +9 weeks | High Threshold | Suppl Table 5g | Suppl Table 14g | - | - |
| 3 μg/mL | +17 weeks | High Threshold | Suppl Table 5h | Suppl Table 14h | - | - |
| 5 μg/mL | +9 weeks | High Threshold | Suppl Table 5i | Suppl Table 14i | - | - |
| 5 μg/mL | +17 weeks | High Threshold | Suppl Table 5j | Suppl Table 14j | - | - |
| 10 μg/mL | +9 weeks | High Threshold | Suppl Table 5k | Suppl Table 14k | - | - |
| 10 μg/mL | +17 weeks | High Threshold | Suppl Table 5l | Suppl Table 14l | - | - |

DO=dose optimization; IFX=infliximab; TDM=therapeutic drug monitoring.

| Serum IFX  Threshold | Post-Index Period  for DO | Post-TDM DO Criteria | Demographics and Baseline Characteristics | Time-Dependent  Cox Proportional  Hazards Model | Quantitative  Bias Analysis | Sensitivity Analysis |
| --- | --- | --- | --- | --- | --- | --- |
| **Subpopulation: Ulcerative Colitis; DO (Based on Low Threshold Criteria) Prior to TDM** | | | | | | |
| 3 μg/mL | +9 weeks | Low Threshold | Suppl Table 8a | Suppl Table 17a | - | - |
| 3 μg/mL | +17 weeks | Low Threshold | Suppl Table 8b | Suppl Table 17b | - | - |
| 5 μg/mL | +9 weeks | Low Threshold | Suppl Table 8c | Suppl Table 17c | - | - |
| 5 μg/mL | +17 weeks | Low Threshold | Suppl Table 8d | Suppl Table 17d | - | - |
| 10 μg/mL | +9 weeks | Low Threshold | Suppl Table 8e | Suppl Table 17e | - | - |
| 10 μg/mL | +17 weeks | Low Threshold | Suppl Table 8f | Suppl Table 17f | - | - |
| 3 μg/mL | +9 weeks | High Threshold | Suppl Table 8g | Suppl Table 17g | - | - |
| 3 μg/mL | +17 weeks | High Threshold | Suppl Table 8h | Suppl Table 17h | - | - |
| 5 μg/mL | +9 weeks | High Threshold | Suppl Table 8i | Suppl Table 17i | - | - |
| 5 μg/mL | +17 weeks | High Threshold | Suppl Table 8j | Suppl Table 17j | - | - |
| 10 μg/mL | +9 weeks | High Threshold | Suppl Table 8k | Suppl Table 17k | - | - |
| 10 μg/mL | +17 weeks | High Threshold | Suppl Table 8l | Suppl Table 17l | - | - |
| **Subpopulation: Ulcerative Colitis; DO (Based on High Threshold Criteria) Prior to TDM** | | | | | | |
| 3 μg/mL | +9 weeks | Low Threshold | Suppl Table 9a | Suppl Table 18a | Suppl Table 22 | - |
| 3 μg/mL | +17 weeks | Low Threshold | Suppl Table 9b | Suppl Table 18b | - | - |
| 5 μg/mL | +9 weeks | Low Threshold | Suppl Table 9c | Suppl Table 18c | - | - |
| 5 μg/mL | +17 weeks | Low Threshold | Suppl Table 9d | Suppl Table 18d | - | - |
| 10 μg/mL | +9 weeks | Low Threshold | Suppl Table 9e | Suppl Table 18e | - | - |
| 10 μg/mL | +17 weeks | Low Threshold | Suppl Table 9f | Suppl Table 18f | - | - |
| 3 μg/mL | +9 weeks | High Threshold | Suppl Table 9g | Suppl Table 18g | - | - |
| 3 μg/mL | +17 weeks | High Threshold | Suppl Table 9h | Suppl Table 18h | - | - |
| 5 μg/mL | +9 weeks | High Threshold | Suppl Table 9i | Suppl Table 18i | - | - |
| 5 μg/mL | +17 weeks | High Threshold | Suppl Table 9j | Suppl Table 18j | - | - |
| 10 μg/mL | +9 weeks | High Threshold | Suppl Table 9k | Suppl Table 18k | - | - |
| 10 μg/mL | +17 weeks | High Threshold | Suppl Table 9l | Suppl Table 18l | - | - |

DO=dose optimization; IFX=infliximab; TDM=therapeutic drug monitoring.

**General**

The Strengthening the Reporting of Observational Studies in Epidemiology (STROBE) Checklist

**Supplementary Text**

Suppl Text 1: Impact of TDM-Associated Dose Optimization on Persistence in Subgroup Subpopulations of Interest

***Patients with No Dose Optimization Meeting Low-Threshold Criteria Prior to TDM***

If TDM-guided dose optimization resulted in longer persistence, analysis of the subpopulation of patients without dose optimization meeting low-threshold criteria prior to TDM would have the greatest discriminatory power to observe such an effect. A total of 1,684 patients with Crohn’s disease and 1,045 patients with UC were included in this subpopulation. In Crohn’s disease, patient subsets differed significantly by mean age, age group, province, year of IFX treatment initiation, and mean time to first TDM (Suppl Table 2a; *P*<0.05). In UC, patient subsets differed significantly by mean age, year of IFX treatment initiation, mean time to first TDM, and year of first TDM (Suppl Table 4a; *P*<0.05).

Results of the main Cox proportional hazards models evaluating persistence are shown in Suppl Tables 11a-f and 13a-f, with the main subset comparisons of interest summarized in Figures 4a and 4b. The covariate of log(days to TDM) was associated with longer persistence in both Crohn’s disease and UC, indicating that as more time elapsed prior to the first TDM, treatment persistence after TDM was longer. Treatment initiation in 2018 was generally associated with longer persistence compared with 2015 and 2016 in Crohn’s disease, and with 2015, 2016 and 2017 in UC. Age <65 years was associated with longer persistence in UC.

In patients who did not have dose optimization within 9 weeks after first TDM, serum IFX ≥3 μg/mL (subset C) was associated with longer persistence than <3 μg/mL (subset A) (Crohn’s disease: HR 0.23, 95% CI 0.18, 0.30, Figure 4a, C vs. A; UC: HR 0.19, 95% CI 0.14, 0.26, Figure 4b). This observation was consistent in sensitivity analyses with serum IFX concentrations of 5 μg/mL or 10 μg/mL, and/or a post-TDM period of 17 weeks.

In patients with Crohn’s disease with serum IFX <3 μg/mL, dose optimization within 9 weeks after first TDM (subset B) was associated with longer persistence than no dose optimization (subset A) (HR 0.36, 95% CI 0.26, 0.50; Figure 4a, B vs. A). Assessment of demographic and baseline characteristic similarities and differences revealed covariates with either confounding (at least partially; year of IFX treatment initiation), competing (time to first TDM) or no expected impact on the differences in persistence observed. To assess the potential impact of measured and unmeasured confounders, models were developed in which the post-TDM persistence of patients who received dose optimization was reduced iteratively (Suppl Tables 19 and 20). After a mean/median post-TDM reduction in persistence of 193.3/250 days (equivalent to 3.5/4.5 8-week cycles of IFX treatment), the positive association was no longer significant (Suppl Table 19a). Therefore, confounding was considered unlikely to account for the positive association observed. A comprehensive analysis of the association of persistence with ATI could not be completed due to missing data (ATI not tested or not reported; see Methods). In subsets A and B, the proportions of patients who discontinued treatment and had ATI at first TDM were 69.3% and 48.1%, respectively; the proportions with no ATI data were 25.5% and 42.6%, respectively.

In patients with UC with serum IFX <3 μg/mL, dose optimization at 4 weeks (HR 0.36, 95% CI 0.25, 0.51) and 6 weeks (HR 0.30, 95% CI 0.21, 0.43) after first TDM (subset B) was associated with longer persistence than no dose optimization (subset A) (Figure 4b, B vs. A). Assessment of demographic and baseline characteristic similarities and differences revealed covariates with either confounding (at least partially; year of IFX treatment initiation), competing (time to first TDM) or no expected impact on the differences in persistence observed. Using modelling as described above, after a mean/median reduction of 144.8/175 days and 175.4/225 days in the post-TDM persistence of patients with dose optimization at 4 weeks and 6 weeks, respectively, the positive association was no longer significant (Suppl Table 20a). These reductions are equivalent to 2.6/3.1 and 3.1/4.0 8‑week cycles, respectively, of IFX treatment. Therefore, confounding was considered unlikely to account for the positive association observed. In subsets A and B, the proportions of patients who discontinued treatment and had ATI at first TDM were 75.7% and 36.2%, respectively; the proportions with no ATI data were 21.7% and 51.1%, respectively.

Sensitivity analyses conducted with a serum IFX concentration of 5 μg/mL and/or with a longer post-TDM period for dose optimization similarly found a significant association between post-TDM dose optimization and longer persistence (Figures 4a and 4b, B vs. A). When a serum IFX concentration of 10 μg/mL was used, dose optimization within 17 weeks after first TDM in patients with Crohn’s disease, or at 4 weeks after first TDM in patients with UC was not associated with longer persistence. However, the remaining sensitivity analyses conducted with a serum IFX concentration of 10 μg/mL found a significant association between post-TDM dose optimization and longer persistence.

In patients with Crohn’s disease, serum IFX ≥3 μg/mL followed by no dose optimization within 9 weeks (subset C) was associated with longer persistence than serum IFX <3 μg/mL followed by dose optimization (subset B) (HR 0.63, 95% CI 0.45, 0.88; Figure 4a, C vs. B). Similar results were observed in patients with UC (dose optimization at 4 weeks: HR 0.54, 95% CI 0.37, 0.79; dose optimization at 6 weeks: HR 0.65, 95% CI 0.44, 0.97; Figure 4b, C vs. B).

***Patients with Dose Optimization Meeting High-Threshold Criteria Prior to TDM***

Whether dose optimization prior to TDM leads to different persistence results than those observed in the subpopulation of patients with no dose optimization meeting low-threshold criteria prior to first TDM (as described above) is also of interest. A total of 342 patients with Crohn’s disease and 380 patients with UC were included in this subpopulation. In Crohn’s disease, patient subsets differed significantly by mean age, age group and province (Suppl Table 7a; *P*<0.05). In UC, patient subsets differed significantly by mean age, age group, year of IFX treatment initiation, and mean time to first TDM (Suppl Table 9a; *P*<0.05).

Results of the main Cox proportional hazards models evaluating persistence are shown in Suppl Tables 16a-f and 18a-f, with the main subset comparisons summarized in Suppl Figures 3f and 3h. In Crohn’s disease, a first recorded weight of 62 kg to <73 kg was associated with longer persistence than a weight of ≥86 kg. In UC, the covariates of log(days to TDM) and age <65 years were associated with longer persistence. Treatment initiation in 2018 was generally associated with longer persistence compared with 2016 in Crohn’s disease, and with 2015 and 2016 in UC.

In patients who did not have dose optimization within 9 weeks after first TDM, serum IFX ≥3 μg/mL (subset C) was associated with longer persistence than <3 μg/mL (subset A) (Crohn’s disease: HR 0.45, 95% CI, 0.27, 0.73, Suppl Figure 3f, C vs. A; UC: HR 0.51, 95% CI, 0.33, 0.79, Suppl Figure 3h). This observation was consistent in sensitivity analyses with serum IFX concentrations of 5 μg/mL or 10 μg/mL, and/or a post-TDM period of 17 weeks, except for serum IFX concentration of 10 μg/mL in patients with UC where no association was found.

In patients with Crohn’s disease with serum IFX <3 μg/mL, dose optimization within 9 weeks after TDM (subset B) was associated with a trend towards longer persistence that was not statistically significant compared with no dose optimization (subset A) (HR 0.56, 95% CI 0.30, 1.05; Suppl Figure 3f, B vs. A). In patients with UC with serum IFX <3 μg/mL, dose optimization within 9 weeks after TDM (subset B) was not significantly associated with longer persistence (HR 0.78, 95% CI 0.46, 1.31; Suppl Figure 3h, B vs. A). Results of the sensitivity analyses at serum IFX concentrations of 3 μg/mL,5 μg/mL or 10 μg/mL were similar. Assessment of demographic and baseline characteristics revealed covariates with no expected impact on the results. To assess the potential impact of unmeasured confounders on the lack of significant association with longer persistence in patients who received dose optimization, models were developed in which persistence in these patients was increased iteratively. After a mean/median increase of 23.7/25 days for Crohn’s disease (equivalent to 0.4 8-week cycles of IFX treatment) and 174.4/175 days for UC (equivalent to 3.1 8-week cycles of IFX treatment), post-TDM dose optimization was significantly associated with longer persistence (Suppl Tables 21a and 22a). Therefore, confounding was considered possible to account for the lack of significant association observed for Crohn’s disease, and unlikely for UC. In subsets A and B, the proportions of patients with Crohn’s disease who discontinued treatment and had ATI at first TDM were 45.8% and 12.5%, respectively; the proportions with no ATI data were 41.7% and 62.5%, respectively. The proportions of patients with UC who discontinued treatment and had ATI at first TDM were 64.5% and 23.1%, respectively; the proportions with no ATI data were 32.3% and 61.5%, respectively.

Serum IFX ≥3 μg/mL with no dose optimization after TDM (subset C) was not significantly associated with improved persistence compared with serum IFX <3 μg/mL followed by dose optimization (subset B) (Crohn’s disease: 0.80, 95% CI 0.41, 1.55; UC: 0.66, 95% CI 0.38, 1.16; Suppl Figures 3f and 3h, C vs. B). Results of sensitivity analyses were similar.

**Supplementary Figures**

Suppl Figure 1a: Overall Study Population Analysis Decision Tree to Select Time-Dependent Cox Proportional Hazards Model for Interpretation

TDM=therapeutic drug monitoring.

Four models were developed to evaluate interaction terms and the proportional hazards assumption, as summarized in Suppl Table 10a. The appropriate model was selected for interpretation according to the decision tree shown. Models for each primary analysis and sensitivity analysis were determined independently. The proportional hazards assumption was tested using the term: (TDM vs No TDM) * Log (time to TDM).

Suppl Figure 1b: Subgroup Analysis Decision Tree to Select Time-Dependent Cox Proportional Hazards Model for Interpretation

DO=dose optimization; TDM=therapeutic drug monitoring.

Four models were developed to evaluate interaction terms and the proportional hazards assumption, as summarized in Suppl Table 10b. The appropriate model was selected for interpretation according to the decision tree shown. Models for each primary analysis and sensitivity analysis were determined independently. The proportional hazards assumption was tested using the term: Dose optimization within post-TDM period (yes or no) * Log (time to dose optimization within post-TDM period).

Suppl Figure 2: Flow Chart of Patient Selection


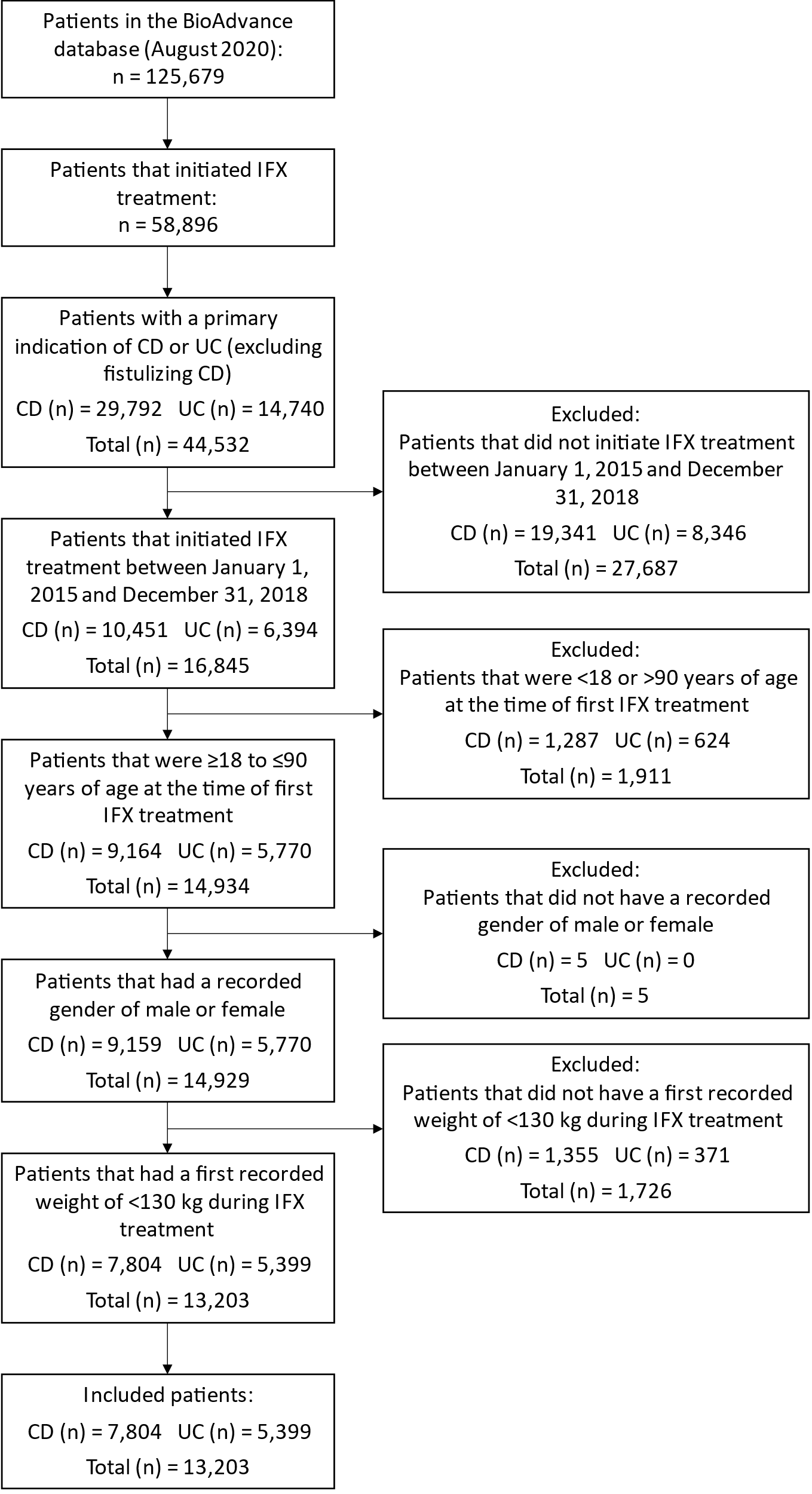


CD=Crohn’s disease; HR=hazard ratio; IFX=infliximab; UC=ulcerative colitis.

Suppl Figure 3: Kaplan-Meier Estimates of Persistence (Sensitivity Analysis)

a. Crohn’s Disease


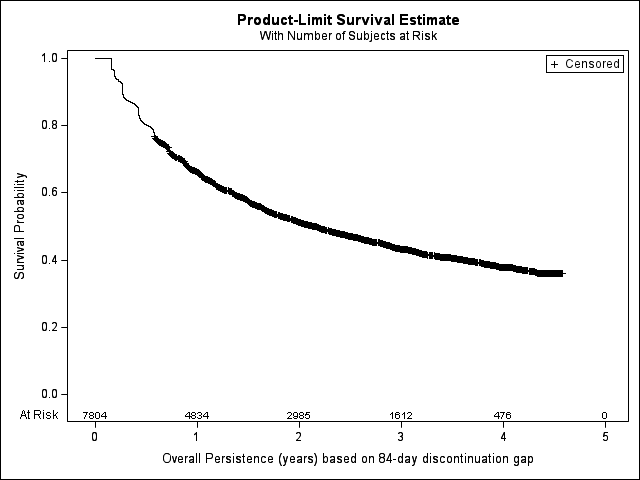


b. Ulcerative Colitis


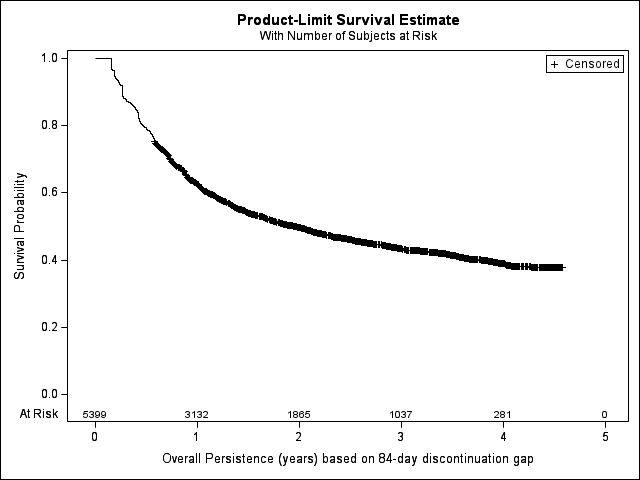


Suppl Figure 4a: Comparisons of Persistence in Patients with Crohn’s Disease that did not Receive Dose Optimization Based on Low Threshold Criteria Prior to First TDM


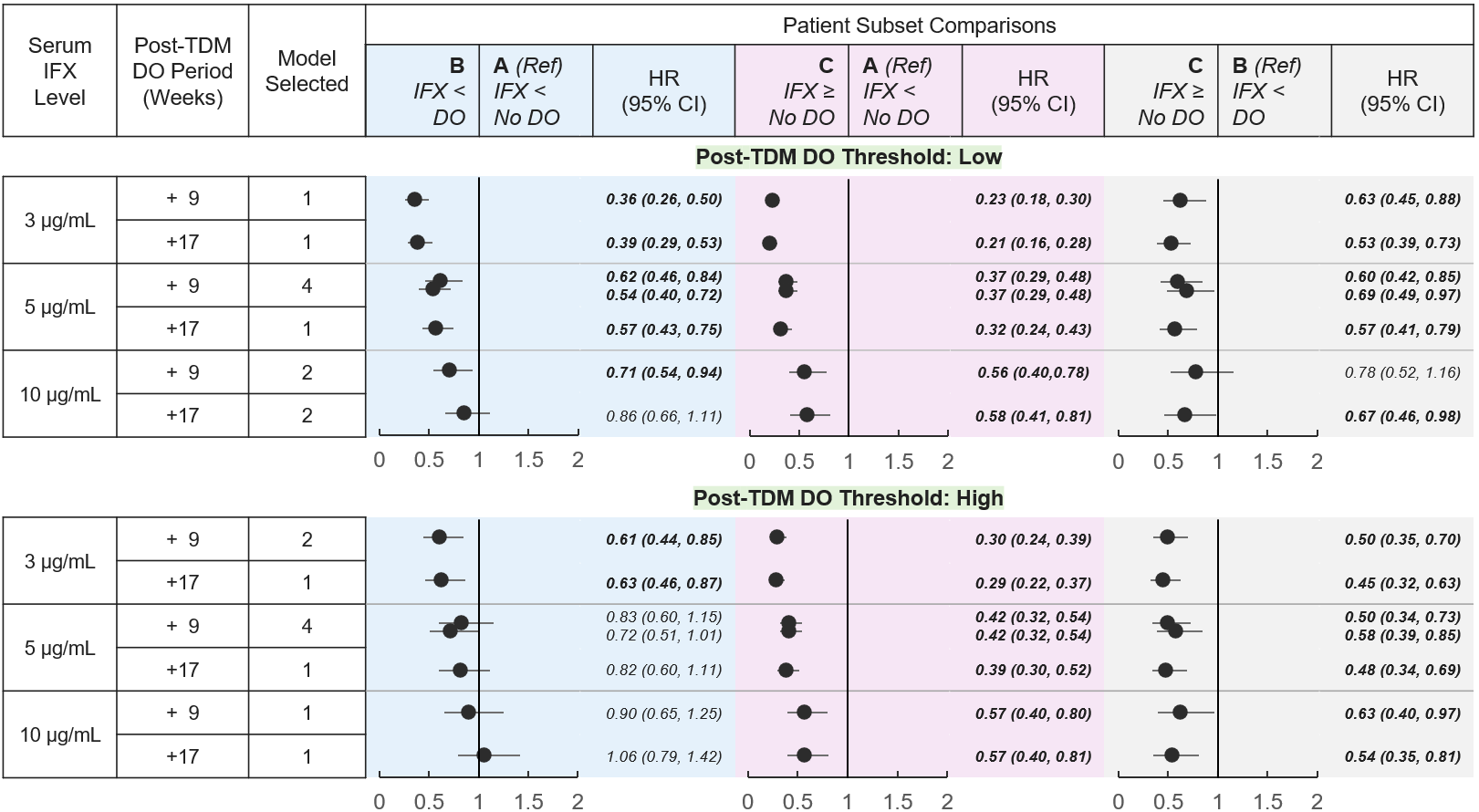


CI=confidence interval; DO=dose optimization; HR=hazard ratio; IFX=infliximab; Ref=reference; TDM=therapeutic drug monitoring.

Dose optimization thresholds: High: A treatment interval decrease of ≥11 days (1.57 weeks) with a posterior interval of ≤35 days (5 weeks), and/or a dose level increase of ≥1.5 mg/kg with a posterior dose level of ≥9 mg/kg. Low: A treatment interval decrease of ≥11 days (1.57 weeks) with a posterior interval of ≤46 days (6.57 weeks), and/or dose level increase of ≥1.5 mg/kg with a posterior dose level of ≥7 mg/kg. Model details are described in Suppl Table 10b. Model selection decision tree is shown in Suppl Figure 1b. Model results available in Suppl Table 11. HR (95% CI) displayed to a maximum of 2.0 and shown in bold when p<0.05. Doublet results based on DO at 4 weeks and 6 weeks, respectively.

A hazard ratio of <1 indicates a persistence advantage over the reference. If the CI includes 1, there is no statistically significant difference in persistence. If the CI does not include 1, the difference in persistence is statistically significant.

A=First TDM serum IFX concentration below level and no dose optimization within post-TDM period;

B=First TDM serum IFX concentration below level and dose optimization within post-TDM period;

C=First TDM serum IFX concentration at or above level and no dose optimization within post-TDM period.

Suppl Figure 4b: Comparisons of Persistence in Patients with Crohn’s Disease that did not Receive Dose Optimization Based on High Threshold Criteria Prior to First TDM


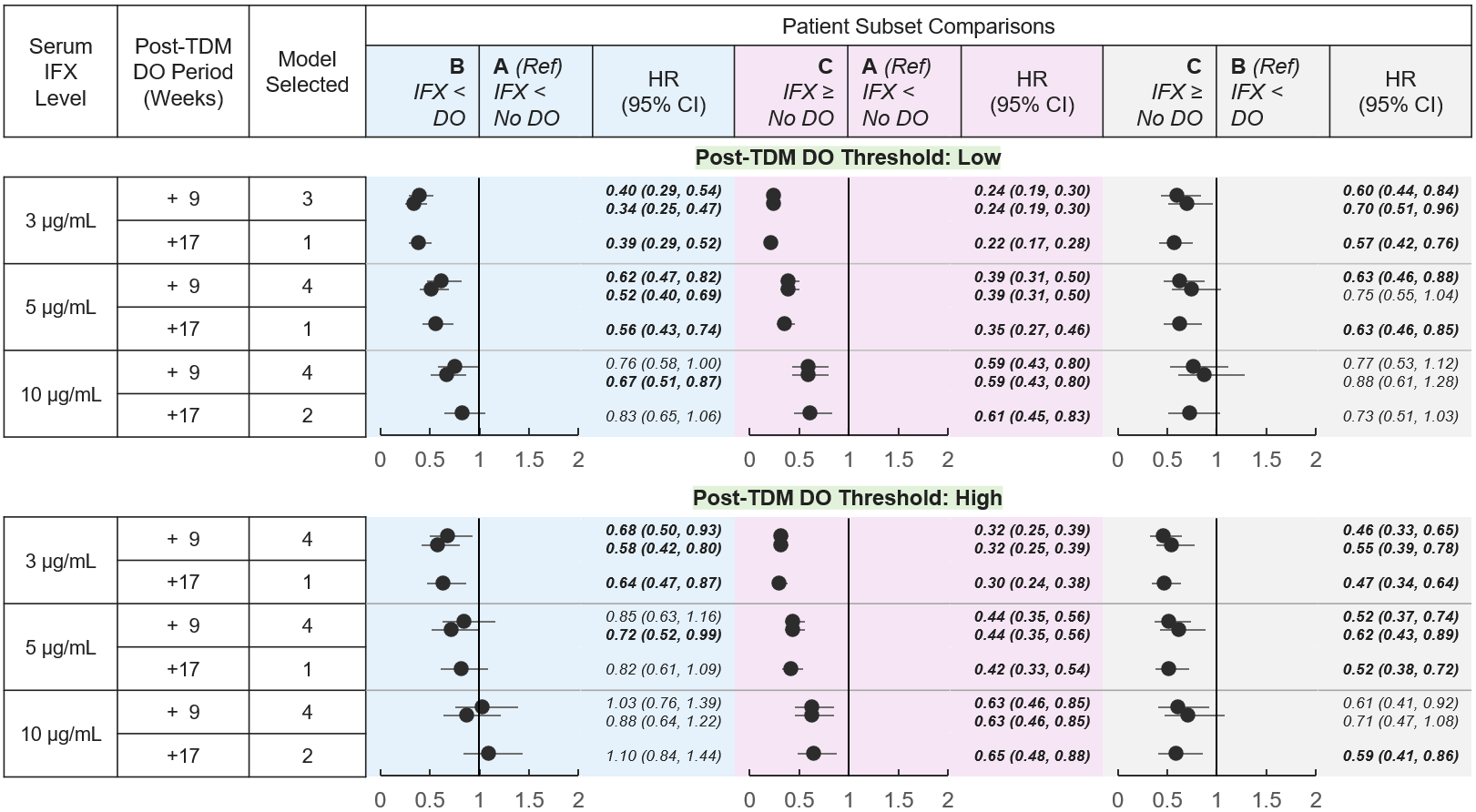


CI=confidence interval; DO=dose optimization; HR=hazard ratio; IFX=infliximab; Ref=reference; TDM=therapeutic drug monitoring.

Dose optimization thresholds: High: A treatment interval decrease of ≥11 days (1.57 weeks) with a posterior interval of ≤35 days (5 weeks), and/or a dose level increase of ≥1.5 mg/kg with a posterior dose level of ≥9 mg/kg. Low: A treatment interval decrease of ≥11 days (1.57 weeks) with a posterior interval of ≤46 days (6.57 weeks), and/or dose level increase of ≥1.5 mg/kg with a posterior dose level of ≥7 mg/kg. Model details are described in Suppl Table 10b. Model selection decision tree is shown in Suppl Figure 1b. Model results available in Suppl Table 12. HR (95% CI) displayed to a maximum of 2.0 and shown in bold when p<0.05. Doublet results based on DO at 4 weeks and 6 weeks, respectively.

A hazard ratio of <1 indicates a persistence advantage over the reference. If the CI includes 1, there is no statistically significant difference in persistence. If the CI does not include 1, the difference in persistence is statistically significant.

A=First TDM serum IFX concentration below level and no dose optimization within post-TDM period;

B=First TDM serum IFX concentration below level and dose optimization within post-TDM period;

C=First TDM serum IFX concentration at or above level and no dose optimization within post-TDM period.

Suppl Figure 4c: Comparisons of Persistence in Patients with Ulcerative Colitis that did not Receive Dose Optimization Based on Low Threshold Criteria Prior to First TDM


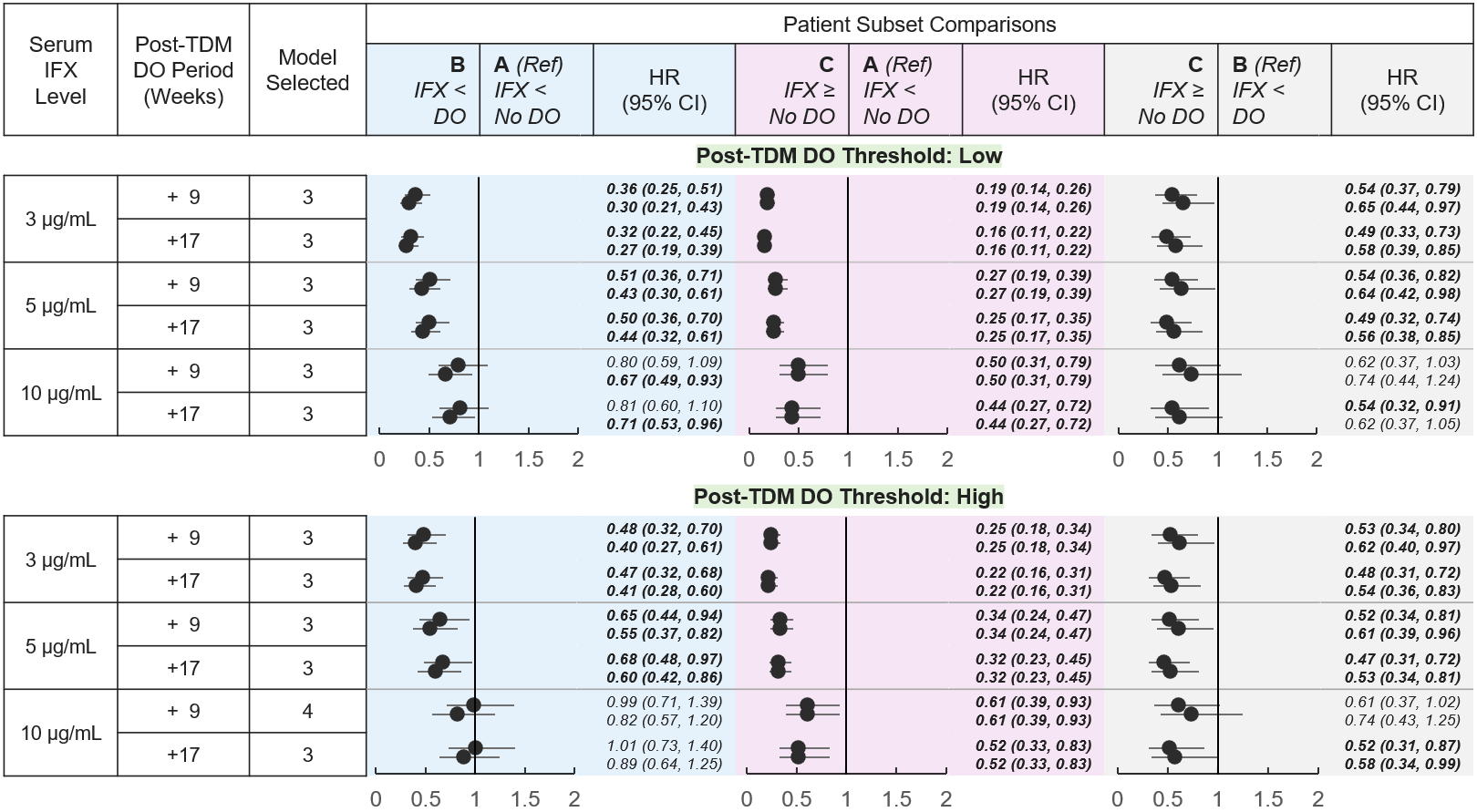


CI=confidence interval; DO=dose optimization; HR=hazard ratio; IFX=infliximab; Ref=reference; TDM=therapeutic drug monitoring.

Dose optimization thresholds: High: A treatment interval decrease of ≥11 days (1.57 weeks) with a posterior interval of ≤35 days (5 weeks), and/or a dose level increase of ≥1.5 mg/kg with a posterior dose level of ≥9 mg/kg. Low: A treatment interval decrease of ≥11 days (1.57 weeks) with a posterior interval of ≤46 days (6.57 weeks), and/or dose level increase of ≥1.5 mg/kg with a posterior dose level of ≥7 mg/kg. Model details are described in Suppl Table 10b. Model selection decision tree is shown in Suppl Figure 1b. Model results available in Suppl Table 13. HR (95% CI) displayed to a maximum of 2.0 and shown in bold when p<0.05. Doublet results based on DO at 4 weeks and 6 weeks, respectively.

A hazard ratio of <1 indicates a persistence advantage over the reference. If the CI includes 1, there is no statistically significant difference in persistence. If the CI does not include 1, the difference in persistence is statistically significant.

A=First TDM serum IFX concentration below level and no dose optimization within post-TDM period;

B=First TDM serum IFX concentration below level and dose optimization within post-TDM period;

C=First TDM serum IFX concentration at or above level and no dose optimization within post-TDM period.

Suppl Figure 4d: Comparisons of Persistence in Patients with Ulcerative Colitis that did not Receive Dose Optimization Based on High Threshold Criteria Prior to First TDM


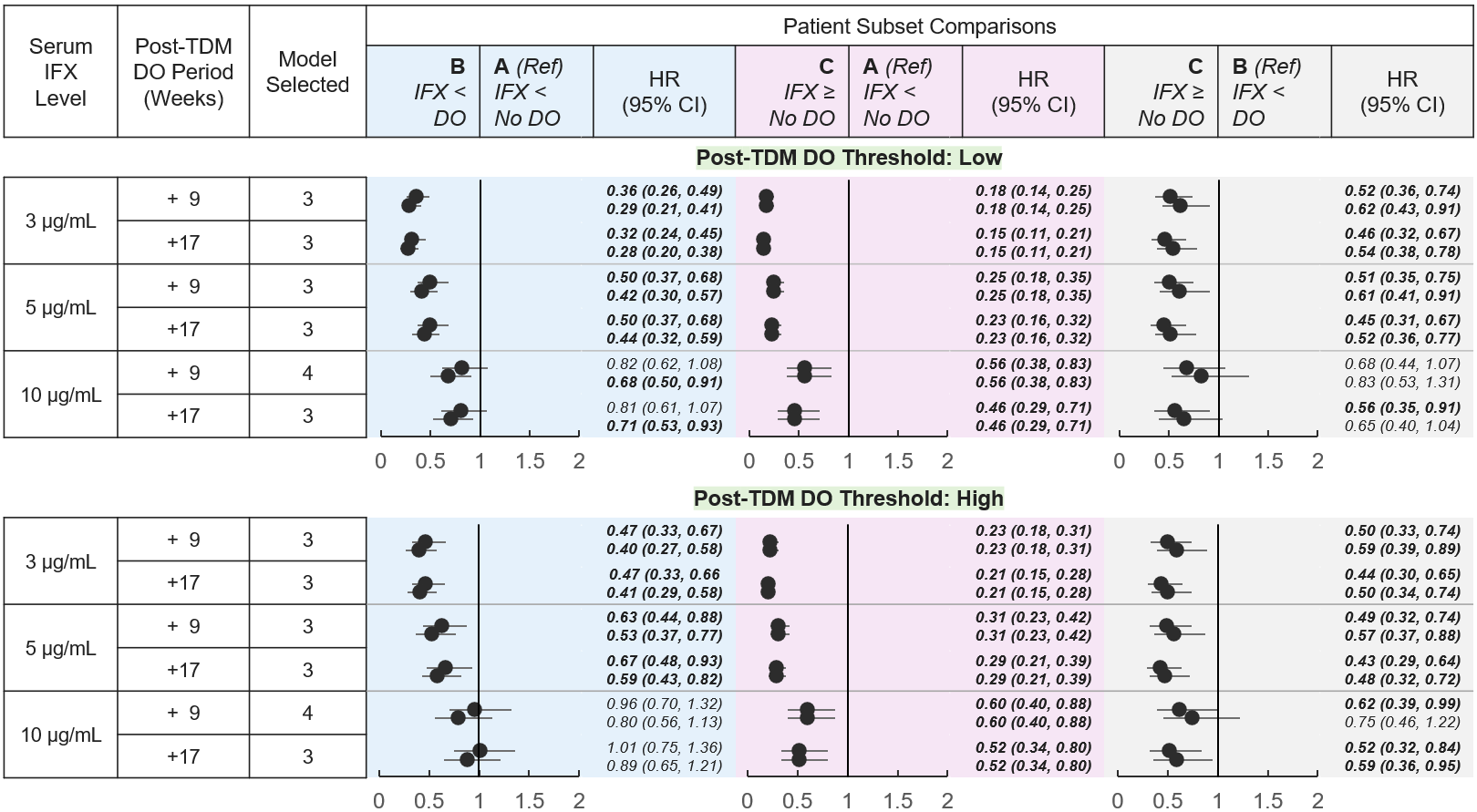


CI=confidence interval; DO=dose optimization; HR=hazard ratio; IFX=infliximab; Ref=reference; TDM=therapeutic drug monitoring.

Dose optimization thresholds: High: A treatment interval decrease of ≥11 days (1.57 weeks) with a posterior interval of ≤35 days (5 weeks), and/or a dose level increase of ≥1.5 mg/kg with a posterior dose level of ≥9 mg/kg. Low: A treatment interval decrease of ≥11 days (1.57 weeks) with a posterior interval of ≤46 days (6.57 weeks), and/or dose level increase of ≥1.5 mg/kg with a posterior dose level of ≥7 mg/kg. Model details are described in Suppl Table 10b. Model selection decision tree is shown in Suppl Figure 1b. Model results available in Suppl Table 14. HR (95% CI) displayed to a maximum of 2.0 and shown in bold when p<0.05. Doublet results based on DO at 4 weeks and 6 weeks, respectively.

A hazard ratio of <1 indicates a persistence advantage over the reference. If the CI includes 1, there is no statistically significant difference in persistence. If the CI does not include 1, the difference in persistence is statistically significant.

A=First TDM serum IFX concentration below level and no dose optimization within post-TDM period;

B=First TDM serum IFX concentration below level and dose optimization within post-TDM period;

C=First TDM serum IFX concentration at or above level and no dose optimization within post-TDM period.

Suppl Figure 4e: Comparisons of Persistence in Patients with Crohn’s Disease that Received Dose Optimization Based on Low Threshold Criteria Prior to First TDM


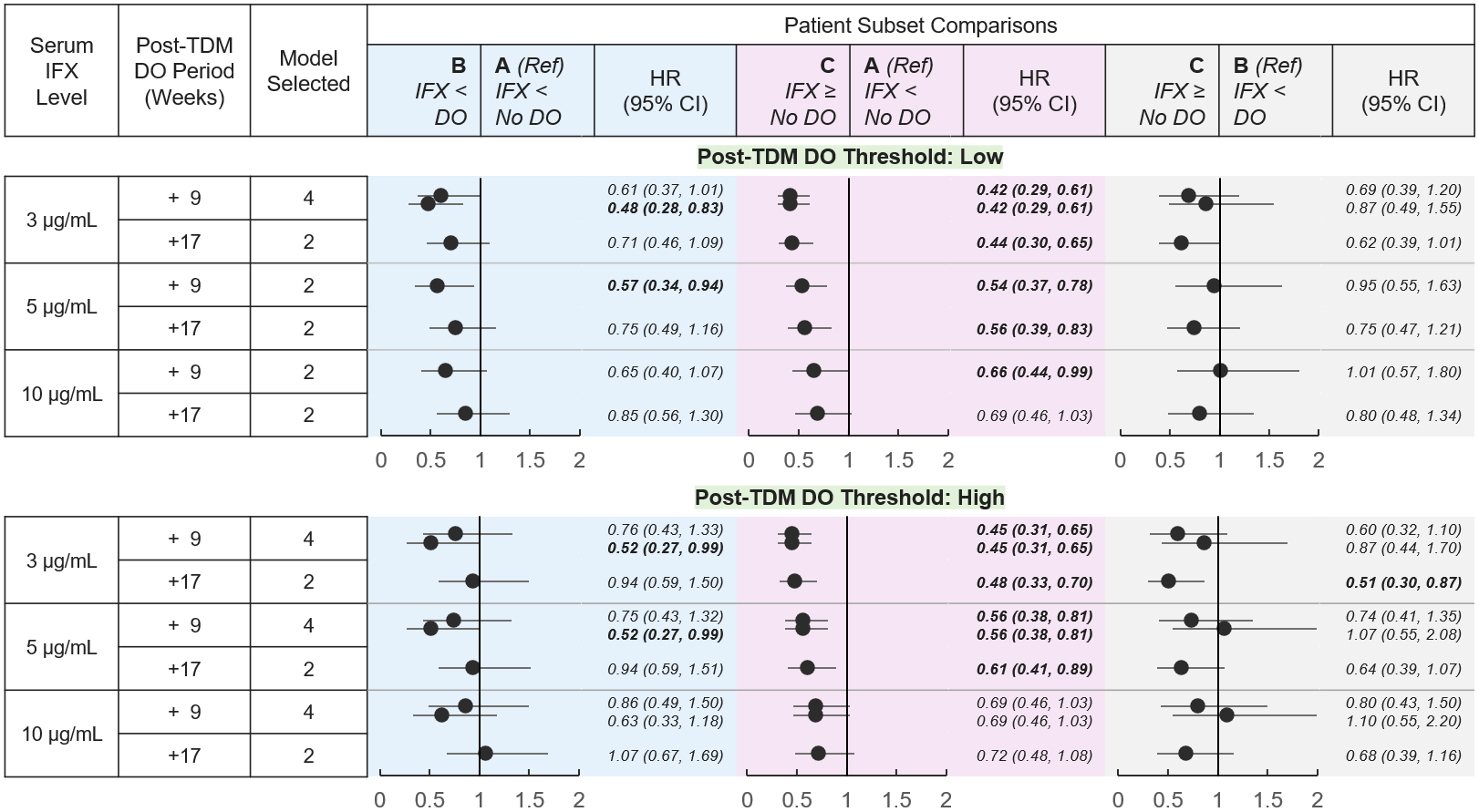


CI=confidence interval; DO=dose optimization; HR=hazard ratio; IFX=infliximab; Ref=reference; TDM=therapeutic drug monitoring.

Dose optimization thresholds: High: A treatment interval decrease of ≥11 days (1.57 weeks) with a posterior interval of ≤35 days (5 weeks), and/or a dose level increase of ≥1.5 mg/kg with a posterior dose level of ≥9 mg/kg. Low: A treatment interval decrease of ≥11 days (1.57 weeks) with a posterior interval of ≤46 days (6.57 weeks), and/or dose level increase of ≥1.5 mg/kg with a posterior dose level of ≥7 mg/kg. Model details are described in Suppl Table 10b. Model selection decision tree is shown in Suppl Figure 1b. Model results available in Suppl Table 15. HR (95% CI) displayed to a maximum of 2.0 and shown in bold when p<0.05. Doublet results based on DO at 4 weeks and 6 weeks, respectively.

A hazard ratio of <1 indicates a persistence advantage over the reference. If the CI includes 1, there is no statistically significant difference in persistence. If the CI does not include 1, the difference in persistence is statistically significant.

A=First TDM serum IFX concentration below level and no dose optimization within post-TDM period;

B=First TDM serum IFX concentration below level and dose optimization within post-TDM period;

C=First TDM serum IFX concentration at or above level and no dose optimization within post-TDM period.

Suppl Figure 4f: Comparisons of Persistence in Patients with Crohn’s Disease that Received Dose Optimization Based on High Threshold Criteria Prior to First TDM


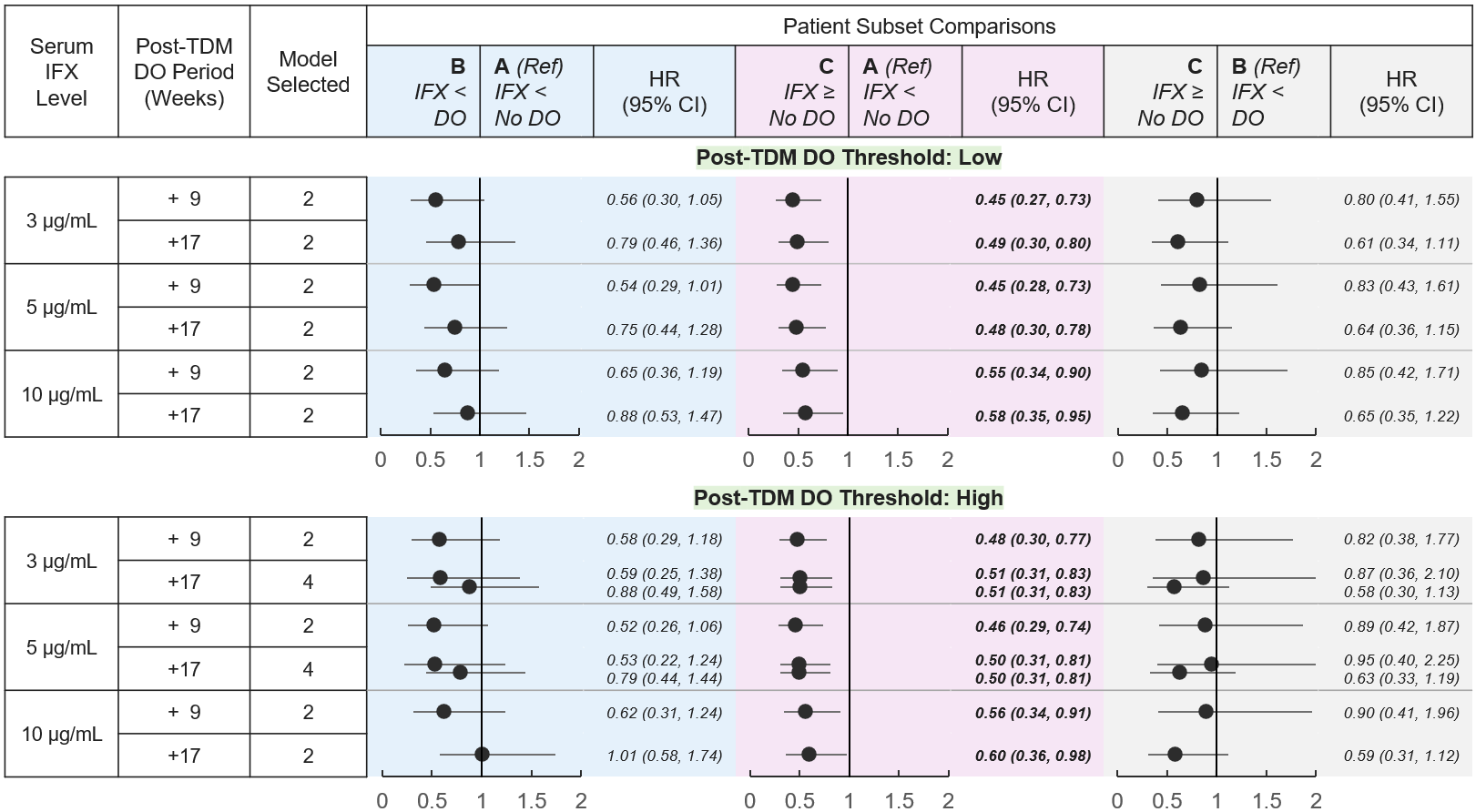


CI=confidence interval; DO=dose optimization; HR=hazard ratio; IFX=infliximab; Ref=reference; TDM=therapeutic drug monitoring.

Dose optimization thresholds: High: A treatment interval decrease of ≥11 days (1.57 weeks) with a posterior interval of ≤35 days (5 weeks), and/or a dose level increase of ≥1.5 mg/kg with a posterior dose level of ≥9 mg/kg. Low: A treatment interval decrease of ≥11 days (1.57 weeks) with a posterior interval of ≤46 days (6.57 weeks), and/or dose level increase of ≥1.5 mg/kg with a posterior dose level of ≥7 mg/kg. Model details are described in Suppl Table 10b. Model selection decision tree is shown in Suppl Figure 1b. Model results available in Suppl Table 16. HR (95% CI) displayed to a maximum of 2.0 and shown in bold when p<0.05. Doublet results based on DO at 4 weeks and 6 weeks, respectively.

A hazard ratio of <1 indicates a persistence advantage over the reference. If the CI includes 1, there is no statistically significant difference in persistence. If the CI does not include 1, the difference in persistence is statistically significant.

A=First TDM serum IFX concentration below level and no dose optimization within post-TDM period;

B=First TDM serum IFX concentration below level and dose optimization within post-TDM period;

C=First TDM serum IFX concentration at or above level and no dose optimization within post-TDM period.

Suppl Figure 4g: Comparisons of Persistence in Patients with Ulcerative Colitis that Received Dose Optimization Based on Low Threshold Criteria Prior to First TDM


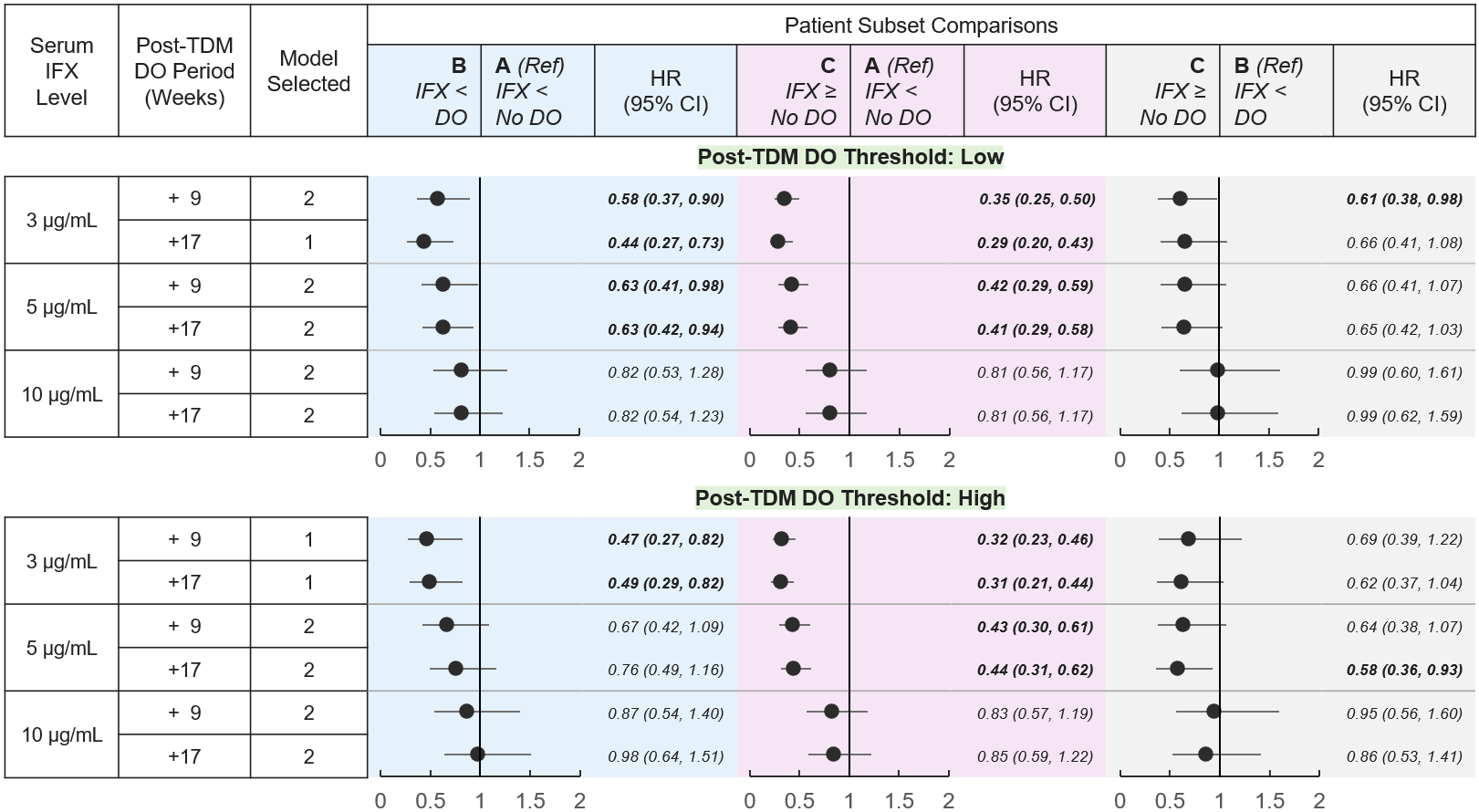


CI=confidence interval; DO=dose optimization; HR=hazard ratio; IFX=infliximab; Ref=reference; TDM=therapeutic drug monitoring.

Dose optimization thresholds: High: A treatment interval decrease of ≥11 days (1.57 weeks) with a posterior interval of ≤35 days (5 weeks), and/or a dose level increase of ≥1.5 mg/kg with a posterior dose level of ≥9 mg/kg. Low: A treatment interval decrease of ≥11 days (1.57 weeks) with a posterior interval of ≤46 days (6.57 weeks), and/or dose level increase of ≥1.5 mg/kg with a posterior dose level of ≥7 mg/kg. Model details are described in Suppl Table 10b. Model selection decision tree is shown in Suppl Figure 1b. Model results available in Suppl Table 17. HR (95% CI) displayed to a maximum of 2.0 and shown in bold when p<0.05. Doublet results based on DO at 4 weeks and 6 weeks, respectively.

A hazard ratio of <1 indicates a persistence advantage over the reference. If the CI includes 1, there is no statistically significant difference in persistence. If the CI does not include 1, the difference in persistence is statistically significant.

A=First TDM serum IFX concentration below level and no dose optimization within post-TDM period;

B=First TDM serum IFX concentration below level and dose optimization within post-TDM period;

C=First TDM serum IFX concentration at or above level and no dose optimization within post-TDM period.

Suppl Figure 4h: Comparisons of Persistence in Patients with Ulcerative Colitis that Received Dose Optimization Based on High Threshold Criteria Prior to First TDM


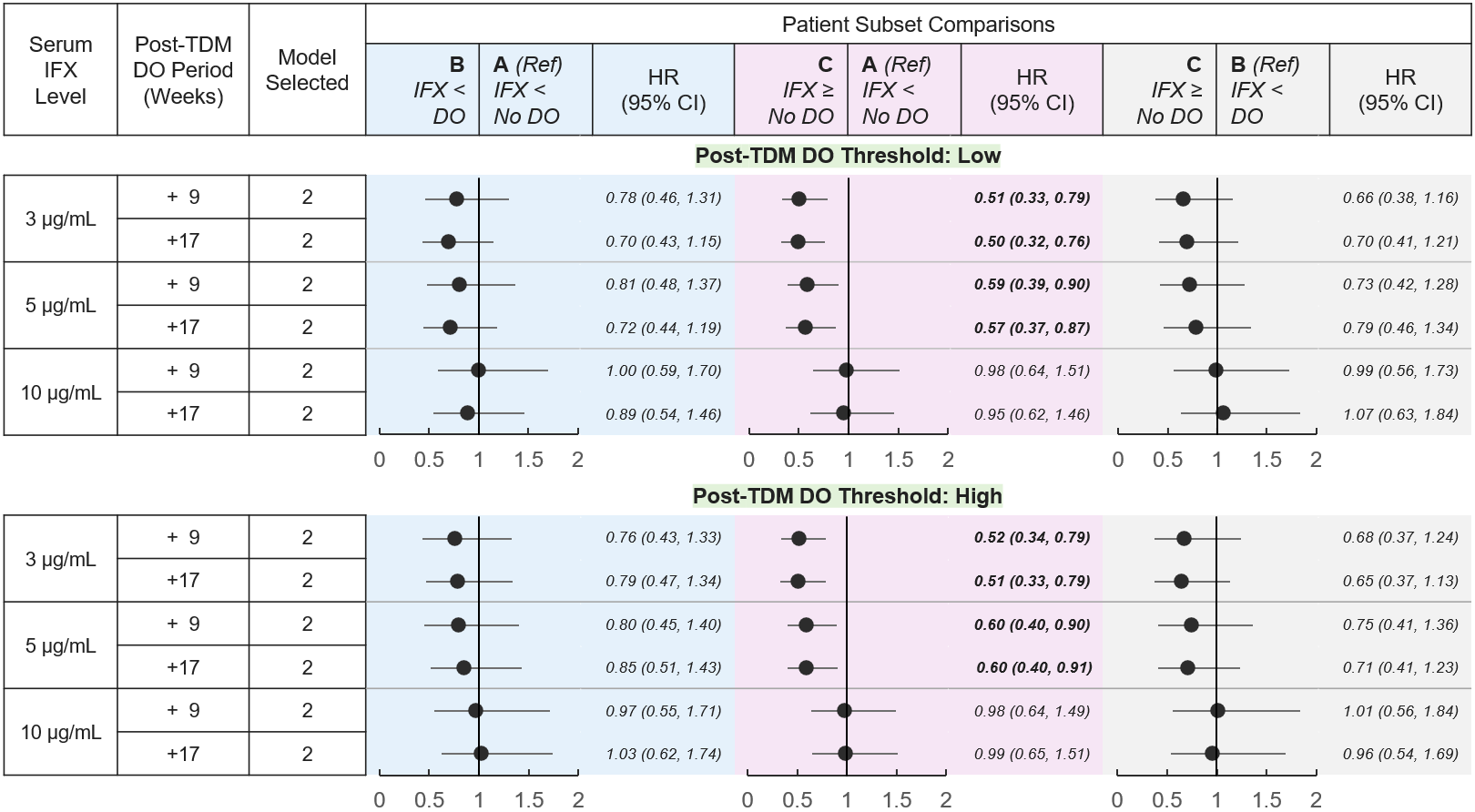


CI=confidence interval; DO=dose optimization; HR=hazard ratio; IFX=infliximab; Ref=reference; TDM=therapeutic drug monitoring.

Dose optimization thresholds: High: A treatment interval decrease of ≥11 days (1.57 weeks) with a posterior interval of ≤35 days (5 weeks), and/or a dose level increase of ≥1.5 mg/kg with a posterior dose level of ≥9 mg/kg. Low: A treatment interval decrease of ≥11 days (1.57 weeks) with a posterior interval of ≤46 days (6.57 weeks), and/or dose level increase of ≥1.5 mg/kg with a posterior dose level of ≥7 mg/kg. Model details are described in Suppl Table 10b. Model selection decision tree is shown in Suppl Figure 1b. Model results available in Suppl Table 18. HR (95% CI) displayed to a maximum of 2.0 and shown in bold when p<0.05. Doublet results based on DO at 4 weeks and 6 weeks, respectively.

A hazard ratio of <1 indicates a persistence advantage over the reference. If the CI includes 1, there is no statistically significant difference in persistence. If the CI does not include 1, the difference in persistence is statistically significant.

A=First TDM serum IFX concentration below level and no dose optimization within post-TDM period;

B=First TDM serum IFX concentration below level and dose optimization within post-TDM period;

C=First TDM serum IFX concentration at or above level and no dose optimization within post-TDM period.

Suppl Figure 5: Comparisons of Persistence in Patients that did not Receive Dose Optimization Based on Low Threshold Criteria Prior to First TDM: Sensitivity Analysis Excluding Patients with no Treatment after TDM


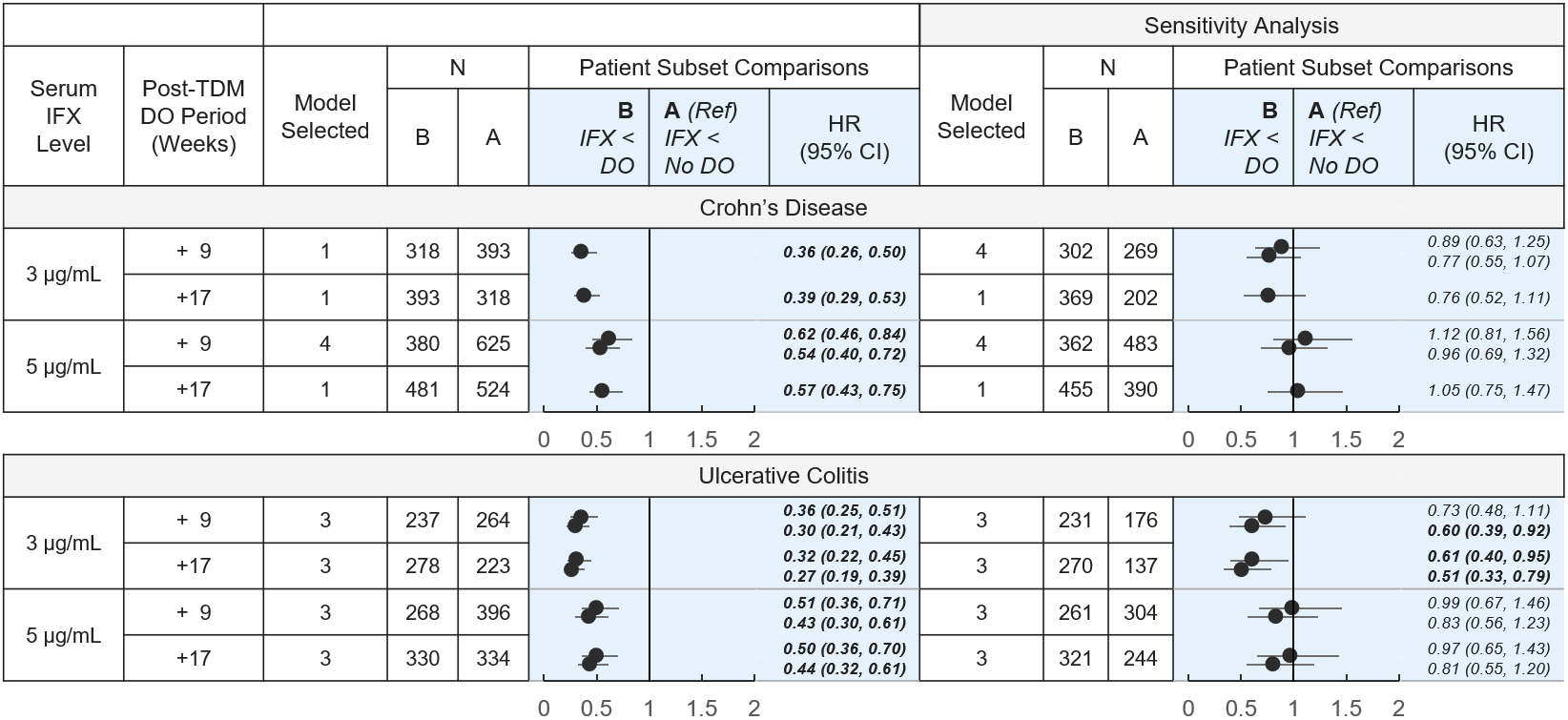


CI=confidence interval; DO=dose optimization; HR=hazard ratio; IFX=infliximab; Ref=reference; TDM=therapeutic drug monitoring.

Dose optimization thresholds: High: A treatment interval decrease of ≥11 days (1.57 weeks) with a posterior interval of ≤35 days (5 weeks), and/or a dose level increase of ≥1.5 mg/kg with a posterior dose level of ≥9 mg/kg. Low: A treatment interval decrease of ≥11 days (1.57 weeks) with a posterior interval of ≤46 days (6.57 weeks), and/or dose level increase of ≥1.5 mg/kg with a posterior dose level of ≥7 mg/kg. Dose optimization within post-TDM periods were identified using low threshold criteria.

Model details are described in Suppl Table 10b. Model selection decision tree is shown in Suppl Figure 1b. Model results available in Suppl Tables 23 and 24.

HR (95% CI) displayed to a maximum of 2.0 and shown in bold when p<0.05. Doublet results based on DO at 4 weeks and 6 weeks, respectively.

A hazard ratio of <1 indicates a persistence advantage over the reference. If the CI includes 1, there is no statistically significant difference in persistence. If the CI does not include 1, the difference in persistence is statistically significant.

A=First TDM serum IFX concentration below level and no dose optimization within post-TDM period;

B=First TDM serum IFX concentration below level and dose optimization within post-TDM period.

**Supplementary Tables**

Suppl Table 1a: Comparison of Persistence After TDM and Before Any TDM (Province Interaction) in Patients with Crohn’s Disease

| **Model Variable** | **Class Level** | **Reference Level** | **Hazard Ratio**  **(95% CI)** | **p-value** |
| --- | --- | --- | --- | --- |
| Time-dependent covariate x Province |  |  |  |  |
| Alberta | TDM | No TDM | 1.19 (0.96, 1.47) | 0.1153 |
| Atlantic^†^ | TDM | No TDM | **1.29 (1.04, 1.61)** | **0.0200** |
| British Columbia | TDM | No TDM | 1.15 (0.92, 1.44) | 0.2146 |
| Ontario | TDM | No TDM | 1.18 (0.98, 1.42) | 0.0763 |
| Quebec | TDM | No TDM | **1.25 (1.04, 1.50)** | **0.0193** |
| Saskatchewan/Manitoba | TDM | No TDM | 1.19 (0.81, 1.76) | 0.3682 |
| Interaction^‡^ |  |  |  | **0.0421** |
| Covariates |  |  |  |  |
| Age group | <65 years | ≥65 years | **0.77 (0.70, 0.86)** | **<0.0001** |
| Gender | Female | Male | **1.19 (1.11, 1.28)** | **<0.0001** |
| Weight | <62 kg | ≥86 kg | 1.10 (0.99, 1.21) | 0.0733 |
|  | 62 to <73 kg | Q4 (≥86 kg) | 1.02 (0.92, 1.12) | 0.7519 |
|  | 73 to <86 kg | Q4 (≥86 kg) | 1.05 (0.95, 1.16) | 0.3049 |

CI=confidence interval; Q=quartile; TDM=therapeutic drug monitoring

^†^Atlantic includes New Brunswick, Nova Scotia, Prince Edward Island, Newfoundland and Labrador.

^‡^Statistical significance was tested using a threshold of p<0.1.

Note: Results are based on 140-day discontinuation rule (see Methods). Hazard ratio and 95% CI are from time-dependent Cox proportional hazards model 1 (Suppl Table 10a) stratified by year of initiation of infliximab treatment, with covariates of age group, gender, quartiles of first recorded weight, and province/region, with a time-dependent covariate modelling TDM (TDM vs. No TDM) and its interaction with province/region. A hazard ratio of <1 indicates an advantage for the class level over the reference level. HR (95% CI) is shown in bold when p<0.05.

Suppl Table 1b: Comparison of Persistence After TDM and Before Any TDM (Province Interaction) in Patients with Ulcerative Colitis

| **Model Variable** | **Class Level** | **Reference Level** | **Hazard Ratio**  **(95% CI)** | **p-value** |
| --- | --- | --- | --- | --- |
| Time-dependent covariate x Province |  |  |  |  |
| Alberta | TDM | No TDM | 1.06 (0.82, 1.35) | 0.6723 |
| Atlantic^†^ | TDM | No TDM | **1.36 (1.03, 1.79)** | **0.0292** |
| British Columbia | TDM | No TDM | **1.40 (1.09, 1.81)** | **0.0095** |
| Ontario | TDM | No TDM | **1.39 (1.12, 1.73)** | **0.0025** |
| Quebec | TDM | No TDM | 1.09 (0.86, 1.37) | 0.4815 |
| Saskatchewan/Manitoba | TDM | No TDM | 1.09 (0.69, 1.73) | 0.7080 |
| Interaction^‡^ |  |  |  | 0.0918 |
| Covariates |  |  |  |  |
| Age group | <65 years | ≥65 years | **0.73 (0.65, 0.82)** | **<0.0001** |
| Gender | Female | Male | 1.01 (0.92, 1.10) | 0.8580 |
| Weight | <62 kg | ≥86 kg | 1.12 (1.00, 1.27) | 0.0587 |
|  | 62 to <73 kg | Q4 (≥86 kg) | 1.06 (0.94, 1.19) | 0.3528 |
|  | 73 to <86 kg | Q4 (≥86 kg) | 0.95 (0.85, 1.06) | 0.3706 |

CI=confidence interval; Q=quartile; TDM=therapeutic drug monitoring.

^†^Atlantic includes New Brunswick, Nova Scotia, Prince Edward Island, Newfoundland and Labrador.

^‡^Statistical significance was tested using a threshold of p<0.1.

Note: Results are based on 140-day discontinuation rule (see Methods). Hazard ratio and 95% CI are from time-dependent Cox proportional hazards model 1 (Suppl Table 10a) stratified by year of initiation of infliximab treatment, with covariates of age group, gender, quartiles of first recorded weight, and province/region, with a time-dependent covariate modelling TDM (TDM vs. No TDM) and its interaction with province/region. A hazard ratio of <1 indicates an advantage for the class level over the reference level. HR (95% CI) is shown in bold when p<0.05.

Suppl Table 2a: Subgroup Demographics and Baseline Characteristics of Patients with CD with no DO (Based on Low Threshold Criteria) Prior to their First Instance of TDM (Serum IFX Threshold: 3 μg/mL; Post-Index Period for Dose Optimization: +9 weeks; Post-Index Dose Optimization Threshold: Low)

|  | **A**  **(N=393)** | **B**  **(N=318)** | **C**  **(N=864)** | **D**  **(N=109)** | **p-value** |
| --- | --- | --- | --- | --- | --- |
| **Age (years)** | | | | | <0.0001 |
| Mean (SD) | 46.2 (15.4) | 43.7 (15.7) | 41.4 (15.1) | 41.9 (14.4) |  |
| **Age group (years)** | | | | | 0.0026 |
| 18 to 64 | 337 (85.8%) | 289 (90.9%) | 794 (91.9%) | 103 (94.5%) |  |
| 65 to 90 | 56 (14.2%) | 29 (9.1%) | 70 (8.1%) | 6 (5.5%) |  |
| **Gender** | | | | | 0.8218 |
| Female | 201 (51.1%) | 169 (53.1%) | 460 (53.2%) | 54 (49.5%) |  |
| Male | 192 (48.9%) | 149 (46.9%) | 404 (46.8%) | 55 (50.5%) |  |
| **First recorded weight** | | | | | 0.2623 |
| Mean (SD) | 74.5 (19.2) | 72.6 (18.2) | 74.8 (16.9) | 75.2 (17.8) |  |
| Missing | 1 | 0 | 2 | 0 |  |
| **Province/region of treating physician** | | | | | 0.0001 |
| Alberta | 11 (2.8%) | 9 (2.8%) | 29 (3.4%) | 4 (3.7%) |  |
| Atlantic^†^ | 75 (19.1%) | 44 (13.8%) | 178 (20.6%) | 12 (11.0%) |  |
| British Columbia | 38 (9.7%) | 28 (8.8%) | 107 (12.4%) | 10 (9.2%) |  |
| Ontario | 184 (46.8%) | 174 (54.7%) | 439 (50.8%) | 53 (48.6%) |  |
| Quebec | 84 (21.4%) | 63 (19.8%) | 106 (12.3%) | 30 (27.5%) |  |
| Saskatchewan/  Manitoba | 1 (0.3%) | 0 (0.0%) | 5 (0.6%) | 0 (0.0%) |  |
| **Year of initiation of IFX treatment** | | | | | 0.0005 |
| 2015 | 50 (12.7%) | 20 (6.3%) | 126 (14.6%) | 12 (11.0%) |  |
| 2016 | 91 (23.2%) | 54 (17.0%) | 145 (16.8%) | 11 (10.1%) |  |
| 2017 | 136 (34.6%) | 125 (39.3%) | 316 (36.6%) | 47 (43.1%) |  |
| 2018 | 116 (29.5%) | 119 (37.4%) | 277 (32.1%) | 39 (35.8%) |  |
| **Time to first TDM (days)** | | | | | <0.0001 |
| Mean (SD) | 395.9 (337.6) | 285.8 (263.6) | 352.4 (329.2) | 302.9 (281.0) |  |
| **Year of first instance of TDM** | | | | | 0.0840 |
| 2015 | 0 (0.0%) | 0 (0.0%) | 1 (0.1%) | 0 (0.0%) |  |
| 2016 | 1 (0.3%) | 0 (0.0%) | 2 (0.2%) | 0 (0.0%) |  |
| 2017 | 131 (33.3%) | 112 (35.2%) | 299 (34.6%) | 30 (27.5%) |  |
| 2018 | 168 (42.7%) | 133 (41.8%) | 415 (48.0%) | 63 (57.8%) |  |
| 2019 | 93 (23.7%) | 73 (23.0%) | 147 (17.0%) | 16 (14.7%) |  |

TDM=therapeutic drug monitoring. P-value: categorical variables, chi-square test; numeric variables, ANOVA.

^†^Atlantic includes New Brunswick, Nova Scotia, Prince Edward Island, Newfoundland and Labrador.

Dose optimization thresholds: High: A treatment interval decrease of ≥11 days (1.57 weeks) with a posterior interval of ≤35 days (5 weeks), and/or a dose level increase of ≥1.5 mg/kg with a posterior dose level of ≥9 mg/kg. Low: A treatment interval decrease of ≥11 days (1.57 weeks) with a posterior interval of ≤46 days (6.57 weeks), and/or dose level increase of ≥1.5 mg/kg with a posterior dose level of ≥7 mg/kg.

A=First TDM serum IFX concentration below threshold and no dose optimization within post-index period;

B=First TDM serum IFX concentration below threshold and dose optimization within post-index period;

C=First TDM serum IFX concentration at or above threshold and no dose optimization within post-index period;

D=First TDM serum IFX concentration at or above threshold and dose optimization within post-index period.

Suppl Table 2b: Subgroup Demographics and Baseline Characteristics of Patients with CD with no DO (Based on Low Threshold Criteria) Prior to their First Instance of TDM (Serum IFX Threshold: 3 μg/mL; Post-Index Period for Dose Optimization: +17 weeks; Post-Index Dose Optimization Threshold: Low)

|  | **A (N=318)** | **B (N=393)** | **C (N=806)** | **D (N=167)** | **p-value** |
| --- | --- | --- | --- | --- | --- |
| **Age (years)** | | | | | <0.0001 |
| Mean (SD) | 46.7 (15.22) | 43.8 (15.67) | 41.2 (15.01) | 42.7 (15.18) |  |
| **Age group (years)** | | | | | 0.0037 |
| 18 to 64 | 271 (85.2%) | 355 (90.3%) | 743 (92.2%) | 154 (92.2%) |  |
| 65 to 90 | 47 (14.8%) | 38 (9.7%) | 63 (7.8%) | 13 (7.8%) |  |
| **Gender** | | | | | 0.9432 |
| Female | 163 (51.3%) | 207 (52.7%) | 428 (53.1%) | 86 (51.5%) |  |
| Male | 155 (48.7%) | 186 (47.3%) | 378 (46.9%) | 81 (48.5%) |  |
| **First recorded weight** | | | | | 0.2233 |
| Mean (SD) | 74.79 (19.566) | 72.75 (18.070) | 74.74 (16.974) | 75.45 (17.135) |  |
| Missing | 1 | 0 | 0 | 2 |  |
| **Province/region of treating physician** | | | | | 0.0002 |
| Alberta | 10 (3.1%) | 10 (2.5%) | 29 (3.6%) | 4 (2.4%) |  |
| Atlantic^†^ | 68 (21.4%) | 51 (13.0%) | 166 (20.6%) | 24 (14.4%) |  |
| British Columbia | 30 (9.4%) | 36 (9.2%) | 96 (11.9%) | 21 (12.6%) |  |
| Ontario | 143 (45.0%) | 215 (54.7%) | 412 (51.1%) | 80 (47.9%) |  |
| Quebec | 66 (20.8%) | 81 (20.6%) | 98 (12.2%) | 38 (22.8%) |  |
| Saskatchewan/  Manitoba | 1 (0.3%) | 0 (0.0%) | 5 (0.6%) | 0 (0.0%) |  |
| **Year of initiation of IFX treatment** | | | | | <0.0001 |
| 2015 | 40 (12.6%) | 30 (7.6%) | 120 (14.9%) | 18 (10.8%) |  |
| 2016 | 82 (25.8%) | 63 (16.0%) | 141 (17.5%) | 15 (9.0%) |  |
| 2017 | 108 (34.0%) | 153 (38.9%) | 289 (35.9%) | 74 (44.3%) |  |
| 2018 | 88 (27.7%) | 147 (37.4%) | 256 (31.8%) | 60 (35.9%) |  |
| **Time to first TDM (days)** | | | | | <0.0001 |
| Mean (SD) | 402.2 (326.86) | 301.7 (290.99) | 358.5 (330.14) | 290.6 (289.25) |  |
| **Year of first instance of TDM** | | | | | 0.0770 |
| 2015 | 0 (0.0%) | 0 (0.0%) | 1 (0.1%) | 0 (0.0%) |  |
| 2016 | 1 (0.3%) | 0 (0.0%) | 2 (0.2%) | 0 (0.0%) |  |
| 2017 | 110 (34.6%) | 133 (33.8%) | 278 (34.5%) | 51 (30.5%) |  |
| 2018 | 135 (42.5%) | 166 (42.2%) | 385 (47.8%) | 93 (55.7%) |  |
| 2019 | 72 (22.6%) | 94 (23.9%) | 140 (17.4%) | 23 (13.8%) |  |

TDM=therapeutic drug monitoring. P-value: categorical variables, chi-square test; numeric variables, ANOVA.

^†^Atlantic includes New Brunswick, Nova Scotia, Prince Edward Island, Newfoundland and Labrador.

Dose optimization thresholds: High: A treatment interval decrease of ≥11 days (1.57 weeks) with a posterior interval of ≤35 days (5 weeks), and/or a dose level increase of ≥1.5 mg/kg with a posterior dose level of ≥9 mg/kg. Low: A treatment interval decrease of ≥11 days (1.57 weeks) with a posterior interval of ≤46 days (6.57 weeks), and/or dose level increase of ≥1.5 mg/kg with a posterior dose level of ≥7 mg/kg.

A=First TDM serum IFX concentration below threshold and no dose optimization within post-index period;

B=First TDM serum IFX concentration below threshold and dose optimization within post-index period;

C=First TDM serum IFX concentration at or above threshold and no dose optimization within post-index period;

D=First TDM serum IFX concentration at or above threshold and dose optimization within post-index period.

Suppl Table 2c: Subgroup Demographics and Baseline Characteristics of Patients with CD with no DO (Based on Low Threshold Criteria) Prior to their First Instance of TDM (Serum IFX Threshold: 5 μg/mL; Post-Index Period for Dose Optimization: +9 weeks; Post-Index Dose Optimization Threshold: Low)

|  | **A (N=625)** | **B (N=380)** | **C (N=632)** | **D (N=47)** | **p-value** |
| --- | --- | --- | --- | --- | --- |
| **Age (years)** | | | | | 0.0001 |
| Mean (SD) | 44.8 (15.51) | 43.5 (15.75) | 41.0 (14.97) | 41.0 (11.60) |  |
| **Age group (years)** | | | | | 0.0012 |
| 18 to 64 | 544 (87.0%) | 346 (91.1%) | 587 (92.9%) | 46 (97.9%) |  |
| 65 to 90 | 81 (13.0%) | 34 (8.9%) | 45 (7.1%) | 1 (2.1%) |  |
| **Gender** | | | | | 0.1167 |
| Female | 311 (49.8%) | 194 (51.1%) | 350 (55.4%) | 29 (61.7%) |  |
| Male | 314 (50.2%) | 186 (48.9%) | 282 (44.6%) | 18 (38.3%) |  |
| **First recorded weight** | | | | | 0.4752 |
| Mean (SD) | 75.00 (18.112) | 73.21 (18.235) | 74.45 (17.179) | 73.61 (17.207) |  |
| Missing | 1 | 0 | 2 | 0 |  |
| **Province/region of treating physician** | | | | | 0.0001 |
| Alberta | 19 (3.0%) | 10 (2.6%) | 21 (3.3%) | 3 (6.4%) |  |
| Atlantic^†^ | 121 (19.4%) | 52 (13.7%) | 132 (20.9%) | 4 (8.5%) |  |
| British Columbia | 64 (10.2%) | 34 (8.9%) | 81 (12.8%) | 4 (8.5%) |  |
| Ontario | 295 (47.2%) | 202 (53.2%) | 328 (51.9%) | 25 (53.2%) |  |
| Quebec | 123 (19.7%) | 82 (21.6%) | 67 (10.6%) | 11 (23.4%) |  |
| Saskatchewan/  Manitoba | 3 (0.5%) | 0 (0.0%) | 3 (0.5%) | 0 (0.0%) |  |
| **Year of initiation of IFX treatment** | | | | | <0.0001 |
| 2015 | 81 (13.0%) | 28 (7.4%) | 95 (15.0%) | 4 (8.5%) |  |
| 2016 | 145 (23.2%) | 63 (16.6%) | 91 (14.4%) | 2 (4.3%) |  |
| 2017 | 209 (33.4%) | 147 (38.7%) | 243 (38.4%) | 25 (53.2%) |  |
| 2018 | 190 (30.4%) | 142 (37.4%) | 203 (32.1%) | 16 (34.0%) |  |
| **Time to first TDM (days)** | | | | | <0.0001 |
| Mean (SD) | 387.8 (328.80) | 294.3 (271.96) | 344.4 (334.60) | 257.2 (232.22) |  |
| **Year of first instance of TDM** | | | | | 0.4313 |
| 2015 | 0 (0.0%) | 0 (0.0%) | 1 (0.2%) | 0 (0.0%) |  |
| 2016 | 2 (0.3%) | 0 (0.0%) | 1 (0.2%) | 0 (0.0%) |  |
| 2017 | 207 (33.1%) | 127 (33.4%) | 223 (35.3%) | 15 (31.9%) |  |
| 2018 | 280 (44.8%) | 170 (44.7%) | 303 (47.9%) | 26 (55.3%) |  |
| 2019 | 136 (21.8%) | 83 (21.8%) | 104 (16.5%) | 6 (12.8%) |  |

TDM=therapeutic drug monitoring. P-value: categorical variables, chi-square test; numeric variables, ANOVA.

^†^Atlantic includes New Brunswick, Nova Scotia, Prince Edward Island, Newfoundland and Labrador.

Dose optimization thresholds: High: A treatment interval decrease of ≥11 days (1.57 weeks) with a posterior interval of ≤35 days (5 weeks), and/or a dose level increase of ≥1.5 mg/kg with a posterior dose level of ≥9 mg/kg. Low: A treatment interval decrease of ≥11 days (1.57 weeks) with a posterior interval of ≤46 days (6.57 weeks), and/or dose level increase of ≥1.5 mg/kg with a posterior dose level of ≥7 mg/kg.

A=First TDM serum IFX concentration below threshold and no dose optimization within post-index period;

B=First TDM serum IFX concentration below threshold and dose optimization within post-index period;

C=First TDM serum IFX concentration at or above threshold and no dose optimization within post-index period;

D=First TDM serum IFX concentration at or above threshold and dose optimization within post-index period.

Suppl Table 2d: Subgroup Demographics and Baseline Characteristics of Patients with CD with no DO (Based on Low Threshold Criteria) Prior to their First Instance of TDM (Serum IFX Threshold: 5 μg/mL; Post-Index Period for Dose Optimization: +17 weeks; Post-Index Dose Optimization Threshold: Low)

|  | **A (N=524)** | **B (N=481)** | **C (N=600)** | **D (N=79)** | **p-value** |
| --- | --- | --- | --- | --- | --- |
| **Age (years)** | | | | | 0.0001 |
| Mean (SD) | 44.8 (15.32) | 43.8 (15.91) | 40.9 (14.99) | 41.7 (12.87) |  |
| **Age group (years)** | | | | | 0.0037 |
| 18 to 64 | 456 (87.0%) | 434 (90.2%) | 558 (93.0%) | 75 (94.9%) |  |
| 65 to 90 | 68 (13.0%) | 47 (9.8%) | 42 (7.0%) | 4 (5.1%) |  |
| **Gender** | | | | | 0.1273 |
| Female | 258 (49.2%) | 247 (51.4%) | 333 (55.5%) | 46 (58.2%) |  |
| Male | 266 (50.8%) | 234 (48.6%) | 267 (44.5%) | 33 (41.8%) |  |
| **First recorded weight** | | | | | 0.2859 |
| Mean (SD) | 75.32 (18.322) | 73.24 (17.960) | 74.25 (17.206) | 75.49 (16.950) |  |
| Missing | 1 | 0 | 0 | 2 |  |
| **Province/region of treating physician** | | | | | <0.0001 |
| Alberta | 18 (3.4%) | 11 (2.3%) | 21 (3.5%) | 3 (3.8%) |  |
| Atlantic^†^ | 108 (20.6%) | 65 (13.5%) | 126 (21.0%) | 10 (12.7%) |  |
| British Columbia | 51 (9.7%) | 47 (9.8%) | 75 (12.5%) | 10 (12.7%) |  |
| Ontario | 243 (46.4%) | 254 (52.8%) | 312 (52.0%) | 41 (51.9%) |  |
| Quebec | 101 (19.3%) | 104 (21.6%) | 63 (10.5%) | 15 (19.0%) |  |
| Saskatchewan/  Manitoba | 3 (0.6%) | 0 (0.0%) | 3 (0.5%) | 0 (0.0%) |  |
| **Year of initiation of IFX treatment** | | | | | <0.0001 |
| 2015 | 68 (13.0%) | 41 (8.5%) | 92 (15.3%) | 7 (8.9%) |  |
| 2016 | 134 (25.6%) | 74 (15.4%) | 89 (14.8%) | 4 (5.1%) |  |
| 2017 | 170 (32.4%) | 186 (38.7%) | 227 (37.8%) | 41 (51.9%) |  |
| 2018 | 152 (29.0%) | 180 (37.4%) | 192 (32.0%) | 27 (34.2%) |  |
| **Time to first TDM (days)** | | | | | <0.0001 |
| Mean (SD) | 396.9 (323.30) | 304.0 (291.28) | 348.1 (333.71) | 264.6 (283.39) |  |
| **Year of first instance of TDM** | | | | | 0.3762 |
| 2015 | 0 (0.0%) | 0 (0.0%) | 1 (0.2%) | 0 (0.0%) |  |
| 2016 | 2 (0.4%) | 0 (0.0%) | 1 (0.2%) | 0 (0.0%) |  |
| 2017 | 175 (33.4%) | 159 (33.1%) | 213 (35.5%) | 25 (31.6%) |  |
| 2018 | 233 (44.5%) | 217 (45.1%) | 287 (47.8%) | 42 (53.2%) |  |
| 2019 | 114 (21.8%) | 105 (21.8%) | 98 (16.3%) | 12 (15.2%) |  |

TDM=therapeutic drug monitoring. P-value: categorical variables, chi-square test; numeric variables, ANOVA.

^†^Atlantic includes New Brunswick, Nova Scotia, Prince Edward Island, Newfoundland and Labrador.

Dose optimization thresholds: High: A treatment interval decrease of ≥11 days (1.57 weeks) with a posterior interval of ≤35 days (5 weeks), and/or a dose level increase of ≥1.5 mg/kg with a posterior dose level of ≥9 mg/kg. Low: A treatment interval decrease of ≥11 days (1.57 weeks) with a posterior interval of ≤46 days (6.57 weeks), and/or dose level increase of ≥1.5 mg/kg with a posterior dose level of ≥7 mg/kg.

A=First TDM serum IFX concentration below threshold and no dose optimization within post-index period;

B=First TDM serum IFX concentration below threshold and dose optimization within post-index period;

C=First TDM serum IFX concentration at or above threshold and no dose optimization within post-index period;

D=First TDM serum IFX concentration at or above threshold and dose optimization within post-index period.

Suppl Table 2e: Subgroup Demographics and Baseline Characteristics of Patients with CD with no DO (Based on Low Threshold Criteria) Prior to their First Instance of TDM (Serum IFX Threshold: 10 μg/mL; Post-Index Period for Dose Optimization: +9 weeks; Post-Index Dose Optimization Threshold: Low)

|  | **A (N=979)** | **B (N=416)** | **C (N=278)** | **D (N=11)** | **p-value** |
| --- | --- | --- | --- | --- | --- |
| **Age (years)** | | | | | 0.1749 |
| Mean (SD) | 43.4 (15.40) | 43.4 (15.46) | 41.2 (15.09) | 39.5 (10.65) |  |
| **Age group (years)** | | | | | 0.0382 |
| 18 to 64 | 870 (88.9%) | 381 (91.6%) | 261 (93.9%) | 11 (100.0%) |  |
| 65 to 90 | 109 (11.1%) | 35 (8.4%) | 17 (6.1%) | 0 |  |
| **Gender** | | | | | 0.0349 |
| Female | 499 (51.0%) | 214 (51.4%) | 162 (58.3%) | 9 (81.8%) |  |
| Male | 480 (49.0%) | 202 (48.6%) | 116 (41.7%) | 2 (18.2%) |  |
| **First recorded weight** | | | | | 0.2807 |
| Mean (SD) | 75.03 (17.799) | 73.18 (18.011) | 73.64 (17.077) | 76.28 (22.189) |  |
| Missing | 3 | 0 | 0 | 0 |  |
| **Province/region of treating physician** | | | | | <0.0001 |
| Alberta | 29 (3.0%) | 10 (2.4%) | 11 (4.0%) | 3 (27.3%) |  |
| Atlantic^†^ | 184 (18.8%) | 54 (13.0%) | 69 (24.8%) | 2 (18.2%) |  |
| British Columbia | 113 (11.5%) | 38 (9.1%) | 32 (11.5%) | 0 (0.0%) |  |
| Ontario | 485 (49.5%) | 225 (54.1%) | 138 (49.6%) | 2 (18.2%) |  |
| Quebec | 164 (16.8%) | 89 (21.4%) | 26 (9.4%) | 4 (36.4%) |  |
| Saskatchewan/  Manitoba | 4 (0.4%) | 0 (0.0%) | 2 (0.7%) | 0 (0.0%) |  |
| **Year of initiation of IFX treatment** | | | | | <0.0001 |
| 2015 | 142 (14.5%) | 28 (6.7%) | 34 (12.2%) | 4 (36.4%) |  |
| 2016 | 200 (20.4%) | 65 (15.6%) | 36 (12.9%) | 0 |  |
| 2017 | 335 (34.2%) | 168 (40.4%) | 117 (42.1%) | 4 (36.4%) |  |
| 2018 | 302 (30.8%) | 155 (37.3%) | 91 (32.7%) | 3 (27.3%) |  |
| **Time to first TDM (days)** | | | | | <0.0001 |
| Mean (SD) | 382.4 (334.81) | 287.2 (264.59) | 308.1 (317.17) | 402.7 (370.64) |  |
| **Year of first instance of TDM** | | | | | 0.4875 |
| 2015 | 0 (0.0%) | 0 (0.0%) | 1 (0.4%) | 0 (0.0%) |  |
| 2016 | 3 (0.3%) | 0 (0.0%) | 0 (0.0%) | 0 (0.0%) |  |
| 2017 | 325 (33.2%) | 137 (32.9%) | 105 (37.8%) | 5 (45.5%) |  |
| 2018 | 456 (46.6%) | 192 (46.2%) | 127 (45.7%) | 4 (36.4%) |  |
| 2019 | 195 (19.9%) | 87 (20.9%) | 45 (16.2%) | 2 (18.2%) |  |

TDM=therapeutic drug monitoring. P-value: categorical variables, chi-square test; numeric variables, ANOVA.

^†^Atlantic includes New Brunswick, Nova Scotia, Prince Edward Island, Newfoundland and Labrador.

Dose optimization thresholds: High: A treatment interval decrease of ≥11 days (1.57 weeks) with a posterior interval of ≤35 days (5 weeks), and/or a dose level increase of ≥1.5 mg/kg with a posterior dose level of ≥9 mg/kg. Low: A treatment interval decrease of ≥11 days (1.57 weeks) with a posterior interval of ≤46 days (6.57 weeks), and/or dose level increase of ≥1.5 mg/kg with a posterior dose level of ≥7 mg/kg.

A=First TDM serum IFX concentration below threshold and no dose optimization within post-index period;

B=First TDM serum IFX concentration below threshold and dose optimization within post-index period;

C=First TDM serum IFX concentration at or above threshold and no dose optimization within post-index period;

D=First TDM serum IFX concentration at or above threshold and dose optimization within post-index period.

Suppl Table 2f: Subgroup Demographics and Baseline Characteristics of Patients with CD with no DO (Based on Low Threshold Criteria) Prior to their First Instance of TDM (Serum IFX Threshold: 10 μg/mL; Post-Index Period for Dose Optimization: +17 weeks; Post-Index Dose Optimization Threshold: Low)

|  | **A (N=850)** | **B (N=545)** | **C (N=274)** | **D (N=15)** | **p-value** |
| --- | --- | --- | --- | --- | --- |
| **Age (years)** | | | | | 0.1697 |
| Mean (SD) | 43.3 (15.29) | 43.5 (15.62) | 41.1 (15.10) | 42.5 (11.86) |  |
| **Age group (years)** | | | | | 0.0702 |
| 18 to 64 | 757 (89.1%) | 494 (90.6%) | 257 (93.8%) | 15 (100.0%) |  |
| 65 to 90 | 93 (10.9%) | 51 (9.4%) | 17 (6.2%) | 0 (0.0%) |  |
| **Gender** | | | | | 0.0775 |
| Female | 430 (50.6%) | 283 (51.9%) | 161 (58.8%) | 10 (66.7%) |  |
| Male | 420 (49.4%) | 262 (48.1%) | 113 (41.2%) | 5 (33.3%) |  |
| **First recorded weight** | | | | | 0.2393 |
| Mean (SD) | 75.14 (17.906) | 73.44 (17.798) | 73.53 (17.169) | 77.62 (19.017) |  |
| Missing | 1 | 2 | 0 | 0 |  |
| **Province/region of treating physician** | | | | | <0.0001 |
| Alberta | 28 (3.3%) | 11 (2.0%) | 11 (4.0%) | 3 (20.0%) |  |
| Atlantic^†^ | 166 (19.5%) | 72 (13.2%) | 68 (24.8%) | 3 (20.0%) |  |
| British Columbia | 94 (11.1%) | 57 (10.5%) | 32 (11.7%) | 0 (0.0%) |  |
| Ontario | 417 (49.1%) | 293 (53.8%) | 138 (50.4%) | 2 (13.3%) |  |
| Quebec | 141 (16.6%) | 112 (20.6%) | 23 (8.4%) | 7 (46.7%) |  |
| Saskatchewan/  Manitoba | 4 (0.5%) | 0 (0.0%) | 2 (0.7%) | 0 (0.0%) |  |
| **Year of initiation of IFX treatment** | | | | | <0.0001 |
| 2015 | 126 (14.8%) | 44 (8.1%) | 34 (12.4%) | 4 (26.7%) |  |
| 2016 | 187 (22.0%) | 78 (14.3%) | 36 (13.1%) | 0 (0.0%) |  |
| 2017 | 284 (33.4%) | 219 (40.2%) | 113 (41.2%) | 8 (53.3%) |  |
| 2018 | 253 (29.8%) | 204 (37.4%) | 91 (33.2%) | 3 (20.0%) |  |
| **Time to first TDM (days)** | | | | | <0.0001 |
| Mean (SD) | 390.2 (330.99) | 297.6 (289.11) | 310.8 (318.64) | 329.1 (339.22) |  |
| **Year of first instance of TDM** | | | | | 0.4213 |
| 2015 | 0 (0.0%) | 0 (0.0%) | 1 (0.4%) | 0 (0.0%) |  |
| 2016 | 3 (0.4%) | 0 (0.0%) | 0 (0.0%) | 0 (0.0%) |  |
| 2017 | 284 (33.4%) | 178 (32.7%) | 104 (38.0%) | 6 (40.0%) |  |
| 2018 | 396 (46.6%) | 252 (46.2%) | 124 (45.3%) | 7 (46.7%) |  |
| 2019 | 167 (19.6%) | 115 (21.1%) | 45 (16.4%) | 2 (13.3%) |  |

TDM=therapeutic drug monitoring. P-value: categorical variables, chi-square test; numeric variables, ANOVA.

^†^Atlantic includes New Brunswick, Nova Scotia, Prince Edward Island, Newfoundland and Labrador.

Dose optimization thresholds: High: A treatment interval decrease of ≥11 days (1.57 weeks) with a posterior interval of ≤35 days (5 weeks), and/or a dose level increase of ≥1.5 mg/kg with a posterior dose level of ≥9 mg/kg. Low: A treatment interval decrease of ≥11 days (1.57 weeks) with a posterior interval of ≤46 days (6.57 weeks), and/or dose level increase of ≥1.5 mg/kg with a posterior dose level of ≥7 mg/kg.

A=First TDM serum IFX concentration below threshold and no dose optimization within post-index period;

B=First TDM serum IFX concentration below threshold and dose optimization within post-index period;

C=First TDM serum IFX concentration at or above threshold and no dose optimization within post-index period;

D=First TDM serum IFX concentration at or above threshold and dose optimization within post-index period.

Suppl Table 2g: Subgroup Demographics and Baseline Characteristics of Patients with CD with no DO (Based on Low Threshold Criteria) Prior to their First Instance of TDM (Serum IFX Threshold: 3 μg/mL; Post-Index Period for Dose Optimization: +9 weeks; Post-Index Dose Optimization Threshold: High)

|  | **A (N=507)** | **B (N=204)** | **C (N=928)** | **D (N=45)** | **p-value** |
| --- | --- | --- | --- | --- | --- |
| **Age (years)** | | | | | <0.0001 |
| Mean (SD) | 46.0 (15.36) | 42.9 (15.76) | 41.3 (15.00) | 44.1 (15.84) |  |
| **Age group (years)** | | | | | 0.0006 |
| 18 to 64 | 436 (86.0%) | 190 (93.1%) | 857 (92.3%) | 40 (88.9%) |  |
| 65 to 90 | 71 (14.0%) | 14 (6.9%) | 71 (7.7%) | 5 (11.1%) |  |
| **Gender** | | | | | 0.8491 |
| Female | 260 (51.3%) | 110 (53.9%) | 492 (53.0%) | 22 (48.9%) |  |
| Male | 247 (48.7%) | 94 (46.1%) | 436 (47.0%) | 23 (51.1%) |  |
| **First recorded weight** | | | | | 0.5227 |
| Mean (SD) | 73.93 (18.626) | 73.00 (19.141) | 74.85 (16.980) | 75.02 (17.479) |  |
| Missing | 1 | 0 | 2 | 0 |  |
| **Province/region of treating physician** | | | | | 0.0030 |
| Alberta | 11 (2.2%) | 9 (4.4%) | 32 (3.4%) | 1 (2.2%) |  |
| Atlantic^†^ | 95 (18.7%) | 24 (11.8%) | 186 (20.0%) | 4 (8.9%) |  |
| British Columbia | 48 (9.5%) | 18 (8.8%) | 112 (12.1%) | 5 (11.1%) |  |
| Ontario | 249 (49.1%) | 109 (53.4%) | 470 (50.6%) | 22 (48.9%) |  |
| Quebec | 103 (20.3%) | 44 (21.6%) | 123 (13.3%) | 13 (28.9%) |  |
| Saskatchewan/  Manitoba | 1 (0.2%) | 0 (0.0%) | 5 (0.5%) | 0 (0.0%) |  |
| **Year of initiation of IFX treatment** | | | | | 0.0203 |
| 2015 | 57 (11.2%) | 13 (6.4%) | 134 (14.4%) | 4 (8.9%) |  |
| 2016 | 110 (21.7%) | 35 (17.2%) | 150 (16.2%) | 6 (13.3%) |  |
| 2017 | 183 (36.1%) | 78 (38.2%) | 343 (37.0%) | 20 (44.4%) |  |
| 2018 | 157 (31.0%) | 78 (38.2%) | 301 (32.4%) | 15 (33.3%) |  |
| **Time to first TDM (days)** | | | | | 0.0069 |
| Mean (SD) | 371.5 (322.41) | 284.9 (273.12) | 349.5 (327.05) | 292.2 (259.73) |  |
| **Year of first instance of TDM** | | | | | 0.0923 |
| 2015 | 0 (0.0%) | 0 (0.0%) | 1 (0.1%) | 0 (0.0%) |  |
| 2016 | 1 (0.2%) | 0 (0.0%) | 2 (0.2%) | 0 (0.0%) |  |
| 2017 | 168 (33.1%) | 75 (36.8%) | 318 (34.3%) | 11 (24.4%) |  |
| 2018 | 220 (43.4%) | 81 (39.7%) | 450 (48.5%) | 28 (62.2%) |  |
| 2019 | 118 (23.3%) | 48 (23.5%) | 157 (16.9%) | 6 (13.3%) |  |

TDM=therapeutic drug monitoring. P-value: categorical variables, chi-square test; numeric variables, ANOVA.

^†^Atlantic includes New Brunswick, Nova Scotia, Prince Edward Island, Newfoundland, and Labrador.

Dose optimization thresholds: High: A treatment interval decrease of ≥11 days (1.57 weeks) with a posterior interval of ≤35 days (5 weeks), and/or a dose level increase of ≥1.5 mg/kg with a posterior dose level of ≥9 mg/kg. Low: A treatment interval decrease of ≥11 days (1.57 weeks) with a posterior interval of ≤46 days (6.57 weeks), and/or dose level increase of ≥1.5 mg/kg with a posterior dose level of ≥7 mg/kg.

A=First TDM serum IFX concentration below threshold and no dose optimization within post-index period;

B=First TDM serum IFX concentration below threshold and dose optimization within post-index period;

C=First TDM serum IFX concentration at or above threshold and no dose optimization within post-index period;

D=First TDM serum IFX concentration at or above threshold and dose optimization within post-index period.

Suppl Table 2h: Subgroup Demographics and Baseline Characteristics of Patients with CD with no DO (Based on Low Threshold Criteria) Prior to their First Instance of TDM (Serum IFX Threshold: 3 μg/mL; Post-Index Period for Dose Optimization: +17 weeks; Post-Index Dose Optimization Threshold: High)

|  | **A (N=458)** | **B (N=253)** | **C (N=902)** | **D (N=71)** | **p-value** |
| --- | --- | --- | --- | --- | --- |
| **Age (years)** | | | | | <0.0001 |
| Mean (SD) | 46.1 (15.46) | 43.3 (15.52) | 41.2 (14.99) | 44.3 (15.54) |  |
| **Age group (years)** | | | | | 0.0016 |
| 18 to 64 | 394 (86.0%) | 232 (91.7%) | 834 (92.5%) | 63 (88.7%) |  |
| 65 to 90 | 64 (14.0%) | 21 (8.3%) | 68 (7.5%) | 8 (11.3%) |  |
| **Gender** | | | | | 0.8346 |
| Female | 239 (52.2%) | 131 (51.8%) | 480 (53.2%) | 34 (47.9%) |  |
| Male | 219 (47.8%) | 122 (48.2%) | 422 (46.8%) | 37 (52.1%) |  |
| **First recorded weight** | | | | | 0.5948 |
| Mean (SD) | 73.69 (18.700) | 73.62 (18.924) | 74.82 (17.029) | 75.27 (16.655) |  |
| Missing | 1 | 0 | 1 | 1 |  |
| **Province/region of treating physician** | | | | | 0.0020 |
| Alberta | 11 (2.4%) | 9 (3.6%) | 32 (3.5%) | 1 (1.4%) |  |
| Atlantic^†^ | 90 (19.7%) | 29 (11.5%) | 180 (20.0%) | 10 (14.1%) |  |
| British Columbia | 44 (9.6%) | 22 (8.7%) | 107 (11.9%) | 10 (14.1%) |  |
| Ontario | 220 (48.0%) | 138 (54.5%) | 460 (51.0%) | 32 (45.1%) |  |
| Quebec | 92 (20.1%) | 55 (21.7%) | 118 (13.1%) | 18 (25.4%) |  |
| Saskatchewan/  Manitoba | 1 (0.2%) | 0 (0.0%) | 5 (0.6%) | 0 (0.0%) |  |
| **Year of initiation of IFX treatment** | | | | | 0.0076 |
| 2015 | 54 (11.8%) | 16 (6.3%) | 131 (14.5%) | 7 (9.9%) |  |
| 2016 | 100 (21.8%) | 45 (17.8%) | 149 (16.5%) | 7 (9.9%) |  |
| 2017 | 162 (35.4%) | 99 (39.1%) | 332 (36.8%) | 31 (43.7%) |  |
| 2018 | 142 (31.0%) | 93 (36.8%) | 290 (32.2%) | 26 (36.6%) |  |
| **Time to first TDM (days)** | | | | | 0.0003 |
| Mean (SD) | 382.2 (325.59) | 282.4 (272.88) | 351.6 (326.53) | 286.0 (290.55) |  |
| **Year of first instance of TDM** | | | | | 0.0331 |
| 2015 | 0 (0.0%) | 0 (0.0%) | 1 (0.1%) | 0 (0.0%) |  |
| 2016 | 1 (0.2%) | 0 (0.0%) | 2 (0.2%) | 0 (0.0%) |  |
| 2017 | 147 (32.1%) | 96 (37.9%) | 310 (34.4%) | 19 (26.8%) |  |
| 2018 | 202 (44.1%) | 99 (39.1%) | 434 (48.1%) | 44 (62.0%) |  |
| 2019 | 108 (23.6%) | 58 (22.9%) | 155 (17.2%) | 8 (11.3%) |  |

TDM=therapeutic drug monitoring. P-value: categorical variables, chi-square test; numeric variables, ANOVA.

^†^Atlantic includes New Brunswick, Nova Scotia, Prince Edward Island, Newfoundland, and Labrador.

Dose optimization thresholds: High: A treatment interval decrease of ≥11 days (1.57 weeks) with a posterior interval of ≤35 days (5 weeks), and/or a dose level increase of ≥1.5 mg/kg with a posterior dose level of ≥9 mg/kg. Low: A treatment interval decrease of ≥11 days (1.57 weeks) with a posterior interval of ≤46 days (6.57 weeks), and/or dose level increase of ≥1.5 mg/kg with a posterior dose level of ≥7 mg/kg.

A=First TDM serum IFX concentration below threshold and no dose optimization within post-index period;

B=First TDM serum IFX concentration below threshold and dose optimization within post-index period;

C=First TDM serum IFX concentration at or above threshold and no dose optimization within post-index period;

D=First TDM serum IFX concentration at or above threshold and dose optimization within post-index period.

Suppl Table 2i: Subgroup Demographics and Baseline Characteristics of Patients with CD with no DO (Based on Low Threshold Criteria) Prior to their First Instance of TDM (Serum IFX Threshold: 5 μg/mL; Post-Index Period for Dose Optimization: +9 weeks; Post-Index Dose Optimization Threshold: High)

|  | **A (N=774)** | **B (N=231)** | **C (N=661)** | **D (N=18)** | **p-value** |
| --- | --- | --- | --- | --- | --- |
| **Age (years)** | | | | | 0.0001 |
| Mean (SD) | 44.7 (15.50) | 43.2 (15.94) | 41.0 (14.80) | 41.0 (13.18) |  |
| **Age group (years)** | | | | | 0.0019 |
| 18 to 64 | 677 (87.5%) | 213 (92.2%) | 616 (93.2%) | 17 (94.4%) |  |
| 65 to 90 | 97 (12.5%) | 18 (7.8%) | 45 (6.8%) | 1 (5.6%) |  |
| **Gender** | | | | | 0.1221 |
| Female | 384 (49.6%) | 121 (52.4%) | 368 (55.7%) | 11 (61.1%) |  |
| Male | 390 (50.4%) | 110 (47.6%) | 293 (44.3%) | 7 (38.9%) |  |
| **First recorded weight** | | | | | 0.7948 |
| Mean (SD) | 74.58 (17.863) | 73.48 (19.180) | 74.46 (17.252) | 72.00 (13.987) |  |
| Missing | 1 | 0 | 2 | 0 |  |
| **Province/region of treating physician** | | | | | 0.0001 |
| Alberta | 19 (2.5%) | 10 (4.3%) | 24 (3.6%) | 0 (0.0%) |  |
| Atlantic^†^ | 145 (18.7%) | 28 (12.1%) | 136 (20.6%) | 0 (0.0%) |  |
| British Columbia | 76 (9.8%) | 22 (9.5%) | 84 (12.7%) | 1 (5.6%) |  |
| Ontario | 377 (48.7%) | 120 (51.9%) | 342 (51.7%) | 11 (61.1%) |  |
| Quebec | 154 (19.9%) | 51 (22.1%) | 72 (10.9%) | 6 (33.3%) |  |
| Saskatchewan/  Manitoba | 3 (0.4%) | 0 (0.0%) | 3 (0.5%) | 0 (0.0%) |  |
| **Year of initiation of IFX treatment** | | | | | 0.0009 |
| 2015 | 94 (12.1%) | 15 (6.5%) | 97 (14.7%) | 2 (11.1%) |  |
| 2016 | 168 (21.7%) | 40 (17.3%) | 92 (13.9%) | 1 (5.6%) |  |
| 2017 | 267 (34.5%) | 89 (38.5%) | 259 (39.2%) | 9 (50.0%) |  |
| 2018 | 245 (31.7%) | 87 (37.7%) | 213 (32.2%) | 6 (33.3%) |  |
| **Time to first TDM (days)** | | | | | 0.0027 |
| Mean (SD) | 371.0 (320.29) | 290.2 (272.65) | 341.2 (330.97) | 235.5 (238.37) |  |
| **Year of first instance of TDM** | | | | | 0.3820 |
| 2015 | 0 (0.0%) | 0 (0.0%) | 1 (0.2%) | 0 (0.0%) |  |
| 2016 | 2 (0.3%) | 0 (0.0%) | 1 (0.2%) | 0 (0.0%) |  |
| 2017 | 254 (32.8%) | 80 (34.6%) | 232 (35.1%) | 6 (33.3%) |  |
| 2018 | 352 (45.5%) | 98 (42.4%) | 318 (48.1%) | 11 (61.1%) |  |
| 2019 | 166 (21.4%) | 53 (22.9%) | 109 (16.5%) | 1 (5.6%) |  |

TDM=therapeutic drug monitoring. P-value: categorical variables, chi-square test; numeric variables, ANOVA.

^†^Atlantic includes New Brunswick, Nova Scotia, Prince Edward Island, Newfoundland, and Labrador.

Dose optimization thresholds: High: A treatment interval decrease of ≥11 days (1.57 weeks) with a posterior interval of ≤35 days (5 weeks), and/or a dose level increase of ≥1.5 mg/kg with a posterior dose level of ≥9 mg/kg. Low: A treatment interval decrease of ≥11 days (1.57 weeks) with a posterior interval of ≤46 days (6.57 weeks), and/or dose level increase of ≥1.5 mg/kg with a posterior dose level of ≥7 mg/kg.

A=First TDM serum IFX concentration below threshold and no dose optimization within post-index period;

B=First TDM serum IFX concentration below threshold and dose optimization within post-index period;

C=First TDM serum IFX concentration at or above threshold and no dose optimization within post-index period;

D=First TDM serum IFX concentration at or above threshold and dose optimization within post-index period.

Suppl Table 2j: Subgroup Demographics and Baseline Characteristics of Patients with CD with no DO (Based on Low Threshold Criteria) Prior to their First Instance of TDM (Serum IFX Threshold: 5 μg/mL; Post-Index Period for Dose Optimization: +17 weeks; Post-Index Dose Optimization Threshold: High)

|  | **A (N=714)** | **B (N=291)** | **C (N=646)** | **D (N=33)** | **p-value** |
| --- | --- | --- | --- | --- | --- |
| **Age (years)** | | | | | 0.0001 |
| Mean (SD) | 44.6 (15.56) | 43.6 (15.73) | 40.9 (14.81) | 42.9 (13.57) |  |
| **Age group (years)** | | | | | 0.0038 |
| 18 to 64 | 625 (87.5%) | 265 (91.1%) | 603 (93.3%) | 30 (90.9%) |  |
| 65 to 90 | 89 (12.5%) | 26 (8.9%) | 43 (6.7%) | 3 (9.1%) |  |
| **Gender** | | | | | 0.1658 |
| Female | 359 (50.3%) | 146 (50.2%) | 360 (55.7%) | 19 (57.6%) |  |
| Male | 355 (49.7%) | 145 (49.8%) | 286 (44.3%) | 14 (42.4%) |  |
| **First recorded weight** | | | | | 0.9590 |
| Mean (SD) | 74.42 (17.854) | 74.09 (18.953) | 74.46 (17.351) | 72.93 (13.103) |  |
| Missing | 1 | 0 | 1 | 1 |  |
| **Province/region of treating physician** | | | | | 0.0002 |
| Alberta | 19 (2.7%) | 10 (3.4%) | 24 (3.7%) | 0 (0.0%) |  |
| Atlantic^†^ | 138 (19.3%) | 35 (12.0%) | 132 (20.4%) | 4 (12.1%) |  |
| British Columbia | 69 (9.7%) | 29 (10.0%) | 82 (12.7%) | 3 (9.1%) |  |
| Ontario | 344 (48.2%) | 153 (52.6%) | 336 (52.0%) | 17 (51.5%) |  |
| Quebec | 141 (19.7%) | 64 (22.0%) | 69 (10.7%) | 9 (27.3%) |  |
| Saskatchewan/  Manitoba | 3 (0.4%) | 0 (0.0%) | 3 (0.5%) | 0 (0.0%) |  |
| **Year of initiation of IFX treatment** | | | | | 0.0002 |
| 2015 | 90 (12.6%) | 19 (6.5%) | 95 (14.7%) | 4 (12.1%) |  |
| 2016 | 158 (22.1%) | 50 (17.2%) | 91 (14.1%) | 2 (6.1%) |  |
| 2017 | 241 (33.8%) | 115 (39.5%) | 253 (39.2%) | 15 (45.5%) |  |
| 2018 | 225 (31.5%) | 107 (36.8%) | 207 (32.0%) | 12 (36.4%) |  |
| **Time to first TDM (days)** | | | | | 0.0001 |
| Mean (SD) | 380.1 (323.44) | 284.5 (269.62) | 341.8 (328.75) | 271.0 (334.85) |  |
| **Year of first instance of TDM** | | | | | 0.2725 |
| 2015 | 0 (0.0%) | 0 (0.0%) | 1 (0.2%) | 0 (0.0%) |  |
| 2016 | 2 (0.3%) | 0 (0.0%) | 1 (0.2%) | 0 (0.0%) |  |
| 2017 | 229 (32.1%) | 105 (36.1%) | 228 (35.3%) | 10 (30.3%) |  |
| 2018 | 327 (45.8%) | 123 (42.3%) | 309 (47.8%) | 20 (60.6%) |  |
| 2019 | 156 (21.8%) | 63 (21.6%) | 107 (16.6%) | 3 (9.1%) |  |

TDM=therapeutic drug monitoring. P-value: categorical variables, chi-square test; numeric variables, ANOVA.

^†^Atlantic includes New Brunswick, Nova Scotia, Prince Edward Island, Newfoundland, and Labrador.

Dose optimization thresholds: High: A treatment interval decrease of ≥11 days (1.57 weeks) with a posterior interval of ≤35 days (5 weeks), and/or a dose level increase of ≥1.5 mg/kg with a posterior dose level of ≥9 mg/kg. Low: A treatment interval decrease of ≥11 days (1.57 weeks) with a posterior interval of ≤46 days (6.57 weeks), and/or dose level increase of ≥1.5 mg/kg with a posterior dose level of ≥7 mg/kg.

A=First TDM serum IFX concentration below threshold and no dose optimization within post-index period;

B=First TDM serum IFX concentration below threshold and dose optimization within post-index period;

C=First TDM serum IFX concentration at or above threshold and no dose optimization within post-index period;

D=First TDM serum IFX concentration at or above threshold and dose optimization within post-index period.

Suppl Table 2k: Subgroup Demographics and Baseline Characteristics of Patients with CD with no DO (Based on Low Threshold Criteria) Prior to their First Instance of TDM (Serum IFX Threshold: 10 μg/mL; Post-Index Period for Dose Optimization: +9 weeks; Post-Index Dose Optimization Threshold: High)

|  | **A (N=1149)** | **B (N=246)** | **C (N=286)** | **D (N=3)** | **p-value** |
| --- | --- | --- | --- | --- | --- |
| **Age (years)** | | | | | 0.1780 |
| Mean (SD) | 43.4 (15.33) | 43.1 (15.81) | 41.2 (14.99) | 40.3 (10.21) |  |
| **Age group (years)** | | | | | 0.0478 |
| 18 to 64 | 1024 (89.1%) | 227 (92.3%) | 269 (94.1%) | 3 (100.0%) |  |
| 65 to 90 | 125 (10.9%) | 19 (7.7%) | 17 (5.9%) | 0 (0.0%) |  |
| **Gender** | | | | | 0.0835 |
| Female | 583 (50.7%) | 130 (52.8%) | 169 (59.1%) | 2 (66.7%) |  |
| Male | 566 (49.3%) | 116 (47.2%) | 117 (40.9%) | 1 (33.3%) |  |
| **First recorded weight** | | | | | 0.6530 |
| Mean (SD) | 74.72 (17.669) | 73.35 (18.809) | 73.73 (17.218) | 74.97 (25.344) |  |
| Missing | 3 | 0 | 0 | 0 |  |
| **Province/region of treating physician** | | | | | <0.0001 |
| Alberta | 29 (2.5%) | 10 (4.1%) | 14 (4.9%) | 0 (0.0%) |  |
| Atlantic^†^ | 210 (18.3%) | 28 (11.4%) | 71 (24.8%) | 0 (0.0%) |  |
| British Columbia | 128 (11.1%) | 23 (9.3%) | 32 (11.2%) | 0 (0.0%) |  |
| Ontario | 579 (50.4%) | 131 (53.3%) | 140 (49.0%) | 0 (0.0%) |  |
| Quebec | 199 (17.3%) | 54 (22.0%) | 27 (9.4%) | 3 (100.0%) |  |
| Saskatchewan/  Manitoba | 4 (0.3%) | 0 (0.0%) | 2 (0.7%) | 0 (0.0%) |  |
| **Year of initiation of IFX treatment** | | | | | 0.0005 |
| 2015 | 155 (13.5%) | 15 (6.1%) | 36 (12.6%) | 2 (66.7%) |  |
| 2016 | 224 (19.5%) | 41 (16.7%) | 36 (12.6%) | 0 (0.0%) |  |
| 2017 | 406 (35.3%) | 97 (39.4%) | 120 (42.0%) | 1 (33.3%) |  |
| 2018 | 364 (31.7%) | 93 (37.8%) | 94 (32.9%) | 0 (0.0%) |  |
| **Time to first TDM (days)** | | | | | <0.0001 |
| Mean (SD) | 369.4 (326.46) | 282.2 (266.84) | 308.6 (317.42) | 614.0 (413.92) |  |
| **Year of first instance of TDM** | | | | | 0.4301 |
| 2015 | 0 (0.0%) | 0 (0.0%) | 1 (0.3%) | 0 (0.0%) |  |
| 2016 | 3 (0.3%) | 0 (0.0%) | 0 (0.0%) | 0 (0.0%) |  |
| 2017 | 378 (32.9%) | 84 (34.1%) | 108 (37.8%) | 2 (66.7%) |  |
| 2018 | 540 (47.0%) | 108 (43.9%) | 130 (45.5%) | 1 (33.3%) |  |
| 2019 | 228 (19.8%) | 54 (22.0%) | 47 (16.4%) | 0 (0.0%) |  |

TDM=therapeutic drug monitoring. P-value: categorical variables, chi-square test; numeric variables, ANOVA.

^†^Atlantic includes New Brunswick, Nova Scotia, Prince Edward Island, Newfoundland, and Labrador.

Dose optimization thresholds: High: A treatment interval decrease of ≥11 days (1.57 weeks) with a posterior interval of ≤35 days (5 weeks), and/or a dose level increase of ≥1.5 mg/kg with a posterior dose level of ≥9 mg/kg. Low: A treatment interval decrease of ≥11 days (1.57 weeks) with a posterior interval of ≤46 days (6.57 weeks), and/or dose level increase of ≥1.5 mg/kg with a posterior dose level of ≥7 mg/kg.

A=First TDM serum IFX concentration below threshold and no dose optimization within post-index period;

B=First TDM serum IFX concentration below threshold and dose optimization within post-index period;

C=First TDM serum IFX concentration at or above threshold and no dose optimization within post-index period;

D=First TDM serum IFX concentration at or above threshold and dose optimization within post-index period.

Suppl Table 2l: Subgroup Demographics and Baseline Characteristics of Patients with CD with no DO (Based on Low Threshold Criteria) Prior to their First Instance of TDM (Serum IFX Threshold: 10 μg/mL; Post-Index Period for Dose Optimization: +17 weeks; Post-Index Dose Optimization Threshold: High)

|  | **A (N=1076)** | **B (N=319)** | **C (N=284)** | **D (N=5)** | **p-value** |
| --- | --- | --- | --- | --- | --- |
| **Age (years)** | | | | | 0.1622 |
| Mean (SD) | 43.3 (15.37) | 43.5 (15.57) | 41.1 (14.99) | 44.6 (11.67) |  |
| **Age group (years)** | | | | | 0.0942 |
| 18 to 64 | 961 (89.3%) | 290 (90.9%) | 267 (94.0%) | 5 (100.0%) |  |
| 65 to 90 | 115 (10.7%) | 29 (9.1%) | 17 (6.0%) | 0 (0.0%) |  |
| **Gender** | | | | | 0.0724 |
| Female | 550 (51.1%) | 163 (51.1%) | 169 (59.5%) | 2 (40.0%) |  |
| Male | 526 (48.9%) | 156 (48.9%) | 115 (40.5%) | 3 (60.0%) |  |
| **First recorded weight** | | | | | 0.7796 |
| Mean (SD) | 74.65 (17.703) | 73.91 (18.468) | 73.67 (17.264) | 77.82 (18.359) |  |
| Missing | 2 | 1 | 0 | 0 |  |
| **Province/region of treating physician** | | | | | <0.0001 |
| Alberta | 29 (2.7%) | 10 (3.1%) | 14 (4.9%) | 0 (0.0%) |  |
| Atlantic^†^ | 199 (18.5%) | 39 (12.2%) | 71 (25.0%) | 0 (0.0%) |  |
| British Columbia | 119 (11.1%) | 32 (10.0%) | 32 (11.3%) | 0 (0.0%) |  |
| Ontario | 540 (50.2%) | 170 (53.3%) | 140 (49.3%) | 0 (0.0%) |  |
| Quebec | 185 (17.2%) | 68 (21.3%) | 25 (8.8%) | 5 (100.0%) |  |
| Saskatchewan/  Manitoba | 4 (0.4%) | 0 (0.0%) | 2 (0.7%) | 0 (0.0%) |  |
| **Year of initiation of IFX treatment** | | | | | 0.0004 |
| 2015 | 149 (13.8%) | 21 (6.6%) | 36 (12.7%) | 2 (40.0%) |  |
| 2016 | 213 (19.8%) | 52 (16.3%) | 36 (12.7%) | 0 (0.0%) |  |
| 2017 | 376 (34.9%) | 127 (39.8%) | 118 (41.5%) | 3 (60.0%) |  |
| 2018 | 338 (31.4%) | 119 (37.3%) | 94 (33.1%) | 0 (0.0%) |  |
| **Time to first TDM (days)** | | | | | <0.0001 |
| Mean (SD) | 375.6 (327.38) | 281.3 (274.30) | 310.1 (317.99) | 403.4 (410.89) |  |
| **Year of first instance of TDM** | | | | | 0.3874 |
| 2015 | 0 (0.0%) | 0 (0.0%) | 1 (0.4%) | 0 (0.0%) |  |
| 2016 | 3 (0.3%) | 0 (0.0%) | 0 (0.0%) | 0 (0.0%) |  |
| 2017 | 350 (32.5%) | 112 (35.1%) | 107 (37.7%) | 3 (60.0%) |  |
| 2018 | 507 (47.1%) | 141 (44.2%) | 129 (45.4%) | 2 (40.0%) |  |
| 2019 | 216 (20.1%) | 66 (20.7%) | 47 (16.5%) | 0 (0.0%) |  |

TDM=therapeutic drug monitoring. P-value: categorical variables, chi-square test; numeric variables, ANOVA.

^†^Atlantic includes New Brunswick, Nova Scotia, Prince Edward Island, Newfoundland, and Labrador.

Dose optimization thresholds: High: A treatment interval decrease of ≥11 days (1.57 weeks) with a posterior interval of ≤35 days (5 weeks), and/or a dose level increase of ≥1.5 mg/kg with a posterior dose level of ≥9 mg/kg. Low: A treatment interval decrease of ≥11 days (1.57 weeks) with a posterior interval of ≤46 days (6.57 weeks), and/or dose level increase of ≥1.5 mg/kg with a posterior dose level of ≥7 mg/kg.

A=First TDM serum IFX concentration below threshold and no dose optimization within post-index period;

B=First TDM serum IFX concentration below threshold and dose optimization within post-index period;

C=First TDM serum IFX concentration at or above threshold and no dose optimization within post-index period;

D=First TDM serum IFX concentration at or above threshold and dose optimization within post-index period.

Suppl Table 3a: Subgroup Demographics and Baseline Characteristics of Patients with CD with no DO (Based on High Threshold Criteria) Prior to their First Instance of TDM (Serum IFX Threshold: 3 μg/mL; Post-Index Period for Dose Optimization: +9 weeks; Post-Index Dose Optimization Threshold: Low)

|  | **A (N=442)** | **B (N=340)** | **C (N=970)** | **D (N=125)** | **p-value** |
| --- | --- | --- | --- | --- | --- |
| **Age (years)** | | | | | <0.0001 |
| Mean (SD) | 45.6 (15.57) | 44.0 (15.52) | 41.7 (15.11) | 42.1 (14.44) |  |
| **Age group (years)** | | | | | 0.0023 |
| 18 to 64 | 379 (85.7%) | 308 (90.6%) | 888 (91.5%) | 118 (94.4%) |  |
| 65 to 90 | 63 (14.3%) | 32 (9.4%) | 82 (8.5%) | 7 (5.6%) |  |
| **Gender** | | | | | 0.3538 |
| Female | 218 (49.3%) | 182 (53.5%) | 524 (54.0%) | 62 (49.6%) |  |
| Male | 224 (50.7%) | 158 (46.5%) | 446 (46.0%) | 63 (50.4%) |  |
| **First recorded weight** | | | | | 0.2185 |
| Mean (SD) | 74.85 (19.416) | 72.55 (18.081) | 74.69 (16.864) | 75.15 (17.365) |  |
| Missing | 1 | 0 | 2 | 0 |  |
| **Province/region of treating physician** | | | | | 0.0011 |
| Alberta | 12 (2.7%) | 9 (2.6%) | 32 (3.3%) | 4 (3.2%) |  |
| Atlantic^†^ | 86 (19.5%) | 45 (13.2%) | 196 (20.2%) | 18 (14.4%) |  |
| British Columbia | 42 (9.5%) | 29 (8.5%) | 117 (12.1%) | 12 (9.6%) |  |
| Ontario | 204 (46.2%) | 184 (54.1%) | 483 (49.8%) | 59 (47.2%) |  |
| Quebec | 97 (21.9%) | 73 (21.5%) | 136 (14.0%) | 32 (25.6%) |  |
| Saskatchewan/  Manitoba | 1 (0.2%) | 0 (0.0%) | 6 (0.6%) | 0 (0.0%) |  |
| **Year of initiation of IFX treatment** | | | | | 0.0006 |
| 2015 | 65 (14.7%) | 28 (8.2%) | 169 (17.4%) | 17 (13.6%) |  |
| 2016 | 107 (24.2%) | 63 (18.5%) | 180 (18.6%) | 17 (13.6%) |  |
| 2017 | 151 (34.2%) | 128 (37.6%) | 333 (34.3%) | 51 (40.8%) |  |
| 2018 | 119 (26.9%) | 121 (35.6%) | 288 (29.7%) | 40 (32.0%) |  |
| **Time to first TDM (days)** | | | | | <0.0001 |
| Mean (SD) | 423.1 (349.38) | 305.9 (285.10) | 383.5 (345.35) | 347.0 (316.58) |  |
| **Year of first instance of TDM** | | | | | 0.1051 |
| 2015 | 0 (0.0%) | 0 (0.0%) | 1 (0.1%) | 0 (0.0%) |  |
| 2016 | 1 (0.2%) | 0 (0.0%) | 2 (0.2%) | 0 (0.0%) |  |
| 2017 | 149 (33.7%) | 122 (35.9%) | 353 (36.4%) | 36 (28.8%) |  |
| 2018 | 189 (42.8%) | 142 (41.8%) | 450 (46.4%) | 70 (56.0%) |  |
| 2019 | 103 (23.3%) | 76 (22.4%) | 164 (16.9%) | 19 (15.2%) |  |

TDM=therapeutic drug monitoring. P-value: categorical variables, chi-square test; numeric variables, ANOVA.

^†^Atlantic includes New Brunswick, Nova Scotia, Prince Edward Island, Newfoundland, and Labrador.

Dose optimization thresholds: High: A treatment interval decrease of ≥11 days (1.57 weeks) with a posterior interval of ≤35 days (5 weeks), and/or a dose level increase of ≥1.5 mg/kg with a posterior dose level of ≥9 mg/kg. Low: A treatment interval decrease of ≥11 days (1.57 weeks) with a posterior interval of ≤46 days (6.57 weeks), and/or dose level increase of ≥1.5 mg/kg with a posterior dose level of ≥7 mg/kg.

A=First TDM serum IFX concentration below threshold and no dose optimization within post-index period;

B=First TDM serum IFX concentration below threshold and dose optimization within post-index period;

C=First TDM serum IFX concentration at or above threshold and no dose optimization within post-index period;

D=First TDM serum IFX concentration at or above threshold and dose optimization within post-index period.

Suppl Table 3b: Subgroup Demographics and Baseline Characteristics of Patients with CD with no DO (Based on High Threshold Criteria) Prior to their First Instance of TDM (Serum IFX Threshold: 3 μg/mL; Post-Index Period for Dose Optimization: +17 weeks; Post-Index Dose Optimization Threshold: Low)

|  | **A (N=358)** | **B (N=424)** | **C (N=904)** | **D (N=191)** | **p-value** |
| --- | --- | --- | --- | --- | --- |
| **Age (years)** | | | | | <0.0001 |
| Mean (SD) | 46.1 (15.57) | 43.9 (15.50) | 41.4 (15.01) | 43.1 (15.08) |  |
| **Age group (years)** | | | | | 0.0020 |
| 18 to 64 | 304 (84.9%) | 383 (90.3%) | 830 (91.8%) | 176 (92.1%) |  |
| 65 to 90 | 54 (15.1%) | 41 (9.7%) | 74 (8.2%) | 15 (7.9%) |  |
| **Gender** | | | | | 0.4734 |
| Female | 175 (48.9%) | 225 (53.1%) | 486 (53.8%) | 100 (52.4%) |  |
| Male | 183 (51.1%) | 199 (46.9%) | 418 (46.2%) | 91 (47.6%) |  |
| **First recorded weight** | | | | | 0.2427 |
| Mean (SD) | 75.04 (19.656) | 72.85 (18.143) | 74.66 (16.955) | 75.11 (16.759) |  |
| Missing | 1 | 0 | 0 | 2 |  |
| **Province/region of treating physician** | | | | | 0.0008 |
| Alberta | 10 (2.8%) | 11 (2.6%) | 32 (3.5%) | 4 (2.1%) |  |
| Atlantic^†^ | 75 (20.9%) | 56 (13.2%) | 182 (20.1%) | 32 (16.8%) |  |
| British Columbia | 34 (9.5%) | 37 (8.7%) | 105 (11.6%) | 24 (12.6%) |  |
| Ontario | 160 (44.7%) | 228 (53.8%) | 453 (50.1%) | 89 (46.6%) |  |
| Quebec | 78 (21.8%) | 92 (21.7%) | 126 (13.9%) | 42 (22.0%) |  |
| Saskatchewan/  Manitoba | 1 (0.3%) | 0 (0.0%) | 6 (0.7%) | 0 (0.0%) |  |
| **Year of initiation of IFX treatment** | | | | | <0.0001 |
| 2015 | 52 (14.5%) | 41 (9.7%) | 158 (17.5%) | 28 (14.7%) |  |
| 2016 | 94 (26.3%) | 76 (17.9%) | 175 (19.4%) | 22 (11.5%) |  |
| 2017 | 121 (33.8%) | 158 (37.3%) | 306 (33.8%) | 78 (40.8%) |  |
| 2018 | 91 (25.4%) | 149 (35.1%) | 265 (29.3%) | 63 (33.0%) |  |
| **Time to first TDM (days)** | | | | | 0.0001 |
| Mean (SD) | 427.1 (338.73) | 325.8 (311.60) | 387.7 (344.14) | 340.0 (331.21) |  |
| **Year of first instance of TDM** | | | | | 0.1084 |
| 2015 | 0 (0.0%) | 0 (0.0%) | 1 (0.1%) | 0 (0.0%) |  |
| 2016 | 1 (0.3%) | 0 (0.0%) | 2 (0.2%) | 0 (0.0%) |  |
| 2017 | 125 (34.9%) | 146 (34.4%) | 329 (36.4%) | 60 (31.4%) |  |
| 2018 | 152 (42.5%) | 179 (42.2%) | 417 (46.1%) | 103 (53.9%) |  |
| 2019 | 80 (22.3%) | 99 (23.3%) | 155 (17.1%) | 28 (14.7%) |  |

TDM=therapeutic drug monitoring. P-value: categorical variables, chi-square test; numeric variables, ANOVA.

^†^Atlantic includes New Brunswick, Nova Scotia, Prince Edward Island, Newfoundland, and Labrador.

Dose optimization thresholds: High: A treatment interval decrease of ≥11 days (1.57 weeks) with a posterior interval of ≤35 days (5 weeks), and/or a dose level increase of ≥1.5 mg/kg with a posterior dose level of ≥9 mg/kg. Low: A treatment interval decrease of ≥11 days (1.57 weeks) with a posterior interval of ≤46 days (6.57 weeks), and/or dose level increase of ≥1.5 mg/kg with a posterior dose level of ≥7 mg/kg.

A=First TDM serum IFX concentration below threshold and no dose optimization within post-index period;

B=First TDM serum IFX concentration below threshold and dose optimization within post-index period;

C=First TDM serum IFX concentration at or above threshold and no dose optimization within post-index period;

D=First TDM serum IFX concentration at or above threshold and dose optimization within post-index period.

Suppl Table 3c: Subgroup Demographics and Baseline Characteristics of Patients with CD with no DO (Based on High Threshold Criteria) Prior to their First Instance of TDM (Serum IFX Threshold: 5 μg/mL; Post-Index Period for Dose Optimization: +9 weeks; Post-Index Dose Optimization Threshold: Low)

|  | **A (N=695)** | **B (N=407)** | **C (N=717)** | **D (N=58)** | **p-value** |
| --- | --- | --- | --- | --- | --- |
| **Age (years)** | | | | | 0.0006 |
| Mean (SD) | 44.6 (15.57) | 43.8 (15.67) | 41.3 (14.99) | 41.5 (11.73) |  |
| **Age group (years)** | | | | | 0.0012 |
| 18 to 64 | 605 (87.1%) | 369 (90.7%) | 662 (92.3%) | 57 (98.3%) |  |
| 65 to 90 | 90 (12.9%) | 38 (9.3%) | 55 (7.7%) | 1 (1.7%) |  |
| **Gender** | | | | | 0.0242 |
| Female | 338 (48.6%) | 210 (51.6%) | 404 (56.3%) | 34 (58.6%) |  |
| Male | 357 (51.4%) | 197 (48.4%) | 313 (43.7%) | 24 (41.4%) |  |
| **First recorded weight** | | | | | 0.2814 |
| Mean (SD) | 75.27 (18.338) | 73.13 (18.146) | 74.22 (17.045) | 74.07 (16.283) |  |
| Missing | 1 | 0 | 2 | 0 |  |
| **Province/region of treating physician** | | | | | 0.0005 |
| Alberta | 20 (2.9%) | 10 (2.5%) | 24 (3.3%) | 3 (5.2%) |  |
| Atlantic^†^ | 134 (19.3%) | 54 (13.3%) | 148 (20.6%) | 9 (15.5%) |  |
| British Columbia | 69 (9.9%) | 36 (8.8%) | 90 (12.6%) | 5 (8.6%) |  |
| Ontario | 325 (46.8%) | 215 (52.8%) | 362 (50.5%) | 28 (48.3%) |  |
| Quebec | 144 (20.7%) | 92 (22.6%) | 89 (12.4%) | 13 (22.4%) |  |
| Saskatchewan/  Manitoba | 3 (0.4%) | 0 (0.0%) | 4 (0.6%) | 0 (0.0%) |  |
| **Year of initiation of IFX treatment** | | | | | <0.0001 |
| 2015 | 102 (14.7%) | 38 (9.3%) | 132 (18.4%) | 7 (12.1%) |  |
| 2016 | 169 (24.3%) | 72 (17.7%) | 118 (16.5%) | 8 (13.8%) |  |
| 2017 | 228 (32.8%) | 152 (37.3%) | 256 (35.7%) | 27 (46.6%) |  |
| 2018 | 196 (28.2%) | 145 (35.6%) | 211 (29.4%) | 16 (27.6%) |  |
| **Time to first TDM (days)** | | | | | <0.0001 |
| Mean (SD) | 412.5 (340.22) | 313.7 (290.75) | 379.9 (352.91) | 339.7 (318.48) |  |
| **Year of first instance of TDM** | | | | | 0.3040 |
| 2015 | 0 (0.0%) | 0 (0.0%) | 1 (0.1%) | 0 (0.0%) |  |
| 2016 | 2 (0.3%) | 0 (0.0%) | 1 (0.1%) | 0 (0.0%) |  |
| 2017 | 235 (33.8%) | 140 (34.4%) | 267 (37.2%) | 18 (31.0%) |  |
| 2018 | 307 (44.2%) | 180 (44.2%) | 332 (46.3%) | 32 (55.2%) |  |
| 2019 | 151 (21.7%) | 87 (21.4%) | 116 (16.2%) | 8 (13.8%) |  |

TDM=therapeutic drug monitoring. P-value: categorical variables, chi-square test; numeric variables, ANOVA.

^†^Atlantic includes New Brunswick, Nova Scotia, Prince Edward Island, Newfoundland, and Labrador.

Dose optimization thresholds: High: A treatment interval decrease of ≥11 days (1.57 weeks) with a posterior interval of ≤35 days (5 weeks), and/or a dose level increase of ≥1.5 mg/kg with a posterior dose level of ≥9 mg/kg. Low: A treatment interval decrease of ≥11 days (1.57 weeks) with a posterior interval of ≤46 days (6.57 weeks), and/or dose level increase of ≥1.5 mg/kg with a posterior dose level of ≥7 mg/kg.

A=First TDM serum IFX concentration below threshold and no dose optimization within post-index period;

B=First TDM serum IFX concentration below threshold and dose optimization within post-index period;

C=First TDM serum IFX concentration at or above threshold and no dose optimization within post-index period;

D=First TDM serum IFX concentration at or above threshold and dose optimization within post-index period.

Suppl Table 3d: Subgroup Demographics and Baseline Characteristics of Patients with CD with no DO (Based on High Threshold Criteria) Prior to their First Instance of TDM (Serum IFX Threshold: 5 μg/mL; Post-Index Period for Dose Optimization: +17 weeks; Post-Index Dose Optimization Threshold: Low)

|  | **A (N=583)** | **B (N=519)** | **C (N=679)** | **D (N=96)** | **p-value** |
| --- | --- | --- | --- | --- | --- |
| **Age (years)** | | | | | 0.0004 |
| Mean (SD) | 44.6 (15.46) | 43.8 (15.78) | 41.2 (15.01) | 42.5 (12.90) |  |
| **Age group (years)** | | | | | 0.0029 |
| 18 to 64 | 506 (86.8%) | 468 (90.2%) | 628 (92.5%) | 91 (94.8%) |  |
| 65 to 90 | 77 (13.2%) | 51 (9.8%) | 51 (7.5%) | 5 (5.2%) |  |
| **Gender** | | | | | 0.0162 |
| Female | 279 (47.9%) | 269 (51.8%) | 382 (56.3%) | 56 (58.3%) |  |
| Male | 304 (52.1%) | 250 (48.2%) | 297 (43.7%) | 40 (41.7%) |  |
| **First recorded weight** | | | | | 0.1485 |
| Mean (SD) | 75.59 (18.470) | 73.23 (18.018) | 74.06 (17.099) | 75.29 (16.134) |  |
| Missing | 1 | 0 | 0 | 2 |  |
| **Province/region of treating physician** | | | | | 0.0006 |
| Alberta | 18 (3.1%) | 12 (2.3%) | 24 (3.5%) | 3 (3.1%) |  |
| Atlantic^†^ | 117 (20.1%) | 71 (13.7%) | 140 (20.6%) | 17 (17.7%) |  |
| British Columbia | 56 (9.6%) | 49 (9.4%) | 83 (12.2%) | 12 (12.5%) |  |
| Ontario | 269 (46.1%) | 271 (52.2%) | 344 (50.7%) | 46 (47.9%) |  |
| Quebec | 120 (20.6%) | 116 (22.4%) | 84 (12.4%) | 18 (18.8%) |  |
| Saskatchewan/  Manitoba | 3 (0.5%) | 0 (0.0%) | 4 (0.6%) | 0 (0.0%) |  |
| **Year of initiation of IFX treatment** | | | | | <0.0001 |
| 2015 | 85 (14.6%) | 55 (10.6%) | 125 (18.4%) | 14 (14.6%) |  |
| 2016 | 154 (26.4%) | 87 (16.8%) | 115 (16.9%) | 11 (11.5%) |  |
| 2017 | 187 (32.1%) | 193 (37.2%) | 240 (35.3%) | 43 (44.8%) |  |
| 2018 | 157 (26.9%) | 184 (35.5%) | 199 (29.3%) | 28 (29.2%) |  |
| **Time to first TDM (days)** | | | | | <0.0001 |
| Mean (SD) | 419.3 (332.95) | 327.4 (311.65) | 381.3 (350.58) | 345.5 (349.48) |  |
| **Year of first instance of TDM** | | | | | 0.2504 |
| 2015 | 0 (0.0%) | 0 (0.0%) | 1 (0.1%) | 0 (0.0%) |  |
| 2016 | 2 (0.3%) | 0 (0.0%) | 1 (0.1%) | 0 (0.0%) |  |
| 2017 | 200 (34.3%) | 175 (33.7%) | 254 (37.4%) | 31 (32.3%) |  |
| 2018 | 256 (43.9%) | 231 (44.5%) | 313 (46.1%) | 51 (53.1%) |  |
| 2019 | 125 (21.4%) | 113 (21.8%) | 110 (16.2%) | 14 (14.6%) |  |

TDM=therapeutic drug monitoring. P-value: categorical variables, chi-square test; numeric variables, ANOVA.

^†^Atlantic includes New Brunswick, Nova Scotia, Prince Edward Island, Newfoundland, and Labrador.

Dose optimization thresholds: High: A treatment interval decrease of ≥11 days (1.57 weeks) with a posterior interval of ≤35 days (5 weeks), and/or a dose level increase of ≥1.5 mg/kg with a posterior dose level of ≥9 mg/kg. Low: A treatment interval decrease of ≥11 days (1.57 weeks) with a posterior interval of ≤46 days (6.57 weeks), and/or dose level increase of ≥1.5 mg/kg with a posterior dose level of ≥7 mg/kg.

A=First TDM serum IFX concentration below threshold and no dose optimization within post-index period;

B=First TDM serum IFX concentration below threshold and dose optimization within post-index period;

C=First TDM serum IFX concentration at or above threshold and no dose optimization within post-index period;

D=First TDM serum IFX concentration at or above threshold and dose optimization within post-index period.

Suppl Table 3e: Subgroup Demographics and Baseline Characteristics of Patients with CD with no DO (Based on High Threshold Criteria) Prior to their First Instance of TDM (Serum IFX Threshold: 10 μg/mL; Post-Index Period for Dose Optimization: +9 weeks; Post-Index Dose Optimization Threshold: Low)

|  | **A (N=1092)** | **B (N=452)** | **C (N=320)** | **D (N=13)** | **p-value** |
| --- | --- | --- | --- | --- | --- |
| **Age (years)** | | | | | 0.1930 |
| Mean (SD) | 43.3 (15.40) | 43.6 (15.37) | 41.5 (15.15) | 40.5 (10.10) |  |
| **Age group (years)** | | | | | 0.0307 |
| 18 to 64 | 968 (88.6%) | 413 (91.4%) | 299 (93.4%) | 13 (100.0%) |  |
| 65 to 90 | 124 (11.4%) | 39 (8.6%) | 21 (6.6%) | 0 (0.0%) |  |
| **Gender** | | | | | 0.0063 |
| Female | 548 (50.2%) | 235 (52.0%) | 194 (60.6%) | 9 (69.2%) |  |
| Male | 544 (49.8%) | 217 (48.0%) | 126 (39.4%) | 4 (30.8%) |  |
| **First recorded weight** | | | | | 0.0964 |
| Mean (SD) | 75.20 (17.895) | 73.13 (17.842) | 73.18 (16.932) | 77.31 (20.514) |  |
| Missing | 3 | 0 | 0 | 0 |  |
| **Province/region of treating physician** | | | | | <0.0001 |
| Alberta | 32 (2.9%) | 10 (2.2%) | 12 (3.8%) | 3 (23.1%) |  |
| Atlantic^†^ | 204 (18.7%) | 59 (13.1%) | 78 (24.4%) | 4 (30.8%) |  |
| British Columbia | 121 (11.1%) | 41 (9.1%) | 38 (11.9%) | 0 (0.0%) |  |
| Ontario | 534 (48.9%) | 241 (53.3%) | 153 (47.8%) | 2 (15.4%) |  |
| Quebec | 197 (18.0%) | 101 (22.3%) | 36 (11.3%) | 4 (30.8%) |  |
| Saskatchewan/  Manitoba | 4 (0.4%) | 0 (0.0%) | 3 (0.9%) | 0 (0.0%) |  |
| **Year of initiation of IFX treatment** | | | | | 0.0003 |
| 2015 | 184 (16.8%) | 41 (9.1%) | 50 (15.6%) | 4 (30.8%) |  |
| 2016 | 237 (21.7%) | 78 (17.3%) | 50 (15.6%) | 2 (15.4%) |  |
| 2017 | 360 (33.0%) | 175 (38.7%) | 124 (38.8%) | 4 (30.8%) |  |
| 2018 | 311 (28.5%) | 158 (35.0%) | 96 (30.0%) | 3 (23.1%) |  |
| **Time to first TDM (days)** | | | | | <0.0001 |
| Mean (SD) | 410.8 (348.86) | 313.7 (291.34) | 345.3 (336.12) | 431.5 (374.15) |  |
| **Year of first instance of TDM** | | | | | 0.3702 |
| 2015 | 0 (0.0%) | 0 (0.0%) | 1 (0.3%) | 0 (0.0%) |  |
| 2016 | 3 (0.3%) | 0 (0.0%) | 0 (0.0%) | 0 (0.0%) |  |
| 2017 | 375 (34.3%) | 152 (33.6%) | 127 (39.7%) | 6 (46.2%) |  |
| 2018 | 497 (45.5%) | 207 (45.8%) | 142 (44.4%) | 5 (38.5%) |  |
| 2019 | 217 (19.9%) | 93 (20.6%) | 50 (15.6%) | 2 (15.4%) |  |

TDM=therapeutic drug monitoring. P-value: categorical variables, chi-square test; numeric variables, ANOVA.

^†^Atlantic includes New Brunswick, Nova Scotia, Prince Edward Island, Newfoundland, and Labrador.

Dose optimization thresholds: High: A treatment interval decrease of ≥11 days (1.57 weeks) with a posterior interval of ≤35 days (5 weeks), and/or a dose level increase of ≥1.5 mg/kg with a posterior dose level of ≥9 mg/kg. Low: A treatment interval decrease of ≥11 days (1.57 weeks) with a posterior interval of ≤46 days (6.57 weeks), and/or dose level increase of ≥1.5 mg/kg with a posterior dose level of ≥7 mg/kg.

A=First TDM serum IFX concentration below threshold and no dose optimization within post-index period;

B=First TDM serum IFX concentration below threshold and dose optimization within post-index period;

C=First TDM serum IFX concentration at or above threshold and no dose optimization within post-index period;

D=First TDM serum IFX concentration at or above threshold and dose optimization within post-index period.

Suppl Table 3f: Subgroup Demographics and Baseline Characteristics of Patients with CD with no DO (Based on High Threshold Criteria) Prior to their First Instance of TDM (Serum IFX Threshold: 10 μg/mL; Post-Index Period for Dose Optimization: +17 weeks; Post-Index Dose Optimization Threshold: Low)

|  | **A (N=950)** | **B (N=594)** | **C (N=312)** | **D (N=21)** | **p-value** |
| --- | --- | --- | --- | --- | --- |
| **Age (years)** | | | | | 0.1141 |
| Mean (SD) | 43.3 (15.35) | 43.6 (15.46) | 41.2 (15.12) | 45.0 (12.35) |  |
| **Age group (years)** | | | | | 0.0576 |
| 18 to 64 | 842 (88.6%) | 539 (90.7%) | 292 (93.6%) | 20 (95.2%) |  |
| 65 to 90 | 108 (11.4%) | 55 (9.3%) | 20 (6.4%) | 1 (4.8%) |  |
| **Gender** | | | | | 0.0050 |
| Female | 471 (49.6%) | 312 (52.5%) | 190 (60.9%) | 13 (61.9%) |  |
| Male | 479 (50.4%) | 282 (47.5%) | 122 (39.1%) | 8 (38.1%) |  |
| **First recorded weight** | | | | | 0.1119 |
| Mean (SD) | 75.30 (17.958) | 73.45 (17.758) | 73.14 (17.043) | 76.39 (17.572) |  |
| Missing | 1 | 2 | 0 | 0 |  |
| **Province/region of treating physician** | | | | | <0.0001 |
| Alberta | 30 (3.2%) | 12 (2.0%) | 12 (3.8%) | 3 (14.3%) |  |
| Atlantic^†^ | 181 (19.1%) | 82 (13.8%) | 76 (24.4%) | 6 (28.6%) |  |
| British Columbia | 102 (10.7%) | 60 (10.1%) | 37 (11.9%) | 1 (4.8%) |  |
| Ontario | 461 (48.5%) | 314 (52.9%) | 152 (48.7%) | 3 (14.3%) |  |
| Quebec | 172 (18.1%) | 126 (21.2%) | 32 (10.3%) | 8 (38.1%) |  |
| Saskatchewan/  Manitoba | 4 (0.4%) | 0 (0.0%) | 3 (1.0%) | 0 (0.0%) |  |
| **Year of initiation of IFX treatment** | | | | | <0.0001 |
| 2015 | 162 (17.1%) | 63 (10.6%) | 48 (15.4%) | 6 (28.6%) |  |
| 2016 | 220 (23.2%) | 95 (16.0%) | 49 (15.7%) | 3 (14.3%) |  |
| 2017 | 307 (32.3%) | 228 (38.4%) | 120 (38.5%) | 8 (38.1%) |  |
| 2018 | 261 (27.5%) | 208 (35.0%) | 95 (30.4%) | 4 (19.0%) |  |
| **Time to first TDM (days)** | | | | | <0.0001 |
| Mean (SD) | 417.3 (344.40) | 326.5 (314.09) | 342.9 (332.79) | 435.2 (400.17) |  |
| **Year of first instance of TDM** | | | | | 0.2524 |
| 2015 | 0 (0.0%) | 0 (0.0%) | 1 (0.3%) | 0 (0.0%) |  |
| 2016 | 3 (0.3%) | 0 (0.0%) | 0 (0.0%) | 0 (0.0%) |  |
| 2017 | 330 (34.7%) | 197 (33.2%) | 124 (39.7%) | 9 (42.9%) |  |
| 2018 | 432 (45.5%) | 272 (45.8%) | 137 (43.9%) | 10 (47.6%) |  |
| 2019 | 185 (19.5%) | 125 (21.0%) | 50 (16.0%) | 2 (9.5%) |  |

TDM=therapeutic drug monitoring. P-value: categorical variables, chi-square test; numeric variables, ANOVA.

^†^Atlantic includes New Brunswick, Nova Scotia, Prince Edward Island, Newfoundland, and Labrador.

Dose optimization thresholds: High: A treatment interval decrease of ≥11 days (1.57 weeks) with a posterior interval of ≤35 days (5 weeks), and/or a dose level increase of ≥1.5 mg/kg with a posterior dose level of ≥9 mg/kg. Low: A treatment interval decrease of ≥11 days (1.57 weeks) with a posterior interval of ≤46 days (6.57 weeks), and/or dose level increase of ≥1.5 mg/kg with a posterior dose level of ≥7 mg/kg.

A=First TDM serum IFX concentration below threshold and no dose optimization within post-index period;

B=First TDM serum IFX concentration below threshold and dose optimization within post-index period;

C=First TDM serum IFX concentration at or above threshold and no dose optimization within post-index period;

D=First TDM serum IFX concentration at or above threshold and dose optimization within post-index period.

Suppl Table 3g: Subgroup Demographics and Baseline Characteristics of Patients with CD with no DO (Based on High Threshold Criteria) Prior to their First Instance of TDM (Serum IFX Threshold: 3 μg/mL; Post-Index Period for Dose Optimization: +9 weeks; Post-Index Dose Optimization Threshold: High)

|  | **A (N=564)** | **B (N=218)** | **C (N=1045)** | **D (N=50)** | **p-value** |
| --- | --- | --- | --- | --- | --- |
| **Age (years)** | | | | | <0.0001 |
| Mean (SD) | 45.6 (15.51) | 43.2 (15.60) | 41.6 (15.00) | 44.2 (15.68) |  |
| **Age group (years)** | | | | | 0.0009 |
| 18 to 64 | 485 (86.0%) | 202 (92.7%) | 961 (92.0%) | 45 (90.0%) |  |
| 65 to 90 | 79 (14.0%) | 16 (7.3%) | 84 (8.0%) | 5 (10.0%) |  |
| **Gender** | | | | | 0.2658 |
| Female | 279 (49.5%) | 121 (55.5%) | 562 (53.8%) | 24 (48.0%) |  |
| Male | 285 (50.5%) | 97 (44.5%) | 483 (46.2%) | 26 (52.0%) |  |
| **First recorded weight** | | | | | 0.4792 |
| Mean (SD) | 74.29 (18.858) | 72.72 (18.892) | 74.70 (16.927) | 75.52 (16.802) |  |
| Missing | 1 | 0 | 2 | 0 |  |
| **Province/region of treating physician** | | | | | 0.0047 |
| Alberta | 12 (2.1%) | 9 (4.1%) | 35 (3.3%) | 1 (2.0%) |  |
| Atlantic^†^ | 106 (18.8%) | 25 (11.5%) | 208 (19.9%) | 6 (12.0%) |  |
| British Columbia | 52 (9.2%) | 19 (8.7%) | 123 (11.8%) | 6 (12.0%) |  |
| Ontario | 272 (48.2%) | 116 (53.2%) | 519 (49.7%) | 23 (46.0%) |  |
| Quebec | 121 (21.5%) | 49 (22.5%) | 154 (14.7%) | 14 (28.0%) |  |
| Saskatchewan/  Manitoba | 1 (0.2%) | 0 (0.0%) | 6 (0.6%) | 0 (0.0%) |  |
| **Year of initiation of IFX treatment** | | | | | 0.0117 |
| 2015 | 75 (13.3%) | 18 (8.3%) | 180 (17.2%) | 6 (12.0%) |  |
| 2016 | 130 (23.0%) | 40 (18.3%) | 189 (18.1%) | 8 (16.0%) |  |
| 2017 | 199 (35.3%) | 80 (36.7%) | 363 (34.7%) | 21 (42.0%) |  |
| 2018 | 160 (28.4%) | 80 (36.7%) | 313 (30.0%) | 15 (30.0%) |  |
| **Time to first TDM (days)** | | | | | 0.0035 |
| Mean (SD) | 398.0 (336.44) | 305.3 (295.50) | 382.0 (344.63) | 324.3 (285.41) |  |
| **Year of first instance of TDM** | | | | | 0.1440 |
| 2015 | 0 (0.0%) | 0 (0.0%) | 1 (0.1%) | 0 (0.0%) |  |
| 2016 | 1 (0.2%) | 0 (0.0%) | 2 (0.2%) | 0 (0.0%) |  |
| 2017 | 191 (33.9%) | 80 (36.7%) | 375 (35.9%) | 14 (28.0%) |  |
| 2018 | 244 (43.3%) | 87 (39.9%) | 490 (46.9%) | 30 (60.0%) |  |
| 2019 | 128 (22.7%) | 51 (23.4%) | 177 (16.9%) | 6 (12.0%) |  |

TDM=therapeutic drug monitoring. P-value: categorical variables, chi-square test; numeric variables, ANOVA.

^†^Atlantic includes New Brunswick, Nova Scotia, Prince Edward Island, Newfoundland, and Labrador.

Dose optimization thresholds: High: A treatment interval decrease of ≥11 days (1.57 weeks) with a posterior interval of ≤35 days (5 weeks), and/or a dose level increase of ≥1.5 mg/kg with a posterior dose level of ≥9 mg/kg. Low: A treatment interval decrease of ≥11 days (1.57 weeks) with a posterior interval of ≤46 days (6.57 weeks), and/or dose level increase of ≥1.5 mg/kg with a posterior dose level of ≥7 mg/kg.

A=First TDM serum IFX concentration below threshold and no dose optimization within post-index period;

B=First TDM serum IFX concentration below threshold and dose optimization within post-index period;

C=First TDM serum IFX concentration at or above threshold and no dose optimization within post-index period;

D=First TDM serum IFX concentration at or above threshold and dose optimization within post-index period.

Suppl Table 3h: Subgroup Demographics and Baseline Characteristics of Patients with CD with no DO (Based on High Threshold Criteria) Prior to their First Instance of TDM (Serum IFX Threshold: 3 μg/mL; Post-Index Period for Dose Optimization: +17 weeks; Post-Index Dose Optimization Threshold: High)

|  | **A (N=510)** | **B (N=272)** | **C (N=1017)** | **D (N=78)** | **p-value** |
| --- | --- | --- | --- | --- | --- |
| **Age (years)** | | | | | <0.0001 |
| Mean (SD) | 45.6 (15.63) | 43.7 (15.39) | 41.5 (14.97) | 44.6 (15.60) |  |
| **Age group (years)** | | | | | 0.0021 |
| 18 to 64 | 439 (86.1%) | 248 (91.2%) | 937 (92.1%) | 69 (88.5%) |  |
| 65 to 90 | 71 (13.9%) | 24 (8.8%) | 80 (7.9%) | 9 (11.5%) |  |
| **Gender** | | | | | 0.4228 |
| Female | 256 (50.2%) | 144 (52.9%) | 549 (54.0%) | 37 (47.4%) |  |
| Male | 254 (49.8%) | 128 (47.1%) | 468 (46.0%) | 41 (52.6%) |  |
| **First recorded weight** | | | | | 0.6812 |
| Mean (SD) | 74.07 (18.913) | 73.44 (18.813) | 74.69 (16.960) | 75.45 (16.389) |  |
| Missing | 1 | 0 | 1 | 1 |  |
| **Province/region of treating physician** | | | | | 0.0021 |
| Alberta | 12 (2.4%) | 9 (3.3%) | 35 (3.4%) | 1 (1.3%) |  |
| Atlantic^†^ | 100 (19.6%) | 31 (11.4%) | 202 (19.9%) | 12 (15.4%) |  |
| British Columbia | 48 (9.4%) | 23 (8.5%) | 117 (11.5%) | 12 (15.4%) |  |
| Ontario | 240 (47.1%) | 148 (54.4%) | 508 (50.0%) | 34 (43.6%) |  |
| Quebec | 109 (21.4%) | 61 (22.4%) | 149 (14.7%) | 19 (24.4%) |  |
| Saskatchewan/  Manitoba | 1 (0.2%) | 0 (0.0%) | 6 (0.6%) | 0 (0.0%) |  |
| **Year of initiation of IFX treatment** | | | | | 0.0080 |
| 2015 | 70 (13.7%) | 23 (8.5%) | 176 (17.3%) | 10 (12.8%) |  |
| 2016 | 118 (23.1%) | 52 (19.1%) | 187 (18.4%) | 10 (12.8%) |  |
| 2017 | 177 (34.7%) | 102 (37.5%) | 352 (34.6%) | 32 (41.0%) |  |
| 2018 | 145 (28.4%) | 95 (34.9%) | 302 (29.7%) | 26 (33.3%) |  |
| **Time to first TDM (days)** | | | | | 0.0003 |
| Mean (SD) | 407.2 (339.03) | 306.5 (295.87) | 384.0 (344.34) | 318.8 (309.37) |  |
| **Year of first instance of TDM** | | | | | 0.0636 |
| 2015 | 0 (0.0%) | 0 (0.0%) | 1 (0.1%) | 0 (0.0%) |  |
| 2016 | 1 (0.2%) | 0 (0.0%) | 2 (0.2%) | 0 (0.0%) |  |
| 2017 | 168 (32.9%) | 103 (37.9%) | 365 (35.9%) | 24 (30.8%) |  |
| 2018 | 224 (43.9%) | 107 (39.3%) | 474 (46.6%) | 46 (59.0%) |  |
| 2019 | 117 (22.9%) | 62 (22.8%) | 175 (17.2%) | 8 (10.3%) |  |

TDM=therapeutic drug monitoring. P-value: categorical variables, chi-square test; numeric variables, ANOVA.

^†^Atlantic includes New Brunswick, Nova Scotia, Prince Edward Island, Newfoundland, and Labrador.

Dose optimization thresholds: High: A treatment interval decrease of ≥11 days (1.57 weeks) with a posterior interval of ≤35 days (5 weeks), and/or a dose level increase of ≥1.5 mg/kg with a posterior dose level of ≥9 mg/kg. Low: A treatment interval decrease of ≥11 days (1.57 weeks) with a posterior interval of ≤46 days (6.57 weeks), and/or dose level increase of ≥1.5 mg/kg with a posterior dose level of ≥7 mg/kg.

A=First TDM serum IFX concentration below threshold and no dose optimization within post-index period;

B=First TDM serum IFX concentration below threshold and dose optimization within post-index period;

C=First TDM serum IFX concentration at or above threshold and no dose optimization within post-index period;

D=First TDM serum IFX concentration at or above threshold and dose optimization within post-index period.

Suppl Table 3i: Subgroup Demographics and Baseline Characteristics of Patients with CD with no DO (Based on High Threshold Criteria) Prior to their First Instance of TDM (Serum IFX Threshold: 5 μg/mL; Post-Index Period for Dose Optimization: +9 weeks; Post-Index Dose Optimization Threshold: High)

|  | **A (N=855)** | **B (N=247)** | **C (N=754)** | **D (N=21)** | **p-value** |
| --- | --- | --- | --- | --- | --- |
| **Age (years)** | | | | | 0.0005 |
| Mean (SD) | 44.5 (15.55) | 43.4 (15.80) | 41.3 (14.82) | 42.9 (13.16) |  |
| **Age group (years)** | | | | | 0.0023 |
| 18 to 64 | 747 (87.4%) | 227 (91.9%) | 699 (92.7%) | 20 (95.2%) |  |
| 65 to 90 | 108 (12.6%) | 20 (8.1%) | 55 (7.3%) | 1 (4.8%) |  |
| **Gender** | | | | | 0.0142 |
| Female | 415 (48.5%) | 133 (53.8%) | 426 (56.5%) | 12 (57.1%) |  |
| Male | 440 (51.5%) | 114 (46.2%) | 328 (43.5%) | 9 (42.9%) |  |
| **First recorded weight** | | | | | 0.6361 |
| Mean (SD) | 74.83 (18.098) | 73.29 (18.918) | 74.26 (17.075) | 72.67 (13.316) |  |
| Missing | 1 | 0 | 2 | 0 |  |
| **Province/region of treating physician** | | | | | 0.0003 |
| Alberta | 20 (2.3%) | 10 (4.0%) | 27 (3.6%) | 0 (0.0%) |  |
| Atlantic^†^ | 159 (18.6%) | 29 (11.7%) | 155 (20.6%) | 2 (9.5%) |  |
| British Columbia | 81 (9.5%) | 24 (9.7%) | 94 (12.5%) | 1 (4.8%) |  |
| Ontario | 412 (48.2%) | 128 (51.8%) | 379 (50.3%) | 11 (52.4%) |  |
| Quebec | 180 (21.1%) | 56 (22.7%) | 95 (12.6%) | 7 (33.3%) |  |
| Saskatchewan/  Manitoba | 3 (0.4%) | 0 (0.0%) | 4 (0.5%) | 0 (0.0%) |  |
| **Year of initiation of IFX treatment** | | | | | 0.0017 |
| 2015 | 119 (13.9%) | 21 (8.5%) | 136 (18.0%) | 3 (14.3%) |  |
| 2016 | 196 (22.9%) | 45 (18.2%) | 123 (16.3%) | 3 (14.3%) |  |
| 2017 | 288 (33.7%) | 92 (37.2%) | 274 (36.3%) | 9 (42.9%) |  |
| 2018 | 252 (29.5%) | 89 (36.0%) | 221 (29.3%) | 6 (28.6%) |  |
| **Time to first TDM (days)** | | | | | 0.0036 |
| Mean (SD) | 395.0 (332.57) | 310.3 (294.42) | 379.2 (351.93) | 292.4 (285.03) |  |
| **Year of first instance of TDM** | | | | | 0.2664 |
| 2015 | 0 (0.0%) | 0 (0.0%) | 1 (0.1%) | 0 (0.0%) |  |
| 2016 | 2 (0.2%) | 0 (0.0%) | 1 (0.1%) | 0 (0.0%) |  |
| 2017 | 288 (33.7%) | 87 (35.2%) | 278 (36.9%) | 7 (33.3%) |  |
| 2018 | 383 (44.8%) | 104 (42.1%) | 351 (46.6%) | 13 (61.9%) |  |
| 2019 | 182 (21.3%) | 56 (22.7%) | 123 (16.3%) | 1 (4.8%) |  |

TDM=therapeutic drug monitoring. P-value: categorical variables, chi-square test; numeric variables, ANOVA.

^†^Atlantic includes New Brunswick, Nova Scotia, Prince Edward Island, Newfoundland, and Labrador.

Dose optimization thresholds: High: A treatment interval decrease of ≥11 days (1.57 weeks) with a posterior interval of ≤35 days (5 weeks), and/or a dose level increase of ≥1.5 mg/kg with a posterior dose level of ≥9 mg/kg. Low: A treatment interval decrease of ≥11 days (1.57 weeks) with a posterior interval of ≤46 days (6.57 weeks), and/or dose level increase of ≥1.5 mg/kg with a posterior dose level of ≥7 mg/kg.

A=First TDM serum IFX concentration below threshold and no dose optimization within post-index period;

B=First TDM serum IFX concentration below threshold and dose optimization within post-index period;

C=First TDM serum IFX concentration at or above threshold and no dose optimization within post-index period;

D=First TDM serum IFX concentration at or above threshold and dose optimization within post-index period.

Suppl Table 3j: Subgroup Demographics and Baseline Characteristics of Patients with CD with no DO (Based on High Threshold Criteria) Prior to their First Instance of TDM (Serum IFX Threshold: 5 μg/mL; Post-Index Period for Dose Optimization: +17 weeks; Post-Index Dose Optimization Threshold: High)

|  | **A (N=789)** | **B (N=313)** | **C (N=738)** | **D (N=37)** | **p-value** |
| --- | --- | --- | --- | --- | --- |
| **Age (years)** | | | | | 0.0005 |
| Mean (SD) | 44.4 (15.59) | 43.9 (15.67) | 41.2 (14.84) | 43.6 (13.30) |  |
| **Age group (years)** | | | | | 0.0074 |
| 18 to 64 | 691 (87.6%) | 283 (90.4%) | 685 (92.8%) | 34 (91.9%) |  |
| 65 to 90 | 98 (12.4%) | 30 (9.6%) | 53 (7.2%) | 3 (8.1%) |  |
| **Gender** | | | | | 0.0291 |
| Female | 387 (49.0%) | 161 (51.4%) | 418 (56.6%) | 20 (54.1%) |  |
| Male | 402 (51.0%) | 152 (48.6%) | 320 (43.4%) | 17 (45.9%) |  |
| **First recorded weight** | | | | | 0.8966 |
| Mean (SD) | 74.71 (18.071) | 73.90 (18.840) | 74.23 (17.159) | 73.74 (12.908) |  |
| Missing | 1 | 0 | 1 | 1 |  |
| **Province/region of treating physician** | | | | | 0.0004 |
| Alberta | 20 (2.5%) | 10 (3.2%) | 27 (3.7%) | 0 (0.0%) |  |
| Atlantic^†^ | 151 (19.1%) | 37 (11.8%) | 151 (20.5%) | 6 (16.2%) |  |
| British Columbia | 74 (9.4%) | 31 (9.9%) | 91 (12.3%) | 4 (10.8%) |  |
| Ontario | 375 (47.5%) | 165 (52.7%) | 373 (50.5%) | 17 (45.9%) |  |
| Quebec | 166 (21.0%) | 70 (22.4%) | 92 (12.5%) | 10 (27.0%) |  |
| Saskatchewan/  Manitoba | 3 (0.4%) | 0 (0.0%) | 4 (0.5%) | 0 (0.0%) |  |
| **Year of initiation of IFX treatment** | | | | | 0.0008 |
| 2015 | 112 (14.2%) | 28 (8.9%) | 134 (18.2%) | 5 (13.5%) |  |
| 2016 | 184 (23.3%) | 57 (18.2%) | 121 (16.4%) | 5 (13.5%) |  |
| 2017 | 261 (33.1%) | 119 (38.0%) | 268 (36.3%) | 15 (40.5%) |  |
| 2018 | 232 (29.4%) | 109 (34.8%) | 215 (29.1%) | 12 (32.4%) |  |
| **Time to first TDM (days)** | | | | | 0.0003 |
| Mean (SD) | 402.6 (334.96) | 309.1 (293.00) | 380.2 (350.52) | 310.6 (346.33) |  |
| **Year of first instance of TDM** | | | | | 0.1934 |
| 2015 | 0 (0.0%) | 0 (0.0%) | 1 (0.1%) | 0 (0.0%) |  |
| 2016 | 2 (0.3%) | 0 (0.0%) | 1 (0.1%) | 0 (0.0%) |  |
| 2017 | 260 (33.0%) | 115 (36.7%) | 273 (37.0%) | 12 (32.4%) |  |
| 2018 | 356 (45.1%) | 131 (41.9%) | 342 (46.3%) | 22 (59.5%) |  |
| 2019 | 171 (21.7%) | 67 (21.4%) | 121 (16.4%) | 3 (8.1%) |  |

TDM=therapeutic drug monitoring. P-value: categorical variables, chi-square test; numeric variables, ANOVA.

^†^Atlantic includes New Brunswick, Nova Scotia, Prince Edward Island, Newfoundland, and Labrador.

Dose optimization thresholds: High: A treatment interval decrease of ≥11 days (1.57 weeks) with a posterior interval of ≤35 days (5 weeks), and/or a dose level increase of ≥1.5 mg/kg with a posterior dose level of ≥9 mg/kg. Low: A treatment interval decrease of ≥11 days (1.57 weeks) with a posterior interval of ≤46 days (6.57 weeks), and/or dose level increase of ≥1.5 mg/kg with a posterior dose level of ≥7 mg/kg.

A=First TDM serum IFX concentration below threshold and no dose optimization within post-index period;

B=First TDM serum IFX concentration below threshold and dose optimization within post-index period;

C=First TDM serum IFX concentration at or above threshold and no dose optimization within post-index period;

D=First TDM serum IFX concentration at or above threshold and dose optimization within post-index period.

Suppl Table 3k: Subgroup Demographics and Baseline Characteristics of Patients with CD with no DO (Based on High Threshold Criteria) Prior to their First Instance of TDM (Serum IFX Threshold: 10 μg/mL; Post-Index Period for Dose Optimization: +9 weeks; Post-Index Dose Optimization Threshold: High)

|  | **A (N=1280)** | **B (N=264)** | **C (N=329)** | **D (N=4)** | **p-value** |
| --- | --- | --- | --- | --- | --- |
| **Age (years)** | | | | | 0.2002 |
| Mean (SD) | 43.4 (15.33) | 43.4 (15.68) | 41.4 (15.04) | 42.5 (9.40) |  |
| **Age group (years)** | | | | | 0.0417 |
| 18 to 64 | 1138 (88.9%) | 243 (92.0%) | 308 (93.6%) | 4 (100.0%) |  |
| 65 to 90 | 142 (11.1%) | 21 (8.0%) | 21 (6.4%) | 0 (0.0%) |  |
| **Gender** | | | | | 0.0041 |
| Female | 640 (50.0%) | 143 (54.2%) | 201 (61.1%) | 2 (50.0%) |  |
| Male | 640 (50.0%) | 121 (45.8%) | 128 (38.9%) | 2 (50.0%) |  |
| **First recorded weight** | | | | | 0.3380 |
| Mean (SD) | 74.88 (17.759) | 73.20 (18.528) | 73.32 (17.055) | 75.73 (20.749) |  |
| Missing | 3 | 0 | 0 | 0 |  |
| **Province/region of treating physician** | | | | | <0.0001 |
| Alberta | 32 (2.5%) | 10 (3.8%) | 15 (4.6%) | 0 (0.0%) |  |
| Atlantic^†^ | 233 (18.2%) | 30 (11.4%) | 81 (24.6%) | 1 (25.0%) |  |
| British Columbia | 137 (10.7%) | 25 (9.5%) | 38 (11.6%) | 0 (0.0%) |  |
| Ontario | 636 (49.7%) | 139 (52.7%) | 155 (47.1%) | 0 (0.0%) |  |
| Quebec | 238 (18.6%) | 60 (22.7%) | 37 (11.2%) | 3 (75.0%) |  |
| Saskatchewan/  Manitoba | 4 (0.3%) | 0 (0.0%) | 3 (0.9%) | 0 (0.0%) |  |
| **Year of initiation of IFX treatment** | | | | | 0.0057 |
| 2015 | 203 (15.9%) | 22 (8.3%) | 52 (15.8%) | 2 (50.0%) |  |
| 2016 | 268 (20.9%) | 47 (17.8%) | 51 (15.5%) | 1 (25.0%) |  |
| 2017 | 435 (34.0%) | 100 (37.9%) | 127 (38.6%) | 1 (25.0%) |  |
| 2018 | 374 (29.2%) | 95 (36.0%) | 99 (30.1%) | 0 |  |
| **Time to first TDM (days)** | | | | | 0.0001 |
| Mean (SD) | 398.2 (342.32) | 305.7 (291.35) | 346.6 (336.97) | 520.0 (386.74) |  |
| **Year of first instance of TDM** | | | | | 0.2686 |
| 2015 | 0 (0.0%) | 0 (0.0%) | 1 (0.3%) | 0 (0.0%) |  |
| 2016 | 3 (0.2%) | 0 (0.0%) | 0 (0.0%) | 0 (0.0%) |  |
| 2017 | 436 (34.1%) | 91 (34.5%) | 130 (39.5%) | 3 (75.0%) |  |
| 2018 | 588 (45.9%) | 116 (43.9%) | 146 (44.4%) | 1 (25.0%) |  |
| 2019 | 253 (19.8%) | 57 (21.6%) | 52 (15.8%) | 0 |  |

TDM=therapeutic drug monitoring. P-value: categorical variables, chi-square test; numeric variables, ANOVA.

^†^Atlantic includes New Brunswick, Nova Scotia, Prince Edward Island, Newfoundland, and Labrador.

Dose optimization thresholds: High: A treatment interval decrease of ≥11 days (1.57 weeks) with a posterior interval of ≤35 days (5 weeks), and/or a dose level increase of ≥1.5 mg/kg with a posterior dose level of ≥9 mg/kg. Low: A treatment interval decrease of ≥11 days (1.57 weeks) with a posterior interval of ≤46 days (6.57 weeks), and/or dose level increase of ≥1.5 mg/kg with a posterior dose level of ≥7 mg/kg.

A=First TDM serum IFX concentration below threshold and no dose optimization within post-index period;

B=First TDM serum IFX concentration below threshold and dose optimization within post-index period;

C=First TDM serum IFX concentration at or above threshold and no dose optimization within post-index period;

D=First TDM serum IFX concentration at or above threshold and dose optimization within post-index period.

Suppl Table 3l: Subgroup Demographics and Baseline Characteristics of Patients with CD with no DO (Based on High Threshold Criteria) Prior to their First Instance of TDM (Serum IFX Threshold: 10 μg/mL; Post-Index Period for Dose Optimization: +17 weeks; Post-Index Dose Optimization Threshold: High)

|  | **A (N=1201)** | **B (N=343)** | **C (N=326)** | **D (N=7)** | **p-value** |
| --- | --- | --- | --- | --- | --- |
| **Age (years)** | | | | | 0.1536 |
| Mean (SD) | 43.3 (15.35) | 43.9 (15.51) | 41.4 (15.07) | 44.0 (10.30) |  |
| **Age group (years)** | | | | | 0.0955 |
| 18 to 64 | 1071 (89.2%) | 310 (90.4%) | 305 (93.6%) | 7 (100.0%) |  |
| 65 to 90 | 130 (10.8%) | 33 (9.6%) | 21 (6.4%) | 0 (0.0%) |  |
| **Gender** | | | | | 0.0019 |
| Female | 604 (50.3%) | 179 (52.2%) | 201 (61.7%) | 2 (28.6%) |  |
| Male | 597 (49.7%) | 164 (47.8%) | 125 (38.3%) | 5 (71.4%) |  |
| **First recorded weight** | | | | | 0.3582 |
| Mean (SD) | 74.83 (17.768) | 73.76 (18.351) | 73.21 (17.091) | 79.73 (15.792) |  |
| Missing | 2 | 1 | 0 | 0 |  |
| **Province/region of treating physician** | | | | | <0.0001 |
| Alberta | 32 (2.7%) | 10 (2.9%) | 15 (4.6%) | 0 (0.0%) |  |
| Atlantic^†^ | 221 (18.4%) | 42 (12.2%) | 81 (24.8%) | 1 (14.3%) |  |
| British Columbia | 128 (10.7%) | 34 (9.9%) | 37 (11.3%) | 1 (14.3%) |  |
| Ontario | 593 (49.4%) | 182 (53.1%) | 155 (47.5%) | 0 (0.0%) |  |
| Quebec | 223 (18.6%) | 75 (21.9%) | 35 (10.7%) | 5 (71.4%) |  |
| Saskatchewan/  Manitoba | 4 (0.3%) | 0 (0.0%) | 3 (0.9%) | 0 (0.0%) |  |
| **Year of initiation of IFX treatment** | | | | | 0.0037 |
| 2015 | 194 (16.2%) | 31 (9.0%) | 52 (16.0%) | 2 (28.6%) |  |
| 2016 | 255 (21.2%) | 60 (17.5%) | 50 (15.3%) | 2 (28.6%) |  |
| 2017 | 404 (33.6%) | 131 (38.2%) | 125 (38.3%) | 3 (42.9%) |  |
| 2018 | 348 (29.0%) | 121 (35.3%) | 99 (30.4%) | 0 (0.0%) |  |
| **Time to first TDM (days)** | | | | | <0.0001 |
| Mean (SD) | 403.8 (343.17) | 307.1 (297.46) | 347.3 (337.50) | 414.7 (356.28) |  |
| **Year of first instance of TDM** | | | | | 0.1979 |
| 2015 | 0 (0.0%) | 0 (0.0%) | 1 (0.3%) | 0 (0.0%) |  |
| 2016 | 3 (0.2%) | 0 (0.0%) | 0 (0.0%) | 0 (0.0%) |  |
| 2017 | 405 (33.7%) | 122 (35.6%) | 128 (39.3%) | 5 (71.4%) |  |
| 2018 | 553 (46.0%) | 151 (44.0%) | 145 (44.5%) | 2 (28.6%) |  |
| 2019 | 240 (20.0%) | 70 (20.4%) | 52 (16.0%) | 0 (0.0%) |  |

TDM=therapeutic drug monitoring. P-value: categorical variables, chi-square test; numeric variables, ANOVA.

^†^Atlantic includes New Brunswick, Nova Scotia, Prince Edward Island, Newfoundland, and Labrador.

Dose optimization thresholds: High: A treatment interval decrease of ≥11 days (1.57 weeks) with a posterior interval of ≤35 days (5 weeks), and/or a dose level increase of ≥1.5 mg/kg with a posterior dose level of ≥9 mg/kg. Low: A treatment interval decrease of ≥11 days (1.57 weeks) with a posterior interval of ≤46 days (6.57 weeks), and/or dose level increase of ≥1.5 mg/kg with a posterior dose level of ≥7 mg/kg.

A=First TDM serum IFX concentration below threshold and no dose optimization within post-index period;

B=First TDM serum IFX concentration below threshold and dose optimization within post-index period;

C=First TDM serum IFX concentration at or above threshold and no dose optimization within post-index period;

D=First TDM serum IFX concentration at or above threshold and dose optimization within post-index period.

Suppl Table 4a: Subgroup Demographics and Baseline Characteristics of Patients with UC with no DO (Based on Low Threshold Criteria) Prior to their First Instance of TDM (Serum IFX Threshold: 3 μg/mL; Post-Index Period for Dose Optimization: +9 weeks; Post-Index Dose Optimization Threshold: Low)

|  | **A (N=264)** | **B (N=237)** | **C (N=471)** | **D (N=73)** | **p-value** |
| --- | --- | --- | --- | --- | --- |
| **Age (years)** | | | | | 0.0102 |
| Mean (SD) | 44.7 (16.06) | 43.2 (16.61) | 41.0 (15.51) | 40.4 (14.43) |  |
| Missing | 0 | 0 | 1 | 0 |  |
| **Age group (years)** | | | | | 0.1183 |
| 18 to 64 | 232 (87.9%) | 210 (88.6%) | 433 (92.1%) | 69 (94.5%) |  |
| 65 to 90 | 32 (12.1%) | 27 (11.4%) | 37 (7.9%) | 4 (5.5%) |  |
| Missing | 0 | 0 | 1 | 0 |  |
| **Gender** | | | | | 0.5728 |
| Female | 120 (45.5%) | 107 (45.1%) | 229 (48.6%) | 30 (41.1%) |  |
| Male | 144 (54.5%) | 130 (54.9%) | 242 (51.4%) | 43 (58.9%) |  |
| **First recorded weight** | | | | | 0.3363 |
| Mean (SD) | 75.82 (17.044) | 73.99 (16.609) | 76.05 (17.239) | 77.56 (19.613) |  |
| Missing | 1 | 0 | 0 | 0 |  |
| **Province/region of treating physician** | | | | | 0.5313 |
| Alberta | 19 (7.2%) | 16 (6.8%) | 26 (5.5%) | 3 (4.1%) |  |
| Atlantic^†^ | 41 (15.5%) | 28 (11.8%) | 70 (14.9%) | 9 (12.3%) |  |
| British Columbia | 39 (14.8%) | 19 (8.0%) | 58 (12.3%) | 6 (8.2%) |  |
| Ontario | 139 (52.7%) | 146 (61.6%) | 264 (56.1%) | 45 (61.6%) |  |
| Quebec | 24 (9.1%) | 27 (11.4%) | 52 (11.0%) | 10 (13.7%) |  |
| Saskatchewan/  Manitoba | 2 (0.8%) | 1 (0.4%) | 1 (0.2%) | 0 (0.0%) |  |
| **Year of initiation of IFX treatment** | | | | | <0.0001 |
| 2015 | 26 (9.8%) | 13 (5.5%) | 66 (14.0%) | 2 (2.7%) |  |
| 2016 | 56 (21.2%) | 29 (12.2%) | 76 (16.1%) | 6 (8.2%) |  |
| 2017 | 90 (34.1%) | 94 (39.7%) | 172 (36.5%) | 28 (38.4%) |  |
| 2018 | 92 (34.8%) | 101 (42.6%) | 157 (33.3%) | 37 (50.7%) |  |
| **Time to first TDM (days)** | | | | | <0.0001 |
| Mean (SD) | 357.0 (315.18) | 256.7 (263.69) | 340.9 (328.15) | 200.7 (230.86) |  |
| **Year of first instance of TDM** | | | | | 0.0012 |
| 2015 | 0 (0.0%) | 0 (0.0%) | 0 (0.0%) | 0 (0.0%) |  |
| 2016 | 0 (0.0%) | 0 (0.0%) | 0 (0.0%) | 0 (0.0%) |  |
| 2017 | 84 (31.8%) | 74 (31.2%) | 158 (33.5%) | 16 (21.9%) |  |
| 2018 | 115 (43.6%) | 112 (47.3%) | 246 (52.2%) | 48 (65.8%) |  |
| 2019 | 65 (24.6%) | 51 (21.5%) | 67 (14.2%) | 9 (12.3%) |  |

TDM=therapeutic drug monitoring. P-value: categorical variables, chi-square test; numeric variables, ANOVA.

^†^Atlantic includes New Brunswick, Nova Scotia, Prince Edward Island, Newfoundland and Labrador.

Dose optimization thresholds: High: A treatment interval decrease of ≥11 days (1.57 weeks) with a posterior interval of ≤35 days (5 weeks), and/or a dose level increase of ≥1.5 mg/kg with a posterior dose level of ≥9 mg/kg. Low: A treatment interval decrease of ≥11 days (1.57 weeks) with a posterior interval of ≤46 days (6.57 weeks), and/or dose level increase of ≥1.5 mg/kg with a posterior dose level of ≥7 mg/kg.

A=First TDM serum IFX concentration below threshold and no dose optimization within post-index period;

B=First TDM serum IFX concentration below threshold and dose optimization within post-index period;

C=First TDM serum IFX concentration at or above threshold and no dose optimization within post-index period;

D=First TDM serum IFX concentration at or above threshold and dose optimization within post-index period.

Suppl Table 4b: Subgroup Demographics and Baseline Characteristics of Patients with UC with no DO (Based on Low Threshold Criteria) Prior to their First Instance of TDM (Serum IFX Threshold: 3 μg/mL; Post-Index Period for Dose Optimization: +17 weeks; Post-Index Dose Optimization Threshold: Low)

|  | **A (N=223)** | **B (N=278)** | **C (N=431)** | **D (N=113)** | **p-value** |
| --- | --- | --- | --- | --- | --- |
| **Age (years)** | | | | | 0.0068 |
| Mean (SD) | 45.1 (16.22) | 43.1 (16.38) | 41.0 (15.56) | 40.4 (14.62) |  |
| Missing | 0 | 0 | 1 | 0 |  |
| **Age group (years)** | | | | | 0.0752 |
| 18 to 64 | 194 (87.0%) | 248 (89.2%) | 395 (91.9%) | 107 (94.7%) |  |
| 65 to 90 | 29 (13.0%) | 30 (10.8%) | 35 (8.1%) | 6 (5.3%) |  |
| Missing | 0 | 0 | 1 | 0 |  |
| **Gender** | | | | | 0.6951 |
| Female | 96 (43.0%) | 131 (47.1%) | 204 (47.3%) | 55 (48.7%) |  |
| Male | 127 (57.0%) | 147 (52.9%) | 227 (52.7%) | 58 (51.3%) |  |
| **First recorded weight** | | | | | 0.3830 |
| Mean (SD) | 75.96 (16.527) | 74.14 (17.085) | 76.42 (17.231) | 75.63 (18.851) |  |
| Missing | 1 | 0 | 0 | 0 |  |
| **Province/region of treating physician** | | | | | 0.1127 |
| Alberta | 15 (6.7%) | 20 (7.2%) | 20 (4.6%) | 9 (8.0%) |  |
| Atlantic^†^ | 38 (17.0%) | 31 (11.2%) | 65 (15.1%) | 14 (12.4%) |  |
| British Columbia | 36 (16.1%) | 22 (7.9%) | 54 (12.5%) | 10 (8.8%) |  |
| Ontario | 115 (51.6%) | 170 (61.2%) | 242 (56.1%) | 67 (59.3%) |  |
| Quebec | 17 (7.6%) | 34 (12.2%) | 49 (11.4%) | 13 (11.5%) |  |
| Saskatchewan/  Manitoba | 2 (0.9%) | 1 (0.4%) | 1 (0.2%) | 0 (0.0%) |  |
| **Year of initiation of IFX treatment** | | | | | <0.0001 |
| 2015 | 20 (9.0%) | 19 (6.8%) | 62 (14.4%) | 6 (5.3%) |  |
| 2016 | 50 (22.4%) | 35 (12.6%) | 74 (17.2%) | 8 (7.1%) |  |
| 2017 | 82 (36.8%) | 102 (36.7%) | 157 (36.4%) | 43 (38.1%) |  |
| 2018 | 71 (31.8%) | 122 (43.9%) | 138 (32.0%) | 56 (49.6%) |  |
| **Time to first TDM (days)** | | | | | <0.0001 |
| Mean (SD) | 363.1 (305.88) | 266.5 (280.97) | 350.9 (332.33) | 212.2 (240.44) |  |
| **Year of first instance of TDM** | | | | | 0.0032 |
| 2015 | 0 (0.0%) | 0 (0.0%) | 0 (0.0%) | 0 (0.0%) |  |
| 2016 | 0 (0.0%) | 0 (0.0%) | 0 (0.0%) | 0 (0.0%) |  |
| 2017 | 71 (31.8%) | 87 (31.3%) | 146 (33.9%) | 28 (24.8%) |  |
| 2018 | 99 (44.4%) | 128 (46.0%) | 226 (52.4%) | 68 (60.2%) |  |
| 2019 | 53 (23.8%) | 63 (22.7%) | 59 (13.7%) | 17 (15.0%) |  |

TDM=therapeutic drug monitoring. P-value: categorical variables, chi-square test; numeric variables, ANOVA.

^†^Atlantic includes New Brunswick, Nova Scotia, Prince Edward Island, Newfoundland and Labrador.

Dose optimization thresholds: High: A treatment interval decrease of ≥11 days (1.57 weeks) with a posterior interval of ≤35 days (5 weeks), and/or a dose level increase of ≥1.5 mg/kg with a posterior dose level of ≥9 mg/kg. Low: A treatment interval decrease of ≥11 days (1.57 weeks) with a posterior interval of ≤46 days (6.57 weeks), and/or dose level increase of ≥1.5 mg/kg with a posterior dose level of ≥7 mg/kg.

A=First TDM serum IFX concentration below threshold and no dose optimization within post-index period;

B=First TDM serum IFX concentration below threshold and dose optimization within post-index period;

C=First TDM serum IFX concentration at or above threshold and no dose optimization within post-index period;

D=First TDM serum IFX concentration at or above threshold and dose optimization within post-index period.

Suppl Table 4c: Subgroup Demographics and Baseline Characteristics of Patients with UC with no DO (Based on Low Threshold Criteria) Prior to their First Instance of TDM (Serum IFX Threshold: 5 μg/mL; Post-Index Period for Dose Optimization: +9 weeks; Post-Index Dose Optimization Threshold: Low)

|  | **A (N=396)** | **B (N=268)** | **C (N=339)** | **D (N=42)** | **p-value** |
| --- | --- | --- | --- | --- | --- |
| **Age (years)** | | | | | 0.0020 |
| Mean (SD) | 44.1 (16.16) | 43.2 (16.46) | 40.2 (15.13) | 38.1 (13.34) |  |
| Missing | 1 | 0 | 0 | 0 |  |
| **Age group (years)** | | | | | 0.0965 |
| 18 to 64 | 351 (88.9%) | 238 (88.8%) | 314 (92.6%) | 41 (97.6%) |  |
| 65 to 90 | 44 (11.1%) | 30 (11.2%) | 25 (7.4%) | 1 (2.4%) |  |
| Missing | 1 | 0 | 0 | 0 |  |
| **Gender** | | | | | 0.3895 |
| Female | 179 (45.2%) | 120 (44.8%) | 170 (50.1%) | 17 (40.5%) |  |
| Male | 217 (54.8%) | 148 (55.2%) | 169 (49.9%) | 25 (59.5%) |  |
| **First recorded weight** | | | | | 0.6910 |
| Mean (SD) | 75.56 (17.570) | 74.76 (16.999) | 76.44 (16.680) | 75.28 (19.965) |  |
| Missing | 1 | 0 | 0 | 0 |  |
| **Province/region of treating physician** | | | | | 0.6047 |
| Alberta | 26 (6.6%) | 16 (6.0%) | 19 (5.6%) | 3 (7.1%) |  |
| Atlantic^†^ | 56 (14.1%) | 32 (11.9%) | 55 (16.2%) | 5 (11.9%) |  |
| British Columbia | 57 (14.4%) | 24 (9.0%) | 40 (11.8%) | 1 (2.4%) |  |
| Ontario | 213 (53.8%) | 164 (61.2%) | 190 (56.0%) | 27 (64.3%) |  |
| Quebec | 42 (10.6%) | 31 (11.6%) | 34 (10.0%) | 6 (14.3%) |  |
| Saskatchewan/  Manitoba | 2 (0.5%) | 1 (0.4%) | 1 (0.3%) | 0 (0.0%) |  |
| **Year of initiation of IFX treatment** | | | | | 0.0006 |
| 2015 | 49 (12.4%) | 14 (5.2%) | 43 (12.7%) | 1 (2.4%) |  |
| 2016 | 75 (18.9%) | 32 (11.9%) | 57 (16.8%) | 3 (7.1%) |  |
| 2017 | 135 (34.1%) | 102 (38.1%) | 127 (37.5%) | 20 (47.6%) |  |
| 2018 | 137 (34.6%) | 120 (44.8%) | 112 (33.0%) | 18 (42.9%) |  |
| **Time to first TDM (days)** | | | | | <0.0001 |
| Mean (SD) | 364.0 (323.46) | 256.7 (268.75) | 326.4 (322.69) | 159.0 (138.85) |  |
| **Year of first instance of TDM** | | | | | 0.0140 |
| 2015 | 0 (0.0%) | 0 (0.0%) | 0 (0.0%) | 0 (0.0%) |  |
| 2016 | 0 (0.0%) | 0 (0.0%) | 0 (0.0%) | 0 (0.0%) |  |
| 2017 | 127 (32.1%) | 77 (28.7%) | 115 (33.9%) | 13 (31.0%) |  |
| 2018 | 184 (46.5%) | 133 (49.6%) | 177 (52.2%) | 27 (64.3%) |  |
| 2019 | 85 (21.5%) | 58 (21.6%) | 47 (13.9%) | 2 (4.8%) |  |

TDM=therapeutic drug monitoring. P-value: categorical variables, chi-square test; numeric variables, ANOVA.

^†^Atlantic includes New Brunswick, Nova Scotia, Prince Edward Island, Newfoundland and Labrador.

Dose optimization thresholds: High: A treatment interval decrease of ≥11 days (1.57 weeks) with a posterior interval of ≤35 days (5 weeks), and/or a dose level increase of ≥1.5 mg/kg with a posterior dose level of ≥9 mg/kg. Low: A treatment interval decrease of ≥11 days (1.57 weeks) with a posterior interval of ≤46 days (6.57 weeks), and/or dose level increase of ≥1.5 mg/kg with a posterior dose level of ≥7 mg/kg.

A=First TDM serum IFX concentration below threshold and no dose optimization within post-index period;

B=First TDM serum IFX concentration below threshold and dose optimization within post-index period;

C=First TDM serum IFX concentration at or above threshold and no dose optimization within post-index period;

D=First TDM serum IFX concentration at or above threshold and dose optimization within post-index period.

Suppl Table 4d: Subgroup Demographics and Baseline Characteristics of Patients with UC with no DO (Based on Low Threshold Criteria) Prior to their First Instance of TDM (Serum IFX Threshold: 5 μg/mL; Post-Index Period for Dose Optimization: +17 weeks; Post-Index Dose Optimization Threshold: Low)

|  | **A (N=334)** | **B (N=330)** | **C (N=320)** | **D (N=61)** | **p-value** |
| --- | --- | --- | --- | --- | --- |
| **Age (years)** | | | | | 0.0013 |
| Mean (SD) | 44.6 (16.33) | 42.9 (16.20) | 40.2 (15.13) | 39.1 (13.98) |  |
| Missing | 1 | 0 | 0 | 0 |  |
| **Age group (years)** | | | | | 0.0762 |
| 18 to 64 | 293 (88.0%) | 296 (89.7%) | 296 (92.5%) | 59 (96.7%) |  |
| 65 to 90 | 40 (12.0%) | 34 (10.3%) | 24 (7.5%) | 2 (3.3%) |  |
| Missing | 1 | 0 | 0 | 0 |  |
| **Gender** | | | | | 0.4511 |
| Female | 145 (43.4%) | 154 (46.7%) | 155 (48.4%) | 32 (52.5%) |  |
| Male | 189 (56.6%) | 176 (53.3%) | 165 (51.6%) | 29 (47.5%) |  |
| **First recorded weight** | | | | | 0.3344 |
| Mean (SD) | 75.69 (17.565) | 74.78 (17.110) | 76.86 (16.362) | 73.47 (20.181) |  |
| Missing | 1 | 0 | 0 | 0 |  |
| **Province/region of treating physician** | | | | | 0.0674 |
| Alberta | 21 (6.3%) | 21 (6.4%) | 14 (4.4%) | 8 (13.1%) |  |
| Atlantic^†^ | 48 (14.4%) | 40 (12.1%) | 55 (17.2%) | 5 (8.2%) |  |
| British Columbia | 53 (15.9%) | 28 (8.5%) | 37 (11.6%) | 4 (6.6%) |  |
| Ontario | 177 (53.0%) | 200 (60.6%) | 180 (56.3%) | 37 (60.7%) |  |
| Quebec | 33 (9.9%) | 40 (12.1%) | 33 (10.3%) | 7 (11.5%) |  |
| Saskatchewan/  Manitoba | 2 (0.6%) | 1 (0.3%) | 1 (0.3%) | 0 (0.0%) |  |
| **Year of initiation of IFX treatment** | | | | | <0.0001 |
| 2015 | 41 (12.3%) | 22 (6.7%) | 41 (12.8%) | 3 (4.9%) |  |
| 2016 | 68 (20.4%) | 39 (11.8%) | 56 (17.5%) | 4 (6.6%) |  |
| 2017 | 120 (35.9%) | 117 (35.5%) | 119 (37.2%) | 28 (45.9%) |  |
| 2018 | 105 (31.4%) | 152 (46.1%) | 104 (32.5%) | 26 (42.6%) |  |
| **Time to first TDM (days)** | | | | | <0.0001 |
| Mean (SD) | 379.1 (322.68) | 261.5 (278.30) | 329.9 (322.68) | 192.9 (218.16) |  |
| **Year of first instance of TDM** | | | | | 0.0368 |
| 2015 | 0 (0.0%) | 0 (0.0%) | 0 (0.0%) | 0 (0.0%) |  |
| 2016 | 0 (0.0%) | 0 (0.0%) | 0 (0.0%) | 0 (0.0%) |  |
| 2017 | 108 (32.3%) | 96 (29.1%) | 109 (34.1%) | 19 (31.1%) |  |
| 2018 | 156 (46.7%) | 161 (48.8%) | 169 (52.8%) | 35 (57.4%) |  |
| 2019 | 70 (21.0%) | 73 (22.1%) | 42 (13.1%) | 7 (11.5%) |  |

TDM=therapeutic drug monitoring. P-value: categorical variables, chi-square test; numeric variables, ANOVA.

^†^Atlantic includes New Brunswick, Nova Scotia, Prince Edward Island, Newfoundland and Labrador.

Dose optimization thresholds: High: A treatment interval decrease of ≥11 days (1.57 weeks) with a posterior interval of ≤35 days (5 weeks), and/or a dose level increase of ≥1.5 mg/kg with a posterior dose level of ≥9 mg/kg. Low: A treatment interval decrease of ≥11 days (1.57 weeks) with a posterior interval of ≤46 days (6.57 weeks), and/or dose level increase of ≥1.5 mg/kg with a posterior dose level of ≥7 mg/kg.

A=First TDM serum IFX concentration below threshold and no dose optimization within post-index period;

B=First TDM serum IFX concentration below threshold and dose optimization within post-index period;

C=First TDM serum IFX concentration at or above threshold and no dose optimization within post-index period;

D=First TDM serum IFX concentration at or above threshold and dose optimization within post-index period.

Suppl Table 4e: Subgroup Demographics and Baseline Characteristics of Patients with UC with no DO (Based on Low Threshold Criteria) Prior to their First Instance of TDM (Serum IFX Threshold: 10 μg/mL; Post-Index Period for Dose Optimization: +9 weeks; Post-Index Dose Optimization Threshold: Low)

|  | **A (N=605)** | **B (N=300)** | **C (N=130)** | **D (N=10)** | **p-value** |
| --- | --- | --- | --- | --- | --- |
| **Age (years)** | | | | | 0.1256 |
| Mean (SD) | 42.9 (16.18) | 42.7 (16.33) | 39.6 (13.65) | 37.6 (8.10) |  |
| Missing | 1 | 0 | 0 | 0 |  |
| **Age group (years)** | | | | | 0.1410 |
| 18 to 64 | 541 (89.6%) | 269 (89.7%) | 124 (95.4%) | 10 (100.0%) |  |
| 65 to 90 | 63 (10.4%) | 31 (10.3%) | 6 (4.6%) | 0 (0.0%) |  |
| Missing | 1 | 0 | 0 | 0 |  |
| **Gender** | | | | | 0.0874 |
| Female | 276 (45.6%) | 134 (44.7%) | 73 (56.2%) | 3 (30.0%) |  |
| Male | 329 (54.4%) | 166 (55.3%) | 57 (43.8%) | 7 (70.0%) |  |
| **First recorded weight** | | | | | 0.3039 |
| Mean (SD) | 75.80 (16.910) | 75.10 (17.413) | 76.74 (18.316) | 66.59 (15.450) |  |
| Missing | 1 | 0 | 0 | 0 |  |
| **Province/region of treating physician** | | | | | 0.2989 |
| Alberta | 40 (6.6%) | 19 (6.3%) | 5 (3.8%) | 0 (0.0%) |  |
| Atlantic^†^ | 93 (15.4%) | 34 (11.3%) | 18 (13.8%) | 3 (30.0%) |  |
| British Columbia | 81 (13.4%) | 25 (8.3%) | 16 (12.3%) | 0 (0.0%) |  |
| Ontario | 323 (53.4%) | 186 (62.0%) | 80 (61.5%) | 5 (50.0%) |  |
| Quebec | 65 (10.7%) | 35 (11.7%) | 11 (8.5%) | 2 (20.0%) |  |
| Saskatchewan/  Manitoba | 3 (0.5%) | 1 (0.3%) | 0 (0.0%) | 0 (0.0%) |  |
| **Year of initiation of IFX treatment** | | | | | 0.0004 |
| 2015 | 77 (12.7%) | 14 (4.7%) | 15 (11.5%) | 1 (10.0%) |  |
| 2016 | 115 (19.0%) | 34 (11.3%) | 17 (13.1%) | 1 (10.0%) |  |
| 2017 | 208 (34.4%) | 118 (39.3%) | 54 (41.5%) | 4 (40.0%) |  |
| 2018 | 205 (33.9%) | 134 (44.7%) | 44 (33.8%) | 4 (40.0%) |  |
| **Time to first TDM (days)** | | | | | <0.0001 |
| Mean (SD) | 357.3 (329.67) | 244.1 (258.24) | 296.9 (288.60) | 225.5 (230.65) |  |
| **Year of first instance of TDM** | | | | | 0.1283 |
| 2015 | 0 (0.0%) | 0 (0.0%) | 0 (0.0%) | 0 (0.0%) |  |
| 2016 | 0 (0.0%) | 0 (0.0%) | 0 (0.0%) | 0 (0.0%) |  |
| 2017 | 194 (32.1%) | 87 (29.0%) | 48 (36.9%) | 3 (30.0%) |  |
| 2018 | 293 (48.4%) | 153 (51.0%) | 68 (52.3%) | 7 (70.0%) |  |
| 2019 | 118 (19.5%) | 60 (20.0%) | 14 (10.8%) | 0 (0.0%) |  |

TDM=therapeutic drug monitoring. P-value: categorical variables, chi-square test; numeric variables, ANOVA.

^†^Atlantic includes New Brunswick, Nova Scotia, Prince Edward Island, Newfoundland and Labrador.

Dose optimization thresholds: High: A treatment interval decrease of ≥11 days (1.57 weeks) with a posterior interval of ≤35 days (5 weeks), and/or a dose level increase of ≥1.5 mg/kg with a posterior dose level of ≥9 mg/kg. Low: A treatment interval decrease of ≥11 days (1.57 weeks) with a posterior interval of ≤46 days (6.57 weeks), and/or dose level increase of ≥1.5 mg/kg with a posterior dose level of ≥7 mg/kg.

A=First TDM serum IFX concentration below threshold and no dose optimization within post-index period;

B=First TDM serum IFX concentration below threshold and dose optimization within post-index period;

C=First TDM serum IFX concentration at or above threshold and no dose optimization within post-index period;

D=First TDM serum IFX concentration at or above threshold and dose optimization within post-index period.

Suppl Table 4f: Subgroup Demographics and Baseline Characteristics of Patients with UC with no DO (Based on Low Threshold Criteria) Prior to their First Instance of TDM (Serum IFX Threshold: 10 μg/mL; Post-Index Period for Dose Optimization: +17 weeks; Post-Index Dose Optimization Threshold: Low)

|  | **A (N=529)** | **B (N=376)** | **C (N=125)** | **D (N=15)** | **p-value** |
| --- | --- | --- | --- | --- | --- |
| **Age (years)** | | | | | 0.0966 |
| Mean (SD) | 43.2 (16.33) | 42.4 (16.07) | 39.2 (13.52) | 41.2 (11.80) |  |
| Missing | 1 | 0 | 0 | 0 |  |
| **Age group (years)** | | | | | 0.1073 |
| 18 to 64 | 470 (89.0%) | 340 (90.4%) | 119 (95.2%) | 15 (100.0%) |  |
| 65 to 90 | 58 (11.0%) | 36 (9.6%) | 6 (4.8%) | 0 (0.0%) |  |
| Missing | 1 | 0 | 0 | 0 |  |
| **Gender** | | | | | 0.1275 |
| Female | 231 (43.7%) | 179 (47.6%) | 69 (55.2%) | 7 (46.7%) |  |
| Male | 298 (56.3%) | 197 (52.4%) | 56 (44.8%) | 8 (53.3%) |  |
| **First recorded weight** | | | | | 0.1276 |
| Mean (SD) | 76.07 (16.698) | 74.87 (17.583) | 77.10 (18.187) | 67.05 (16.936) |  |
| Missing | 1 | 0 | 0 | 0 |  |
| **Province/region of treating physician** | | | | | 0.1467 |
| Alberta | 30 (5.7%) | 29 (7.7%) | 5 (4.0%) | 0 (0.0%) |  |
| Atlantic^†^ | 85 (16.1%) | 42 (11.2%) | 18 (14.4%) | 3 (20.0%) |  |
| British Columbia | 76 (14.4%) | 30 (8.0%) | 14 (11.2%) | 2 (13.3%) |  |
| Ontario | 280 (52.9%) | 229 (60.9%) | 77 (61.6%) | 8 (53.3%) |  |
| Quebec | 55 (10.4%) | 45 (12.0%) | 11 (8.8%) | 2 (13.3%) |  |
| Saskatchewan/  Manitoba | 3 (0.6%) | 1 (0.3%) | 0 (0.0%) | 0 (0.0%) |  |
| **Year of initiation of IFX treatment** | | | | | <0.0001 |
| 2015 | 68 (12.9%) | 23 (6.1%) | 14 (11.2%) | 2 (13.3%) |  |
| 2016 | 107 (20.2%) | 42 (11.2%) | 17 (13.6%) | 1 (6.7%) |  |
| 2017 | 188 (35.5%) | 138 (36.7%) | 51 (40.8%) | 7 (46.7%) |  |
| 2018 | 166 (31.4%) | 173 (46.0%) | 43 (34.4%) | 5 (33.3%) |  |
| **Time to first TDM (days)** | | | | | <0.0001 |
| Mean (SD) | 369.9 (331.06) | 249.3 (268.76) | 292.1 (281.16) | 289.7 (323.88) |  |
| **Year of first instance of TDM** | | | | | 0.1563 |
| 2015 | 0 (0.0%) | 0 (0.0%) | 0 (0.0%) | 0 (0.0%) |  |
| 2016 | 0 (0.0%) | 0 (0.0%) | 0 (0.0%) | 0 (0.0%) |  |
| 2017 | 171 (32.3%) | 110 (29.3%) | 46 (36.8%) | 5 (33.3%) |  |
| 2018 | 259 (49.0%) | 187 (49.7%) | 66 (52.8%) | 9 (60.0%) |  |
| 2019 | 99 (18.7%) | 79 (21.0%) | 13 (10.4%) | 1 (6.7%) |  |

TDM=therapeutic drug monitoring. P-value: categorical variables, chi-square test; numeric variables, ANOVA.

^†^Atlantic includes New Brunswick, Nova Scotia, Prince Edward Island, Newfoundland and Labrador.

Dose optimization thresholds: High: A treatment interval decrease of ≥11 days (1.57 weeks) with a posterior interval of ≤35 days (5 weeks), and/or a dose level increase of ≥1.5 mg/kg with a posterior dose level of ≥9 mg/kg. Low: A treatment interval decrease of ≥11 days (1.57 weeks) with a posterior interval of ≤46 days (6.57 weeks), and/or dose level increase of ≥1.5 mg/kg with a posterior dose level of ≥7 mg/kg.

A=First TDM serum IFX concentration below threshold and no dose optimization within post-index period;

B=First TDM serum IFX concentration below threshold and dose optimization within post-index period;

C=First TDM serum IFX concentration at or above threshold and no dose optimization within post-index period;

D=First TDM serum IFX concentration at or above threshold and dose optimization within post-index period.

Suppl Table 4g: Subgroup Demographics and Baseline Characteristics of Patients with UC with no DO (Based on Low Threshold Criteria) Prior to their First Instance of TDM (Serum IFX Threshold: 3 μg/mL; Post-Index Period for Dose Optimization: +9 weeks; Post-Index Dose Optimization Threshold: High)

|  | **A (N=341)** | **B (N=160)** | **C (N=504)** | **D (N=40)** | **p-value** |
| --- | --- | --- | --- | --- | --- |
| **Age (years)** | | | | | 0.0144 |
| Mean (SD) | 44.4 (16.26) | 43.3 (16.49) | 40.9 (15.41) | 41.2 (14.97) |  |
| Missing | 0 | 0 | 1 | 0 |  |
| **Age group (years)** | | | | | 0.1089 |
| 18 to 64 | 299 (87.7%) | 143 (89.4%) | 464 (92.2%) | 38 (95.0%) |  |
| 65 to 90 | 42 (12.3%) | 17 (10.6%) | 39 (7.8%) | 2 (5.0%) |  |
| Missing | 0 | 0 | 1 | 0 |  |
| **Gender** | | | | | 0.8600 |
| Female | 156 (45.7%) | 71 (44.4%) | 241 (47.8%) | 18 (45.0%) |  |
| Male | 185 (54.3%) | 89 (55.6%) | 263 (52.2%) | 22 (55.0%) |  |
| **First recorded weight** | | | | | 0.6102 |
| Mean (SD) | 75.08 (17.240) | 74.67 (16.029) | 76.15 (17.411) | 77.61 (19.588) |  |
| Missing | 1 | 0 | 0 | 0 |  |
| **Province/region of treating physician** | | | | | 0.0128 |
| Alberta | 20 (5.9%) | 15 (9.4%) | 29 (5.8%) | 0 (0.0%) |  |
| Atlantic^†^ | 52 (15.2%) | 17 (10.6%) | 78 (15.5%) | 1 (2.5%) |  |
| British Columbia | 51 (15.0%) | 7 (4.4%) | 59 (11.7%) | 5 (12.5%) |  |
| Ontario | 181 (53.1%) | 104 (65.0%) | 283 (56.2%) | 26 (65.0%) |  |
| Quebec | 35 (10.3%) | 16 (10.0%) | 54 (10.7%) | 8 (20.0%) |  |
| Saskatchewan/  Manitoba | 2 (0.6%) | 1 (0.6%) | 1 (0.2%) | 0 (0.0%) |  |
| **Year of initiation of IFX treatment** | | | | | 0.0002 |
| 2015 | 33 (9.7%) | 6 (3.8%) | 67 (13.3%) | 1 (2.5%) |  |
| 2016 | 63 (18.5%) | 22 (13.8%) | 80 (15.9%) | 2 (5.0%) |  |
| 2017 | 122 (35.8%) | 62 (38.8%) | 189 (37.5%) | 11 (27.5%) |  |
| 2018 | 123 (36.1%) | 70 (43.8%) | 168 (33.3%) | 26 (65.0%) |  |
| **Time to first TDM (days)** | | | | | 0.0006 |
| Mean (SD) | 339.7 (309.49) | 245.2 (253.98) | 331.6 (323.38) | 201.4 (251.12) |  |
| **Year of first instance of TDM** | | | | | 0.0003 |
| 2015 | 0 (0.0%) | 0 (0.0%) | 0 (0.0%) | 0 (0.0%) |  |
| 2016 | 0 (0.0%) | 0 (0.0%) | 0 (0.0%) | 0 (0.0%) |  |
| 2017 | 109 (32.0%) | 49 (30.6%) | 169 (33.5%) | 5 (12.5%) |  |
| 2018 | 151 (44.3%) | 76 (47.5%) | 264 (52.4%) | 30 (75.0%) |  |
| 2019 | 81 (23.8%) | 35 (21.9%) | 71 (14.1%) | 5 (12.5%) |  |

TDM=therapeutic drug monitoring. P-value: categorical variables, chi-square test; numeric variables, ANOVA.

^†^Atlantic includes New Brunswick, Nova Scotia, Prince Edward Island, Newfoundland, and Labrador.

Dose optimization thresholds: High: A treatment interval decrease of ≥11 days (1.57 weeks) with a posterior interval of ≤35 days (5 weeks), and/or a dose level increase of ≥1.5 mg/kg with a posterior dose level of ≥9 mg/kg. Low: A treatment interval decrease of ≥11 days (1.57 weeks) with a posterior interval of ≤46 days (6.57 weeks), and/or dose level increase of ≥1.5 mg/kg with a posterior dose level of ≥7 mg/kg.

A=First TDM serum IFX concentration below threshold and no dose optimization within post-index period;

B=First TDM serum IFX concentration below threshold and dose optimization within post-index period;

C=First TDM serum IFX concentration at or above threshold and no dose optimization within post-index period;

D=First TDM serum IFX concentration at or above threshold and dose optimization within post-index period.

Suppl Table 4h: Subgroup Demographics and Baseline Characteristics of Patients with UC with no DO (Based on Low Threshold Criteria) Prior to their First Instance of TDM (Serum IFX Threshold: 3 μg/mL; Post-Index Period for Dose Optimization: +17 weeks; Post-Index Dose Optimization Threshold: High)

|  | **A (N=313)** | **B (N=188)** | **C (N=476)** | **D (N=68)** | **p-value** |
| --- | --- | --- | --- | --- | --- |
| **Age (years)** | | | | | 0.0134 |
| Mean (SD) | 44.4 (16.41) | 43.3 (16.20) | 40.8 (15.46) | 41.5 (14.76) |  |
| Missing | 0 | 0 | 1 | 0 |  |
| **Age group (years)** | | | | | 0.0861 |
| 18 to 64 | 273 (87.2%) | 169 (89.9%) | 438 (92.2%) | 64 (94.1%) |  |
| 65 to 90 | 40 (12.8%) | 19 (10.1%) | 37 (7.8%) | 4 (5.9%) |  |
| Missing | 0 | 0 | 1 | 0 |  |
| **Gender** | | | | | 0.9004 |
| Female | 142 (45.4%) | 85 (45.2%) | 226 (47.5%) | 33 (48.5%) |  |
| Male | 171 (54.6%) | 103 (54.8%) | 250 (52.5%) | 35 (51.5%) |  |
| **First recorded weight** | | | | | 0.6479 |
| Mean (SD) | 74.95 (17.166) | 74.95 (16.349) | 76.37 (17.520) | 75.48 (17.986) |  |
| Missing | 1 | 0 | 0 | 0 |  |
| **Province/region of treating physician** | | | | | 0.0813 |
| Alberta | 19 (6.1%) | 16 (8.5%) | 25 (5.3%) | 4 (5.9%) |  |
| Atlantic^†^ | 51 (16.3%) | 18 (9.6%) | 74 (15.5%) | 5 (7.4%) |  |
| British Columbia | 47 (15.0%) | 11 (5.9%) | 56 (11.8%) | 8 (11.8%) |  |
| Ontario | 163 (52.1%) | 122 (64.9%) | 266 (55.9%) | 43 (63.2%) |  |
| Quebec | 31 (9.9%) | 20 (10.6%) | 54 (11.3%) | 8 (11.8%) |  |
| Saskatchewan/  Manitoba | 2 (0.6%) | 1 (0.5%) | 1 (0.2%) | 0 (0.0%) |  |
| **Year of initiation of IFX treatment** | | | | | <0.0001 |
| 2015 | 28 (8.9%) | 11 (5.9%) | 67 (14.1%) | 1 (1.5%) |  |
| 2016 | 59 (18.8%) | 26 (13.8%) | 79 (16.6%) | 3 (4.4%) |  |
| 2017 | 117 (37.4%) | 67 (35.6%) | 177 (37.2%) | 23 (33.8%) |  |
| 2018 | 109 (34.8%) | 84 (44.7%) | 153 (32.1%) | 41 (60.3%) |  |
| **Time to first TDM (days)** | | | | | <0.0001 |
| Mean (SD) | 343.8 (310.98) | 252.5 (260.01) | 342.8 (328.63) | 176.9 (202.67) |  |
| **Year of first instance of TDM** | | | | | 0.0006 |
| 2015 | 0 (0.0%) | 0 (0.0%) | 0 (0.0%) | 0 (0.0%) |  |
| 2016 | 0 (0.0%) | 0 (0.0%) | 0 (0.0%) | 0 (0.0%) |  |
| 2017 | 99 (31.6%) | 59 (31.4%) | 162 (34.0%) | 12 (17.6%) |  |
| 2018 | 140 (44.7%) | 87 (46.3%) | 249 (52.3%) | 45 (66.2%) |  |
| 2019 | 74 (23.6%) | 42 (22.3%) | 65 (13.7%) | 11 (16.2%) |  |

TDM=therapeutic drug monitoring. P-value: categorical variables, chi-square test; numeric variables, ANOVA.

^†^Atlantic includes New Brunswick, Nova Scotia, Prince Edward Island, Newfoundland, and Labrador.

Dose optimization thresholds: High: A treatment interval decrease of ≥11 days (1.57 weeks) with a posterior interval of ≤35 days (5 weeks), and/or a dose level increase of ≥1.5 mg/kg with a posterior dose level of ≥9 mg/kg. Low: A treatment interval decrease of ≥11 days (1.57 weeks) with a posterior interval of ≤46 days (6.57 weeks), and/or dose level increase of ≥1.5 mg/kg with a posterior dose level of ≥7 mg/kg.

A=First TDM serum IFX concentration below threshold and no dose optimization within post-index period;

B=First TDM serum IFX concentration below threshold and dose optimization within post-index period;

C=First TDM serum IFX concentration at or above threshold and no dose optimization within post-index period;

D=First TDM serum IFX concentration at or above threshold and dose optimization within post-index period.

Suppl Table 4i: Subgroup Demographics and Baseline Characteristics of Patients with UC with no DO (Based on Low Threshold Criteria) Prior to their First Instance of TDM (Serum IFX Threshold: 5 μg/mL; Post-Index Period for Dose Optimization: +9 weeks; Post-Index Dose Optimization Threshold: High)

|  | **A (N=485)** | **B (N=179)** | **C (N=360)** | **D (N=21)** | **p-value** |
| --- | --- | --- | --- | --- | --- |
| **Age (years)** | | | | | 0.0024 |
| Mean (SD) | 43.9 (16.27) | 43.5 (16.35) | 40.2 (15.01) | 37.0 (13.61) |  |
| Missing | 1 | 0 | 0 | 0 |  |
| **Age group (years)** | | | | | 0.0883 |
| 18 to 64 | 429 (88.6%) | 160 (89.4%) | 334 (92.8%) | 21 (100.0%) |  |
| 65 to 90 | 55 (11.4%) | 19 (10.6%) | 26 (7.2%) | 0 (0.0%) |  |
| Missing | 1 | 0 | 0 | 0 |  |
| **Gender** | | | | | 0.5819 |
| Female | 219 (45.2%) | 80 (44.7%) | 178 (49.4%) | 9 (42.9%) |  |
| Male | 266 (54.8%) | 99 (55.3%) | 182 (50.6%) | 12 (57.1%) |  |
| **First recorded weight** | | | | | 0.7639 |
| Mean (SD) | 75.20 (17.646) | 75.32 (16.503) | 76.41 (16.919) | 74.67 (19.498) |  |
| Missing | 1 | 0 | 0 | 0 |  |
| **Province/region of treating physician** | | | | | 0.0885 |
| Alberta | 27 (5.6%) | 15 (8.4%) | 22 (6.1%) | 0 (0.0%) |  |
| Atlantic^†^ | 71 (14.6%) | 17 (9.5%) | 59 (16.4%) | 1 (4.8%) |  |
| British Columbia | 69 (14.2%) | 12 (6.7%) | 41 (11.4%) | 0 (0.0%) |  |
| Ontario | 263 (54.2%) | 114 (63.7%) | 201 (55.8%) | 16 (76.2%) |  |
| Quebec | 53 (10.9%) | 20 (11.2%) | 36 (10.0%) | 4 (19.0%) |  |
| Saskatchewan/  Manitoba | 2 (0.4%) | 1 (0.6%) | 1 (0.3%) | 0 |  |
| **Year of initiation of IFX treatment** | | | | | 0.0018 |
| 2015 | 57 (11.8%) | 6 (3.4%) | 43 (11.9%) | 1 (4.8%) |  |
| 2016 | 83 (17.1%) | 24 (13.4%) | 60 (16.7%) | 0 (0.0%) |  |
| 2017 | 171 (35.3%) | 66 (36.9%) | 140 (38.9%) | 7 (33.3%) |  |
| 2018 | 174 (35.9%) | 83 (46.4%) | 117 (32.5%) | 13 (61.9%) |  |
| **Time to first TDM (days)** | | | | | 0.0001 |
| Mean (SD) | 347.5 (318.53) | 248.0 (260.20) | 317.9 (316.20) | 137.8 (157.61) |  |
| **Year of first instance of TDM** | | | | | 0.0053 |
| 2015 | 0 (0.0%) | 0 (0.0%) | 0 (0.0%) | 0 (0.0%) |  |
| 2016 | 0 (0.0%) | 0 (0.0%) | 0 (0.0%) | 0 (0.0%) |  |
| 2017 | 155 (32.0%) | 49 (27.4%) | 123 (34.2%) | 5 (23.8%) |  |
| 2018 | 227 (46.8%) | 90 (50.3%) | 188 (52.2%) | 16 (76.2%) |  |
| 2019 | 103 (21.2%) | 40 (22.3%) | 49 (13.6%) | 0 (0.0%) |  |

TDM=therapeutic drug monitoring. P-value: categorical variables, chi-square test; numeric variables, ANOVA.

^†^Atlantic includes New Brunswick, Nova Scotia, Prince Edward Island, Newfoundland, and Labrador.

Dose optimization thresholds: High: A treatment interval decrease of ≥11 days (1.57 weeks) with a posterior interval of ≤35 days (5 weeks), and/or a dose level increase of ≥1.5 mg/kg with a posterior dose level of ≥9 mg/kg. Low: A treatment interval decrease of ≥11 days (1.57 weeks) with a posterior interval of ≤46 days (6.57 weeks), and/or dose level increase of ≥1.5 mg/kg with a posterior dose level of ≥7 mg/kg.

A=First TDM serum IFX concentration below threshold and no dose optimization within post-index period;

B=First TDM serum IFX concentration below threshold and dose optimization within post-index period;

C=First TDM serum IFX concentration at or above threshold and no dose optimization within post-index period;

D=First TDM serum IFX concentration at or above threshold and dose optimization within post-index period.

Suppl Table 4j: Subgroup Demographics and Baseline Characteristics of Patients with UC with no DO (Based on Low Threshold Criteria) Prior to their First Instance of TDM (Serum IFX Threshold: 5 μg/mL; Post-Index Period for Dose Optimization: +17 weeks; Post-Index Dose Optimization Threshold: High)

|  | **A (N=442)** | **B (N=222)** | **C (N=347)** | **D (N=34)** | **p-value** |
| --- | --- | --- | --- | --- | --- |
| **Age (years)** | | | | | 0.0031 |
| Mean (SD) | 44.0 (16.39) | 43.4 (16.09) | 40.1 (15.08) | 39.3 (13.63) |  |
| Missing | 1 | 0 | 0 | 0 |  |
| **Age group (years)** | | | | | 0.0893 |
| 18 to 64 | 389 (88.2%) | 200 (90.1%) | 322 (92.8%) | 33 (97.1%) |  |
| 65 to 90 | 52 (11.8%) | 22 (9.9%) | 25 (7.2%) | 1 (2.9%) |  |
| Missing | 1 | 0 | 0 | 0 |  |
| **Gender** | | | | | 0.6506 |
| Female | 198 (44.8%) | 101 (45.5%) | 170 (49.0%) | 17 (50.0%) |  |
| Male | 244 (55.2%) | 121 (54.5%) | 177 (51.0%) | 17 (50.0%) |  |
| **First recorded weight** | | | | | 0.5546 |
| Mean (SD) | 75.17 (17.714) | 75.36 (16.589) | 76.61 (16.947) | 73.33 (18.031) |  |
| Missing | 1 | 0 | 0 | 0 |  |
| **Province/region of treating physician** | | | | | 0.1126 |
| Alberta | 26 (5.9%) | 16 (7.2%) | 18 (5.2%) | 4 (11.8%) |  |
| Atlantic^†^ | 67 (15.2%) | 21 (9.5%) | 58 (16.7%) | 2 (5.9%) |  |
| British Columbia | 65 (14.7%) | 16 (7.2%) | 38 (11.0%) | 3 (8.8%) |  |
| Ontario | 233 (52.7%) | 144 (64.9%) | 196 (56.5%) | 21 (61.8%) |  |
| Quebec | 49 (11.1%) | 24 (10.8%) | 36 (10.4%) | 4 (11.8%) |  |
| Saskatchewan/  Manitoba | 2 (0.5%) | 1 (0.5%) | 1 (0.3%) | 0 (0.0%) |  |
| **Year of initiation of IFX treatment** | | | | | 0.0002 |
| 2015 | 52 (11.8%) | 11 (5.0%) | 43 (12.4%) | 1 (2.9%) |  |
| 2016 | 78 (17.6%) | 29 (13.1%) | 60 (17.3%) | 0 (0.0%) |  |
| 2017 | 161 (36.4%) | 76 (34.2%) | 133 (38.3%) | 14 (41.2%) |  |
| 2018 | 151 (34.2%) | 106 (47.7%) | 111 (32.0%) | 19 (55.9%) |  |
| **Time to first TDM (days)** | | | | | <0.0001 |
| Mean (SD) | 357.1 (322.76) | 248.1 (258.35) | 325.4 (319.56) | 130.1 (125.33) |  |
| **Year of first instance of TDM** | | | | | 0.0215 |
| 2015 | 0 (0.0%) | 0 (0.0%) | 0 (0.0%) | 0 (0.0%) |  |
| 2016 | 0 (0.0%) | 0 (0.0%) | 0 (0.0%) | 0 (0.0%) |  |
| 2017 | 143 (32.4%) | 61 (27.5%) | 118 (34.0%) | 10 (29.4%) |  |
| 2018 | 206 (46.6%) | 111 (50.0%) | 183 (52.7%) | 21 (61.8%) |  |
| 2019 | 93 (21.0%) | 50 (22.5%) | 46 (13.3%) | 3 (8.8%) |  |

TDM=therapeutic drug monitoring. P-value: categorical variables, chi-square test; numeric variables, ANOVA.

^†^Atlantic includes New Brunswick, Nova Scotia, Prince Edward Island, Newfoundland, and Labrador.

Dose optimization thresholds: High: A treatment interval decrease of ≥11 days (1.57 weeks) with a posterior interval of ≤35 days (5 weeks), and/or a dose level increase of ≥1.5 mg/kg with a posterior dose level of ≥9 mg/kg. Low: A treatment interval decrease of ≥11 days (1.57 weeks) with a posterior interval of ≤46 days (6.57 weeks), and/or dose level increase of ≥1.5 mg/kg with a posterior dose level of ≥7 mg/kg.

A=First TDM serum IFX concentration below threshold and no dose optimization within post-index period;

B=First TDM serum IFX concentration below threshold and dose optimization within post-index period;

C=First TDM serum IFX concentration at or above threshold and no dose optimization within post-index period;

D=First TDM serum IFX concentration at or above threshold and dose optimization within post-index period.

Suppl Table 4k: Subgroup Demographics and Baseline Characteristics of Patients with UC with no DO (Based on Low Threshold Criteria) Prior to their First Instance of TDM (Serum IFX Threshold: 10 μg/mL; Post-Index Period for Dose Optimization: +9 weeks; Post-Index Dose Optimization Threshold: High)

|  | **A (N=710)** | **B (N=195)** | **C (N=135)** | **D (N=5)** | **p-value** |
| --- | --- | --- | --- | --- | --- |
| **Age (years)** | | | | | 0.1356 |
| Mean (SD) | 42.8 (16.20) | 42.9 (16.35) | 39.4 (13.50) | 40.2 (7.56) |  |
| Missing | 1 | 0 | 0 | 0 |  |
| **Age group (years)** | | | | | 0.1409 |
| 18 to 64 | 634 (89.4%) | 176 (90.3%) | 129 (95.6%) | 5 (100.0%) |  |
| 65 to 90 | 75 (10.6%) | 19 (9.7%) | 6 (4.4%) | 0 (0.0%) |  |
| Missing | 1 | 0 | 0 | 0 |  |
| **Gender** | | | | | 0.2210 |
| Female | 323 (45.5%) | 87 (44.6%) | 74 (54.8%) | 2 (40.0%) |  |
| Male | 387 (54.5%) | 108 (55.4%) | 61 (45.2%) | 3 (60.0%) |  |
| **First recorded weight** | | | | | 0.8143 |
| Mean (SD) | 75.61 (17.190) | 75.41 (16.681) | 76.28 (18.166) | 69.06 (21.867) |  |
| Missing | 1 | 0 | 0 | 0 |  |
| **Province/region of treating physician** | | | | | 0.1629 |
| Alberta | 44 (6.2%) | 15 (7.7%) | 5 (3.7%) | 0 (0.0%) |  |
| Atlantic^†^ | 110 (15.5%) | 17 (8.7%) | 20 (14.8%) | 1 (20.0%) |  |
| British Columbia | 94 (13.2%) | 12 (6.2%) | 16 (11.9%) | 0 (0.0%) |  |
| Ontario | 382 (53.8%) | 127 (65.1%) | 82 (60.7%) | 3 (60.0%) |  |
| Quebec | 77 (10.8%) | 23 (11.8%) | 12 (8.9%) | 1 (20.0%) |  |
| Saskatchewan/  Manitoba | 3 (0.4%) | 1 (0.5%) | 0 (0.0%) | 0 (0.0%) |  |
| **Year of initiation of IFX treatment** | | | | | 0.0016 |
| 2015 | 85 (12.0%) | 6 (3.1%) | 15 (11.1%) | 1 (20.0%) |  |
| 2016 | 125 (17.6%) | 24 (12.3%) | 18 (13.3%) | 0 (0.0%) |  |
| 2017 | 254 (35.8%) | 72 (36.9%) | 57 (42.2%) | 1 (20.0%) |  |
| 2018 | 246 (34.6%) | 93 (47.7%) | 45 (33.3%) | 3 (60.0%) |  |
| **Time to first TDM (days)** | | | | | 0.0002 |
| Mean (SD) | 342.8 (323.12) | 235.9 (252.65) | 293.1 (284.86) | 256.8 (312.72) |  |
| **Year of first instance of TDM** | | | | | 0.1130 |
| 2015 | 0 (0.0%) | 0 (0.0%) | 0 (0.0%) | 0 (0.0%) |  |
| 2016 | 0 (0.0%) | 0 (0.0%) | 0 (0.0%) | 0 (0.0%) |  |
| 2017 | 229 (32.3%) | 52 (26.7%) | 49 (36.3%) | 2 (40.0%) |  |
| 2018 | 343 (48.3%) | 103 (52.8%) | 72 (53.3%) | 3 (60.0%) |  |
| 2019 | 138 (19.4%) | 40 (20.5%) | 14 (10.4%) | 0 (0.0%) |  |

TDM=therapeutic drug monitoring. P-value: categorical variables, chi-square test; numeric variables, ANOVA.

^†^Atlantic includes New Brunswick, Nova Scotia, Prince Edward Island, Newfoundland, and Labrador.

Dose optimization thresholds: High: A treatment interval decrease of ≥11 days (1.57 weeks) with a posterior interval of ≤35 days (5 weeks), and/or a dose level increase of ≥1.5 mg/kg with a posterior dose level of ≥9 mg/kg. Low: A treatment interval decrease of ≥11 days (1.57 weeks) with a posterior interval of ≤46 days (6.57 weeks), and/or dose level increase of ≥1.5 mg/kg with a posterior dose level of ≥7 mg/kg.

A=First TDM serum IFX concentration below threshold and no dose optimization within post-index period;

B=First TDM serum IFX concentration below threshold and dose optimization within post-index period;

C=First TDM serum IFX concentration at or above threshold and no dose optimization within post-index period;

D=First TDM serum IFX concentration at or above threshold and dose optimization within post-index period.

Suppl Table 4l: Subgroup Demographics and Baseline Characteristics of Patients with UC with no DO (Based on Low Threshold Criteria) Prior to their First Instance of TDM (Serum IFX Threshold: 10 μg/mL; Post-Index Period for Dose Optimization: +17 weeks; Post-Index Dose Optimization Threshold: High)

|  | **A (N=656)** | **B (N=249)** | **C (N=133)** | **D (N=7)** | **p-value** |
| --- | --- | --- | --- | --- | --- |
| **Age (years)** | | | | | 0.1355 |
| Mean (SD) | 42.8 (16.32) | 42.9 (15.99) | 39.5 (13.58) | 39.0 (7.37) |  |
| Missing | 1 | 0 | 0 | 0 |  |
| **Age group (years)** | | | | | 0.1155 |
| 18 to 64 | 584 (89.2%) | 226 (90.8%) | 127 (95.5%) | 7 (100.0%) |  |
| 65 to 90 | 71 (10.8%) | 23 (9.2%) | 6 (4.5%) | 0 (0.0%) |  |
| Missing | 1 | 0 | 0 | 0 |  |
| **Gender** | | | | | 0.2190 |
| Female | 295 (45.0%) | 115 (46.2%) | 73 (54.9%) | 3 (42.9%) |  |
| Male | 361 (55.0%) | 134 (53.8%) | 60 (45.1%) | 4 (57.1%) |  |
| **First recorded weight** | | | | | 0.4515 |
| Mean (SD) | 75.65 (17.232) | 75.35 (16.677) | 76.55 (18.162) | 65.99 (18.708) |  |
| Missing | 1 | 0 | 0 | 0 |  |
| **Province/region of treating physician** | | | | | 0.0574 |
| Alberta | 39 (5.9%) | 20 (8.0%) | 5 (3.8%) | 0 (0.0%) |  |
| Atlantic^†^ | 106 (16.2%) | 21 (8.4%) | 19 (14.3%) | 2 (28.6%) |  |
| British Columbia | 88 (13.4%) | 18 (7.2%) | 15 (11.3%) | 1 (14.3%) |  |
| Ontario | 347 (52.9%) | 162 (65.1%) | 82 (61.7%) | 3 (42.9%) |  |
| Quebec | 73 (11.1%) | 27 (10.8%) | 12 (9.0%) | 1 (14.3%) |  |
| Saskatchewan/  Manitoba | 3 (0.5%) | 1 (0.4%) | 0 (0.0%) | 0 (0.0%) |  |
| **Year of initiation of IFX treatment** | | | | | 0.0002 |
| 2015 | 80 (12.2%) | 11 (4.4%) | 15 (11.3%) | 1 (14.3%) |  |
| 2016 | 120 (18.3%) | 29 (11.6%) | 18 (13.5%) | 0 (0.0%) |  |
| 2017 | 239 (36.4%) | 87 (34.9%) | 55 (41.4%) | 3 (42.9%) |  |
| 2018 | 217 (33.1%) | 122 (49.0%) | 45 (33.8%) | 3 (42.9%) |  |
| **Time to first TDM (days)** | | | | | <0.0001 |
| Mean (SD) | 352.9 (327.57) | 232.6 (248.09) | 295.4 (286.32) | 223.6 (263.06) |  |
| **Year of first instance of TDM** | | | | | 0.0604 |
| 2015 | 0 (0.0%) | 0 (0.0%) | 0 (0.0%) | 0 (0.0%) |  |
| 2016 | 0 (0.0%) | 0 (0.0%) | 0 (0.0%) | 0 (0.0%) |  |
| 2017 | 214 (32.6%) | 67 (26.9%) | 47 (35.3%) | 4 (57.1%) |  |
| 2018 | 317 (48.3%) | 129 (51.8%) | 72 (54.1%) | 3 (42.9%) |  |
| 2019 | 125 (19.1%) | 53 (21.3%) | 14 (10.5%) | 0 (0.0%) |  |

TDM=therapeutic drug monitoring. P-value: categorical variables, chi-square test; numeric variables, ANOVA.

^†^Atlantic includes New Brunswick, Nova Scotia, Prince Edward Island, Newfoundland, and Labrador.

Dose optimization thresholds: High: A treatment interval decrease of ≥11 days (1.57 weeks) with a posterior interval of ≤35 days (5 weeks), and/or a dose level increase of ≥1.5 mg/kg with a posterior dose level of ≥9 mg/kg. Low: A treatment interval decrease of ≥11 days (1.57 weeks) with a posterior interval of ≤46 days (6.57 weeks), and/or dose level increase of ≥1.5 mg/kg with a posterior dose level of ≥7 mg/kg.

A=First TDM serum IFX concentration below threshold and no dose optimization within post-index period;

B=First TDM serum IFX concentration below threshold and dose optimization within post-index period;

C=First TDM serum IFX concentration at or above threshold and no dose optimization within post-index period;

D=First TDM serum IFX concentration at or above threshold and dose optimization within post-index period.

Suppl Table 5a: Subgroup Demographics and Baseline Characteristics of Patients with UC with no DO (Based on High Threshold Criteria) Prior to their First Instance of TDM (Serum IFX Threshold: 3 μg/mL; Post-Index Period for Dose Optimization: +9 weeks; Post-Index Dose Optimization Threshold: Low)

|  | **A (N=308)** | **B (N=263)** | **C (N=542)** | **D (N=82)** | **p-value** |
| --- | --- | --- | --- | --- | --- |
| **Age (years)** | | | | | 0.0085 |
| Mean (SD) | 44.3 (15.91) | 43.2 (16.52) | 41.0 (15.30) | 39.8 (14.39) |  |
| Missing | 0 | 0 | 1 | 1 |  |
| **Age group (years)** | | | | | 0.0964 |
| 18 to 64 | 273 (88.6%) | 232 (88.2%) | 498 (92.1%) | 77 (95.1%) |  |
| 65 to 90 | 35 (11.4%) | 31 (11.8%) | 43 (7.9%) | 4 (4.9%) |  |
| Missing | 0 | 0 | 1 | 1 |  |
| **Gender** | | | | | 0.4560 |
| Female | 136 (44.2%) | 117 (44.5%) | 264 (48.7%) | 35 (42.7%) |  |
| Male | 172 (55.8%) | 146 (55.5%) | 278 (51.3%) | 47 (57.3%) |  |
| **First recorded weight** | | | | | 0.6013 |
| Mean (SD) | 76.12 (16.806) | 74.47 (17.115) | 76.01 (17.404) | 76.52 (18.980) |  |
| Missing | 2 | 0 | 0 | 0 |  |
| **Province/region of treating physician** | | | | | 0.3915 |
| Alberta | 22 (7.1%) | 17 (6.5%) | 27 (5.0%) | 4 (4.9%) |  |
| Atlantic^†^ | 46 (14.9%) | 32 (12.2%) | 76 (14.0%) | 9 (11.0%) |  |
| British Columbia | 46 (14.9%) | 22 (8.4%) | 68 (12.5%) | 7 (8.5%) |  |
| Ontario | 159 (51.6%) | 161 (61.2%) | 296 (54.6%) | 49 (59.8%) |  |
| Quebec | 33 (10.7%) | 30 (11.4%) | 74 (13.7%) | 13 (15.9%) |  |
| Saskatchewan/  Manitoba | 2 (0.6%) | 1 (0.4%) | 1 (0.2%) | 0 (0.0%) |  |
| **Year of initiation of IFX treatment** | | | | | <0.0001 |
| 2015 | 38 (12.3%) | 17 (6.5%) | 94 (17.3%) | 4 (4.9%) |  |
| 2016 | 68 (22.1%) | 33 (12.5%) | 96 (17.7%) | 11 (13.4%) |  |
| 2017 | 102 (33.1%) | 105 (39.9%) | 184 (33.9%) | 30 (36.6%) |  |
| 2018 | 100 (32.5%) | 108 (41.1%) | 168 (31.0%) | 37 (45.1%) |  |
| **Time to first TDM (days)** | | | | | <0.0001 |
| Mean (SD) | 380.4 (323.93) | 272.2 (277.26) | 382.1 (353.93) | 245.7 (279.22) |  |
| **Year of first instance of TDM** | | | | | 0.0056 |
| 2015 | 0 (0.0%) | 0 (0.0%) | 0 (0.0%) | 0 (0.0%) |  |
| 2016 | 0 (0.0%) | 0 (0.0%) | 0 (0.0%) | 0 (0.0%) |  |
| 2017 | 99 (32.1%) | 83 (31.6%) | 185 (34.1%) | 22 (26.8%) |  |
| 2018 | 134 (43.5%) | 123 (46.8%) | 275 (50.7%) | 50 (61.0%) |  |
| 2019 | 75 (24.4%) | 57 (21.7%) | 82 (15.1%) | 10 (12.2%) |  |

TDM=therapeutic drug monitoring. P-value: categorical variables, chi-square test; numeric variables, ANOVA.

^†^Atlantic includes New Brunswick, Nova Scotia, Prince Edward Island, Newfoundland, and Labrador.

Dose optimization thresholds: High: A treatment interval decrease of ≥11 days (1.57 weeks) with a posterior interval of ≤35 days (5 weeks), and/or a dose level increase of ≥1.5 mg/kg with a posterior dose level of ≥9 mg/kg. Low: A treatment interval decrease of ≥11 days (1.57 weeks) with a posterior interval of ≤46 days (6.57 weeks), and/or dose level increase of ≥1.5 mg/kg with a posterior dose level of ≥7 mg/kg.

A=First TDM serum IFX concentration below threshold and no dose optimization within post-index period;

B=First TDM serum IFX concentration below threshold and dose optimization within post-index period;

C=First TDM serum IFX concentration at or above threshold and no dose optimization within post-index period;

D=First TDM serum IFX concentration at or above threshold and dose optimization within post-index period.

Suppl Table 5b: Subgroup Demographics and Baseline Characteristics of Patients with UC with no DO (Based on High Threshold Criteria) Prior to their First Instance of TDM (Serum IFX Threshold: 3 μg/mL; Post-Index Period for Dose Optimization: +17 weeks; Post-Index Dose Optimization Threshold: Low)

|  | **A (N=262)** | **B (N=309)** | **C (N=496)** | **D (N=128)** | **p-value** |
| --- | --- | --- | --- | --- | --- |
| **Age (years)** | | | | | 0.0052 |
| Mean (SD) | 44.6 (16.13) | 43.1 (16.23) | 41.1 (15.37) | 39.7 (14.41) |  |
| Missing | 0 | 0 | 1 | 1 |  |
| **Age group (years)** | | | | | 0.0630 |
| 18 to 64 | 230 (87.8%) | 275 (89.0%) | 454 (91.7%) | 121 (95.3%) |  |
| 65 to 90 | 32 (12.2%) | 34 (11.0%) | 41 (8.3%) | 6 (4.7%) |  |
| Missing | 0 | 0 | 1 | 1 |  |
| **Gender** | | | | | 0.3698 |
| Female | 110 (42.0%) | 143 (46.3%) | 234 (47.2%) | 65 (50.8%) |  |
| Male | 152 (58.0%) | 166 (53.7%) | 262 (52.8%) | 63 (49.2%) |  |
| **First recorded weight** | | | | | 0.3742 |
| Mean (SD) | 76.33 (16.444) | 74.54 (17.357) | 76.44 (17.417) | 74.67 (18.309) |  |
| Missing | 2 | 0 | 0 | 0 |  |
| **Province/region of treating physician** | | | | | 0.0476 |
| Alberta | 18 (6.9%) | 21 (6.8%) | 21 (4.2%) | 10 (7.8%) |  |
| Atlantic^†^ | 43 (16.4%) | 35 (11.3%) | 71 (14.3%) | 14 (10.9%) |  |
| British Columbia | 43 (16.4%) | 25 (8.1%) | 63 (12.7%) | 12 (9.4%) |  |
| Ontario | 132 (50.4%) | 188 (60.8%) | 271 (54.6%) | 74 (57.8%) |  |
| Quebec | 24 (9.2%) | 39 (12.6%) | 69 (13.9%) | 18 (14.1%) |  |
| Saskatchewan/  Manitoba | 2 (0.8%) | 1 (0.3%) | 1 (0.2%) | 0 (0.0%) |  |
| **Year of initiation of IFX treatment** | | | | | <0.0001 |
| 2015 | 30 (11.5%) | 25 (8.1%) | 89 (17.9%) | 9 (7.0%) |  |
| 2016 | 62 (23.7%) | 39 (12.6%) | 93 (18.8%) | 14 (10.9%) |  |
| 2017 | 92 (35.1%) | 115 (37.2%) | 166 (33.5%) | 48 (37.5%) |  |
| 2018 | 78 (29.8%) | 130 (42.1%) | 148 (29.8%) | 57 (44.5%) |  |
| **Time to first TDM (days)** | | | | | <0.0001 |
| Mean (SD) | 386.8 (314.57) | 282.8 (294.18) | 394.3 (358.68) | 247.5 (273.93) |  |
| **Year of first instance of TDM** | | | | | 0.0127 |
| 2015 | 0 (0.0%) | 0 (0.0%) | 0 (0.0%) | 0 (0.0%) |  |
| 2016 | 0 (0.0%) | 0 (0.0%) | 0 (0.0%) | 0 (0.0%) |  |
| 2017 | 84 (32.1%) | 98 (31.7%) | 170 (34.3%) | 37 (28.9%) |  |
| 2018 | 115 (43.9%) | 142 (46.0%) | 253 (51.0%) | 72 (56.3%) |  |
| 2019 | 63 (24.0%) | 69 (22.3%) | 73 (14.7%) | 19 (14.8%) |  |

TDM=therapeutic drug monitoring. P-value: categorical variables, chi-square test; numeric variables, ANOVA.

^†^Atlantic includes New Brunswick, Nova Scotia, Prince Edward Island, Newfoundland, and Labrador.

Dose optimization thresholds: High: A treatment interval decrease of ≥11 days (1.57 weeks) with a posterior interval of ≤35 days (5 weeks), and/or a dose level increase of ≥1.5 mg/kg with a posterior dose level of ≥9 mg/kg. Low: A treatment interval decrease of ≥11 days (1.57 weeks) with a posterior interval of ≤46 days (6.57 weeks), and/or dose level increase of ≥1.5 mg/kg with a posterior dose level of ≥7 mg/kg.

A=First TDM serum IFX concentration below threshold and no dose optimization within post-index period;

B=First TDM serum IFX concentration below threshold and dose optimization within post-index period;

C=First TDM serum IFX concentration at or above threshold and no dose optimization within post-index period;

D=First TDM serum IFX concentration at or above threshold and dose optimization within post-index period.

Suppl Table 5c: Subgroup Demographics and Baseline Characteristics of Patients with UC with no DO (Based on High Threshold Criteria) Prior to their First Instance of TDM (Serum IFX Threshold: 5 μg/mL; Post-Index Period for Dose Optimization: +9 weeks; Post-Index Dose Optimization Threshold: Low)

|  | **A (N=454)** | **B (N=295)** | **C (N=396)** | **D (N=50)** | **p-value** |
| --- | --- | --- | --- | --- | --- |
| **Age (years)** | | | | | 0.0006 |
| Mean (SD) | 44.0 (16.02) | 43.2 (16.38) | 40.2 (14.86) | 37.8 (13.44) |  |
| Missing | 1 | 0 | 0 | 1 |  |
| **Age group (years)** | | | | | 0.0528 |
| 18 to 64 | 404 (89.2%) | 261 (88.5%) | 367 (92.7%) | 48 (98.0%) |  |
| 65 to 90 | 49 (10.8%) | 34 (11.5%) | 29 (7.3%) | 1 (2.0%) |  |
| Missing | 1 | 0 | 0 | 1 |  |
| **Gender** | | | | | 0.1740 |
| Female | 199 (43.8%) | 130 (44.1%) | 201 (50.8%) | 22 (44.0%) |  |
| Male | 255 (56.2%) | 165 (55.9%) | 195 (49.2%) | 28 (56.0%) |  |
| **First recorded weight** | | | | | 0.7717 |
| Mean (SD) | 76.03 (17.449) | 75.10 (17.367) | 76.07 (16.890) | 74.10 (18.885) |  |
| Missing | 2 | 0 | 0 | 0 |  |
| **Province/region of treating physician** | | | | | 0.6287 |
| Alberta | 29 (6.4%) | 17 (5.8%) | 20 (5.1%) | 4 (8.0%) |  |
| Atlantic^†^ | 62 (13.7%) | 36 (12.2%) | 60 (15.2%) | 5 (10.0%) |  |
| British Columbia | 64 (14.1%) | 27 (9.2%) | 50 (12.6%) | 2 (4.0%) |  |
| Ontario | 239 (52.6%) | 179 (60.7%) | 216 (54.5%) | 31 (62.0%) |  |
| Quebec | 58 (12.8%) | 35 (11.9%) | 49 (12.4%) | 8 (16.0%) |  |
| Saskatchewan/  Manitoba | 2 (0.4%) | 1 (0.3%) | 1 (0.3%) | 0 (0.0%) |  |
| **Year of initiation of IFX treatment** | | | | | <0.0001 |
| 2015 | 67 (14.8%) | 19 (6.4%) | 65 (16.4%) | 2 (4.0%) |  |
| 2016 | 90 (19.8%) | 36 (12.2%) | 74 (18.7%) | 8 (16.0%) |  |
| 2017 | 149 (32.8%) | 113 (38.3%) | 137 (34.6%) | 22 (44.0%) |  |
| 2018 | 148 (32.6%) | 127 (43.1%) | 120 (30.3%) | 18 (36.0%) |  |
| **Time to first TDM (days)** | | | | | <0.0001 |
| Mean (SD) | 387.8 (334.16) | 273.2 (283.40) | 374.3 (353.50) | 222.7 (238.09) |  |
| **Year of first instance of TDM** | | | | | 0.0126 |
| 2015 | 0 (0.0%) | 0 (0.0%) | 0 (0.0%) | 0 (0.0%) |  |
| 2016 | 0 (0.0%) | 0 (0.0%) | 0 (0.0%) | 0 (0.0%) |  |
| 2017 | 147 (32.4%) | 86 (29.2%) | 137 (34.6%) | 19 (38.0%) |  |
| 2018 | 207 (45.6%) | 145 (49.2%) | 202 (51.0%) | 28 (56.0%) |  |
| 2019 | 100 (22.0%) | 64 (21.7%) | 57 (14.4%) | 3 (6.0%) |  |

TDM=therapeutic drug monitoring. P-value: categorical variables, chi-square test; numeric variables, ANOVA.

^†^Atlantic includes New Brunswick, Nova Scotia, Prince Edward Island, Newfoundland, and Labrador.

Dose optimization thresholds: High: A treatment interval decrease of ≥11 days (1.57 weeks) with a posterior interval of ≤35 days (5 weeks), and/or a dose level increase of ≥1.5 mg/kg with a posterior dose level of ≥9 mg/kg. Low: A treatment interval decrease of ≥11 days (1.57 weeks) with a posterior interval of ≤46 days (6.57 weeks), and/or dose level increase of ≥1.5 mg/kg with a posterior dose level of ≥7 mg/kg.

A=First TDM serum IFX concentration below threshold and no dose optimization within post-index period;

B=First TDM serum IFX concentration below threshold and dose optimization within post-index period;

C=First TDM serum IFX concentration at or above threshold and no dose optimization within post-index period;

D=First TDM serum IFX concentration at or above threshold and dose optimization within post-index period.

Suppl Table 5d: Subgroup Demographics and Baseline Characteristics of Patients with UC with no DO (Based on High Threshold Criteria) Prior to their First Instance of TDM (Serum IFX Threshold: 5 μg/mL; Post-Index Period for Dose Optimization: +17 weeks; Post-Index Dose Optimization Threshold: Low)

|  | **A (N=387)** | **B (N=362)** | **C (N=371)** | **D (N=75)** | **p-value** |
| --- | --- | --- | --- | --- | --- |
| **Age (years)** | | | | | 0.0004 |
| Mean (SD) | 44.4 (16.22) | 42.9 (16.08) | 40.3 (14.91) | 38.3 (13.68) |  |
| Missing | 1 | 0 | 0 | 1 |  |
| **Age group (years)** | | | | | 0.0430 |
| 18 to 64 | 341 (88.3%) | 324 (89.5%) | 343 (92.5%) | 72 (97.3%) |  |
| 65 to 90 | 45 (11.7%) | 38 (10.5%) | 28 (7.5%) | 2 (2.7%) |  |
| Missing | 1 | 0 | 0 | 1 |  |
| **Gender** | | | | | 0.0894 |
| Female | 163 (42.1%) | 166 (45.9%) | 181 (48.8%) | 42 (56.0%) |  |
| Male | 224 (57.9%) | 196 (54.1%) | 190 (51.2%) | 33 (44.0%) |  |
| **First recorded weight** | | | | | 0.1922 |
| Mean (SD) | 76.25 (17.510) | 75.05 (17.308) | 76.57 (16.640) | 72.30 (19.012) |  |
| Missing | 2 | 0 | 0 | 0 |  |
| **Province/region of treating physician** | | | | | 0.0596 |
| Alberta | 24 (6.2%) | 22 (6.1%) | 15 (4.0%) | 9 (12.0%) |  |
| Atlantic^†^ | 54 (14.0%) | 44 (12.2%) | 60 (16.2%) | 5 (6.7%) |  |
| British Columbia | 60 (15.5%) | 31 (8.6%) | 46 (12.4%) | 6 (8.0%) |  |
| Ontario | 200 (51.7%) | 218 (60.2%) | 203 (54.7%) | 44 (58.7%) |  |
| Quebec | 47 (12.1%) | 46 (12.7%) | 46 (12.4%) | 11 (14.7%) |  |
| Saskatchewan/  Manitoba | 2 (0.5%) | 1 (0.3%) | 1 (0.3%) | 0 (0.0%) |  |
| **Year of initiation of IFX treatment** | | | | | <0.0001 |
| 2015 | 57 (14.7%) | 29 (8.0%) | 62 (16.7%) | 5 (6.7%) |  |
| 2016 | 83 (21.4%) | 43 (11.9%) | 72 (19.4%) | 10 (13.3%) |  |
| 2017 | 132 (34.1%) | 130 (35.9%) | 126 (34.0%) | 33 (44.0%) |  |
| 2018 | 115 (29.7%) | 160 (44.2%) | 111 (29.9%) | 27 (36.0%) |  |
| **Time to first TDM (days)** | | | | | <0.0001 |
| Mean (SD) | 403.2 (332.61) | 278.0 (292.62) | 379.8 (355.32) | 245.7 (268.17) |  |
| **Year of first instance of TDM** | | | | | 0.0266 |
| 2015 | 0 (0.0%) | 0 (0.0%) | 0 (0.0%) | 0 (0.0%) |  |
| 2016 | 0 (0.0%) | 0 (0.0%) | 0 (0.0%) | 0 (0.0%) |  |
| 2017 | 126 (32.6%) | 107 (29.6%) | 128 (34.5%) | 28 (37.3%) |  |
| 2018 | 176 (45.5%) | 176 (48.6%) | 192 (51.8%) | 38 (50.7%) |  |
| 2019 | 85 (22.0%) | 79 (21.8%) | 51 (13.7%) | 9 (12.0%) |  |

TDM=therapeutic drug monitoring. P-value: categorical variables, chi-square test; numeric variables, ANOVA.

^†^Atlantic includes New Brunswick, Nova Scotia, Prince Edward Island, Newfoundland, and Labrador.

Dose optimization thresholds: High: A treatment interval decrease of ≥11 days (1.57 weeks) with a posterior interval of ≤35 days (5 weeks), and/or a dose level increase of ≥1.5 mg/kg with a posterior dose level of ≥9 mg/kg. Low: A treatment interval decrease of ≥11 days (1.57 weeks) with a posterior interval of ≤46 days (6.57 weeks), and/or dose level increase of ≥1.5 mg/kg with a posterior dose level of ≥7 mg/kg.

A=First TDM serum IFX concentration below threshold and no dose optimization within post-index period;

B=First TDM serum IFX concentration below threshold and dose optimization within post-index period;

C=First TDM serum IFX concentration at or above threshold and no dose optimization within post-index period;

D=First TDM serum IFX concentration at or above threshold and dose optimization within post-index period.

Suppl Table 5e: Subgroup Demographics and Baseline Characteristics of Patients with UC with no DO (Based on High Threshold Criteria) Prior to their First Instance of TDM (Serum IFX Threshold: 10 μg/mL; Post-Index Period for Dose Optimization: +9 weeks; Post-Index Dose Optimization Threshold: Low)

|  | **A (N=694)** | **B (N=331)** | **C (N=156)** | **D (N=14)** | **p-value** |
| --- | --- | --- | --- | --- | --- |
| **Age (years)** | | | | | 0.0837 |
| Mean (SD) | 42.8 (15.97) | 42.6 (16.30) | 39.5 (13.55) | 38.2 (8.27) |  |
| Missing | 1 | 0 | 0 | 1 |  |
| **Age group (years)** | | | | | 0.1338 |
| 18 to 64 | 623 (89.9%) | 296 (89.4%) | 148 (94.9%) | 13 (100.0%) |  |
| 65 to 90 | 70 (10.1%) | 35 (10.6%) | 8 (5.1%) | 0 (0.0%) |  |
| Missing | 1 | 0 | 0 | 1 |  |
| **Gender** | | | | | 0.0296 |
| Female | 311 (44.8%) | 147 (44.4%) | 89 (57.1%) | 5 (35.7%) |  |
| Male | 383 (55.2%) | 184 (55.6%) | 67 (42.9%) | 9 (64.3%) |  |
| **First recorded weight** | | | | | 0.3676 |
| Mean (SD) | 75.98 (17.003) | 75.24 (17.675) | 76.38 (17.998) | 68.37 (13.770) |  |
| Missing | 2 | 0 | 0 | 0 |  |
| **Province/region of treating physician** | | | | | 0.3232 |
| Alberta | 44 (6.3%) | 20 (6.0%) | 5 (3.2%) | 1 (7.1%) |  |
| Atlantic^†^ | 101 (14.6%) | 38 (11.5%) | 21 (13.5%) | 3 (21.4%) |  |
| British Columbia | 94 (13.5%) | 28 (8.5%) | 20 (12.8%) | 1 (7.1%) |  |
| Ontario | 361 (52.0%) | 204 (61.6%) | 94 (60.3%) | 6 (42.9%) |  |
| Quebec | 91 (13.1%) | 40 (12.1%) | 16 (10.3%) | 3 (21.4%) |  |
| Saskatchewan/  Manitoba | 3 (0.4%) | 1 (0.3%) | 0 (0.0%) | 0 (0.0%) |  |
| **Year of initiation of IFX treatment** | | | | | <0.0001 |
| 2015 | 108 (15.6%) | 20 (6.0%) | 24 (15.4%) | 1 (7.1%) |  |
| 2016 | 139 (20.0%) | 40 (12.1%) | 25 (16.0%) | 4 (28.6%) |  |
| 2017 | 227 (32.7%) | 130 (39.3%) | 59 (37.8%) | 5 (35.7%) |  |
| 2018 | 220 (31.7%) | 141 (42.6%) | 48 (30.8%) | 4 (28.6%) |  |
| **Time to first TDM (days)** | | | | | <0.0001 |
| Mean (SD) | 387.2 (346.51) | 265.0 (279.13) | 356.0 (327.72) | 287.7 (245.14) |  |
| **Year of first instance of TDM** | | | | | 0.1347 |
| 2015 | 0 (0.0%) | 0 (0.0%) | 0 (0.0%) | 0 (0.0%) |  |
| 2016 | 0 (0.0%) | 0 (0.0%) | 0 (0.0%) | 0 (0.0%) |  |
| 2017 | 227 (32.7%) | 99 (29.9%) | 57 (36.5%) | 6 (42.9%) |  |
| 2018 | 329 (47.4%) | 165 (49.8%) | 80 (51.3%) | 8 (57.1%) |  |
| 2019 | 138 (19.9%) | 67 (20.2%) | 19 (12.2%) | 0 (0.0%) |  |

TDM=therapeutic drug monitoring. P-value: categorical variables, chi-square test; numeric variables, ANOVA.

^†^Atlantic includes New Brunswick, Nova Scotia, Prince Edward Island, Newfoundland, and Labrador.

Dose optimization thresholds: High: A treatment interval decrease of ≥11 days (1.57 weeks) with a posterior interval of ≤35 days (5 weeks), and/or a dose level increase of ≥1.5 mg/kg with a posterior dose level of ≥9 mg/kg. Low: A treatment interval decrease of ≥11 days (1.57 weeks) with a posterior interval of ≤46 days (6.57 weeks), and/or dose level increase of ≥1.5 mg/kg with a posterior dose level of ≥7 mg/kg.

A=First TDM serum IFX concentration below threshold and no dose optimization within post-index period;

B=First TDM serum IFX concentration below threshold and dose optimization within post-index period;

C=First TDM serum IFX concentration at or above threshold and no dose optimization within post-index period;

D=First TDM serum IFX concentration at or above threshold and dose optimization within post-index period.

Suppl Table 5f: Subgroup Demographics and Baseline Characteristics of Patients with UC with no DO (Based on High Threshold Criteria) Prior to their First Instance of TDM (Serum IFX Threshold: 10 μg/mL; Post-Index Period for Dose Optimization: +17 weeks; Post-Index Dose Optimization Threshold: Low)

|  | **A (N=611)** | **B (N=414)** | **C (N=147)** | **D (N=23)** | **p-value** |
| --- | --- | --- | --- | --- | --- |
| **Age (years)** | | | | | 0.0653 |
| Mean (SD) | 43.1 (16.12) | 42.2 (16.00) | 39.4 (13.56) | 39.7 (10.86) |  |
| Missing | 1 | 0 | 0 | 1 |  |
| **Age group (years)** | | | | | 0.1068 |
| 18 to 64 | 545 (89.3%) | 374 (90.3%) | 139 (94.6%) | 22 (100.0%) |  |
| 65 to 90 | 65 (10.7%) | 40 (9.7%) | 8 (5.4%) | 0 (0.0%) |  |
| Missing | 1 | 0 | 0 | 1 |  |
| **Gender** | | | | | 0.0410 |
| Female | 263 (43.0%) | 195 (47.1%) | 81 (55.1%) | 13 (56.5%) |  |
| Male | 348 (57.0%) | 219 (52.9%) | 66 (44.9%) | 10 (43.5%) |  |
| **First recorded weight** | | | | | 0.0657 |
| Mean (SD) | 76.27 (16.867) | 74.96 (17.715) | 76.98 (17.975) | 67.65 (14.448) |  |
| Missing | 2 | 0 | 0 | 0 |  |
| **Province/region of treating physician** | | | | | 0.0910 |
| Alberta | 34 (5.6%) | 30 (7.2%) | 5 (3.4%) | 1 (4.3%) |  |
| Atlantic^†^ | 93 (15.2%) | 46 (11.1%) | 21 (14.3%) | 3 (13.0%) |  |
| British Columbia | 89 (14.6%) | 33 (8.0%) | 17 (11.6%) | 4 (17.4%) |  |
| Ontario | 314 (51.4%) | 251 (60.6%) | 89 (60.5%) | 11 (47.8%) |  |
| Quebec | 78 (12.8%) | 53 (12.8%) | 15 (10.2%) | 4 (17.4%) |  |
| Saskatchewan/  Manitoba | 3 (0.5%) | 1 (0.2%) | 0 (0.0%) | 0 (0.0%) |  |
| **Year of initiation of IFX treatment** | | | | | <0.0001 |
| 2015 | 97 (15.9%) | 31 (7.5%) | 22 (15.0%) | 3 (13.0%) |  |
| 2016 | 130 (21.3%) | 49 (11.8%) | 25 (17.0%) | 4 (17.4%) |  |
| 2017 | 204 (33.4%) | 153 (37.0%) | 54 (36.7%) | 10 (43.5%) |  |
| 2018 | 180 (29.5%) | 181 (43.7%) | 46 (31.3%) | 6 (26.1%) |  |
| **Time to first TDM (days)** | | | | | <0.0001 |
| Mean (SD) | 400.8 (347.93) | 269.5 (287.67) | 354.3 (324.98) | 325.4 (305.45) |  |
| **Year of first instance of TDM** | | | | | 0.1703 |
| 2015 | 0 (0.0%) | 0 (0.0%) | 0 (0.0%) | 0 (0.0%) |  |
| 2016 | 0 (0.0%) | 0 (0.0%) | 0 (0.0%) | 0 (0.0%) |  |
| 2017 | 201 (32.9%) | 125 (30.2%) | 53 (36.1%) | 10 (43.5%) |  |
| 2018 | 291 (47.6%) | 203 (49.0%) | 77 (52.4%) | 11 (47.8%) |  |
| 2019 | 119 (19.5%) | 86 (20.8%) | 17 (11.6%) | 2 (8.7%) |  |

TDM=therapeutic drug monitoring. P-value: categorical variables, chi-square test; numeric variables, ANOVA.

^†^Atlantic includes New Brunswick, Nova Scotia, Prince Edward Island, Newfoundland, and Labrador.

Dose optimization thresholds: High: A treatment interval decrease of ≥11 days (1.57 weeks) with a posterior interval of ≤35 days (5 weeks), and/or a dose level increase of ≥1.5 mg/kg with a posterior dose level of ≥9 mg/kg. Low: A treatment interval decrease of ≥11 days (1.57 weeks) with a posterior interval of ≤46 days (6.57 weeks), and/or dose level increase of ≥1.5 mg/kg with a posterior dose level of ≥7 mg/kg.

A=First TDM serum IFX concentration below threshold and no dose optimization within post-index period;

B=First TDM serum IFX concentration below threshold and dose optimization within post-index period;

C=First TDM serum IFX concentration at or above threshold and no dose optimization within post-index period;

D=First TDM serum IFX concentration at or above threshold and dose optimization within post-index period.

Suppl Table 5g: Subgroup Demographics and Baseline Characteristics of Patients with UC with no DO (Based on High Threshold Criteria) Prior to their First Instance of TDM (Serum IFX Threshold: 3 μg/mL; Post-Index Period for Dose Optimization: +9 weeks; Post-Index Dose Optimization Threshold: High)

|  | **A (N=390)** | **B (N=181)** | **C (N=580)** | **D (N=44)** | **p-value** |
| --- | --- | --- | --- | --- | --- |
| **Age (years)** | | | | | 0.0123 |
| Mean (SD) | 44.1 (16.07) | 43.2 (16.47) | 40.8 (15.20) | 41.0 (15.10) |  |
| Missing | 0 | 0 | 1 | 1 |  |
| **Age group (years)** | | | | | 0.1070 |
| 18 to 64 | 344 (88.2%) | 161 (89.0%) | 534 (92.2%) | 41 (95.3%) |  |
| 65 to 90 | 46 (11.8%) | 20 (11.0%) | 45 (7.8%) | 2 (4.7%) |  |
| Missing | 0 | 0 | 1 | 1 |  |
| **Gender** | | | | | 0.6325 |
| Female | 175 (44.9%) | 78 (43.1%) | 278 (47.9%) | 21 (47.7%) |  |
| Male | 215 (55.1%) | 103 (56.9%) | 302 (52.1%) | 23 (52.3%) |  |
| **First recorded weight** | | | | | 0.7976 |
| Mean (SD) | 75.70 (17.201) | 74.62 (16.436) | 76.06 (17.479) | 76.37 (19.390) |  |
| Missing | 2 | 0 | 0 | 0 |  |
| **Province/region of treating physician** | | | | | 0.0147 |
| Alberta | 23 (5.9%) | 16 (8.8%) | 31 (5.3%) | 0 (0.0%) |  |
| Atlantic^†^ | 58 (14.9%) | 20 (11.0%) | 84 (14.5%) | 1 (2.3%) |  |
| British Columbia | 58 (14.9%) | 10 (5.5%) | 69 (11.9%) | 6 (13.6%) |  |
| Ontario | 204 (52.3%) | 116 (64.1%) | 317 (54.7%) | 28 (63.6%) |  |
| Quebec | 45 (11.5%) | 18 (9.9%) | 78 (13.4%) | 9 (20.5%) |  |
| Saskatchewan/  Manitoba | 2 (0.5%) | 1 (0.6%) | 1 (0.2%) | 0 (0.0%) |  |
| **Year of initiation of IFX treatment** | | | | | <0.0001 |
| 2015 | 47 (12.1%) | 8 (4.4%) | 96 (16.6%) | 2 (4.5%) |  |
| 2016 | 76 (19.5%) | 25 (13.8%) | 102 (17.6%) | 5 (11.4%) |  |
| 2017 | 136 (34.9%) | 71 (39.2%) | 203 (35.0%) | 11 (25.0%) |  |
| 2018 | 131 (33.6%) | 77 (42.5%) | 179 (30.9%) | 26 (59.1%) |  |
| **Time to first TDM (days)** | | | | | <0.0001 |
| Mean (SD) | 364.7 (320.08) | 256.9 (265.87) | 373.9 (350.81) | 236.8 (280.55) |  |
| **Year of first instance of TDM** | | | | | 0.0013 |
| 2015 | 0 (0.0%) | 0 (0.0%) | 0 (0.0%) | 0 (0.0%) |  |
| 2016 | 0 (0.0%) | 0 (0.0%) | 0 (0.0%) | 0 (0.0%) |  |
| 2017 | 127 (32.6%) | 55 (30.4%) | 199 (34.3%) | 8 (18.2%) |  |
| 2018 | 171 (43.8%) | 86 (47.5%) | 294 (50.7%) | 31 (70.5%) |  |
| 2019 | 92 (23.6%) | 40 (22.1%) | 87 (15.0%) | 5 (11.4%) |  |

TDM=therapeutic drug monitoring. P-value: categorical variables, chi-square test; numeric variables, ANOVA.

^†^Atlantic includes New Brunswick, Nova Scotia, Prince Edward Island, Newfoundland, and Labrador.

Dose optimization thresholds: High: A treatment interval decrease of ≥11 days (1.57 weeks) with a posterior interval of ≤35 days (5 weeks), and/or a dose level increase of ≥1.5 mg/kg with a posterior dose level of ≥9 mg/kg. Low: A treatment interval decrease of ≥11 days (1.57 weeks) with a posterior interval of ≤46 days (6.57 weeks), and/or dose level increase of ≥1.5 mg/kg with a posterior dose level of ≥7 mg/kg.

A=First TDM serum IFX concentration below threshold and no dose optimization within post-index period;

B=First TDM serum IFX concentration below threshold and dose optimization within post-index period;

C=First TDM serum IFX concentration at or above threshold and no dose optimization within post-index period;

D=First TDM serum IFX concentration at or above threshold and dose optimization within post-index period.

Suppl Table 5h: Subgroup Demographics and Baseline Characteristics of Patients with UC with no DO (Based on High Threshold Criteria) Prior to their First Instance of TDM (Serum IFX Threshold: 3 μg/mL; Post-Index Period for Dose Optimization: +17 weeks; Post-Index Dose Optimization Threshold: High)

|  | **A (N=355)** | **B (N=216)** | **C (N=548)** | **D (N=76)** | **p-value** |
| --- | --- | --- | --- | --- | --- |
| **Age (years)** | | | | | 0.0125 |
| Mean (SD) | 44.1 (16.26) | 43.3 (16.09) | 40.8 (15.27) | 41.0 (14.61) |  |
| Missing | 0 | 0 | 1 | 1 |  |
| **Age group (years)** | | | | | 0.0941 |
| 18 to 64 | 312 (87.9%) | 193 (89.4%) | 504 (92.1%) | 71 (94.7%) |  |
| 65 to 90 | 43 (12.1%) | 23 (10.6%) | 43 (7.9%) | 4 (5.3%) |  |
| Missing | 0 | 0 | 1 | 1 |  |
| **Gender** | | | | | 0.5028 |
| Female | 158 (44.5%) | 95 (44.0%) | 259 (47.3%) | 40 (52.6%) |  |
| Male | 197 (55.5%) | 121 (56.0%) | 289 (52.7%) | 36 (47.4%) |  |
| **First recorded weight** | | | | | 0.6754 |
| Mean (SD) | 75.54 (17.200) | 75.06 (16.580) | 76.33 (17.602) | 74.31 (17.630) |  |
| Missing | 2 | 0 | 0 | 0 |  |
| **Province/region of treating physician** | | | | | 0.0366 |
| Alberta | 22 (6.2%) | 17 (7.9%) | 27 (4.9%) | 4 (5.3%) |  |
| Atlantic^†^ | 57 (16.1%) | 21 (9.7%) | 80 (14.6%) | 5 (6.6%) |  |
| British Columbia | 54 (15.2%) | 14 (6.5%) | 65 (11.9%) | 10 (13.2%) |  |
| Ontario | 182 (51.3%) | 138 (63.9%) | 298 (54.4%) | 47 (61.8%) |  |
| Quebec | 38 (10.7%) | 25 (11.6%) | 77 (14.1%) | 10 (13.2%) |  |
| Saskatchewan/  Manitoba | 2 (0.6%) | 1 (0.5%) | 1 (0.2%) | 0 (0.0%) |  |
| **Year of initiation of IFX treatment** | | | | | <0.0001 |
| 2015 | 39 (11.0%) | 16 (7.4%) | 95 (17.3%) | 3 (3.9%) |  |
| 2016 | 72 (20.3%) | 29 (13.4%) | 101 (18.4%) | 6 (7.9%) |  |
| 2017 | 128 (36.1%) | 79 (36.6%) | 189 (34.5%) | 25 (32.9%) |  |
| 2018 | 116 (32.7%) | 92 (42.6%) | 163 (29.7%) | 42 (55.3%) |  |
| **Time to first TDM (days)** | | | | | <0.0001 |
| Mean (SD) | 366.3 (319.46) | 271.8 (278.60) | 386.0 (355.39) | 207.3 (236.37) |  |
| **Year of first instance of TDM** | | | | | 0.0034 |
| 2015 | 0 (0.0%) | 0 (0.0%) | 0 (0.0%) | 0 (0.0%) |  |
| 2016 | 0 (0.0%) | 0 (0.0%) | 0 (0.0%) | 0 (0.0%) |  |
| 2017 | 114 (32.1%) | 68 (31.5%) | 190 (34.7%) | 17 (22.4%) |  |
| 2018 | 157 (44.2%) | 100 (46.3%) | 278 (50.7%) | 47 (61.8%) |  |
| 2019 | 84 (23.7%) | 48 (22.2%) | 80 (14.6%) | 12 (15.8%) |  |

TDM=therapeutic drug monitoring. P-value: categorical variables, chi-square test; numeric variables, ANOVA.

^†^Atlantic includes New Brunswick, Nova Scotia, Prince Edward Island, Newfoundland, and Labrador.

Dose optimization thresholds: High: A treatment interval decrease of ≥11 days (1.57 weeks) with a posterior interval of ≤35 days (5 weeks), and/or a dose level increase of ≥1.5 mg/kg with a posterior dose level of ≥9 mg/kg. Low: A treatment interval decrease of ≥11 days (1.57 weeks) with a posterior interval of ≤46 days (6.57 weeks), and/or dose level increase of ≥1.5 mg/kg with a posterior dose level of ≥7 mg/kg.

A=First TDM serum IFX concentration below threshold and no dose optimization within post-index period;

B=First TDM serum IFX concentration below threshold and dose optimization within post-index period;

C=First TDM serum IFX concentration at or above threshold and no dose optimization within post-index period;

D=First TDM serum IFX concentration at or above threshold and dose optimization within post-index period.

Suppl Table 5i: Subgroup Demographics and Baseline Characteristics of Patients with UC with no DO (Based on High Threshold Criteria) Prior to their First Instance of TDM (Serum IFX Threshold: 5 μg/mL; Post-Index Period for Dose Optimization: +9 weeks; Post-Index Dose Optimization Threshold: High)

|  | **A (N=549)** | **B (N=200)** | **C (N=421)** | **D (N=25)** | **p-value** |
| --- | --- | --- | --- | --- | --- |
| **Age (years)** | | | | | 0.0009 |
| Mean (SD) | 43.7 (16.10) | 43.5 (16.35) | 40.1 (14.76) | 37.2 (14.03) |  |
| Missing | 1 | 0 | 0 | 1 |  |
| **Age group (years)** | | | | | 0.0571 |
| 18 to 64 | 487 (88.9%) | 178 (89.0%) | 391 (92.9%) | 24 (100.0%) |  |
| 65 to 90 | 61 (11.1%) | 22 (11.0%) | 30 (7.1%) | 0 (0.0%) |  |
| Missing | 1 | 0 | 0 | 1 |  |
| **Gender** | | | | | 0.2394 |
| Female | 242 (44.1%) | 87 (43.5%) | 211 (50.1%) | 12 (48.0%) |  |
| Male | 307 (55.9%) | 113 (56.5%) | 210 (49.9%) | 13 (52.0%) |  |
| **First recorded weight** | | | | | 0.8118 |
| Mean (SD) | 75.83 (17.636) | 75.21 (16.816) | 76.02 (17.015) | 72.97 (18.838) |  |
| Missing | 2 | 0 | 0 | 0 |  |
| **Province/region of treating physician** | | | | | 0.1793 |
| Alberta | 30 (5.5%) | 16 (8.0%) | 24 (5.7%) | 0 (0.0%) |  |
| Atlantic^†^ | 78 (14.2%) | 20 (10.0%) | 64 (15.2%) | 1 (4.0%) |  |
| British Columbia | 76 (13.8%) | 15 (7.5%) | 51 (12.1%) | 1 (4.0%) |  |
| Ontario | 292 (53.2%) | 126 (63.0%) | 229 (54.4%) | 18 (72.0%) |  |
| Quebec | 71 (12.9%) | 22 (11.0%) | 52 (12.4%) | 5 (20.0%) |  |
| Saskatchewan/  Manitoba | 2 (0.4%) | 1 (0.5%) | 1 (0.2%) | 0 (0.0%) |  |
| **Year of initiation of IFX treatment** | | | | | 0.0003 |
| 2015 | 78 (14.2%) | 8 (4.0%) | 65 (15.4%) | 2 (8.0%) |  |
| 2016 | 99 (18.0%) | 27 (13.5%) | 79 (18.8%) | 3 (12.0%) |  |
| 2017 | 187 (34.1%) | 75 (37.5%) | 152 (36.1%) | 7 (28.0%) |  |
| 2018 | 185 (33.7%) | 90 (45.0%) | 125 (29.7%) | 13 (52.0%) |  |
| **Time to first TDM (days)** | | | | | <0.0001 |
| Mean (SD) | 373.4 (331.14) | 258.3 (270.04) | 366.0 (348.55) | 210.4 (254.98) |  |
| **Year of first instance of TDM** | | | | | 0.0044 |
| 2015 | 0 (0.0%) | 0 (0.0%) | 0 (0.0%) | 0 (0.0%) |  |
| 2016 | 0 (0.0%) | 0 (0.0%) | 0 (0.0%) | 0 (0.0%) |  |
| 2017 | 178 (32.4%) | 55 (27.5%) | 148 (35.2%) | 8 (32.0%) |  |
| 2018 | 252 (45.9%) | 100 (50.0%) | 213 (50.6%) | 17 (68.0%) |  |
| 2019 | 119 (21.7%) | 45 (22.5%) | 60 (14.3%) | 0 (0.0%) |  |

TDM=therapeutic drug monitoring. P-value: categorical variables, chi-square test; numeric variables, ANOVA.

^†^Atlantic includes New Brunswick, Nova Scotia, Prince Edward Island, Newfoundland, and Labrador.

Dose optimization thresholds: High: A treatment interval decrease of ≥11 days (1.57 weeks) with a posterior interval of ≤35 days (5 weeks), and/or a dose level increase of ≥1.5 mg/kg with a posterior dose level of ≥9 mg/kg. Low: A treatment interval decrease of ≥11 days (1.57 weeks) with a posterior interval of ≤46 days (6.57 weeks), and/or dose level increase of ≥1.5 mg/kg with a posterior dose level of ≥7 mg/kg.

A=First TDM serum IFX concentration below threshold and no dose optimization within post-index period;

B=First TDM serum IFX concentration below threshold and dose optimization within post-index period;

C=First TDM serum IFX concentration at or above threshold and no dose optimization within post-index period;

D=First TDM serum IFX concentration at or above threshold and dose optimization within post-index period.

Suppl Table 5j: Subgroup Demographics and Baseline Characteristics of Patients with UC with no DO (Based on High Threshold Criteria) Prior to their First Instance of TDM (Serum IFX Threshold: 5 μg/mL; Post-Index Period for Dose Optimization: +17 weeks; Post-Index Dose Optimization Threshold: High)

|  | **A (N=499)** | **B (N=250)** | **C (N=404)** | **D (N=42)** | **p-value** |
| --- | --- | --- | --- | --- | --- |
| **Age (years)** | | | | | 0.0011 |
| Mean (SD) | 43.8 (16.25) | 43.4 (16.01) | 40.0 (14.85) | 38.7 (13.41) |  |
| Missing | 1 | 0 | 0 | 1 |  |
| **Age group (years)** | | | | | 0.0613 |
| 18 to 64 | 441 (88.6%) | 224 (89.6%) | 375 (92.8%) | 40 (97.6%) |  |
| 65 to 90 | 57 (11.4%) | 26 (10.4%) | 29 (7.2%) | 1 (2.4%) |  |
| Missing | 1 | 0 | 0 | 1 |  |
| **Gender** | | | | | 0.1621 |
| Female | 218 (43.7%) | 111 (44.4%) | 199 (49.3%) | 24 (57.1%) |  |
| Male | 281 (56.3%) | 139 (55.6%) | 205 (50.7%) | 18 (42.9%) |  |
| **First recorded weight** | | | | | 0.4096 |
| Mean (SD) | 75.80 (17.745) | 75.41 (16.758) | 76.29 (17.075) | 71.62 (17.111) |  |
| Missing | 2 | 0 | 0 | 0 |  |
| **Province/region of treating physician** | | | | | 0.1418 |
| Alberta | 29 (5.8%) | 17 (6.8%) | 20 (5.0%) | 4 (9.5%) |  |
| Atlantic^†^ | 74 (14.8%) | 24 (9.6%) | 63 (15.6%) | 2 (4.8%) |  |
| British Columbia | 72 (14.4%) | 19 (7.6%) | 47 (11.6%) | 5 (11.9%) |  |
| Ontario | 258 (51.7%) | 160 (64.0%) | 222 (55.0%) | 25 (59.5%) |  |
| Quebec | 64 (12.8%) | 29 (11.6%) | 51 (12.6%) | 6 (14.3%) |  |
| Saskatchewan/  Manitoba | 2 (0.4%) | 1 (0.4%) | 1 (0.2%) | 0 (0.0%) |  |
| **Year of initiation of IFX treatment** | | | | | <0.0001 |
| 2015 | 70 (14.0%) | 16 (6.4%) | 64 (15.8%) | 3 (7.1%) |  |
| 2016 | 94 (18.8%) | 32 (12.8%) | 79 (19.6%) | 3 (7.1%) |  |
| 2017 | 174 (34.9%) | 88 (35.2%) | 143 (35.4%) | 16 (38.1%) |  |
| 2018 | 161 (32.3%) | 114 (45.6%) | 118 (29.2%) | 20 (47.6%) |  |
| **Time to first TDM (days)** | | | | | <0.0001 |
| Mean (SD) | 381.5 (333.69) | 265.2 (275.08) | 374.2 (351.65) | 194.1 (225.74) |  |
| **Year of first instance of TDM** | | | | | 0.0181 |
| 2015 | 0 (0.0%) | 0 (0.0%) | 0 (0.0%) | 0 (0.0%) |  |
| 2016 | 0 (0.0%) | 0 (0.0%) | 0 (0.0%) | 0 (0.0%) |  |
| 2017 | 163 (32.7%) | 70 (28.0%) | 141 (34.9%) | 15 (35.7%) |  |
| 2018 | 228 (45.7%) | 124 (49.6%) | 207 (51.2%) | 23 (54.8%) |  |
| 2019 | 108 (21.6%) | 56 (22.4%) | 56 (13.9%) | 4 (9.5%) |  |

TDM=therapeutic drug monitoring. P-value: categorical variables, chi-square test; numeric variables, ANOVA.

^†^Atlantic includes New Brunswick, Nova Scotia, Prince Edward Island, Newfoundland, and Labrador.

Dose optimization thresholds: High: A treatment interval decrease of ≥11 days (1.57 weeks) with a posterior interval of ≤35 days (5 weeks), and/or a dose level increase of ≥1.5 mg/kg with a posterior dose level of ≥9 mg/kg. Low: A treatment interval decrease of ≥11 days (1.57 weeks) with a posterior interval of ≤46 days (6.57 weeks), and/or dose level increase of ≥1.5 mg/kg with a posterior dose level of ≥7 mg/kg.

A=First TDM serum IFX concentration below threshold and no dose optimization within post-index period;

B=First TDM serum IFX concentration below threshold and dose optimization within post-index period;

C=First TDM serum IFX concentration at or above threshold and no dose optimization within post-index period;

D=First TDM serum IFX concentration at or above threshold and dose optimization within post-index period.

Suppl Table 5k: Subgroup Demographics and Baseline Characteristics of Patients with UC with no DO (Based on High Threshold Criteria) Prior to their First Instance of TDM (Serum IFX Threshold: 10 μg/mL; Post-Index Period for Dose Optimization: +9 weeks; Post-Index Dose Optimization Threshold: High)

|  | **A (N=807)** | **B (N=218)** | **C (N=163)** | **D (N=7)** | **p-value** |
| --- | --- | --- | --- | --- | --- |
| **Age (years)** | | | | | 0.0885 |
| Mean (SD) | 42.7 (15.99) | 42.9 (16.38) | 39.4 (13.39) | 39.3 (7.09) |  |
| Missing | 1 | 0 | 0 | 1 |  |
| **Age group (years)** | | | | | 0.1494 |
| 18 to 64 | 723 (89.7%) | 196 (89.9%) | 155 (95.1%) | 6 (100.0%) |  |
| 65 to 90 | 83 (10.3%) | 22 (10.1%) | 8 (4.9%) | 0 (0.0%) |  |
| Missing | 1 | 0 | 0 | 1 |  |
| **Gender** | | | | | 0.0686 |
| Female | 362 (44.9%) | 96 (44.0%) | 91 (55.8%) | 3 (42.9%) |  |
| Male | 445 (55.1%) | 122 (56.0%) | 72 (44.2%) | 4 (57.1%) |  |
| **First recorded weight** | | | | | 0.7602 |
| Mean (SD) | 75.91 (17.288) | 75.12 (16.984) | 75.97 (17.765) | 69.92 (18.812) |  |
| Missing | 2 | 0 | 0 | 0 |  |
| **Province/region of treating physician** | | | | | 0.2586 |
| Alberta | 48 (5.9%) | 16 (7.3%) | 6 (3.7%) | 0 (0.0%) |  |
| Atlantic^†^ | 119 (14.7%) | 20 (9.2%) | 23 (14.1%) | 1 (14.3%) |  |
| British Columbia | 107 (13.3%) | 15 (6.9%) | 20 (12.3%) | 1 (14.3%) |  |
| Ontario | 425 (52.7%) | 140 (64.2%) | 96 (58.9%) | 4 (57.1%) |  |
| Quebec | 105 (13.0%) | 26 (11.9%) | 18 (11.0%) | 1 (14.3%) |  |
| Saskatchewan/  Manitoba | 3 (0.4%) | 1 (0.5%) | 0 (0.0%) | 0 (0.0%) |  |
| **Year of initiation of IFX treatment** | | | | | 0.0002 |
| 2015 | 119 (14.7%) | 9 (4.1%) | 24 (14.7%) | 1 (14.3%) |  |
| 2016 | 151 (18.7%) | 28 (12.8%) | 27 (16.6%) | 2 (28.6%) |  |
| 2017 | 276 (34.2%) | 81 (37.2%) | 63 (38.7%) | 1 (14.3%) |  |
| 2018 | 261 (32.3%) | 100 (45.9%) | 49 (30.1%) | 3 (42.9%) |  |
| **Time to first TDM (days)** | | | | | <0.0001 |
| Mean (SD) | 373.9 (341.83) | 250.9 (267.22) | 351.7 (322.85) | 319.1 (315.02) |  |
| **Year of first instance of TDM** | | | | | 0.0997 |
| 2015 | 0 (0.0%) | 0 (0.0%) | 0 (0.0%) | 0 (0.0%) |  |
| 2016 | 0 (0.0%) | 0 (0.0%) | 0 (0.0%) | 0 (0.0%) |  |
| 2017 | 266 (33.0%) | 60 (27.5%) | 60 (36.8%) | 3 (42.9%) |  |
| 2018 | 381 (47.2%) | 113 (51.8%) | 84 (51.5%) | 4 (57.1%) |  |
| 2019 | 160 (19.8%) | 45 (20.6%) | 19 (11.7%) | 0 (0.0%) |  |

TDM=therapeutic drug monitoring. P-value: categorical variables, chi-square test; numeric variables, ANOVA.

^†^Atlantic includes New Brunswick, Nova Scotia, Prince Edward Island, Newfoundland, and Labrador.

Dose optimization thresholds: High: A treatment interval decrease of ≥11 days (1.57 weeks) with a posterior interval of ≤35 days (5 weeks), and/or a dose level increase of ≥1.5 mg/kg with a posterior dose level of ≥9 mg/kg. Low: A treatment interval decrease of ≥11 days (1.57 weeks) with a posterior interval of ≤46 days (6.57 weeks), and/or dose level increase of ≥1.5 mg/kg with a posterior dose level of ≥7 mg/kg.

A=First TDM serum IFX concentration below threshold and no dose optimization within post-index period;

B=First TDM serum IFX concentration below threshold and dose optimization within post-index period;

C=First TDM serum IFX concentration at or above threshold and no dose optimization within post-index period;

D=First TDM serum IFX concentration at or above threshold and dose optimization within post-index period.

Suppl Table 5l: Subgroup Demographics and Baseline Characteristics of Patients with UC with no DO (Based on High Threshold Criteria) Prior to their First Instance of TDM (Serum IFX Threshold: 10 μg/mL; Post-Index Period for Dose Optimization: +17 weeks; Post-Index Dose Optimization Threshold: High)

|  | **A (N=746)** | **B (N=279)** | **C (N=157)** | **D (N=13)** | **p-value** |
| --- | --- | --- | --- | --- | --- |
| **Age (years)** | | | | | 0.0748 |
| Mean (SD) | 42.6 (16.12) | 43.0 (15.96) | 39.6 (13.56) | 36.9 (6.95) |  |
| Missing | 1 | 0 | 0 | 1 |  |
| **Age group (years)** | | | | | 0.1304 |
| 18 to 64 | 667 (89.5%) | 252 (90.3%) | 149 (94.9%) | 12 (100.0%) |  |
| 65 to 90 | 78 (10.5%) | 27 (9.7%) | 8 (5.1%) | 0 (0.0%) |  |
| Missing | 1 | 0 | 0 | 1 |  |
| **Gender** | | | | | 0.0740 |
| Female | 331 (44.4%) | 127 (45.5%) | 86 (54.8%) | 8 (61.5%) |  |
| Male | 415 (55.6%) | 152 (54.5%) | 71 (45.2%) | 5 (38.5%) |  |
| **First recorded weight** | | | | | 0.2339 |
| Mean (SD) | 75.92 (17.363) | 75.25 (16.848) | 76.48 (17.851) | 66.54 (14.715) |  |
| Missing | 2 | 0 | 0 | 0 |  |
| **Province/region of treating physician** | | | | | 0.0434 |
| Alberta | 43 (5.8%) | 21 (7.5%) | 6 (3.8%) | 0 (0.0%) |  |
| Atlantic^†^ | 115 (15.4%) | 24 (8.6%) | 22 (14.0%) | 2 (15.4%) |  |
| British Columbia | 101 (13.5%) | 21 (7.5%) | 18 (11.5%) | 3 (23.1%) |  |
| Ontario | 386 (51.7%) | 179 (64.2%) | 94 (59.9%) | 6 (46.2%) |  |
| Quebec | 98 (13.1%) | 33 (11.8%) | 17 (10.8%) | 2 (15.4%) |  |
| Saskatchewan/  Manitoba | 3 (0.4%) | 1 (0.4%) | 0 (0.0%) | 0 (0.0%) |  |
| **Year of initiation of IFX treatment** | | | | | <0.0001 |
| 2015 | 111 (14.9%) | 17 (6.1%) | 23 (14.6%) | 2 (15.4%) |  |
| 2016 | 146 (19.6%) | 33 (11.8%) | 27 (17.2%) | 2 (15.4%) |  |
| 2017 | 258 (34.6%) | 99 (35.5%) | 59 (37.6%) | 5 (38.5%) |  |
| 2018 | 231 (31.0%) | 130 (46.6%) | 48 (30.6%) | 4 (30.8%) |  |
| **Time to first TDM (days)** | | | | | <0.0001 |
| Mean (SD) | 383.2 (345.14) | 253.0 (268.79) | 354.7 (324.71) | 298.5 (288.94) |  |
| **Year of first instance of TDM** | | | | | 0.0602 |
| 2015 | 0 (0.0%) | 0 (0.0%) | 0 (0.0%) | 0 (0.0%) |  |
| 2016 | 0 (0.0%) | 0 (0.0%) | 0 (0.0%) | 0 (0.0%) |  |
| 2017 | 248 (33.2%) | 78 (28.0%) | 56 (35.7%) | 7 (53.8%) |  |
| 2018 | 352 (47.2%) | 142 (50.9%) | 83 (52.9%) | 5 (38.5%) |  |
| 2019 | 146 (19.6%) | 59 (21.1%) | 18 (11.5%) | 1 (7.7%) |  |

TDM=therapeutic drug monitoring. P-value: categorical variables, chi-square test; numeric variables, ANOVA.

^†^Atlantic includes New Brunswick, Nova Scotia, Prince Edward Island, Newfoundland, and Labrador.

Dose optimization thresholds: High: A treatment interval decrease of ≥11 days (1.57 weeks) with a posterior interval of ≤35 days (5 weeks), and/or a dose level increase of ≥1.5 mg/kg with a posterior dose level of ≥9 mg/kg. Low: A treatment interval decrease of ≥11 days (1.57 weeks) with a posterior interval of ≤46 days (6.57 weeks), and/or dose level increase of ≥1.5 mg/kg with a posterior dose level of ≥7 mg/kg.

A=First TDM serum IFX concentration below threshold and no dose optimization within post-index period;

B=First TDM serum IFX concentration below threshold and dose optimization within post-index period;

C=First TDM serum IFX concentration at or above threshold and no dose optimization within post-index period;

D=First TDM serum IFX concentration at or above threshold and dose optimization within post-index period.

Suppl Table 6a: Subgroup Demographics and Baseline Characteristics of Patients with CD with a DO (Based on Low Threshold Criteria) Prior to their First Instance of TDM (Serum IFX Threshold: 3 μg/mL; Post-Index Period for Dose Optimization: +9 weeks; Post-Index Dose Optimization Threshold: Low)

|  | **A (N=109)** | **B (N=56)** | **C (N=330)** | **D (N=40)** | **p-value** |
| --- | --- | --- | --- | --- | --- |
| **Age (years)** | | | | | 0.0683 |
| Mean (SD) | 43.6 (16.64) | 48.1 (17.43) | 42.6 (15.13) | 40.8 (14.53) |  |
| **Age group (years)** | | | | | 0.0814 |
| 18 to 64 | 94 (86.2%) | 44 (78.6%) | 296 (89.7%) | 37 (92.5%) |  |
| 65 to 90 | 15 (13.8%) | 12 (21.4%) | 34 (10.3%) | 3 (7.5%) |  |
| **Gender** | | | | | 0.0817 |
| Female | 41 (37.6%) | 26 (46.4%) | 170 (51.5%) | 21 (52.5%) |  |
| Male | 68 (62.4%) | 30 (53.6%) | 160 (48.5%) | 19 (47.5%) |  |
| **First recorded weight** | | | | | 0.1721 |
| Mean (SD) | 77.49 (21.010) | 72.06 (17.956) | 73.65 (16.742) | 73.00 (16.775) |  |
| **Province/region of treating physician** | | | | | 0.0543 |
| Alberta | 7 (6.4%) | 1 (1.8%) | 17 (5.2%) | 2 (5.0%) |  |
| Atlantic^†^ | 19 (17.4%) | 3 (5.4%) | 58 (17.6%) | 8 (20.0%) |  |
| British Columbia | 6 (5.5%) | 2 (3.6%) | 41 (12.4%) | 6 (15.0%) |  |
| Ontario | 42 (38.5%) | 33 (58.9%) | 135 (40.9%) | 17 (42.5%) |  |
| Quebec | 35 (32.1%) | 17 (30.4%) | 75 (22.7%) | 7 (17.5%) |  |
| Saskatchewan/  Manitoba | 0 (0.0%) | 0 (0.0%) | 4 (1.2%) | 0 (0.0%) |  |
| **Year of initiation of IFX treatment** | | | | | 0.1548 |
| 2015 | 22 (20.2%) | 17 (30.4%) | 117 (35.5%) | 11 (27.5%) |  |
| 2016 | 33 (30.3%) | 18 (32.1%) | 82 (24.8%) | 12 (30.0%) |  |
| 2017 | 32 (29.4%) | 9 (16.1%) | 74 (22.4%) | 12 (30.0%) |  |
| 2018 | 22 (20.2%) | 12 (21.4%) | 57 (17.3%) | 5 (12.5%) |  |
| **Time to first TDM (days)** | | | | | 0.6215 |
| Mean (SD) | 526.4 (368.56) | 551.3 (437.23) | 573.4 (382.54) | 515.9 (350.13) |  |
| **Year of first instance of TDM** | | | | | 0.1133 |
| 2015 | 0 (0.0%) | 0 (0.0%) | 0 (0.0%) | 0 (0.0%) |  |
| 2016 | 0 (0.0%) | 0 (0.0%) | 0 (0.0%) | 0 (0.0%) |  |
| 2017 | 35 (32.1%) | 20 (35.7%) | 152 (46.1%) | 16 (40.0%) |  |
| 2018 | 46 (42.2%) | 23 (41.1%) | 120 (36.4%) | 19 (47.5%) |  |
| 2019 | 28 (25.7%) | 13 (23.2%) | 58 (17.6%) | 5 (12.5%) |  |

TDM=therapeutic drug monitoring. P-value: categorical variables, chi-square test; numeric variables, ANOVA.

^†^Atlantic includes New Brunswick, Nova Scotia, Prince Edward Island, Newfoundland, and Labrador.

Dose optimization thresholds: High: A treatment interval decrease of ≥11 days (1.57 weeks) with a posterior interval of ≤35 days (5 weeks), and/or a dose level increase of ≥1.5 mg/kg with a posterior dose level of ≥9 mg/kg. Low: A treatment interval decrease of ≥11 days (1.57 weeks) with a posterior interval of ≤46 days (6.57 weeks), and/or dose level increase of ≥1.5 mg/kg with a posterior dose level of ≥7 mg/kg.

A=First TDM serum IFX concentration below threshold and no dose optimization within post-index period;

B=First TDM serum IFX concentration below threshold and dose optimization within post-index period;

C=First TDM serum IFX concentration at or above threshold and no dose optimization within post-index period;

D=First TDM serum IFX concentration at or above threshold and dose optimization within post-index period.

Suppl Table 6b: Subgroup Demographics and Baseline Characteristics of Patients with CD with a DO (Based on Low Threshold Criteria) Prior to their First Instance of TDM (Serum IFX Threshold: 3 μg/mL; Post-Index Period for Dose Optimization: +17 weeks; Post-Index Dose Optimization Threshold: Low)

|  | **A (N=89)** | **B (N=76)** | **C (N=303)** | **D (N=67)** | **p-value** |
| --- | --- | --- | --- | --- | --- |
| **Age (years)** | | | | | 0.1265 |
| Mean (SD) | 43.6 (17.16) | 46.9 (16.74) | 42.2 (15.08) | 43.3 (15.06) |  |
| **Age group (years)** | | | | | 0.1690 |
| 18 to 64 | 76 (85.4%) | 62 (81.6%) | 272 (89.8%) | 61 (91.0%) |  |
| 65 to 90 | 13 (14.6%) | 14 (18.4%) | 31 (10.2%) | 6 (9.0%) |  |
| **Gender** | | | | | 0.0359 |
| Female | 31 (34.8%) | 36 (47.4%) | 154 (50.8%) | 37 (55.2%) |  |
| Male | 58 (65.2%) | 40 (52.6%) | 149 (49.2%) | 30 (44.8%) |  |
| **First recorded weight** | | | | | 0.1321 |
| Mean (SD) | 77.47 (19.583) | 73.50 (20.689) | 74.20 (16.946) | 70.75 (15.489) |  |
| **Province/region of treating physician** | | | | | 0.0719 |
| Alberta | 6 (6.7%) | 2 (2.6%) | 17 (5.6%) | 2 (3.0%) |  |
| Atlantic^†^ | 14 (15.7%) | 8 (10.5%) | 52 (17.2%) | 14 (20.9%) |  |
| British Columbia | 6 (6.7%) | 2 (2.6%) | 36 (11.9%) | 11 (16.4%) |  |
| Ontario | 34 (38.2%) | 41 (53.9%) | 125 (41.3%) | 27 (40.3%) |  |
| Quebec | 29 (32.6%) | 23 (30.3%) | 69 (22.8%) | 13 (19.4%) |  |
| Saskatchewan/  Manitoba | 0 (0.0%) | 0 (0.0%) | 4 (1.3%) | 0 (0.0%) |  |
| **Year of initiation of IFX treatment** | | | | | 0.2094 |
| 2015 | 18 (20.2%) | 21 (27.6%) | 107 (35.3%) | 21 (31.3%) |  |
| 2016 | 26 (29.2%) | 25 (32.9%) | 77 (25.4%) | 17 (25.4%) |  |
| 2017 | 28 (31.5%) | 13 (17.1%) | 70 (23.1%) | 16 (23.9%) |  |
| 2018 | 17 (19.1%) | 17 (22.4%) | 49 (16.2%) | 13 (19.4%) |  |
| **Time to first TDM (days)** | | | | | 0.8150 |
| Mean (SD) | 530.1 (366.88) | 540.4 (422.03) | 570.2 (372.44) | 553.6 (410.83) |  |
| **Year of first instance of TDM** | | | | | 0.1271 |
| 2015 | 0 (0.0%) | 0 (0.0%) | 0 (0.0%) | 0 (0.0%) |  |
| 2016 | 0 (0.0%) | 0 (0.0%) | 0 (0.0%) | 0 (0.0%) |  |
| 2017 | 28 (31.5%) | 27 (35.5%) | 142 (46.9%) | 26 (38.8%) |  |
| 2018 | 39 (43.8%) | 30 (39.5%) | 111 (36.6%) | 28 (41.8%) |  |
| 2019 | 22 (24.7%) | 19 (25.0%) | 50 (16.5%) | 13 (19.4%) |  |

TDM=therapeutic drug monitoring. P-value: categorical variables, chi-square test; numeric variables, ANOVA.

^†^Atlantic includes New Brunswick, Nova Scotia, Prince Edward Island, Newfoundland, and Labrador.

Dose optimization thresholds: High: A treatment interval decrease of ≥11 days (1.57 weeks) with a posterior interval of ≤35 days (5 weeks), and/or a dose level increase of ≥1.5 mg/kg with a posterior dose level of ≥9 mg/kg. Low: A treatment interval decrease of ≥11 days (1.57 weeks) with a posterior interval of ≤46 days (6.57 weeks), and/or dose level increase of ≥1.5 mg/kg with a posterior dose level of ≥7 mg/kg.

A=First TDM serum IFX concentration below threshold and no dose optimization within post-index period;

B=First TDM serum IFX concentration below threshold and dose optimization within post-index period;

C=First TDM serum IFX concentration at or above threshold and no dose optimization within post-index period;

D=First TDM serum IFX concentration at or above threshold and dose optimization within post-index period.

Suppl Table 6c: Subgroup Demographics and Baseline Characteristics of Patients with CD with a DO (Based on Low Threshold Criteria) Prior to their First Instance of TDM (Serum IFX Threshold: 5 μg/mL; Post-Index Period for Dose Optimization: +9 weeks; Post-Index Dose Optimization Threshold: Low)

|  | **A (N=168)** | **B (N=73)** | **C (N=271)** | **D (N=23)** | **p-value** |
| --- | --- | --- | --- | --- | --- |
| **Age (years)** | | | | | 0.0715 |
| Mean (SD) | 44.4 (16.24) | 46.6 (17.70) | 41.9 (14.99) | 40.3 (11.60) |  |
| **Age group (years)** | | | | | 0.0116 |
| 18 to 64 | 144 (85.7%) | 58 (79.5%) | 246 (90.8%) | 23 (100.0%) |  |
| 65 to 90 | 24 (14.3%) | 15 (20.5%) | 25 (9.2%) | 0 (0.0%) |  |
| **Gender** | | | | | 0.2990 |
| Female | 72 (42.9%) | 34 (46.6%) | 139 (51.3%) | 13 (56.5%) |  |
| Male | 96 (57.1%) | 39 (53.4%) | 132 (48.7%) | 10 (43.5%) |  |
| **First recorded weight** | | | | | 0.5113 |
| Mean (SD) | 75.63 (19.299) | 71.93 (18.073) | 73.96 (17.063) | 74.11 (15.264) |  |
| **Province/region of treating physician** | | | | | 0.0010 |
| Alberta | 11 (6.5%) | 2 (2.7%) | 13 (4.8%) | 1 (4.3%) |  |
| Atlantic^†^ | 26 (15.5%) | 6 (8.2%) | 51 (18.8%) | 5 (21.7%) |  |
| British Columbia | 8 (4.8%) | 3 (4.1%) | 39 (14.4%) | 5 (21.7%) |  |
| Ontario | 67 (39.9%) | 42 (57.5%) | 110 (40.6%) | 8 (34.8%) |  |
| Quebec | 56 (33.3%) | 20 (27.4%) | 54 (19.9%) | 4 (17.4%) |  |
| Saskatchewan/  Manitoba | 0 (0.0%) | 0 (0.0%) | 4 (1.5%) | 0 (0.0%) |  |
| **Year of initiation of IFX treatment** | | | | | 0.2840 |
| 2015 | 41 (24.4%) | 21 (28.8%) | 98 (36.2%) | 7 (30.4%) |  |
| 2016 | 49 (29.2%) | 21 (28.8%) | 66 (24.4%) | 9 (39.1%) |  |
| 2017 | 48 (28.6%) | 16 (21.9%) | 58 (21.4%) | 5 (21.7%) |  |
| 2018 | 30 (17.9%) | 15 (20.5%) | 49 (18.1%) | 2 (8.7%) |  |
| **Time to first TDM (days)** | | | | | 0.5462 |
| Mean (SD) | 550.5 (377.19) | 509.1 (413.43) | 568.7 (381.05) | 623.5 (356.07) |  |
| **Year of first instance of TDM** | | | | | 0.1094 |
| 2015 | 0 (0.0%) | 0 (0.0%) | 0 (0.0%) | 0 (0.0%) |  |
| 2016 | 0 (0.0%) | 0 (0.0%) | 0 (0.0%) | 0 (0.0%) |  |
| 2017 | 60 (35.7%) | 30 (41.1%) | 127 (46.9%) | 6 (26.1%) |  |
| 2018 | 70 (41.7%) | 28 (38.4%) | 96 (35.4%) | 14 (60.9%) |  |
| 2019 | 38 (22.6%) | 15 (20.5%) | 48 (17.7%) | 3 (13.0%) |  |

TDM=therapeutic drug monitoring. P-value: categorical variables, chi-square test; numeric variables, ANOVA.

^†^Atlantic includes New Brunswick, Nova Scotia, Prince Edward Island, Newfoundland, and Labrador.

Dose optimization thresholds: High: A treatment interval decrease of ≥11 days (1.57 weeks) with a posterior interval of ≤35 days (5 weeks), and/or a dose level increase of ≥1.5 mg/kg with a posterior dose level of ≥9 mg/kg. Low: A treatment interval decrease of ≥11 days (1.57 weeks) with a posterior interval of ≤46 days (6.57 weeks), and/or dose level increase of ≥1.5 mg/kg with a posterior dose level of ≥7 mg/kg.

A=First TDM serum IFX concentration below threshold and no dose optimization within post-index period;

B=First TDM serum IFX concentration below threshold and dose optimization within post-index period;

C=First TDM serum IFX concentration at or above threshold and no dose optimization within post-index period;

D=First TDM serum IFX concentration at or above threshold and dose optimization within post-index period.

Suppl Table 6d: Subgroup Demographics and Baseline Characteristics of Patients with CD with a DO (Based on Low Threshold Criteria) Prior to their First Instance of TDM (Serum IFX Threshold: 5 μg/mL; Post-Index Period for Dose Optimization: +17 weeks; Post-Index Dose Optimization Threshold: Low)

|  | **A (N=139)** | **B (N=102)** | **C (N=253)** | **D (N=41)** | **p-value** |
| --- | --- | --- | --- | --- | --- |
| **Age (years)** | | | | | 0.0701 |
| Mean (SD) | 44.1 (16.49) | 46.2 (16.97) | 41.6 (14.99) | 42.8 (13.23) |  |
| **Age group (years)** | | | | | 0.0389 |
| 18 to 64 | 118 (84.9%) | 84 (82.4%) | 230 (90.9%) | 39 (95.1%) |  |
| 65 to 90 | 21 (15.1%) | 18 (17.6%) | 23 (9.1%) | 2 (4.9%) |  |
| **Gender** | | | | | 0.1495 |
| Female | 56 (40.3%) | 50 (49.0%) | 129 (51.0%) | 23 (56.1%) |  |
| Male | 83 (59.7%) | 52 (51.0%) | 124 (49.0%) | 18 (43.9%) |  |
| **First recorded weight** | | | | | 0.3221 |
| Mean (SD) | 76.19 (17.995) | 72.22 (20.101) | 74.26 (17.388) | 72.19 (13.598) |  |
| **Province/region of treating physician** | | | | | 0.0006 |
| Alberta | 10 (7.2%) | 3 (2.9%) | 13 (5.1%) | 1 (2.4%) |  |
| Atlantic^†^ | 19 (13.7%) | 13 (12.7%) | 47 (18.6%) | 9 (22.0%) |  |
| British Columbia | 8 (5.8%) | 3 (2.9%) | 34 (13.4%) | 10 (24.4%) |  |
| Ontario | 57 (41.0%) | 52 (51.0%) | 102 (40.3%) | 16 (39.0%) |  |
| Quebec | 45 (32.4%) | 31 (30.4%) | 53 (20.9%) | 5 (12.2%) |  |
| Saskatchewan/  Manitoba | 0 (0.0%) | 0 (0.0%) | 4 (1.6%) | 0 (0.0%) |  |
| **Year of initiation of IFX treatment** | | | | | 0.3148 |
| 2015 | 35 (25.2%) | 27 (26.5%) | 90 (35.6%) | 15 (36.6%) |  |
| 2016 | 40 (28.8%) | 30 (29.4%) | 63 (24.9%) | 12 (29.3%) |  |
| 2017 | 42 (30.2%) | 22 (21.6%) | 56 (22.1%) | 7 (17.1%) |  |
| 2018 | 22 (15.8%) | 23 (22.5%) | 44 (17.4%) | 7 (17.1%) |  |
| **Time to first TDM (days)** | | | | | 0.2722 |
| Mean (SD) | 560.5 (371.60) | 507.2 (409.43) | 561.4 (371.56) | 644.5 (418.98) |  |
| **Year of first instance of TDM** | | | | | 0.2042 |
| 2015 | 0 (0.0%) | 0 (0.0%) | 0 (0.0%) | 0 (0.0%) |  |
| 2016 | 0 (0.0%) | 0 (0.0%) | 0 (0.0%) | 0 (0.0%) |  |
| 2017 | 50 (36.0%) | 40 (39.2%) | 120 (47.4%) | 13 (31.7%) |  |
| 2018 | 60 (43.2%) | 38 (37.3%) | 90 (35.6%) | 20 (48.8%) |  |
| 2019 | 29 (20.9%) | 24 (23.5%) | 43 (17.0%) | 8 (19.5%) |  |

TDM=therapeutic drug monitoring. P-value: categorical variables, chi-square test; numeric variables, ANOVA.

^†^Atlantic includes New Brunswick, Nova Scotia, Prince Edward Island, Newfoundland, and Labrador.

Dose optimization thresholds: High: A treatment interval decrease of ≥11 days (1.57 weeks) with a posterior interval of ≤35 days (5 weeks), and/or a dose level increase of ≥1.5 mg/kg with a posterior dose level of ≥9 mg/kg. Low: A treatment interval decrease of ≥11 days (1.57 weeks) with a posterior interval of ≤46 days (6.57 weeks), and/or dose level increase of ≥1.5 mg/kg with a posterior dose level of ≥7 mg/kg.

A=First TDM serum IFX concentration below threshold and no dose optimization within post-index period;

B=First TDM serum IFX concentration below threshold and dose optimization within post-index period;

C=First TDM serum IFX concentration at or above threshold and no dose optimization within post-index period;

D=First TDM serum IFX concentration at or above threshold and dose optimization within post-index period.

Suppl Table 6e: Subgroup Demographics and Baseline Characteristics of Patients with CD with a DO (Based on Low Threshold Criteria) Prior to their First Instance of TDM (Serum IFX Threshold: 10 μg/mL; Post-Index Period for Dose Optimization: +9 weeks; Post-Index Dose Optimization Threshold: Low)

|  | **A (N=275)** | **B (N=86)** | **C (N=164)** | **D (N=10)** | **p-value** |
| --- | --- | --- | --- | --- | --- |
| **Age (years)** | | | | | 0.0444 |
| Mean (SD) | 44.2 (15.74) | 45.7 (17.12) | 40.6 (14.89) | 40.2 (10.65) |  |
| **Age group (years)** | | | | | 0.0292 |
| 18 to 64 | 237 (86.2%) | 71 (82.6%) | 153 (93.3%) | 10 (100.0%) |  |
| 65 to 90 | 38 (13.8%) | 15 (17.4%) | 11 (6.7%) | 0 (0.0%) |  |
| **Gender** | | | | | 0.3460 |
| Female | 123 (44.7%) | 42 (48.8%) | 88 (53.7%) | 5 (50.0%) |  |
| Male | 152 (55.3%) | 44 (51.2%) | 76 (46.3%) | 5 (50.0%) |  |
| **First recorded weight** | | | | | 0.4052 |
| Mean (SD) | 75.33 (18.423) | 71.99 (17.315) | 73.37 (17.109) | 76.40 (18.470) |  |
| **Province/region of treating physician** | | | | | 0.0002 |
| Alberta | 13 (4.7%) | 2 (2.3%) | 11 (6.7%) | 1 (10.0%) |  |
| Atlantic^†^ | 48 (17.5%) | 9 (10.5%) | 29 (17.7%) | 2 (20.0%) |  |
| British Columbia | 18 (6.5%) | 4 (4.7%) | 29 (17.7%) | 4 (40.0%) |  |
| Ontario | 113 (41.1%) | 47 (54.7%) | 64 (39.0%) | 3 (30.0%) |  |
| Quebec | 81 (29.5%) | 24 (27.9%) | 29 (17.7%) | 0 (0.0%) |  |
| Saskatchewan/  Manitoba | 2 (0.7%) | 0 (0.0%) | 2 (1.2%) | 0 (0.0%) |  |
| **Year of initiation of IFX treatment** | | | | | 0.8511 |
| 2015 | 83 (30.2%) | 25 (29.1%) | 56 (34.1%) | 3 (30.0%) |  |
| 2016 | 77 (28.0%) | 26 (30.2%) | 38 (23.2%) | 4 (40.0%) |  |
| 2017 | 70 (25.5%) | 19 (22.1%) | 36 (22.0%) | 2 (20.0%) |  |
| 2018 | 45 (16.4%) | 16 (18.6%) | 34 (20.7%) | 1 (10.0%) |  |
| **Time to first TDM (days)** | | | | | 0.5143 |
| Mean (SD) | 581.3 (390.22) | 534.3 (417.19) | 528.8 (358.87) | 555.3 (241.95) |  |
| **Year of first instance of TDM** | | | | | 0.5575 |
| 2015 | 0 (0.0%) | 0 (0.0%) | 0 (0.0%) | 0 (0.0%) |  |
| 2016 | 0 (0.0%) | 0 (0.0%) | 0 (0.0%) | 0 (0.0%) |  |
| 2017 | 109 (39.6%) | 32 (37.2%) | 78 (47.6%) | 4 (40.0%) |  |
| 2018 | 107 (38.9%) | 37 (43.0%) | 59 (36.0%) | 5 (50.0%) |  |
| 2019 | 59 (21.5%) | 17 (19.8%) | 27 (16.5%) | 1 (10.0%) |  |

TDM=therapeutic drug monitoring. P-value: categorical variables, chi-square test; numeric variables, ANOVA.

^†^Atlantic includes New Brunswick, Nova Scotia, Prince Edward Island, Newfoundland, and Labrador.

Dose optimization thresholds: High: A treatment interval decrease of ≥11 days (1.57 weeks) with a posterior interval of ≤35 days (5 weeks), and/or a dose level increase of ≥1.5 mg/kg with a posterior dose level of ≥9 mg/kg. Low: A treatment interval decrease of ≥11 days (1.57 weeks) with a posterior interval of ≤46 days (6.57 weeks), and/or dose level increase of ≥1.5 mg/kg with a posterior dose level of ≥7 mg/kg.

A=First TDM serum IFX concentration below threshold and no dose optimization within post-index period;

B=First TDM serum IFX concentration below threshold and dose optimization within post-index period;

C=First TDM serum IFX concentration at or above threshold and no dose optimization within post-index period;

D=First TDM serum IFX concentration at or above threshold and dose optimization within post-index period.

Suppl Table 6f: Subgroup Demographics and Baseline Characteristics of Patients with CD with a DO (Based on Low Threshold Criteria) Prior to their First Instance of TDM (Serum IFX Threshold: 10 μg/mL; Post-Index Period for Dose Optimization: +17 weeks; Post-Index Dose Optimization Threshold: Low)

|  | **A (N=239)** | **B (N=122)** | **C (N=153)** | **D (N=21)** | **p-value** |
| --- | --- | --- | --- | --- | --- |
| **Age (years)** | | | | | 0.0271 |
| Mean (SD) | 44.0 (15.94) | 45.5 (16.34) | 40.1 (14.70) | 43.6 (14.29) |  |
| **Age group (years)** | | | | | 0.0463 |
| 18 to 64 | 205 (85.8%) | 103 (84.4%) | 143 (93.5%) | 20 (95.2%) |  |
| 65 to 90 | 34 (14.2%) | 19 (15.6%) | 10 (6.5%) | 1 (4.8%) |  |
| **Gender** | | | | | 0.1249 |
| Female | 102 (42.7%) | 63 (51.6%) | 83 (54.2%) | 10 (47.6%) |  |
| Male | 137 (57.3%) | 59 (48.4%) | 70 (45.8%) | 11 (52.4%) |  |
| **First recorded weight** | | | | | 0.3062 |
| Mean (SD) | 75.72 (17.740) | 72.20 (18.920) | 73.73 (17.385) | 72.26 (15.637) |  |
| **Province/region of treating physician** | | | | | 0.0005 |
| Alberta | 12 (5.0%) | 3 (2.5%) | 11 (7.2%) | 1 (4.8%) |  |
| Atlantic^†^ | 39 (16.3%) | 18 (14.8%) | 27 (17.6%) | 4 (19.0%) |  |
| British Columbia | 16 (6.7%) | 6 (4.9%) | 26 (17.0%) | 7 (33.3%) |  |
| Ontario | 100 (41.8%) | 60 (49.2%) | 59 (38.6%) | 8 (38.1%) |  |
| Quebec | 70 (29.3%) | 35 (28.7%) | 28 (18.3%) | 1 (4.8%) |  |
| Saskatchewan/  Manitoba | 2 (0.8%) | 0 (0.0%) | 2 (1.3%) | 0 (0.0%) |  |
| **Year of initiation of IFX treatment** | | | | | 0.7343 |
| 2015 | 74 (31.0%) | 34 (27.9%) | 51 (33.3%) | 8 (38.1%) |  |
| 2016 | 66 (27.6%) | 37 (30.3%) | 37 (24.2%) | 5 (23.8%) |  |
| 2017 | 63 (26.4%) | 26 (21.3%) | 35 (22.9%) | 3 (14.3%) |  |
| 2018 | 36 (15.1%) | 25 (20.5%) | 30 (19.6%) | 5 (23.8%) |  |
| **Time to first TDM (days)** | | | | | 0.3298 |
| Mean (SD) | 586.9 (385.52) | 537.2 (417.48) | 520.7 (344.74) | 600.9 (408.82) |  |
| **Year of first instance of TDM** | | | | | 0.5481 |
| 2015 | 0 (0.0%) | 0 (0.0%) | 0 (0.0%) | 0 (0.0%) |  |
| 2016 | 0 (0.0%) | 0 (0.0%) | 0 (0.0%) | 0 (0.0%) |  |
| 2017 | 96 (40.2%) | 45 (36.9%) | 74 (48.4%) | 8 (38.1%) |  |
| 2018 | 95 (39.7%) | 49 (40.2%) | 55 (35.9%) | 9 (42.9%) |  |
| 2019 | 48 (20.1%) | 28 (23.0%) | 24 (15.7%) | 4 (19.0%) |  |

TDM=therapeutic drug monitoring. P-value: categorical variables, chi-square test; numeric variables, ANOVA.

^†^Atlantic includes New Brunswick, Nova Scotia, Prince Edward Island, Newfoundland, and Labrador.

Dose optimization thresholds: High: A treatment interval decrease of ≥11 days (1.57 weeks) with a posterior interval of ≤35 days (5 weeks), and/or a dose level increase of ≥1.5 mg/kg with a posterior dose level of ≥9 mg/kg. Low: A treatment interval decrease of ≥11 days (1.57 weeks) with a posterior interval of ≤46 days (6.57 weeks), and/or dose level increase of ≥1.5 mg/kg with a posterior dose level of ≥7 mg/kg.

A=First TDM serum IFX concentration below threshold and no dose optimization within post-index period;

B=First TDM serum IFX concentration below threshold and dose optimization within post-index period;

C=First TDM serum IFX concentration at or above threshold and no dose optimization within post-index period;

D=First TDM serum IFX concentration at or above threshold and dose optimization within post-index period.

Suppl Table 6g: Subgroup Demographics and Baseline Characteristics of Patients with CD with a DO (Based on Low Threshold Criteria) Prior to their First Instance of TDM (Serum IFX Threshold: 3 μg/mL; Post-Index Period for Dose Optimization: +9 weeks; Post-Index Dose Optimization Threshold: High)

|  | **A (N=126)** | **B (N=39)** | **C (N=346)** | **D (N=24)** | **p-value** |
| --- | --- | --- | --- | --- | --- |
| **Age (years)** | | | | | 0.0974 |
| Mean (SD) | 44.3 (16.93) | 48.0 (17.11) | 42.6 (15.03) | 39.2 (15.50) |  |
| **Age group (years)** | | | | | 0.1517 |
| 18 to 64 | 107 (84.9%) | 31 (79.5%) | 311 (89.9%) | 22 (91.7%) |  |
| 65 to 90 | 19 (15.1%) | 8 (20.5%) | 35 (10.1%) | 2 (8.3%) |  |
| **Gender** | | | | | 0.0173 |
| Female | 46 (36.5%) | 21 (53.8%) | 181 (52.3%) | 10 (41.7%) |  |
| Male | 80 (63.5%) | 18 (46.2%) | 165 (47.7%) | 14 (58.3%) |  |
| **First recorded weight** | | | | | 0.3430 |
| Mean (SD) | 76.67 (21.500) | 72.32 (14.649) | 73.62 (16.686) | 72.87 (17.618) |  |
| **Province/region of treating physician** | | | | | 0.0482 |
| Alberta | 7 (5.6%) | 1 (2.6%) | 17 (4.9%) | 2 (8.3%) |  |
| Atlantic^†^ | 21 (16.7%) | 1 (2.6%) | 62 (17.9%) | 4 (16.7%) |  |
| British Columbia | 7 (5.6%) | 1 (2.6%) | 43 (12.4%) | 4 (16.7%) |  |
| Ontario | 50 (39.7%) | 25 (64.1%) | 144 (41.6%) | 8 (33.3%) |  |
| Quebec | 41 (32.5%) | 11 (28.2%) | 76 (22.0%) | 6 (25.0%) |  |
| Saskatchewan/  Manitoba | 0 (0.0%) | 0 (0.0%) | 4 (1.2%) | 0 (0.0%) |  |
| **Year of initiation of IFX treatment** | | | | | 0.2470 |
| 2015 | 27 (21.4%) | 12 (30.8%) | 120 (34.7%) | 8 (33.3%) |  |
| 2016 | 42 (33.3%) | 9 (23.1%) | 88 (25.4%) | 6 (25.0%) |  |
| 2017 | 34 (27.0%) | 7 (17.9%) | 80 (23.1%) | 6 (25.0%) |  |
| 2018 | 23 (18.3%) | 11 (28.2%) | 58 (16.8%) | 4 (16.7%) |  |
| **Time to first TDM (days)** | | | | | 0.3660 |
| Mean (SD) | 541.3 (379.36) | 513.9 (435.17) | 575.0 (381.42) | 454.8 (331.63) |  |
| **Year of first instance of TDM** | | | | | 0.1370 |
| 2015 | 0 (0.0%) | 0 (0.0%) | 0 (0.0%) | 0 (0.0%) |  |
| 2016 | 0 (0.0%) | 0 (0.0%) | 0 (0.0%) | 0 (0.0%) |  |
| 2017 | 43 (34.1%) | 12 (30.8%) | 155 (44.8%) | 13 (54.2%) |  |
| 2018 | 52 (41.3%) | 17 (43.6%) | 130 (37.6%) | 9 (37.5%) |  |
| 2019 | 31 (24.6%) | 10 (25.6%) | 61 (17.6%) | 2 (8.3%) |  |

TDM=therapeutic drug monitoring. P-value: categorical variables, chi-square test; numeric variables, ANOVA.

^†^Atlantic includes New Brunswick, Nova Scotia, Prince Edward Island, Newfoundland, and Labrador.

Dose optimization thresholds: High: A treatment interval decrease of ≥11 days (1.57 weeks) with a posterior interval of ≤35 days (5 weeks), and/or a dose level increase of ≥1.5 mg/kg with a posterior dose level of ≥9 mg/kg. Low: A treatment interval decrease of ≥11 days (1.57 weeks) with a posterior interval of ≤46 days (6.57 weeks), and/or dose level increase of ≥1.5 mg/kg with a posterior dose level of ≥7 mg/kg.

A=First TDM serum IFX concentration below threshold and no dose optimization within post-index period;

B=First TDM serum IFX concentration below threshold and dose optimization within post-index period;

C=First TDM serum IFX concentration at or above threshold and no dose optimization within post-index period;

D=First TDM serum IFX concentration at or above threshold and dose optimization within post-index period.

Suppl Table 6h: Subgroup Demographics and Baseline Characteristics of Patients with CD with a DO (Based on Low Threshold Criteria) Prior to their First Instance of TDM (Serum IFX Threshold: 3 μg/mL; Post-Index Period for Dose Optimization: +17 weeks; Post-Index Dose Optimization Threshold: High)

|  | **A (N=112)** | **B (N=53)** | **C (N=330)** | **D (N=40)** | **p-value** |
| --- | --- | --- | --- | --- | --- |
| **Age (years)** | | | | | 0.1517 |
| Mean (SD) | 44.1 (17.18) | 47.5 (16.54) | 42.4 (14.91) | 42.0 (16.42) |  |
| **Age group (years)** | | | | | 0.1633 |
| 18 to 64 | 95 (84.8%) | 43 (81.1%) | 298 (90.3%) | 35 (87.5%) |  |
| 65 to 90 | 17 (15.2%) | 10 (18.9%) | 32 (9.7%) | 5 (12.5%) |  |
| **Gender** | | | | | 0.0117 |
| Female | 39 (34.8%) | 28 (52.8%) | 173 (52.4%) | 18 (45.0%) |  |
| Male | 73 (65.2%) | 25 (47.2%) | 157 (47.6%) | 22 (55.0%) |  |
| **First recorded weight** | | | | | 0.2484 |
| Mean (SD) | 76.74 (20.468) | 73.33 (19.403) | 73.94 (16.701) | 70.56 (16.824) |  |
| **Province/region of treating physician** | | | | | 0.0094 |
| Alberta | 7 (6.3%) | 1 (1.9%) | 17 (5.2%) | 2 (5.0%) |  |
| Atlantic^†^ | 19 (17.0%) | 3 (5.7%) | 58 (17.6%) | 8 (20.0%) |  |
| British Columbia | 7 (6.3%) | 1 (1.9%) | 38 (11.5%) | 9 (22.5%) |  |
| Ontario | 42 (37.5%) | 33 (62.3%) | 139 (42.1%) | 13 (32.5%) |  |
| Quebec | 37 (33.0%) | 15 (28.3%) | 74 (22.4%) | 8 (20.0%) |  |
| Saskatchewan/  Manitoba | 0 (0.0%) | 0 (0.0%) | 4 (1.2%) | 0 (0.0%) |  |
| **Year of initiation of IFX treatment** | | | | | 0.1284 |
| 2015 | 24 (21.4%) | 15 (28.3%) | 117 (35.5%) | 11 (27.5%) |  |
| 2016 | 38 (33.9%) | 13 (24.5%) | 83 (25.2%) | 11 (27.5%) |  |
| 2017 | 31 (27.7%) | 10 (18.9%) | 77 (23.3%) | 9 (22.5%) |  |
| 2018 | 19 (17.0%) | 15 (28.3%) | 53 (16.1%) | 9 (22.5%) |  |
| **Time to first TDM (days)** | | | | | 0.2974 |
| Mean (SD) | 548.4 (383.09) | 506.2 (412.61) | 578.0 (378.52) | 477.8 (377.17) |  |
| **Year of first instance of TDM** | | | | | 0.2113 |
| 2015 | 0 (0.0%) | 0 (0.0%) | 0 (0.0%) | 0 (0.0%) |  |
| 2016 | 0 (0.0%) | 0 (0.0%) | 0 (0.0%) | 0 (0.0%) |  |
| 2017 | 38 (33.9%) | 17 (32.1%) | 149 (45.2%) | 19 (47.5%) |  |
| 2018 | 47 (42.0%) | 22 (41.5%) | 124 (37.6%) | 15 (37.5%) |  |
| 2019 | 27 (24.1%) | 14 (26.4%) | 57 (17.3%) | 6 (15.0%) |  |

TDM=therapeutic drug monitoring. P-value: categorical variables, chi-square test; numeric variables, ANOVA.

^†^Atlantic includes New Brunswick, Nova Scotia, Prince Edward Island, Newfoundland, and Labrador.

Dose optimization thresholds: High: A treatment interval decrease of ≥11 days (1.57 weeks) with a posterior interval of ≤35 days (5 weeks), and/or a dose level increase of ≥1.5 mg/kg with a posterior dose level of ≥9 mg/kg. Low: A treatment interval decrease of ≥11 days (1.57 weeks) with a posterior interval of ≤46 days (6.57 weeks), and/or dose level increase of ≥1.5 mg/kg with a posterior dose level of ≥7 mg/kg.

A=First TDM serum IFX concentration below threshold and no dose optimization within post-index period;

B=First TDM serum IFX concentration below threshold and dose optimization within post-index period;

C=First TDM serum IFX concentration at or above threshold and no dose optimization within post-index period;

D=First TDM serum IFX concentration at or above threshold and dose optimization within post-index period.

Suppl Table 6i: Subgroup Demographics and Baseline Characteristics of Patients with CD with a DO (Based on Low Threshold Criteria) Prior to their First Instance of TDM (Serum IFX Threshold: 5 μg/mL; Post-Index Period for Dose Optimization: +9 weeks; Post-Index Dose Optimization Threshold: High)

|  | **A (N=188)** | **B (N=53)** | **C (N=284)** | **D (N=10)** | **p-value** |
| --- | --- | --- | --- | --- | --- |
| **Age (years)** | | | | | 0.1047 |
| Mean (SD) | 44.9 (16.50) | 45.6 (17.50) | 41.8 (14.81) | 39.3 (13.12) |  |
| **Age group (years)** | | | | | 0.0352 |
| 18 to 64 | 159 (84.6%) | 43 (81.1%) | 259 (91.2%) | 10 (100.0%) |  |
| 65 to 90 | 29 (15.4%) | 10 (18.9%) | 25 (8.8%) | 0 (0.0%) |  |
| **Gender** | | | | | 0.1686 |
| Female | 79 (42.0%) | 27 (50.9%) | 148 (52.1%) | 4 (40.0%) |  |
| Male | 109 (58.0%) | 26 (49.1%) | 136 (47.9%) | 6 (60.0%) |  |
| **First recorded weight** | | | | | 0.7989 |
| Mean (SD) | 75.06 (19.832) | 72.55 (15.573) | 74.03 (16.921) | 72.44 (17.282) |  |
| **Province/region of treating physician** | | | | | 0.0003 |
| Alberta | 11 (5.9%) | 2 (3.8%) | 13 (4.6%) | 1 (10.0%) |  |
| Atlantic^†^ | 29 (15.4%) | 3 (5.7%) | 54 (19.0%) | 2 (20.0%) |  |
| British Columbia | 9 (4.8%) | 2 (3.8%) | 41 (14.4%) | 3 (30.0%) |  |
| Ontario | 77 (41.0%) | 32 (60.4%) | 117 (41.2%) | 1 (10.0%) |  |
| Quebec | 62 (33.0%) | 14 (26.4%) | 55 (19.4%) | 3 (30.0%) |  |
| Saskatchewan/  Manitoba | 0 (0.0%) | 0 (0.0%) | 4 (1.4%) | 0 (0.0%) |  |
| **Year of initiation of IFX treatment** | | | | | 0.2233 |
| 2015 | 47 (25.0%) | 15 (28.3%) | 100 (35.2%) | 5 (50.0%) |  |
| 2016 | 58 (30.9%) | 12 (22.6%) | 72 (25.4%) | 3 (30.0%) |  |
| 2017 | 51 (27.1%) | 13 (24.5%) | 63 (22.2%) | 0 (0.0%) |  |
| 2018 | 32 (17.0%) | 13 (24.5%) | 49 (17.3%) | 2 (20.0%) |  |
| **Time to first TDM (days)** | | | | | 0.3114 |
| Mean (SD) | 557.9 (381.89) | 467.2 (405.28) | 571.3 (380.59) | 619.5 (340.16) |  |
| **Year of first instance of TDM** | | | | | 0.5771 |
| 2015 | 0 (0.0%) | 0 (0.0%) | 0 (0.0%) | 0 (0.0%) |  |
| 2016 | 0 (0.0%) | 0 (0.0%) | 0 (0.0%) | 0 (0.0%) |  |
| 2017 | 69 (36.7%) | 21 (39.6%) | 129 (45.4%) | 4 (40.0%) |  |
| 2018 | 77 (41.0%) | 21 (39.6%) | 105 (37.0%) | 5 (50.0%) |  |
| 2019 | 42 (22.3%) | 11 (20.8%) | 50 (17.6%) | 1 (10.0%) |  |

TDM=therapeutic drug monitoring. P-value: categorical variables, chi-square test; numeric variables, ANOVA.

^†^Atlantic includes New Brunswick, Nova Scotia, Prince Edward Island, Newfoundland, and Labrador.

Dose optimization thresholds: High: A treatment interval decrease of ≥11 days (1.57 weeks) with a posterior interval of ≤35 days (5 weeks), and/or a dose level increase of ≥1.5 mg/kg with a posterior dose level of ≥9 mg/kg. Low: A treatment interval decrease of ≥11 days (1.57 weeks) with a posterior interval of ≤46 days (6.57 weeks), and/or dose level increase of ≥1.5 mg/kg with a posterior dose level of ≥7 mg/kg.

A=First TDM serum IFX concentration below threshold and no dose optimization within post-index period;

B=First TDM serum IFX concentration below threshold and dose optimization within post-index period;

C=First TDM serum IFX concentration at or above threshold and no dose optimization within post-index period;

D=First TDM serum IFX concentration at or above threshold and dose optimization within post-index period.

Suppl Table 6j: Subgroup Demographics and Baseline Characteristics of Patients with CD with a DO (Based on Low Threshold Criteria) Prior to their First Instance of TDM (Serum IFX Threshold: 5 μg/mL; Post-Index Period for Dose Optimization: +17 weeks; Post-Index Dose Optimization Threshold: High)

|  | **A (N=168)** | **B (N=73)** | **C (N=274)** | **D (N=20)** | **p-value** |
| --- | --- | --- | --- | --- | --- |
| **Age (years)** | | | | | 0.0920 |
| Mean (SD) | 44.5 (16.47) | 46.2 (17.25) | 41.8 (14.84) | 41.1 (13.71) |  |
| **Age group (years)** | | | | | 0.0358 |
| 18 to 64 | 143 (85.1%) | 59 (80.8%) | 250 (91.2%) | 19 (95.0%) |  |
| 65 to 90 | 25 (14.9%) | 14 (19.2%) | 24 (8.8%) | 1 (5.0%) |  |
| **Gender** | | | | | 0.0698 |
| Female | 68 (40.5%) | 38 (52.1%) | 144 (52.6%) | 8 (40.0%) |  |
| Male | 100 (59.5%) | 35 (47.9%) | 130 (47.4%) | 12 (60.0%) |  |
| **First recorded weight** | | | | | 0.5488 |
| Mean (SD) | 75.47 (18.811) | 72.30 (19.299) | 74.15 (17.081) | 71.55 (14.446) |  |
| **Province/region of treating physician** | | | | | <0.0001 |
| Alberta | 11 (6.5%) | 2 (2.7%) | 13 (4.7%) | 1 (5.0%) |  |
| Atlantic^†^ | 25 (14.9%) | 7 (9.6%) | 52 (19.0%) | 4 (20.0%) |  |
| British Columbia | 9 (5.4%) | 2 (2.7%) | 36 (13.1%) | 8 (40.0%) |  |
| Ontario | 67 (39.9%) | 42 (57.5%) | 114 (41.6%) | 4 (20.0%) |  |
| Quebec | 56 (33.3%) | 20 (27.4%) | 55 (20.1%) | 3 (15.0%) |  |
| Saskatchewan/  Manitoba | 0 (0.0%) | 0 (0.0%) | 4 (1.5%) | 0 (0.0%) |  |
| **Year of initiation of IFX treatment** | | | | | 0.1691 |
| 2015 | 43 (25.6%) | 19 (26.0%) | 98 (35.8%) | 7 (35.0%) |  |
| 2016 | 52 (31.0%) | 18 (24.7%) | 69 (25.2%) | 6 (30.0%) |  |
| 2017 | 47 (28.0%) | 17 (23.3%) | 61 (22.3%) | 2 (10.0%) |  |
| 2018 | 26 (15.5%) | 19 (26.0%) | 46 (16.8%) | 5 (25.0%) |  |
| **Time to first TDM (days)** | | | | | 0.1946 |
| Mean (SD) | 568.6 (385.54) | 467.4 (387.46) | 571.7 (376.40) | 591.0 (421.16) |  |
| **Year of first instance of TDM** | | | | | 0.5399 |
| 2015 | 0 (0.0%) | 0 (0.0%) | 0 (0.0%) | 0 (0.0%) |  |
| 2016 | 0 (0.0%) | 0 (0.0%) | 0 (0.0%) | 0 (0.0%) |  |
| 2017 | 61 (36.3%) | 29 (39.7%) | 126 (46.0%) | 7 (35.0%) |  |
| 2018 | 70 (41.7%) | 28 (38.4%) | 101 (36.9%) | 9 (45.0%) |  |
| 2019 | 37 (22.0%) | 16 (21.9%) | 47 (17.2%) | 4 (20.0%) |  |

TDM=therapeutic drug monitoring. P-value: categorical variables, chi-square test; numeric variables, ANOVA.

^†^Atlantic includes New Brunswick, Nova Scotia, Prince Edward Island, Newfoundland, and Labrador.

Dose optimization thresholds: High: A treatment interval decrease of ≥11 days (1.57 weeks) with a posterior interval of ≤35 days (5 weeks), and/or a dose level increase of ≥1.5 mg/kg with a posterior dose level of ≥9 mg/kg. Low: A treatment interval decrease of ≥11 days (1.57 weeks) with a posterior interval of ≤46 days (6.57 weeks), and/or dose level increase of ≥1.5 mg/kg with a posterior dose level of ≥7 mg/kg.

A=First TDM serum IFX concentration below threshold and no dose optimization within post-index period;

B=First TDM serum IFX concentration below threshold and dose optimization within post-index period;

C=First TDM serum IFX concentration at or above threshold and no dose optimization within post-index period;

D=First TDM serum IFX concentration at or above threshold and dose optimization within post-index period.

Suppl Table 6k: Subgroup Demographics and Baseline Characteristics of Patients with CD with a DO (Based on Low Threshold Criteria) Prior to their First Instance of TDM (Serum IFX Threshold: 10 μg/mL; Post-Index Period for Dose Optimization: +9 weeks; Post-Index Dose Optimization Threshold: High)

|  | **A (N=304)** | **B (N=57)** | **C (N=168)** | **D (N=6)** | **p-value** |
| --- | --- | --- | --- | --- | --- |
| **Age (years)** | | | | | 0.0446 |
| Mean (SD) | 44.3 (15.85) | 45.4 (17.29) | 40.7 (14.76) | 36.8 (11.51) |  |
| **Age group (years)** | | | | | 0.0359 |
| 18 to 64 | 261 (85.9%) | 47 (82.5%) | 157 (93.5%) | 6 (100.0%) |  |
| 65 to 90 | 43 (14.1%) | 10 (17.5%) | 11 (6.5%) | 0 (0.0%) |  |
| **Gender** | | | | | 0.2078 |
| Female | 136 (44.7%) | 29 (50.9%) | 91 (54.2%) | 2 (33.3%) |  |
| Male | 168 (55.3%) | 28 (49.1%) | 77 (45.8%) | 4 (66.7%) |  |
| **First recorded weight** | | | | | 0.7294 |
| Mean (SD) | 74.93 (18.663) | 72.42 (15.455) | 73.55 (17.121) | 73.57 (19.577) |  |
| **Province/region of treating physician** | | | | | <0.0001 |
| Alberta | 13 (4.3%) | 2 (3.5%) | 11 (6.5%) | 1 (16.7%) |  |
| Atlantic^†^ | 53 (17.4%) | 4 (7.0%) | 30 (17.9%) | 1 (16.7%) |  |
| British Columbia | 20 (6.6%) | 2 (3.5%) | 30 (17.9%) | 3 (50.0%) |  |
| Ontario | 128 (42.1%) | 32 (56.1%) | 66 (39.3%) | 1 (16.7%) |  |
| Quebec | 88 (28.9%) | 17 (29.8%) | 29 (17.3%) | 0 (0.0%) |  |
| Saskatchewan/  Manitoba | 2 (0.7%) | 0 (0.0%) | 2 (1.2%) | 0 (0.0%) |  |
| **Year of initiation of IFX treatment** | | | | | 0.5713 |
| 2015 | 91 (29.9%) | 17 (29.8%) | 56 (33.3%) | 3 (50.0%) |  |
| 2016 | 90 (29.6%) | 13 (22.8%) | 40 (23.8%) | 2 (33.3%) |  |
| 2017 | 76 (25.0%) | 13 (22.8%) | 38 (22.6%) | 0 (0.0%) |  |
| 2018 | 47 (15.5%) | 14 (24.6%) | 34 (20.2%) | 1 (16.7%) |  |
| **Time to first TDM (days)** | | | | | 0.2155 |
| Mean (SD) | 585.8 (392.36) | 486.8 (412.84) | 530.2 (357.17) | 534.5 (210.27) |  |
| **Year of first instance of TDM** | | | | | 0.5072 |
| 2015 | 0 (0.0%) | 0 (0.0%) | 0 (0.0%) | 0 (0.0%) |  |
| 2016 | 0 (0.0%) | 0 (0.0%) | 0 (0.0%) | 0 (0.0%) |  |
| 2017 | 120 (39.5%) | 21 (36.8%) | 78 (46.4%) | 4 (66.7%) |  |
| 2018 | 119 (39.1%) | 25 (43.9%) | 63 (37.5%) | 1 (16.7%) |  |
| 2019 | 65 (21.4%) | 11 (19.3%) | 27 (16.1%) | 1 (16.7%) |  |

TDM=therapeutic drug monitoring. P-value: categorical variables, chi-square test; numeric variables, ANOVA.

^†^Atlantic includes New Brunswick, Nova Scotia, Prince Edward Island, Newfoundland, and Labrador.

Dose optimization thresholds: High: A treatment interval decrease of ≥11 days (1.57 weeks) with a posterior interval of ≤35 days (5 weeks), and/or a dose level increase of ≥1.5 mg/kg with a posterior dose level of ≥9 mg/kg. Low: A treatment interval decrease of ≥11 days (1.57 weeks) with a posterior interval of ≤46 days (6.57 weeks), and/or dose level increase of ≥1.5 mg/kg with a posterior dose level of ≥7 mg/kg.

A=First TDM serum IFX concentration below threshold and no dose optimization within post-index period;

B=First TDM serum IFX concentration below threshold and dose optimization within post-index period;

C=First TDM serum IFX concentration at or above threshold and no dose optimization within post-index period;

D=First TDM serum IFX concentration at or above threshold and dose optimization within post-index period.

Suppl Table 6l: Subgroup Demographics and Baseline Characteristics of Patients with CD with a DO (Based on Low Threshold Criteria) Prior to their First Instance of TDM (Serum IFX Threshold: 10 μg/mL; Post-Index Period for Dose Optimization: +17 weeks; Post-Index Dose Optimization Threshold: High)

|  | **A (N=279)** | **B (N=82)** | **C (N=163)** | **D (N=11)** | **p-value** |
| --- | --- | --- | --- | --- | --- |
| **Age (years)** | | | | | 0.0364 |
| Mean (SD) | 44.1 (15.85) | 46.0 (16.83) | 40.7 (14.73) | 38.8 (14.06) |  |
| **Age group (years)** | | | | | 0.0228 |
| 18 to 64 | 241 (86.4%) | 67 (81.7%) | 152 (93.3%) | 11 (100.0%) |  |
| 65 to 90 | 38 (13.6%) | 15 (18.3%) | 11 (6.7%) | 0 (0.0%) |  |
| **Gender** | | | | | 0.0467 |
| Female | 122 (43.7%) | 43 (52.4%) | 90 (55.2%) | 3 (27.3%) |  |
| Male | 157 (56.3%) | 39 (47.6%) | 73 (44.8%) | 8 (72.7%) |  |
| **First recorded weight** | | | | | 0.4895 |
| Mean (SD) | 75.26 (18.045) | 72.05 (18.606) | 73.60 (17.234) | 72.75 (16.568) |  |
| **Province/region of treating physician** | | | | | <0.0001 |
| Alberta | 13 (4.7%) | 2 (2.4%) | 11 (6.7%) | 1 (9.1%) |  |
| Atlantic^†^ | 48 (17.2%) | 9 (11.0%) | 29 (17.8%) | 2 (18.2%) |  |
| British Columbia | 18 (6.5%) | 4 (4.9%) | 27 (16.6%) | 6 (54.5%) |  |
| Ontario | 116 (41.6%) | 44 (53.7%) | 65 (39.9%) | 2 (18.2%) |  |
| Quebec | 82 (29.4%) | 23 (28.0%) | 29 (17.8%) | 0 (0.0%) |  |
| Saskatchewan/  Manitoba | 2 (0.7%) | 0 (0.0%) | 2 (1.2%) | 0 (0.0%) |  |
| **Year of initiation of IFX treatment** | | | | | 0.2561 |
| 2015 | 87 (31.2%) | 21 (25.6%) | 54 (33.1%) | 5 (45.5%) |  |
| 2016 | 82 (29.4%) | 21 (25.6%) | 39 (23.9%) | 3 (27.3%) |  |
| 2017 | 70 (25.1%) | 19 (23.2%) | 38 (23.3%) | 0 (0.0%) |  |
| 2018 | 40 (14.3%) | 21 (25.6%) | 32 (19.6%) | 3 (27.3%) |  |
| **Time to first TDM (days)** | | | | | 0.0515 |
| Mean (SD) | 596.9 (395.37) | 479.0 (390.05) | 525.3 (347.11) | 605.8 (440.47) |  |
| **Year of first instance of TDM** | | | | | 0.5748 |
| 2015 | 0 (0.0%) | 0 (0.0%) | 0 (0.0%) | 0 (0.0%) |  |
| 2016 | 0 (0.0%) | 0 (0.0%) | 0 (0.0%) | 0 (0.0%) |  |
| 2017 | 110 (39.4%) | 31 (37.8%) | 77 (47.2%) | 5 (45.5%) |  |
| 2018 | 110 (39.4%) | 34 (41.5%) | 61 (37.4%) | 3 (27.3%) |  |
| 2019 | 59 (21.1%) | 17 (20.7%) | 25 (15.3%) | 3 (27.3%) |  |

TDM=therapeutic drug monitoring. P-value: categorical variables, chi-square test; numeric variables, ANOVA.

^†^Atlantic includes New Brunswick, Nova Scotia, Prince Edward Island, Newfoundland, and Labrador.

Dose optimization thresholds: High: A treatment interval decrease of ≥11 days (1.57 weeks) with a posterior interval of ≤35 days (5 weeks), and/or a dose level increase of ≥1.5 mg/kg with a posterior dose level of ≥9 mg/kg. Low: A treatment interval decrease of ≥11 days (1.57 weeks) with a posterior interval of ≤46 days (6.57 weeks), and/or dose level increase of ≥1.5 mg/kg with a posterior dose level of ≥7 mg/kg.

A=First TDM serum IFX concentration below threshold and no dose optimization within post-index period;

B=First TDM serum IFX concentration below threshold and dose optimization within post-index period;

C=First TDM serum IFX concentration at or above threshold and no dose optimization within post-index period;

D=First TDM serum IFX concentration at or above threshold and dose optimization within post-index period.

Suppl Table 7a: Subgroup Demographics and Baseline Characteristics of Patients with CD with a DO (Based on High Threshold Criteria) Prior to their First Instance of TDM (Serum IFX Threshold: 3 μg/mL; Post-Index Period for Dose Optimization: +9 weeks; Post-Index Dose Optimization Threshold: Low)

|  | **A (N=60)** | **B (N=34)** | **C (N=224)** | **D (N=24)** | **p-value** |
| --- | --- | --- | --- | --- | --- |
| **Age (years)** | | | | | 0.0395 |
| Mean (SD) | 45.5 (16.47) | 48.6 (19.85) | 41.8 (15.24) | 39.0 (14.37) |  |
| **Age group (years)** | | | | | 0.0428 |
| 18 to 64 | 52 (86.7%) | 25 (73.5%) | 202 (90.2%) | 22 (91.7%) |  |
| 65 to 90 | 8 (13.3%) | 9 (26.5%) | 22 (9.8%) | 2 (8.3%) |  |
| **Gender** | | | | | 0.4790 |
| Female | 24 (40.0%) | 13 (38.2%) | 106 (47.3%) | 13 (54.2%) |  |
| Male | 36 (60.0%) | 21 (61.8%) | 118 (52.7%) | 11 (45.8%) |  |
| **First recorded weight** | | | | | 0.3613 |
| Mean (SD) | 77.57 (21.122) | 72.08 (18.909) | 73.63 (16.843) | 71.79 (18.307) |  |
| **Province/region of treating physician** | | | | | 0.0352 |
| Alberta | 6 (10.0%) | 1 (2.9%) | 14 (6.3%) | 2 (8.3%) |  |
| Atlantic^†^ | 8 (13.3%) | 2 (5.9%) | 40 (17.9%) | 2 (8.3%) |  |
| British Columbia | 2 (3.3%) | 1 (2.9%) | 31 (13.8%) | 4 (16.7%) |  |
| Ontario | 22 (36.7%) | 23 (67.6%) | 91 (40.6%) | 11 (45.8%) |  |
| Quebec | 22 (36.7%) | 7 (20.6%) | 45 (20.1%) | 5 (20.8%) |  |
| Saskatchewan/  Manitoba | 0 (0.0%) | 0 (0.0%) | 3 (1.3%) | 0 (0.0%) |  |
| **Year of initiation of IFX treatment** | | | | | 0.1117 |
| 2015 | 7 (11.7%) | 9 (26.5%) | 74 (33.0%) | 6 (25.0%) |  |
| 2016 | 17 (28.3%) | 9 (26.5%) | 47 (21.0%) | 6 (25.0%) |  |
| 2017 | 17 (28.3%) | 6 (17.6%) | 57 (25.4%) | 8 (33.3%) |  |
| 2018 | 19 (31.7%) | 10 (29.4%) | 46 (20.5%) | 4 (16.7%) |  |
| **Time to first TDM (days)** | | | | | 0.1537 |
| Mean (SD) | 432.3 (343.70) | 522.0 (457.01) | 543.1 (385.33) | 428.2 (300.16) |  |
| **Year of first instance of TDM** | | | | | 0.0933 |
| 2015 | 0 (0.0%) | 0 (0.0%) | 0 (0.0%) | 0 (0.0%) |  |
| 2016 | 0 (0.0%) | 0 (0.0%) | 0 (0.0%) | 0 (0.0%) |  |
| 2017 | 17 (28.3%) | 10 (29.4%) | 98 (43.8%) | 10 (41.7%) |  |
| 2018 | 25 (41.7%) | 14 (41.2%) | 85 (37.9%) | 12 (50.0%) |  |
| 2019 | 18 (30.0%) | 10 (29.4%) | 41 (18.3%) | 2 (8.3%) |  |

TDM=therapeutic drug monitoring. P-value: categorical variables, chi-square test; numeric variables, ANOVA.

^†^Atlantic includes New Brunswick, Nova Scotia, Prince Edward Island, Newfoundland, and Labrador.

Dose optimization thresholds: High: A treatment interval decrease of ≥11 days (1.57 weeks) with a posterior interval of ≤35 days (5 weeks), and/or a dose level increase of ≥1.5 mg/kg with a posterior dose level of ≥9 mg/kg. Low: A treatment interval decrease of ≥11 days (1.57 weeks) with a posterior interval of ≤46 days (6.57 weeks), and/or dose level increase of ≥1.5 mg/kg with a posterior dose level of ≥7 mg/kg.

A=First TDM serum IFX concentration below threshold and no dose optimization within post-index period;

B=First TDM serum IFX concentration below threshold and dose optimization within post-index period;

C=First TDM serum IFX concentration at or above threshold and no dose optimization within post-index period;

D=First TDM serum IFX concentration at or above threshold and dose optimization within post-index period.

Suppl Table 7b: Subgroup Demographics and Baseline Characteristics of Patients with CD with a DO (Based on High Threshold Criteria) Prior to their First Instance of TDM (Serum IFX Threshold: 3 μg/mL; Post-Index Period for Dose Optimization: +17 weeks; Post-Index Dose Optimization Threshold: Low)

|  | **A (N=49)** | **B (N=45)** | **C (N=205)** | **D (N=43)** | **p-value** |
| --- | --- | --- | --- | --- | --- |
| **Age (years)** | | | | | 0.0494 |
| Mean (SD) | 45.1 (16.70) | 48.3 (18.82) | 41.5 (15.13) | 41.9 (15.42) |  |
| **Age group (years)** | | | | | 0.0485 |
| 18 to 64 | 43 (87.8%) | 34 (75.6%) | 185 (90.2%) | 39 (90.7%) |  |
| 65 to 90 | 6 (12.2%) | 11 (24.4%) | 20 (9.8%) | 4 (9.3%) |  |
| **Gender** | | | | | 0.4416 |
| Female | 19 (38.8%) | 18 (40.0%) | 96 (46.8%) | 23 (53.5%) |  |
| Male | 30 (61.2%) | 27 (60.0%) | 109 (53.2%) | 20 (46.5%) |  |
| **First recorded weight** | | | | | 0.1702 |
| Mean (SD) | 77.88 (19.003) | 73.08 (21.795) | 74.26 (17.022) | 69.60 (16.306) |  |
| **Province/region of treating physician** | | | | | 0.0372 |
| Alberta | 6 (12.2%) | 1 (2.2%) | 14 (6.8%) | 2 (4.7%) |  |
| Atlantic^†^ | 7 (14.3%) | 3 (6.7%) | 36 (17.6%) | 6 (14.0%) |  |
| British Columbia | 2 (4.1%) | 1 (2.2%) | 27 (13.2%) | 8 (18.6%) |  |
| Ontario | 17 (34.7%) | 28 (62.2%) | 84 (41.0%) | 18 (41.9%) |  |
| Quebec | 17 (34.7%) | 12 (26.7%) | 41 (20.0%) | 9 (20.9%) |  |
| Saskatchewan/  Manitoba | 0 (0.0%) | 0 (0.0%) | 3 (1.5%) | 0 (0.0%) |  |
| **Year of initiation of IFX treatment** | | | | | 0.1210 |
| 2015 | 6 (12.2%) | 10 (22.2%) | 69 (33.7%) | 11 (25.6%) |  |
| 2016 | 14 (28.6%) | 12 (26.7%) | 43 (21.0%) | 10 (23.3%) |  |
| 2017 | 15 (30.6%) | 8 (17.8%) | 53 (25.9%) | 12 (27.9%) |  |
| 2018 | 14 (28.6%) | 15 (33.3%) | 40 (19.5%) | 10 (23.3%) |  |
| **Time to first TDM (days)** | | | | | 0.3732 |
| Mean (SD) | 452.5 (349.39) | 478.1 (430.69) | 542.6 (374.05) | 481.1 (402.15) |  |
| **Year of first instance of TDM** | | | | | 0.1321 |
| 2015 | 0 (0.0%) | 0 (0.0%) | 0 (0.0%) | 0 (0.0%) |  |
| 2016 | 0 (0.0%) | 0 (0.0%) | 0 (0.0%) | 0 (0.0%) |  |
| 2017 | 13 (26.5%) | 14 (31.1%) | 91 (44.4%) | 17 (39.5%) |  |
| 2018 | 22 (44.9%) | 17 (37.8%) | 79 (38.5%) | 18 (41.9%) |  |
| 2019 | 14 (28.6%) | 14 (31.1%) | 35 (17.1%) | 8 (18.6%) |  |

TDM=therapeutic drug monitoring. P-value: categorical variables, chi-square test; numeric variables, ANOVA.

^†^Atlantic includes New Brunswick, Nova Scotia, Prince Edward Island, Newfoundland, and Labrador.

Dose optimization thresholds: High: A treatment interval decrease of ≥11 days (1.57 weeks) with a posterior interval of ≤35 days (5 weeks), and/or a dose level increase of ≥1.5 mg/kg with a posterior dose level of ≥9 mg/kg. Low: A treatment interval decrease of ≥11 days (1.57 weeks) with a posterior interval of ≤46 days (6.57 weeks), and/or dose level increase of ≥1.5 mg/kg with a posterior dose level of ≥7 mg/kg.

A=First TDM serum IFX concentration below threshold and no dose optimization within post-index period;

B=First TDM serum IFX concentration below threshold and dose optimization within post-index period;

C=First TDM serum IFX concentration at or above threshold and no dose optimization within post-index period;

D=First TDM serum IFX concentration at or above threshold and dose optimization within post-index period.

Suppl Table 7c: Subgroup Demographics and Baseline Characteristics of Patients with CD with a DO (Based on High Threshold Criteria) Prior to their First Instance of TDM (Serum IFX Threshold: 5 μg/mL; Post-Index Period for Dose Optimization: +9 weeks; Post-Index Dose Optimization Threshold: Low)

|  | **A (N=98)** | **B (N=46)** | **C (N=186)** | **D (N=12)** | **p-value** |
| --- | --- | --- | --- | --- | --- |
| **Age (years)** | | | | | 0.0239 |
| Mean (SD) | 45.8 (16.29) | 46.4 (19.52) | 40.9 (14.92) | 37.7 (10.34) |  |
| **Age group (years)** | | | | | 0.0090 |
| 18 to 64 | 83 (84.7%) | 35 (76.1%) | 171 (91.9%) | 12 (100.0%) |  |
| 65 to 90 | 15 (15.3%) | 11 (23.9%) | 15 (8.1%) | 0 (0.0%) |  |
| **Gender** | | | | | 0.4029 |
| Female | 45 (45.9%) | 18 (39.1%) | 85 (45.7%) | 8 (66.7%) |  |
| Male | 53 (54.1%) | 28 (60.9%) | 101 (54.3%) | 4 (33.3%) |  |
| **First recorded weight** | | | | | 0.8084 |
| Mean (SD) | 74.18 (18.580) | 71.86 (18.797) | 74.61 (17.525) | 72.34 (18.108) |  |
| **Province/region of treating physician** | | | | | 0.0001 |
| Alberta | 10 (10.2%) | 2 (4.3%) | 10 (5.4%) | 1 (8.3%) |  |
| Atlantic^†^ | 13 (13.3%) | 4 (8.7%) | 35 (18.8%) | 0 (0.0%) |  |
| British Columbia | 3 (3.1%) | 1 (2.2%) | 30 (16.1%) | 4 (33.3%) |  |
| Ontario | 37 (37.8%) | 29 (63.0%) | 76 (40.9%) | 5 (41.7%) |  |
| Quebec | 35 (35.7%) | 10 (21.7%) | 32 (17.2%) | 2 (16.7%) |  |
| Saskatchewan/  Manitoba | 0 (0.0%) | 0 (0.0%) | 3 (1.6%) | 0 (0.0%) |  |
| **Year of initiation of IFX treatment** | | | | | 0.7294 |
| 2015 | 20 (20.4%) | 11 (23.9%) | 61 (32.8%) | 4 (33.3%) |  |
| 2016 | 25 (25.5%) | 12 (26.1%) | 39 (21.0%) | 3 (25.0%) |  |
| 2017 | 29 (29.6%) | 11 (23.9%) | 45 (24.2%) | 3 (25.0%) |  |
| 2018 | 24 (24.5%) | 12 (26.1%) | 41 (22.0%) | 2 (16.7%) |  |
| **Time to first TDM (days)** | | | | | 0.6039 |
| Mean (SD) | 491.6 (377.95) | 463.1 (419.56) | 534.5 (379.80) | 560.2 (312.94) |  |
| **Year of first instance of TDM** | | | | | 0.2372 |
| 2015 | 0 (0.0%) | 0 (0.0%) | 0 (0.0%) | 0 (0.0%) |  |
| 2016 | 0 (0.0%) | 0 (0.0%) | 0 (0.0%) | 0 (0.0%) |  |
| 2017 | 32 (32.7%) | 17 (37.0%) | 83 (44.6%) | 3 (25.0%) |  |
| 2018 | 43 (43.9%) | 18 (39.1%) | 67 (36.0%) | 8 (66.7%) |  |
| 2019 | 23 (23.5%) | 11 (23.9%) | 36 (19.4%) | 1 (8.3%) |  |

TDM=therapeutic drug monitoring. P-value: categorical variables, chi-square test; numeric variables, ANOVA.

^†^Atlantic includes New Brunswick, Nova Scotia, Prince Edward Island, Newfoundland, and Labrador.

Dose optimization thresholds: High: A treatment interval decrease of ≥11 days (1.57 weeks) with a posterior interval of ≤35 days (5 weeks), and/or a dose level increase of ≥1.5 mg/kg with a posterior dose level of ≥9 mg/kg. Low: A treatment interval decrease of ≥11 days (1.57 weeks) with a posterior interval of ≤46 days (6.57 weeks), and/or dose level increase of ≥1.5 mg/kg with a posterior dose level of ≥7 mg/kg.

A=First TDM serum IFX concentration below threshold and no dose optimization within post-index period;

B=First TDM serum IFX concentration below threshold and dose optimization within post-index period;

C=First TDM serum IFX concentration at or above threshold and no dose optimization within post-index period;

D=First TDM serum IFX concentration at or above threshold and dose optimization within post-index period.

Suppl Table 7d: Subgroup Demographics and Baseline Characteristics of Patients with CD with a DO (Based on High Threshold Criteria) Prior to their First Instance of TDM (Serum IFX Threshold: 5 μg/mL; Post-Index Period for Dose Optimization: +17 weeks; Post-Index Dose Optimization Threshold: Low)

|  | **A (N=80)** | **B (N=64)** | **C (N=174)** | **D (N=24)** | **p-value** |
| --- | --- | --- | --- | --- | --- |
| **Age (years)** | | | | | 0.0241 |
| Mean (SD) | 45.1 (16.36) | 47.0 (18.53) | 40.8 (14.90) | 40.3 (13.26) |  |
| **Age group (years)** | | | | | 0.0143 |
| 18 to 64 | 68 (85.0%) | 50 (78.1%) | 160 (92.0%) | 23 (95.8%) |  |
| 65 to 90 | 12 (15.0%) | 14 (21.9%) | 14 (8.0%) | 1 (4.2%) |  |
| **Gender** | | | | | 0.8209 |
| Female | 35 (43.8%) | 28 (43.8%) | 80 (46.0%) | 13 (54.2%) |  |
| Male | 45 (56.3%) | 36 (56.3%) | 94 (54.0%) | 11 (45.8%) |  |
| **First recorded weight** | | | | | 0.4507 |
| Mean (SD) | 74.86 (16.602) | 71.66 (20.861) | 75.00 (17.860) | 70.64 (14.594) |  |
| **Province/region of treating physician** | | | | | <0.0001 |
| Alberta | 10 (12.5%) | 2 (3.1%) | 10 (5.7%) | 1 (4.2%) |  |
| Atlantic^†^ | 10 (12.5%) | 7 (10.9%) | 33 (19.0%) | 2 (8.3%) |  |
| British Columbia | 3 (3.8%) | 1 (1.6%) | 26 (14.9%) | 8 (33.3%) |  |
| Ontario | 31 (38.8%) | 35 (54.7%) | 70 (40.2%) | 11 (45.8%) |  |
| Quebec | 26 (32.5%) | 19 (29.7%) | 32 (18.4%) | 2 (8.3%) |  |
| Saskatchewan/  Manitoba | 0 (0.0%) | 0 (0.0%) | 3 (1.7%) | 0 (0.0%) |  |
| **Year of initiation of IFX treatment** | | | | | 0.5745 |
| 2015 | 18 (22.5%) | 13 (20.3%) | 57 (32.8%) | 8 (33.3%) |  |
| 2016 | 20 (25.0%) | 17 (26.6%) | 37 (21.3%) | 5 (20.8%) |  |
| 2017 | 25 (31.3%) | 15 (23.4%) | 43 (24.7%) | 5 (20.8%) |  |
| 2018 | 17 (21.3%) | 19 (29.7%) | 37 (21.3%) | 6 (25.0%) |  |
| **Time to first TDM (days)** | | | | | 0.2935 |
| Mean (SD) | 517.9 (380.81) | 438.2 (400.80) | 528.6 (366.67) | 589.9 (438.97) |  |
| **Year of first instance of TDM** | | | | | 0.3422 |
| 2015 | 0 (0.0%) | 0 (0.0%) | 0 (0.0%) | 0 (0.0%) |  |
| 2016 | 0 (0.0%) | 0 (0.0%) | 0 (0.0%) | 0 (0.0%) |  |
| 2017 | 25 (31.3%) | 24 (37.5%) | 79 (45.4%) | 7 (29.2%) |  |
| 2018 | 37 (46.3%) | 24 (37.5%) | 64 (36.8%) | 11 (45.8%) |  |
| 2019 | 18 (22.5%) | 16 (25.0%) | 31 (17.8%) | 6 (25.0%) |  |

TDM=therapeutic drug monitoring. P-value: categorical variables, chi-square test; numeric variables, ANOVA.

^†^Atlantic includes New Brunswick, Nova Scotia, Prince Edward Island, Newfoundland, and Labrador.

Dose optimization thresholds: High: A treatment interval decrease of ≥11 days (1.57 weeks) with a posterior interval of ≤35 days (5 weeks), and/or a dose level increase of ≥1.5 mg/kg with a posterior dose level of ≥9 mg/kg. Low: A treatment interval decrease of ≥11 days (1.57 weeks) with a posterior interval of ≤46 days (6.57 weeks), and/or dose level increase of ≥1.5 mg/kg with a posterior dose level of ≥7 mg/kg.

A=First TDM serum IFX concentration below threshold and no dose optimization within post-index period;

B=First TDM serum IFX concentration below threshold and dose optimization within post-index period;

C=First TDM serum IFX concentration at or above threshold and no dose optimization within post-index period;

D=First TDM serum IFX concentration at or above threshold and dose optimization within post-index period.

Suppl Table 7e: Subgroup Demographics and Baseline Characteristics of Patients with CD with a DO (Based on High Threshold Criteria) Prior to their First Instance of TDM (Serum IFX Threshold: 10 μg/mL; Post-Index Period for Dose Optimization: +9 weeks; Post-Index Dose Optimization Threshold: Low)

|  | **A (N=162)** | **B (N=50)** | **C (N=122)** | **D (N=8)** | **p-value** |
| --- | --- | --- | --- | --- | --- |
| **Age (years)** | | | | | 0.0335 |
| Mean (SD) | 44.7 (15.95) | 45.6 (19.07) | 39.8 (14.59) | 38.8 (11.46) |  |
| **Age group (years)** | | | | | 0.0112 |
| 18 to 64 | 139 (85.8%) | 39 (78.0%) | 115 (94.3%) | 8 (100.0%) |  |
| 65 to 90 | 23 (14.2%) | 11 (22.0%) | 7 (5.7%) | 0 (0.0%) |  |
| **Gender** | | | | | 0.7561 |
| Female | 74 (45.7%) | 21 (42.0%) | 56 (45.9%) | 5 (62.5%) |  |
| Male | 88 (54.3%) | 29 (58.0%) | 66 (54.1%) | 3 (37.5%) |  |
| **First recorded weight** | | | | | 0.7649 |
| Mean (SD) | 74.44 (18.217) | 71.51 (18.365) | 74.49 (17.461) | 74.75 (20.394) |  |
| **Province/region of treating physician** | | | | | 0.0001 |
| Alberta | 10 (6.2%) | 2 (4.0%) | 10 (8.2%) | 1 (12.5%) |  |
| Atlantic^†^ | 28 (17.3%) | 4 (8.0%) | 20 (16.4%) | 0 (0.0%) |  |
| British Columbia | 10 (6.2%) | 1 (2.0%) | 23 (18.9%) | 4 (50.0%) |  |
| Ontario | 64 (39.5%) | 31 (62.0%) | 49 (40.2%) | 3 (37.5%) |  |
| Quebec | 48 (29.6%) | 12 (24.0%) | 19 (15.6%) | 0 (0.0%) |  |
| Saskatchewan/  Manitoba | 2 (1.2%) | 0 (0.0%) | 1 (0.8%) | 0 (0.0%) |  |
| **Year of initiation of IFX treatment** | | | | | 0.9045 |
| 2015 | 41 (25.3%) | 12 (24.0%) | 40 (32.8%) | 3 (37.5%) |  |
| 2016 | 40 (24.7%) | 13 (26.0%) | 24 (19.7%) | 2 (25.0%) |  |
| 2017 | 45 (27.8%) | 12 (24.0%) | 29 (23.8%) | 2 (25.0%) |  |
| 2018 | 36 (22.2%) | 13 (26.0%) | 29 (23.8%) | 1 (12.5%) |  |
| **Time to first TDM (days)** | | | | | 0.8218 |
| Mean (SD) | 529.0 (394.86) | 473.0 (423.16) | 507.3 (358.21) | 546.8 (198.86) |  |
| **Year of first instance of TDM** | | | | | 0.6744 |
| 2015 | 0 (0.0%) | 0 (0.0%) | 0 (0.0%) | 0 (0.0%) |  |
| 2016 | 0 (0.0%) | 0 (0.0%) | 0 (0.0%) | 0 (0.0%) |  |
| 2017 | 59 (36.4%) | 17 (34.0%) | 56 (45.9%) | 3 (37.5%) |  |
| 2018 | 66 (40.7%) | 22 (44.0%) | 44 (36.1%) | 4 (50.0%) |  |
| 2019 | 37 (22.8%) | 11 (22.0%) | 22 (18.0%) | 1 (12.5%) |  |

TDM=therapeutic drug monitoring. P-value: categorical variables, chi-square test; numeric variables, ANOVA.

^†^Atlantic includes New Brunswick, Nova Scotia, Prince Edward Island, Newfoundland, and Labrador.

Dose optimization thresholds: High: A treatment interval decrease of ≥11 days (1.57 weeks) with a posterior interval of ≤35 days (5 weeks), and/or a dose level increase of ≥1.5 mg/kg with a posterior dose level of ≥9 mg/kg. Low: A treatment interval decrease of ≥11 days (1.57 weeks) with a posterior interval of ≤46 days (6.57 weeks), and/or dose level increase of ≥1.5 mg/kg with a posterior dose level of ≥7 mg/kg.

A=First TDM serum IFX concentration below threshold and no dose optimization within post-index period;

B=First TDM serum IFX concentration below threshold and dose optimization within post-index period;

C=First TDM serum IFX concentration at or above threshold and no dose optimization within post-index period;

D=First TDM serum IFX concentration at or above threshold and dose optimization within post-index period.

Suppl Table 7f: Subgroup Demographics and Baseline Characteristics of Patients with CD with a DO (Based on High Threshold Criteria) Prior to their First Instance of TDM (Serum IFX Threshold: 10 μg/mL; Post-Index Period for Dose Optimization: +17 weeks; Post-Index Dose Optimization Threshold: Low)

|  | **A (N=139)** | **B (N=73)** | **C (N=115)** | **D (N=15)** | **p-value** |
| --- | --- | --- | --- | --- | --- |
| **Age (years)** | | | | | 0.0262 |
| Mean (SD) | 44.3 (16.01) | 46.1 (17.97) | 39.6 (14.45) | 40.7 (14.28) |  |
| **Age group (years)** | | | | | 0.0103 |
| 18 to 64 | 120 (86.3%) | 58 (79.5%) | 108 (93.9%) | 15 (100.0%) |  |
| 65 to 90 | 19 (13.7%) | 15 (20.5%) | 7 (6.1%) | 0 (0.0%) |  |
| **Gender** | | | | | 0.9628 |
| Female | 61 (43.9%) | 34 (46.6%) | 54 (47.0%) | 7 (46.7%) |  |
| Male | 78 (56.1%) | 39 (53.4%) | 61 (53.0%) | 8 (53.3%) |  |
| **First recorded weight** | | | | | 0.4580 |
| Mean (SD) | 75.04 (17.263) | 71.29 (19.890) | 74.85 (17.730) | 71.83 (16.577) |  |
| **Province/region of treating physician** | | | | | 0.0005 |
| Alberta | 10 (7.2%) | 2 (2.7%) | 10 (8.7%) | 1 (6.7%) |  |
| Atlantic^†^ | 24 (17.3%) | 8 (11.0%) | 19 (16.5%) | 1 (6.7%) |  |
| British Columbia | 8 (5.8%) | 3 (4.1%) | 21 (18.3%) | 6 (40.0%) |  |
| Ontario | 56 (40.3%) | 39 (53.4%) | 45 (39.1%) | 7 (46.7%) |  |
| Quebec | 39 (28.1%) | 21 (28.8%) | 19 (16.5%) | 0 (0.0%) |  |
| Saskatchewan/  Manitoba | 2 (1.4%) | 0 (0.0%) | 1 (0.9%) | 0 (0.0%) |  |
| **Year of initiation of IFX treatment** | | | | | 0.6364 |
| 2015 | 38 (27.3%) | 15 (20.5%) | 37 (32.2%) | 6 (40.0%) |  |
| 2016 | 33 (23.7%) | 20 (27.4%) | 24 (20.9%) | 2 (13.3%) |  |
| 2017 | 40 (28.8%) | 17 (23.3%) | 28 (24.3%) | 3 (20.0%) |  |
| 2018 | 28 (20.1%) | 21 (28.8%) | 26 (22.6%) | 4 (26.7%) |  |
| **Time to first TDM (days)** | | | | | 0.4835 |
| Mean (SD) | 543.6 (391.10) | 462.8 (417.92) | 503.0 (344.26) | 561.0 (401.71) |  |
| **Year of first instance of TDM** | | | | | 0.5614 |
| 2015 | 0 (0.0%) | 0 (0.0%) | 0 (0.0%) | 0 (0.0%) |  |
| 2016 | 0 (0.0%) | 0 (0.0%) | 0 (0.0%) | 0 (0.0%) |  |
| 2017 | 50 (36.0%) | 26 (35.6%) | 54 (47.0%) | 5 (33.3%) |  |
| 2018 | 59 (42.4%) | 29 (39.7%) | 42 (36.5%) | 6 (40.0%) |  |
| 2019 | 30 (21.6%) | 18 (24.7%) | 19 (16.5%) | 4 (26.7%) |  |

TDM=therapeutic drug monitoring. P-value: categorical variables, chi-square test; numeric variables, ANOVA.

^†^Atlantic includes New Brunswick, Nova Scotia, Prince Edward Island, Newfoundland, and Labrador.

Dose optimization thresholds: High: A treatment interval decrease of ≥11 days (1.57 weeks) with a posterior interval of ≤35 days (5 weeks), and/or a dose level increase of ≥1.5 mg/kg with a posterior dose level of ≥9 mg/kg. Low: A treatment interval decrease of ≥11 days (1.57 weeks) with a posterior interval of ≤46 days (6.57 weeks), and/or dose level increase of ≥1.5 mg/kg with a posterior dose level of ≥7 mg/kg.

A=First TDM serum IFX concentration below threshold and no dose optimization within post-index period;

B=First TDM serum IFX concentration below threshold and dose optimization within post-index period;

C=First TDM serum IFX concentration at or above threshold and no dose optimization within post-index period;

D=First TDM serum IFX concentration at or above threshold and dose optimization within post-index period.

Suppl Table 7g: Subgroup Demographics and Baseline Characteristics of Patients with CD with a DO (based on High Threshold Criteria) Prior to their First Instance of TDM (Serum IFX Threshold: 3 μg/mL; Post-Index Period for Dose Optimization: +9 weeks; Post-Index Dose Optimization Threshold: High)

|  | **A (N=69)** | **B (N=25)** | **C (N=229)** | **D (N=19)** | **p-value** |
| --- | --- | --- | --- | --- | --- |
| **Age (years)** | | | | | 0.0416 |
| Mean (SD) | 46.3 (17.17) | 47.6 (19.51) | 41.9 (15.11) | 37.6 (15.48) |  |
| **Age group (years)** | | | | | 0.1266 |
| 18 to 64 | 58 (84.1%) | 19 (76.0%) | 207 (90.4%) | 17 (89.5%) |  |
| 65 to 90 | 11 (15.9%) | 6 (24.0%) | 22 (9.6%) | 2 (10.5%) |  |
| **Gender** | | | | | 0.5059 |
| Female | 27 (39.1%) | 10 (40.0%) | 111 (48.5%) | 8 (42.1%) |  |
| Male | 42 (60.9%) | 15 (60.0%) | 118 (51.5%) | 11 (57.9%) |  |
| **First recorded weight** | | | | | 0.6886 |
| Mean (SD) | 76.00 (22.260) | 74.43 (14.485) | 73.65 (16.804) | 71.01 (19.065) |  |
| **Province/region of treating physician** | | | | | 0.0491 |
| Alberta | 6 (8.7%) | 1 (4.0%) | 14 (6.1%) | 2 (10.5%) |  |
| Atlantic^†^ | 10 (14.5%) | 0 (0.0%) | 40 (17.5%) | 2 (10.5%) |  |
| British Columbia | 3 (4.3%) | 0 (0.0%) | 32 (14.0%) | 3 (15.8%) |  |
| Ontario | 27 (39.1%) | 18 (72.0%) | 95 (41.5%) | 7 (36.8%) |  |
| Quebec | 23 (33.3%) | 6 (24.0%) | 45 (19.7%) | 5 (26.3%) |  |
| Saskatchewan/  Manitoba | 0 (0.0%) | 0 (0.0%) | 3 (1.3%) | 0 (0.0%) |  |
| **Year of initiation of IFX treatment** | | | | | 0.1091 |
| 2015 | 9 (13.0%) | 7 (28.0%) | 74 (32.3%) | 6 (31.6%) |  |
| 2016 | 22 (31.9%) | 4 (16.0%) | 49 (21.4%) | 4 (21.1%) |  |
| 2017 | 18 (26.1%) | 5 (20.0%) | 60 (26.2%) | 5 (26.3%) |  |
| 2018 | 20 (29.0%) | 9 (36.0%) | 46 (20.1%) | 4 (21.1%) |  |
| **Time to first TDM (days)** | | | | | 0.2519 |
| Mean (SD) | 464.9 (373.70) | 464.2 (434.96) | 541.8 (382.55) | 413.1 (317.21) |  |
| **Year of first instance of TDM** | | | | | 0.1243 |
| 2015 | 0 (0.0%) | 0 (0.0%) | 0 (0.0%) | 0 (0.0%) |  |
| 2016 | 0 (0.0%) | 0 (0.0%) | 0 (0.0%) | 0 (0.0%) |  |
| 2017 | 20 (29.0%) | 7 (28.0%) | 98 (42.8%) | 10 (52.6%) |  |
| 2018 | 28 (40.6%) | 11 (44.0%) | 90 (39.3%) | 7 (36.8%) |  |
| 2019 | 21 (30.4%) | 7 (28.0%) | 41 (17.9%) | 2 (10.5%) |  |

TDM=therapeutic drug monitoring. P-value: categorical variables, chi-square test; numeric variables, ANOVA.

^†^Atlantic includes New Brunswick, Nova Scotia, Prince Edward Island, Newfoundland, and Labrador.

Dose optimization thresholds: High: A treatment interval decrease of ≥11 days (1.57 weeks) with a posterior interval of ≤35 days (5 weeks), and/or a dose level increase of ≥1.5 mg/kg with a posterior dose level of ≥9 mg/kg. Low: A treatment interval decrease of ≥11 days (1.57 weeks) with a posterior interval of ≤46 days (6.57 weeks), and/or dose level increase of ≥1.5 mg/kg with a posterior dose level of ≥7 mg/kg.

A=First TDM serum IFX concentration below threshold and no dose optimization within post-index period;

B=First TDM serum IFX concentration below threshold and dose optimization within post-index period;

C=First TDM serum IFX concentration at or above threshold and no dose optimization within post-index period;

D=First TDM serum IFX concentration at or above threshold and dose optimization within post-index period.

Suppl Table 7h: Subgroup Demographics and Baseline Characteristics of Patients with CD with a DO (based on High Threshold Criteria) Prior to their First Instance of TDM (Serum IFX Threshold: 3 μg/mL; Post-Index Period for Dose Optimization: +17 weeks; Post-Index Dose Optimization Threshold: High)

|  | **A (N=60)** | **B (N=34)** | **C (N=215)** | **D (N=33)** | **p-value** |
| --- | --- | --- | --- | --- | --- |
| **Age (years)** | | | | | 0.0738 |
| Mean (SD) | 46.5 (17.46) | 46.9 (18.44) | 41.7 (15.00) | 40.8 (16.29) |  |
| **Age group (years)** | | | | | 0.1647 |
| 18 to 64 | 50 (83.3%) | 27 (79.4%) | 195 (90.7%) | 29 (87.9%) |  |
| 65 to 90 | 10 (16.7%) | 7 (20.6%) | 20 (9.3%) | 4 (12.1%) |  |
| **Gender** | | | | | 0.4529 |
| Female | 22 (36.7%) | 15 (44.1%) | 104 (48.4%) | 15 (45.5%) |  |
| Male | 38 (63.3%) | 19 (55.9%) | 111 (51.6%) | 18 (54.5%) |  |
| **First recorded weight** | | | | | 0.3460 |
| Mean (SD) | 76.15 (20.524) | 74.58 (20.501) | 74.11 (16.873) | 69.14 (17.148) |  |
| **Province/region of treating physician** | | | | | 0.0300 |
| Alberta | 6 (10.0%) | 1 (2.9%) | 14 (6.5%) | 2 (6.1%) |  |
| Atlantic^†^ | 9 (15.0%) | 1 (2.9%) | 36 (16.7%) | 6 (18.2%) |  |
| British Columbia | 3 (5.0%) | 0 (0.0%) | 28 (13.0%) | 7 (21.2%) |  |
| Ontario | 22 (36.7%) | 23 (67.6%) | 91 (42.3%) | 11 (33.3%) |  |
| Quebec | 20 (33.3%) | 9 (26.5%) | 43 (20.0%) | 7 (21.2%) |  |
| Saskatchewan/  Manitoba | 0 (0.0%) | 0 (0.0%) | 3 (1.4%) | 0 (0.0%) |  |
| **Year of initiation of IFX treatment** | | | | | 0.0513 |
| 2015 | 8 (13.3%) | 8 (23.5%) | 72 (33.5%) | 8 (24.2%) |  |
| 2016 | 20 (33.3%) | 6 (17.6%) | 45 (20.9%) | 8 (24.2%) |  |
| 2017 | 16 (26.7%) | 7 (20.6%) | 57 (26.5%) | 8 (24.2%) |  |
| 2018 | 16 (26.7%) | 13 (38.2%) | 41 (19.1%) | 9 (27.3%) |  |
| **Time to first TDM (days)** | | | | | 0.2102 |
| Mean (SD) | 479.9 (378.05) | 438.0 (410.70) | 545.9 (377.33) | 440.9 (382.59) |  |
| **Year of first instance of TDM** | | | | | 0.1726 |
| 2015 | 0 (0.0%) | 0 (0.0%) | 0 (0.0%) | 0 (0.0%) |  |
| 2016 | 0 (0.0%) | 0 (0.0%) | 0 (0.0%) | 0 (0.0%) |  |
| 2017 | 17 (28.3%) | 10 (29.4%) | 94 (43.7%) | 14 (42.4%) |  |
| 2018 | 25 (41.7%) | 14 (41.2%) | 84 (39.1%) | 13 (39.4%) |  |
| 2019 | 18 (30.0%) | 10 (29.4%) | 37 (17.2%) | 6 (18.2%) |  |

TDM=therapeutic drug monitoring. P-value: categorical variables, chi-square test; numeric variables, ANOVA.

^†^Atlantic includes New Brunswick, Nova Scotia, Prince Edward Island, Newfoundland, and Labrador.

Dose optimization thresholds: High: A treatment interval decrease of ≥11 days (1.57 weeks) with a posterior interval of ≤35 days (5 weeks), and/or a dose level increase of ≥1.5 mg/kg with a posterior dose level of ≥9 mg/kg. Low: A treatment interval decrease of ≥11 days (1.57 weeks) with a posterior interval of ≤46 days (6.57 weeks), and/or dose level increase of ≥1.5 mg/kg with a posterior dose level of ≥7 mg/kg.

A=First TDM serum IFX concentration below threshold and no dose optimization within post-index period;

B=First TDM serum IFX concentration below threshold and dose optimization within post-index period;

C=First TDM serum IFX concentration at or above threshold and no dose optimization within post-index period;

D=First TDM serum IFX concentration at or above threshold and dose optimization within post-index period.

Suppl Table 7i: Subgroup Demographics and Baseline Characteristics of Patients with CD with a DO (based on High Threshold Criteria) Prior to their First Instance of TDM (Serum IFX Threshold: 5 μg/mL; Post-Index Period for Dose Optimization: +9 weeks; Post-Index Dose Optimization Threshold: High)

|  | **A (N=107)** | **B (N=37)** | **C (N=191)** | **D (N=7)** | **p-value** |
| --- | --- | --- | --- | --- | --- |
| **Age (years)** | | | | | 0.0132 |
| Mean (SD) | 46.2 (16.76) | 45.2 (19.08) | 41.0 (14.78) | 33.0 (9.52) |  |
| **Age group (years)** | | | | | 0.0216 |
| 18 to 64 | 89 (83.2%) | 29 (78.4%) | 176 (92.1%) | 7 (100.0%) |  |
| 65 to 90 | 18 (16.8%) | 8 (21.6%) | 15 (7.9%) | 0 (0.0%) |  |
| **Gender** | | | | | 0.8954 |
| Female | 48 (44.9%) | 15 (40.5%) | 90 (47.1%) | 3 (42.9%) |  |
| Male | 59 (55.1%) | 22 (59.5%) | 101 (52.9%) | 4 (57.1%) |  |
| **First recorded weight** | | | | | 0.8957 |
| Mean (SD) | 73.45 (19.509) | 73.40 (15.998) | 74.61 (17.461) | 70.63 (20.194) |  |
| **Province/region of treating physician** | | | | | 0.0001 |
| Alberta | 10 (9.3%) | 2 (5.4%) | 10 (5.2%) | 1 (14.3%) |  |
| Atlantic^†^ | 15 (14.0%) | 2 (5.4%) | 35 (18.3%) | 0 (0.0%) |  |
| British Columbia | 4 (3.7%) | 0 (0.0%) | 31 (16.2%) | 3 (42.9%) |  |
| Ontario | 42 (39.3%) | 24 (64.9%) | 80 (41.9%) | 1 (14.3%) |  |
| Quebec | 36 (33.6%) | 9 (24.3%) | 32 (16.8%) | 2 (28.6%) |  |
| Saskatchewan/  Manitoba | 0 (0.0%) | 0 (0.0%) | 3 (1.6%) | 0 (0.0%) |  |
| **Year of initiation of IFX treatment** | | | | | 0.3178 |
| 2015 | 22 (20.6%) | 9 (24.3%) | 61 (31.9%) | 4 (57.1%) |  |
| 2016 | 30 (28.0%) | 7 (18.9%) | 41 (21.5%) | 1 (14.3%) |  |
| 2017 | 30 (28.0%) | 10 (27.0%) | 48 (25.1%) | 0 (0.0%) |  |
| 2018 | 25 (23.4%) | 11 (29.7%) | 41 (21.5%) | 2 (28.6%) |  |
| **Time to first TDM (days)** | | | | | 0.2923 |
| Mean (SD) | 507.6 (390.85) | 409.8 (385.37) | 533.2 (376.54) | 613.4 (362.71) |  |
| **Year of first instance of TDM** | | | | | 0.7176 |
| 2015 | 0 (0.0%) | 0 (0.0%) | 0 (0.0%) | 0 (0.0%) |  |
| 2016 | 0 (0.0%) | 0 (0.0%) | 0 (0.0%) | 0 (0.0%) |  |
| 2017 | 35 (32.7%) | 14 (37.8%) | 83 (43.5%) | 3 (42.9%) |  |
| 2018 | 46 (43.0%) | 15 (40.5%) | 72 (37.7%) | 3 (42.9%) |  |
| 2019 | 26 (24.3%) | 8 (21.6%) | 36 (18.8%) | 1 (14.3%) |  |

TDM=therapeutic drug monitoring. P-value: categorical variables, chi-square test; numeric variables, ANOVA.

^†^Atlantic includes New Brunswick, Nova Scotia, Prince Edward Island, Newfoundland, and Labrador.

Dose optimization thresholds: High: A treatment interval decrease of ≥11 days (1.57 weeks) with a posterior interval of ≤35 days (5 weeks), and/or a dose level increase of ≥1.5 mg/kg with a posterior dose level of ≥9 mg/kg. Low: A treatment interval decrease of ≥11 days (1.57 weeks) with a posterior interval of ≤46 days (6.57 weeks), and/or dose level increase of ≥1.5 mg/kg with a posterior dose level of ≥7 mg/kg.

A=First TDM serum IFX concentration below threshold and no dose optimization within post-index period;

B=First TDM serum IFX concentration below threshold and dose optimization within post-index period;

C=First TDM serum IFX concentration at or above threshold and no dose optimization within post-index period;

D=First TDM serum IFX concentration at or above threshold and dose optimization within post-index period.

Suppl Table 7j: Subgroup Demographics and Baseline Characteristics of Patients with CD with a DO (based on High Threshold Criteria) Prior to their First Instance of TDM (Serum IFX Threshold: 5 μg/mL; Post-Index Period for Dose Optimization: +17 weeks; Post-Index Dose Optimization Threshold: High)

|  | **A (N=93)** | **B (N=51)** | **C (N=182)** | **D (N=16)** | **p-value** |
| --- | --- | --- | --- | --- | --- |
| **Age (years)** | | | | | 0.0265 |
| Mean (SD) | 46.3 (16.81) | 45.4 (18.39) | 40.9 (14.77) | 38.9 (13.89) |  |
| **Age group (years)** | | | | | 0.0308 |
| 18 to 64 | 77 (82.8%) | 41 (80.4%) | 168 (92.3%) | 15 (93.8%) |  |
| 65 to 90 | 16 (17.2%) | 10 (19.6%) | 14 (7.7%) | 1 (6.3%) |  |
| **Gender** | | | | | 0.9235 |
| Female | 40 (43.0%) | 23 (45.1%) | 86 (47.3%) | 7 (43.8%) |  |
| Male | 53 (57.0%) | 28 (54.9%) | 96 (52.7%) | 9 (56.3%) |  |
| **First recorded weight** | | | | | 0.6139 |
| Mean (SD) | 73.85 (17.797) | 72.69 (20.186) | 74.92 (17.709) | 69.37 (14.760) |  |
| **Province/region of treating physician** | | | | | <0.0001 |
| Alberta | 10 (10.8%) | 2 (3.9%) | 10 (5.5%) | 1 (6.3%) |  |
| Atlantic^†^ | 12 (12.9%) | 5 (9.8%) | 33 (18.1%) | 2 (12.5%) |  |
| British Columbia | 4 (4.3%) | 0 (0.0%) | 27 (14.8%) | 7 (43.8%) |  |
| Ontario | 36 (38.7%) | 30 (58.8%) | 77 (42.3%) | 4 (25.0%) |  |
| Quebec | 31 (33.3%) | 14 (27.5%) | 32 (17.6%) | 2 (12.5%) |  |
| Saskatchewan/  Manitoba | 0 (0.0%) | 0 (0.0%) | 3 (1.6%) | 0 (0.0%) |  |
| **Year of initiation of IFX treatment** | | | | | 0.3244 |
| 2015 | 21 (22.6%) | 10 (19.6%) | 59 (32.4%) | 6 (37.5%) |  |
| 2016 | 26 (28.0%) | 11 (21.6%) | 39 (21.4%) | 3 (18.8%) |  |
| 2017 | 27 (29.0%) | 13 (25.5%) | 46 (25.3%) | 2 (12.5%) |  |
| 2018 | 19 (20.4%) | 17 (33.3%) | 38 (20.9%) | 5 (31.3%) |  |
| **Time to first TDM (days)** | | | | | 0.1138 |
| Mean (SD) | 530.2 (396.66) | 395.5 (366.89) | 532.2 (368.89) | 579.4 (455.20) |  |
| **Year of first instance of TDM** | | | | | 0.5686 |
| 2015 | 0 (0.0%) | 0 (0.0%) | 0 (0.0%) | 0 (0.0%) |  |
| 2016 | 0 (0.0%) | 0 (0.0%) | 0 (0.0%) | 0 (0.0%) |  |
| 2017 | 30 (32.3%) | 19 (37.3%) | 81 (44.5%) | 5 (31.3%) |  |
| 2018 | 41 (44.1%) | 20 (39.2%) | 68 (37.4%) | 7 (43.8%) |  |
| 2019 | 22 (23.7%) | 12 (23.5%) | 33 (18.1%) | 4 (25.0%) |  |

TDM=therapeutic drug monitoring. P-value: categorical variables, chi-square test; numeric variables, ANOVA.

^†^Atlantic includes New Brunswick, Nova Scotia, Prince Edward Island, Newfoundland, and Labrador.

Dose optimization thresholds: High: A treatment interval decrease of ≥11 days (1.57 weeks) with a posterior interval of ≤35 days (5 weeks), and/or a dose level increase of ≥1.5 mg/kg with a posterior dose level of ≥9 mg/kg. Low: A treatment interval decrease of ≥11 days (1.57 weeks) with a posterior interval of ≤46 days (6.57 weeks), and/or dose level increase of ≥1.5 mg/kg with a posterior dose level of ≥7 mg/kg.

A=First TDM serum IFX concentration below threshold and no dose optimization within post-index period;

B=First TDM serum IFX concentration below threshold and dose optimization within post-index period;

C=First TDM serum IFX concentration at or above threshold and no dose optimization within post-index period;

D=First TDM serum IFX concentration at or above threshold and dose optimization within post-index period.

Suppl Table 7k: Subgroup Demographics and Baseline Characteristics of Patients with CD with a DO (based on High Threshold Criteria) Prior to their First Instance of TDM (Serum IFX Threshold: 10 μg/mL; Post-Index Period for Dose Optimization: +9 weeks; Post-Index Dose Optimization Threshold: High)

|  | **A (N=173)** | **B (N=39)** | **C (N=125)** | **D (N=5)** | **p-value** |
| --- | --- | --- | --- | --- | --- |
| **Age (years)** | | | | | 0.0268 |
| Mean (SD) | 45.0 (16.21) | 44.4 (18.91) | 39.9 (14.49) | 34.4 (11.01) |  |
| **Age group (years)** | | | | | 0.0211 |
| 18 to 64 | 147 (85.0%) | 31 (79.5%) | 118 (94.4%) | 5 (100.0%) |  |
| 65 to 90 | 26 (15.0%) | 8 (20.5%) | 7 (5.6%) | 0 (0.0%) |  |
| **Gender** | | | | | 0.9142 |
| Female | 79 (45.7%) | 16 (41.0%) | 59 (47.2%) | 2 (40.0%) |  |
| Male | 94 (54.3%) | 23 (59.0%) | 66 (52.8%) | 3 (60.0%) |  |
| **First recorded weight** | | | | | 0.9640 |
| Mean (SD) | 73.92 (18.746) | 72.99 (16.069) | 74.58 (17.481) | 72.68 (21.753) |  |
| **Province/region of treating physician** | | | | | 0.0001 |
| Alberta | 10 (5.8%) | 2 (5.1%) | 10 (8.0%) | 1 (20.0%) |  |
| Atlantic^†^ | 30 (17.3%) | 2 (5.1%) | 20 (16.0%) | 0 (0.0%) |  |
| British Columbia | 11 (6.4%) | 0 (0.0%) | 24 (19.2%) | 3 (60.0%) |  |
| Ontario | 71 (41.0%) | 24 (61.5%) | 51 (40.8%) | 1 (20.0%) |  |
| Quebec | 49 (28.3%) | 11 (28.2%) | 19 (15.2%) | 0 (0.0%) |  |
| Saskatchewan/  Manitoba | 2 (1.2%) | 0 (0.0%) | 1 (0.8%) | 0 (0.0%) |  |
| **Year of initiation of IFX treatment** | | | | | 0.5519 |
| 2015 | 43 (24.9%) | 10 (25.6%) | 40 (32.0%) | 3 (60.0%) |  |
| 2016 | 46 (26.6%) | 7 (17.9%) | 25 (20.0%) | 1 (20.0%) |  |
| 2017 | 47 (27.2%) | 10 (25.6%) | 31 (24.8%) | 0 (0.0%) |  |
| 2018 | 37 (21.4%) | 12 (30.8%) | 29 (23.2%) | 1 (20.0%) |  |
| **Time to first TDM (days)** | | | | | 0.3767 |
| Mean (SD) | 536.8 (399.47) | 422.7 (401.79) | 506.3 (355.40) | 593.8 (169.99) |  |
| **Year of first instance of TDM** | | | | | 0.6687 |
| 2015 | 0 (0.0%) | 0 (0.0%) | 0 (0.0%) | 0 (0.0%) |  |
| 2016 | 0 (0.0%) | 0 (0.0%) | 0 (0.0%) | 0 (0.0%) |  |
| 2017 | 62 (35.8%) | 14 (35.9%) | 56 (44.8%) | 3 (60.0%) |  |
| 2018 | 71 (41.0%) | 17 (43.6%) | 47 (37.6%) | 1 (20.0%) |  |
| 2019 | 40 (23.1%) | 8 (20.5%) | 22 (17.6%) | 1 (20.0%) |  |

TDM=therapeutic drug monitoring. P-value: categorical variables, chi-square test; numeric variables, ANOVA.

^†^Atlantic includes New Brunswick, Nova Scotia, Prince Edward Island, Newfoundland, and Labrador.

Dose optimization thresholds: High: A treatment interval decrease of ≥11 days (1.57 weeks) with a posterior interval of ≤35 days (5 weeks), and/or a dose level increase of ≥1.5 mg/kg with a posterior dose level of ≥9 mg/kg. Low: A treatment interval decrease of ≥11 days (1.57 weeks) with a posterior interval of ≤46 days (6.57 weeks), and/or dose level increase of ≥1.5 mg/kg with a posterior dose level of ≥7 mg/kg.

A=First TDM serum IFX concentration below threshold and no dose optimization within post-index period;

B=First TDM serum IFX concentration below threshold and dose optimization within post-index period;

C=First TDM serum IFX concentration at or above threshold and no dose optimization within post-index period;

D=First TDM serum IFX concentration at or above threshold and dose optimization within post-index period.

Suppl Table 7l: Subgroup Demographics and Baseline Characteristics of Patients with CD with a DO (based on High Threshold Criteria) Prior to their First Instance of TDM (Serum IFX Threshold: 10 μg/mL; Post-Index Period for Dose Optimization: +17 weeks; Post-Index Dose Optimization Threshold: High)

|  | **A (N=154)** | **B (N=58)** | **C (N=121)** | **D (N=9)** | **p-value** |
| --- | --- | --- | --- | --- | --- |
| **Age (years)** | | | | | 0.0338 |
| Mean (SD) | 45.0 (16.31) | 44.8 (17.82) | 39.8 (14.37) | 38.0 (15.25) |  |
| **Age group (years)** | | | | | 0.0225 |
| 18 to 64 | 131 (85.1%) | 47 (81.0%) | 114 (94.2%) | 9 (100.0%) |  |
| 65 to 90 | 23 (14.9%) | 11 (19.0%) | 7 (5.8%) | 0 (0.0%) |  |
| **Gender** | | | | | 0.8104 |
| Female | 68 (44.2%) | 27 (46.6%) | 58 (47.9%) | 3 (33.3%) |  |
| Male | 86 (55.8%) | 31 (53.4%) | 63 (52.1%) | 6 (66.7%) |  |
| **First recorded weight** | | | | | 0.7243 |
| Mean (SD) | 74.35 (17.834) | 72.17 (19.385) | 74.83 (17.629) | 70.14 (17.040) |  |
| **Province/region of treating physician** | | | | | 0.0002 |
| Alberta | 10 (6.5%) | 2 (3.4%) | 10 (8.3%) | 1 (11.1%) |  |
| Atlantic^†^ | 26 (16.9%) | 6 (10.3%) | 19 (15.7%) | 1 (11.1%) |  |
| British Columbia | 9 (5.8%) | 2 (3.4%) | 22 (18.2%) | 5 (55.6%) |  |
| Ontario | 63 (40.9%) | 32 (55.2%) | 50 (41.3%) | 2 (22.2%) |  |
| Quebec | 44 (28.6%) | 16 (27.6%) | 19 (15.7%) | 0 (0.0%) |  |
| Saskatchewan/  Manitoba | 2 (1.3%) | 0 (0.0%) | 1 (0.8%) | 0 (0.0%) |  |
| **Year of initiation of IFX treatment** | | | | | 0.2023 |
| 2015 | 42 (27.3%) | 11 (19.0%) | 38 (31.4%) | 5 (55.6%) |  |
| 2016 | 40 (26.0%) | 13 (22.4%) | 25 (20.7%) | 1 (11.1%) |  |
| 2017 | 42 (27.3%) | 15 (25.9%) | 31 (25.6%) | 0 (0.0%) |  |
| 2018 | 30 (19.5%) | 19 (32.8%) | 27 (22.3%) | 3 (33.3%) |  |
| **Time to first TDM (days)** | | | | | 0.0561 |
| Mean (SD) | 556.4 (404.51) | 408.0 (375.40) | 499.9 (339.78) | 642.0 (473.19) |  |
| **Year of first instance of TDM** | | | | | 0.5743 |
| 2015 | 0 (0.0%) | 0 (0.0%) | 0 (0.0%) | 0 (0.0%) |  |
| 2016 | 0 (0.0%) | 0 (0.0%) | 0 (0.0%) | 0 (0.0%) |  |
| 2017 | 55 (35.7%) | 21 (36.2%) | 56 (46.3%) | 3 (33.3%) |  |
| 2018 | 64 (41.6%) | 24 (41.4%) | 45 (37.2%) | 3 (33.3%) |  |
| 2019 | 35 (22.7%) | 13 (22.4%) | 20 (16.5%) | 3 (33.3%) |  |

TDM=therapeutic drug monitoring. P-value: categorical variables, chi-square test; numeric variables, ANOVA.

^†^Atlantic includes New Brunswick, Nova Scotia, Prince Edward Island, Newfoundland, and Labrador.

Dose optimization thresholds: High: A treatment interval decrease of ≥11 days (1.57 weeks) with a posterior interval of ≤35 days (5 weeks), and/or a dose level increase of ≥1.5 mg/kg with a posterior dose level of ≥9 mg/kg. Low: A treatment interval decrease of ≥11 days (1.57 weeks) with a posterior interval of ≤46 days (6.57 weeks), and/or dose level increase of ≥1.5 mg/kg with a posterior dose level of ≥7 mg/kg.

A=First TDM serum IFX concentration below threshold and no dose optimization within post-index period;

B=First TDM serum IFX concentration below threshold and dose optimization within post-index period;

C=First TDM serum IFX concentration at or above threshold and no dose optimization within post-index period;

D=First TDM serum IFX concentration at or above threshold and dose optimization within post-index period.

Suppl Table 8a: Subgroup Demographics and Baseline Characteristics of Patients with UC with a DO (Based on Low Threshold Criteria) Prior to their First Instance of TDM (Serum IFX Threshold: 3 μg/mL; Post-Index Period for Dose Optimization: +9 weeks; Post-Index Dose Optimization Threshold: Low)

|  | **A (N=112)** | **B (N=68)** | **C (N=310)** | **D (N=40)** | **p-value** |
| --- | --- | --- | --- | --- | --- |
| **Age (years)** | | | | | 0.0459 |
| Mean (SD) | 45.2 (16.71) | 43.4 (16.73) | 41.1 (15.55) | 38.4 (16.16) |  |
| Missing | 0 | 0 | 0 | 1 |  |
| **Age group (years)** | | | | | 0.0571 |
| 18 to 64 | 93 (83.0%) | 58 (85.3%) | 282 (91.0%) | 37 (94.9%) |  |
| 65 to 90 | 19 (17.0%) | 10 (14.7%) | 28 (9.0%) | 2 (5.1%) |  |
| Missing | 0 | 0 | 0 | 1 |  |
| **Gender** | | | | | 0.5539 |
| Female | 48 (42.9%) | 35 (51.5%) | 152 (49.0%) | 17 (42.5%) |  |
| Male | 64 (57.1%) | 33 (48.5%) | 158 (51.0%) | 23 (57.5%) |  |
| **First recorded weight** | | | | | 0.8207 |
| Mean (SD) | 75.32 (17.794) | 73.39 (18.561) | 74.02 (18.113) | 72.60 (13.134) |  |
| Missing | 1 | 0 | 1 | 0 |  |
| **Province/region of treating physician** | | | | | 0.2336 |
| Alberta | 6 (5.4%) | 1 (1.5%) | 15 (4.8%) | 4 (10.0%) |  |
| Atlantic^†^ | 13 (11.6%) | 12 (17.6%) | 24 (7.7%) | 1 (2.5%) |  |
| British Columbia | 17 (15.2%) | 6 (8.8%) | 48 (15.5%) | 2 (5.0%) |  |
| Ontario | 56 (50.0%) | 34 (50.0%) | 165 (53.2%) | 24 (60.0%) |  |
| Quebec | 20 (17.9%) | 15 (22.1%) | 57 (18.4%) | 9 (22.5%) |  |
| Saskatchewan/  Manitoba | 0 (0.0%) | 0 (0.0%) | 1 (0.3%) | 0 (0.0%) |  |
| **Year of initiation of IFX treatment** | | | | | 0.0183 |
| 2015 | 19 (17.0%) | 5 (7.4%) | 65 (21.0%) | 3 (7.5%) |  |
| 2016 | 25 (22.3%) | 10 (14.7%) | 85 (27.4%) | 11 (27.5%) |  |
| 2017 | 34 (30.4%) | 26 (38.2%) | 81 (26.1%) | 13 (32.5%) |  |
| 2018 | 34 (30.4%) | 27 (39.7%) | 79 (25.5%) | 13 (32.5%) |  |
| **Time to first TDM (days)** | | | | | 0.0013 |
| Mean (SD) | 397.6 (321.09) | 300.4 (286.04) | 469.5 (366.11) | 360.9 (355.09) |  |
| **Year of first instance of TDM** | | | | | 0.9118 |
| 2015 | 0 (0.0%) | 0 (0.0%) | 0 (0.0%) | 0 (0.0%) |  |
| 2016 | 0 (0.0%) | 0 (0.0%) | 0 (0.0%) | 0 (0.0%) |  |
| 2017 | 40 (35.7%) | 21 (30.9%) | 113 (36.5%) | 15 (37.5%) |  |
| 2018 | 50 (44.6%) | 30 (44.1%) | 136 (43.9%) | 15 (37.5%) |  |
| 2019 | 22 (19.6%) | 17 (25.0%) | 61 (19.7%) | 10 (25.0%) |  |

TDM=therapeutic drug monitoring. P-value: categorical variables, chi-square test; numeric variables, ANOVA.

^†^Atlantic includes New Brunswick, Nova Scotia, Prince Edward Island, Newfoundland, and Labrador.

Dose optimization thresholds: High: A treatment interval decrease of ≥11 days (1.57 weeks) with a posterior interval of ≤35 days (5 weeks), and/or a dose level increase of ≥1.5 mg/kg with a posterior dose level of ≥9 mg/kg. Low: A treatment interval decrease of ≥11 days (1.57 weeks) with a posterior interval of ≤46 days (6.57 weeks), and/or dose level increase of ≥1.5 mg/kg with a posterior dose level of ≥7 mg/kg.

A=First TDM serum IFX concentration below threshold and no dose optimization within post-index period;

B=First TDM serum IFX concentration below threshold and dose optimization within post-index period;

C=First TDM serum IFX concentration at or above threshold and no dose optimization within post-index period;

D=First TDM serum IFX concentration at or above threshold and dose optimization within post-index period.

Suppl Table 8b: Subgroup Demographics and Baseline Characteristics of Patients with UC with a DO (Based on Low Threshold Criteria) Prior to their First Instance of TDM (Serum IFX Threshold: 3 μg/mL; Post-Index Period for Dose Optimization: +17 weeks; Post-Index Dose Optimization Threshold: Low)

|  | **A (N=98)** | **B (N=82)** | **C (N=277)** | **D (N=73)** | **p-value** |
| --- | --- | --- | --- | --- | --- |
| **Age (years)** | | | | | 0.0112 |
| Mean (SD) | 45.8 (16.99) | 43.0 (16.30) | 41.6 (15.35) | 37.8 (16.40) |  |
| Missing | 0 | 0 | 0 | 1 |  |
| **Age group (years)** | | | | | 0.0486 |
| 18 to 64 | 80 (81.6%) | 71 (86.6%) | 253 (91.3%) | 66 (91.7%) |  |
| 65 to 90 | 18 (18.4%) | 11 (13.4%) | 24 (8.7%) | 6 (8.3%) |  |
| Missing | 0 | 0 | 0 | 1 |  |
| **Gender** | | | | | 0.5074 |
| Female | 41 (41.8%) | 42 (51.2%) | 131 (47.3%) | 38 (52.1%) |  |
| Male | 57 (58.2%) | 40 (48.8%) | 146 (52.7%) | 35 (47.9%) |  |
| **First recorded weight** | | | | | 0.3238 |
| Mean (SD) | 76.01 (18.011) | 72.90 (18.085) | 74.52 (18.245) | 71.27 (14.712) |  |
| Missing | 1 | 0 | 0 | 1 |  |
| **Province/region of treating physician** | | | | | 0.0782 |
| Alberta | 6 (6.1%) | 1 (1.2%) | 11 (4.0%) | 8 (11.0%) |  |
| Atlantic^†^ | 12 (12.2%) | 13 (15.9%) | 22 (7.9%) | 3 (4.1%) |  |
| British Columbia | 16 (16.3%) | 7 (8.5%) | 44 (15.9%) | 6 (8.2%) |  |
| Ontario | 48 (49.0%) | 42 (51.2%) | 149 (53.8%) | 40 (54.8%) |  |
| Quebec | 16 (16.3%) | 19 (23.2%) | 50 (18.1%) | 16 (21.9%) |  |
| Saskatchewan/  Manitoba | 0 (0.0%) | 0 (0.0%) | 1 (0.4%) | 0 (0.0%) |  |
| **Year of initiation of IFX treatment** | | | | | 0.0206 |
| 2015 | 16 (16.3%) | 8 (9.8%) | 59 (21.3%) | 9 (12.3%) |  |
| 2016 | 24 (24.5%) | 11 (13.4%) | 75 (27.1%) | 21 (28.8%) |  |
| 2017 | 29 (29.6%) | 31 (37.8%) | 75 (27.1%) | 19 (26.0%) |  |
| 2018 | 29 (29.6%) | 32 (39.0%) | 68 (24.5%) | 24 (32.9%) |  |
| **Time to first TDM (days)** | | | | | 0.0010 |
| Mean (SD) | 406.4 (315.64) | 306.5 (298.48) | 475.3 (369.65) | 388.1 (345.62) |  |
| **Year of first instance of TDM** | | | | | 0.9376 |
| 2015 | 0 (0.0%) | 0 (0.0%) | 0 (0.0%) | 0 (0.0%) |  |
| 2016 | 0 (0.0%) | 0 (0.0%) | 0 (0.0%) | 0 (0.0%) |  |
| 2017 | 34 (34.7%) | 27 (32.9%) | 101 (36.5%) | 27 (37.0%) |  |
| 2018 | 43 (43.9%) | 37 (45.1%) | 123 (44.4%) | 28 (38.4%) |  |
| 2019 | 21 (21.4%) | 18 (22.0%) | 53 (19.1%) | 18 (24.7%) |  |

TDM=therapeutic drug monitoring. P-value: categorical variables, chi-square test; numeric variables, ANOVA.

^†^Atlantic includes New Brunswick, Nova Scotia, Prince Edward Island, Newfoundland, and Labrador.

Dose optimization thresholds: High: A treatment interval decrease of ≥11 days (1.57 weeks) with a posterior interval of ≤35 days (5 weeks), and/or a dose level increase of ≥1.5 mg/kg with a posterior dose level of ≥9 mg/kg. Low: A treatment interval decrease of ≥11 days (1.57 weeks) with a posterior interval of ≤46 days (6.57 weeks), and/or dose level increase of ≥1.5 mg/kg with a posterior dose level of ≥7 mg/kg.

A=First TDM serum IFX concentration below threshold and no dose optimization within post-index period;

B=First TDM serum IFX concentration below threshold and dose optimization within post-index period;

C=First TDM serum IFX concentration at or above threshold and no dose optimization within post-index period;

D=First TDM serum IFX concentration at or above threshold and dose optimization within post-index period.

Suppl Table 8c: Subgroup Demographics and Baseline Characteristics of Patients with UC with a DO (Based on Low Threshold Criteria) Prior to their First Instance of TDM (Serum IFX Threshold: 5 μg/mL; Post-Index Period for Dose Optimization: +9 weeks; Post-Index Dose Optimization Threshold: Low)

|  | **A (N=154)** | **B (N=79)** | **C (N=268)** | **D (N=29)** | **p-value** |
| --- | --- | --- | --- | --- | --- |
| **Age (years)** | | | | | 0.027 |
| Mean (SD) | 45.3 (16.70) | 41.6 (16.44) | 40.4 (15.26) | 41.4 (17.43) |  |
| Missing | 0 | 0 | 0 | 1 |  |
| **Age group (years)** | | | | | 0.0722 |
| 18 to 64 | 129 (83.8%) | 69 (87.3%) | 246 (91.8%) | 26 (92.9%) |  |
| 65 to 90 | 25 (16.2%) | 10 (12.7%) | 22 (8.2%) | 2 (7.1%) |  |
| Missing | 0 | 0 | 0 | 1 |  |
| **Gender** | | | | | 0.1742 |
| Female | 62 (40.3%) | 38 (48.1%) | 138 (51.5%) | 14 (48.3%) |  |
| Male | 92 (59.7%) | 41 (51.9%) | 130 (48.5%) | 15 (51.7%) |  |
| **First recorded weight** | | | | | 0.4498 |
| Mean (SD) | 75.98 (17.530) | 73.60 (17.572) | 73.44 (18.256) | 71.72 (14.227) |  |
| Missing | 2 | 0 | 0 | 0 |  |
| **Province/region of treating physician** | | | | | 0.4647 |
| Alberta | 8 (5.2%) | 2 (2.5%) | 13 (4.9%) | 3 (10.3%) |  |
| Atlantic^†^ | 16 (10.4%) | 12 (15.2%) | 21 (7.8%) | 1 (3.4%) |  |
| British Columbia | 25 (16.2%) | 6 (7.6%) | 40 (14.9%) | 2 (6.9%) |  |
| Ontario | 74 (48.1%) | 41 (51.9%) | 147 (54.9%) | 17 (58.6%) |  |
| Quebec | 31 (20.1%) | 18 (22.8%) | 46 (17.2%) | 6 (20.7%) |  |
| Saskatchewan/  Manitoba | 0 (0.0%) | 0 (0.0%) | 1 (0.4%) | 0 (0.0%) |  |
| **Year of initiation of IFX treatment** | | | | | 0.0153 |
| 2015 | 33 (21.4%) | 7 (8.9%) | 51 (19.0%) | 1 (3.4%) |  |
| 2016 | 35 (22.7%) | 12 (15.2%) | 75 (28.0%) | 9 (31.0%) |  |
| 2017 | 41 (26.6%) | 28 (35.4%) | 74 (27.6%) | 11 (37.9%) |  |
| 2018 | 45 (29.2%) | 32 (40.5%) | 68 (25.4%) | 8 (27.6%) |  |
| **Time to first TDM (days)** | | | | | 0.0090 |
| Mean (SD) | 449.5 (353.64) | 315.1 (313.41) | 451.0 (357.64) | 343.9 (316.91) |  |
| **Year of first instance of TDM** | | | | | 0.5305 |
| 2015 | 0 (0.0%) | 0 (0.0%) | 0 (0.0%) | 0 (0.0%) |  |
| 2016 | 0 (0.0%) | 0 (0.0%) | 0 (0.0%) | 0 (0.0%) |  |
| 2017 | 54 (35.1%) | 23 (29.1%) | 99 (36.9%) | 13 (44.8%) |  |
| 2018 | 70 (45.5%) | 37 (46.8%) | 116 (43.3%) | 8 (27.6%) |  |
| 2019 | 30 (19.5%) | 19 (24.1%) | 53 (19.8%) | 8 (27.6%) |  |

TDM=therapeutic drug monitoring. P-value: categorical variables, chi-square test; numeric variables, ANOVA.

^†^Atlantic includes New Brunswick, Nova Scotia, Prince Edward Island, Newfoundland, and Labrador.

Dose optimization thresholds: High: A treatment interval decrease of ≥11 days (1.57 weeks) with a posterior interval of ≤35 days (5 weeks), and/or a dose level increase of ≥1.5 mg/kg with a posterior dose level of ≥9 mg/kg. Low: A treatment interval decrease of ≥11 days (1.57 weeks) with a posterior interval of ≤46 days (6.57 weeks), and/or dose level increase of ≥1.5 mg/kg with a posterior dose level of ≥7 mg/kg.

A=First TDM serum IFX concentration below threshold and no dose optimization within post-index period;

B=First TDM serum IFX concentration below threshold and dose optimization within post-index period;

C=First TDM serum IFX concentration at or above threshold and no dose optimization within post-index period;

D=First TDM serum IFX concentration at or above threshold and dose optimization within post-index period.

Suppl Table 8d: Subgroup Demographics and Baseline Characteristics of Patients with UC with a DO (Based on Low Threshold Criteria) Prior to their First Instance of TDM (Serum IFX Threshold: 5 μg/mL; Post-Index Period for Dose Optimization: +17 weeks; Post-Index Dose Optimization Threshold: Low)

|  | **A (N=135)** | **B (N=98)** | **C (N=240)** | **D (N=57)** | **p-value** |
| --- | --- | --- | --- | --- | --- |
| **Age (years)** | | | | | 0.0115 |
| Mean (SD) | 45.7 (16.72) | 41.7 (16.38) | 41.0 (15.15) | 38.5 (16.65) |  |
| Missing | 0 | 0 | 0 | 1 |  |
| **Age group (years)** | | | | | 0.0538 |
| 18 to 64 | 112 (83.0%) | 86 (87.8%) | 221 (92.1%) | 51 (91.1%) |  |
| 65 to 90 | 23 (17.0%) | 12 (12.2%) | 19 (7.9%) | 5 (8.9%) |  |
| Missing | 0 | 0 | 0 | 1 |  |
| **Gender** | | | | | 0.0869 |
| Female | 53 (39.3%) | 47 (48.0%) | 119 (49.6%) | 33 (57.9%) |  |
| Male | 82 (60.7%) | 51 (52.0%) | 121 (50.4%) | 24 (42.1%) |  |
| **First recorded weight** | | | | | 0.1404 |
| Mean (SD) | 76.55 (17.596) | 73.25 (17.377) | 73.99 (18.459) | 70.26 (15.032) |  |
| Missing | 1 | 1 | 0 | 0 |  |
| **Province/region of treating physician** | | | | | 0.3214 |
| Alberta | 7 (5.2%) | 3 (3.1%) | 10 (4.2%) | 6 (10.5%) |  |
| Atlantic^†^ | 15 (11.1%) | 13 (13.3%) | 19 (7.9%) | 3 (5.3%) |  |
| British Columbia | 22 (16.3%) | 9 (9.2%) | 38 (15.8%) | 4 (7.0%) |  |
| Ontario | 65 (48.1%) | 50 (51.0%) | 132 (55.0%) | 32 (56.1%) |  |
| Quebec | 26 (19.3%) | 23 (23.5%) | 40 (16.7%) | 12 (21.1%) |  |
| Saskatchewan/  Manitoba | 0 (0.0%) | 0 (0.0%) | 1 (0.4%) | 0 (0.0%) |  |
| **Year of initiation of IFX treatment** | | | | | 0.0419 |
| 2015 | 29 (21.5%) | 11 (11.2%) | 46 (19.2%) | 6 (10.5%) |  |
| 2016 | 32 (23.7%) | 15 (15.3%) | 67 (27.9%) | 17 (29.8%) |  |
| 2017 | 35 (25.9%) | 34 (34.7%) | 69 (28.8%) | 16 (28.1%) |  |
| 2018 | 39 (28.9%) | 38 (38.8%) | 58 (24.2%) | 18 (31.6%) |  |
| **Time to first TDM (days)** | | | | | 0.0075 |
| Mean (SD) | 459.4 (351.27) | 327.5 (324.57) | 456.1 (361.22) | 374.9 (321.12) |  |
| **Year of first instance of TDM** | | | | | 0.6903 |
| 2015 | 0 (0.0%) | 0 (0.0%) | 0 (0.0%) | 0 (0.0%) |  |
| 2016 | 0 (0.0%) | 0 (0.0%) | 0 (0.0%) | 0 (0.0%) |  |
| 2017 | 46 (34.1%) | 31 (31.6%) | 89 (37.1%) | 23 (40.4%) |  |
| 2018 | 61 (45.2%) | 46 (46.9%) | 105 (43.8%) | 19 (33.3%) |  |
| 2019 | 28 (20.7%) | 21 (21.4%) | 46 (19.2%) | 15 (26.3%) |  |

TDM=therapeutic drug monitoring. P-value: categorical variables, chi-square test; numeric variables, ANOVA.

^†^Atlantic includes New Brunswick, Nova Scotia, Prince Edward Island, Newfoundland, and Labrador.

Dose optimization thresholds: High: A treatment interval decrease of ≥11 days (1.57 weeks) with a posterior interval of ≤35 days (5 weeks), and/or a dose level increase of ≥1.5 mg/kg with a posterior dose level of ≥9 mg/kg. Low: A treatment interval decrease of ≥11 days (1.57 weeks) with a posterior interval of ≤46 days (6.57 weeks), and/or dose level increase of ≥1.5 mg/kg with a posterior dose level of ≥7 mg/kg.

A=First TDM serum IFX concentration below threshold and no dose optimization within post-index period;

B=First TDM serum IFX concentration below threshold and dose optimization within post-index period;

C=First TDM serum IFX concentration at or above threshold and no dose optimization within post-index period;

D=First TDM serum IFX concentration at or above threshold and dose optimization within post-index period.

Suppl Table 8e: Subgroup Demographics and Baseline Characteristics of Patients with UC with a DO (Based on Low Threshold Criteria) Prior to their First Instance of TDM (Serum IFX Threshold: 10 μg/mL; Post-Index Period for Dose Optimization: +9 weeks; Post-Index Dose Optimization Threshold: Low)

|  | **A (N=246)** | **B (N=99)** | **C (N=176)** | **D (N=9)** | **p-value** |
| --- | --- | --- | --- | --- | --- |
| **Age (years)** | | | | | 0.0543 |
| Mean (SD) | 43.8 (16.12) | 41.1 (16.75) | 39.9 (15.47) | 47.6 (14.56) |  |
| Missing | 0 | 0 | 0 | 1 |  |
| **Age group (years)** | | | | | 0.5523 |
| 18 to 64 | 214 (87.0%) | 88 (88.9%) | 161 (91.5%) | 7 (87.5%) |  |
| 65 to 90 | 32 (13.0%) | 11 (11.1%) | 15 (8.5%) | 1 (12.5%) |  |
| Missing | 0 | 0 | 0 | 1 |  |
| **Gender** | | | | | 0.2879 |
| Female | 108 (43.9%) | 49 (49.5%) | 92 (52.3%) | 3 (33.3%) |  |
| Male | 138 (56.1%) | 50 (50.5%) | 84 (47.7%) | 6 (66.7%) |  |
| **First recorded weight** | | | | | 0.6209 |
| Mean (SD) | 74.86 (18.337) | 72.62 (17.036) | 73.67 (17.593) | 78.42 (11.763) |  |
| Missing | 2 | 0 | 0 | 0 |  |
| **Province/region of treating physician** | | | | | 0.0902 |
| Alberta | 14 (5.7%) | 3 (3.0%) | 7 (4.0%) | 2 (22.2%) |  |
| Atlantic^†^ | 21 (8.5%) | 13 (13.1%) | 16 (9.1%) | 0 (0.0%) |  |
| British Columbia | 39 (15.9%) | 6 (6.1%) | 26 (14.8%) | 2 (22.2%) |  |
| Ontario | 120 (48.8%) | 56 (56.6%) | 101 (57.4%) | 2 (22.2%) |  |
| Quebec | 51 (20.7%) | 21 (21.2%) | 26 (14.8%) | 3 (33.3%) |  |
| Saskatchewan/  Manitoba | 1 (0.4%) | 0 (0.0%) | 0 (0.0%) | 0 (0.0%) |  |
| **Year of initiation of IFX treatment** | | | | | 0.0068 |
| 2015 | 56 (22.8%) | 8 (8.1%) | 28 (15.9%) | 0 (0.0%) |  |
| 2016 | 64 (26.0%) | 17 (17.2%) | 46 (26.1%) | 4 (44.4%) |  |
| 2017 | 60 (24.4%) | 36 (36.4%) | 55 (31.3%) | 3 (33.3%) |  |
| 2018 | 66 (26.8%) | 38 (38.4%) | 47 (26.7%) | 2 (22.2%) |  |
| **Time to first TDM (days)** | | | | | 0.0040 |
| Mean (SD) | 470.2 (364.02) | 322.7 (321.67) | 422.9 (343.03) | 323.9 (209.51) |  |
| **Year of first instance of TDM** | | | | | 0.5823 |
| 2015 | 0 (0.0%) | 0 (0.0%) | 0 (0.0%) | 0 (0.0%) |  |
| 2016 | 0 (0.0%) | 0 (0.0%) | 0 (0.0%) | 0 (0.0%) |  |
| 2017 | 92 (37.4%) | 31 (31.3%) | 61 (34.7%) | 5 (55.6%) |  |
| 2018 | 104 (42.3%) | 42 (42.4%) | 82 (46.6%) | 3 (33.3%) |  |
| 2019 | 50 (20.3%) | 26 (26.3%) | 33 (18.8%) | 1 (11.1%) |  |

TDM=therapeutic drug monitoring. P-value: categorical variables, chi-square test; numeric variables, ANOVA.

^†^Atlantic includes New Brunswick, Nova Scotia, Prince Edward Island, Newfoundland, and Labrador.

Dose optimization thresholds: High: A treatment interval decrease of ≥11 days (1.57 weeks) with a posterior interval of ≤35 days (5 weeks), and/or a dose level increase of ≥1.5 mg/kg with a posterior dose level of ≥9 mg/kg. Low: A treatment interval decrease of ≥11 days (1.57 weeks) with a posterior interval of ≤46 days (6.57 weeks), and/or dose level increase of ≥1.5 mg/kg with a posterior dose level of ≥7 mg/kg.

A=First TDM serum IFX concentration below threshold and no dose optimization within post-index period;

B=First TDM serum IFX concentration below threshold and dose optimization within post-index period;

C=First TDM serum IFX concentration at or above threshold and no dose optimization within post-index period;

D=First TDM serum IFX concentration at or above threshold and dose optimization within post-index period.

Suppl Table 8f: Subgroup Demographics and Baseline Characteristics of Patients with UC with a DO (Based on Low Threshold Criteria) Prior to their First Instance of TDM (Serum IFX Threshold: 10 μg/mL; Post-Index Period for Dose Optimization: +17 weeks; Post-Index Dose Optimization Threshold: Low)

|  | **A (N=216)** | **B (N=129)** | **C (N=159)** | **D (N=26)** | **p-value** |
| --- | --- | --- | --- | --- | --- |
| **Age (years)** | | | | | 0.0524 |
| Mean (SD) | 44.3 (16.06) | 40.9 (16.60) | 40.4 (15.40) | 38.8 (16.19) |  |
| Missing | 0 | 0 | 0 | 1 |  |
| **Age group (years)** | | | | | 0.4614 |
| 18 to 64 | 187 (86.6%) | 115 (89.1%) | 146 (91.8%) | 22 (88.0%) |  |
| 65 to 90 | 29 (13.4%) | 14 (10.9%) | 13 (8.2%) | 3 (12.0%) |  |
| Missing | 0 | 0 | 0 | 1 |  |
| **Gender** | | | | | 0.2308 |
| Female | 93 (43.1%) | 64 (49.6%) | 79 (49.7%) | 16 (61.5%) |  |
| Male | 123 (56.9%) | 65 (50.4%) | 80 (50.3%) | 10 (38.5%) |  |
| **First recorded weight** | | | | | 0.3851 |
| Mean (SD) | 75.39 (18.381) | 72.23 (17.160) | 74.26 (17.923) | 71.69 (13.505) |  |
| Missing | 1 | 1 | 0 | 0 |  |
| **Province/region of treating physician** | | | | | 0.0159 |
| Alberta | 13 (6.0%) | 4 (3.1%) | 4 (2.5%) | 5 (19.2%) |  |
| Atlantic^†^ | 19 (8.8%) | 15 (11.6%) | 15 (9.4%) | 1 (3.8%) |  |
| British Columbia | 36 (16.7%) | 9 (7.0%) | 24 (15.1%) | 4 (15.4%) |  |
| Ontario | 104 (48.1%) | 72 (55.8%) | 93 (58.5%) | 10 (38.5%) |  |
| Quebec | 43 (19.9%) | 29 (22.5%) | 23 (14.5%) | 6 (23.1%) |  |
| Saskatchewan/  Manitoba | 1 (0.5%) | 0 (0.0%) | 0 (0.0%) | 0 (0.0%) |  |
| **Year of initiation of IFX treatment** | | | | | 0.0084 |
| 2015 | 51 (23.6%) | 13 (10.1%) | 24 (15.1%) | 4 (15.4%) |  |
| 2016 | 58 (26.9%) | 23 (17.8%) | 41 (25.8%) | 9 (34.6%) |  |
| 2017 | 53 (24.5%) | 43 (33.3%) | 51 (32.1%) | 7 (26.9%) |  |
| 2018 | 54 (25.0%) | 50 (38.8%) | 43 (27.0%) | 6 (23.1%) |  |
| **Time to first TDM (days)** | | | | | 0.0011 |
| Mean (SD) | 486.1 (364.74) | 330.4 (325.70) | 418.2 (343.98) | 417.3 (305.44) |  |
| **Year of first instance of TDM** | | | | | 0.5884 |
| 2015 | 0 (0.0%) | 0 (0.0%) | 0 (0.0%) | 0 (0.0%) |  |
| 2016 | 0 (0.0%) | 0 (0.0%) | 0 (0.0%) | 0 (0.0%) |  |
| 2017 | 81 (37.5%) | 42 (32.6%) | 54 (34.0%) | 12 (46.2%) |  |
| 2018 | 92 (42.6%) | 54 (41.9%) | 74 (46.5%) | 11 (42.3%) |  |
| 2019 | 43 (19.9%) | 33 (25.6%) | 31 (19.5%) | 3 (11.5%) |  |

TDM=therapeutic drug monitoring. P-value: categorical variables, chi-square test; numeric variables, ANOVA.

^†^Atlantic includes New Brunswick, Nova Scotia, Prince Edward Island, Newfoundland, and Labrador.

Dose optimization thresholds: High: A treatment interval decrease of ≥11 days (1.57 weeks) with a posterior interval of ≤35 days (5 weeks), and/or a dose level increase of ≥1.5 mg/kg with a posterior dose level of ≥9 mg/kg. Low: A treatment interval decrease of ≥11 days (1.57 weeks) with a posterior interval of ≤46 days (6.57 weeks), and/or dose level increase of ≥1.5 mg/kg with a posterior dose level of ≥7 mg/kg.

A=First TDM serum IFX concentration below threshold and no dose optimization within post-index period;

B=First TDM serum IFX concentration below threshold and dose optimization within post-index period;

C=First TDM serum IFX concentration at or above threshold and no dose optimization within post-index period;

D=First TDM serum IFX concentration at or above threshold and dose optimization within post-index period.

Suppl Table 8g: Subgroup Demographics and Baseline Characteristics of Patients with UC with a DO (Based on Low Threshold Criteria) Prior to their First Instance of TDM (Serum IFX Threshold: 3 μg/mL; Post-Index Period for Dose Optimization: +9 weeks; Post-Index Dose Optimization Threshold: High)

|  | **A (N=127)** | **B (N=53)** | **C (N=323)** | **D (N=27)** | **p-value** |
| --- | --- | --- | --- | --- | --- |
| **Age (years)** | | | | | 0.0730 |
| Mean (SD) | 45.1 (16.71) | 43.2 (16.74) | 40.8 (15.46) | 40.5 (17.89) |  |
| Missing | 0 | 0 | 0 | 1 |  |
| **Age group (years)** | | | | | 0.0594 |
| 18 to 64 | 105 (82.7%) | 46 (86.8%) | 295 (91.3%) | 24 (92.3%) |  |
| 65 to 90 | 22 (17.3%) | 7 (13.2%) | 28 (8.7%) | 2 (7.7%) |  |
| Missing | 0 | 0 | 0 | 1 |  |
| **Gender** | | | | | 0.7766 |
| Female | 56 (44.1%) | 27 (50.9%) | 157 (48.6%) | 12 (44.4%) |  |
| Male | 71 (55.9%) | 26 (49.1%) | 166 (51.4%) | 15 (55.6%) |  |
| **First recorded weight** | | | | | 0.4758 |
| Mean (SD) | 75.80 (18.163) | 71.69 (17.649) | 74.00 (17.822) | 72.07 (14.935) |  |
| Missing | 1 | 0 | 1 | 0 |  |
| **Province/region of treating physician** | | | | | 0.5538 |
| Alberta | 6 (4.7%) | 1 (1.9%) | 17 (5.3%) | 2 (7.4%) |  |
| Atlantic^†^ | 16 (12.6%) | 9 (17.0%) | 24 (7.4%) | 1 (3.7%) |  |
| British Columbia | 18 (14.2%) | 5 (9.4%) | 48 (14.9%) | 2 (7.4%) |  |
| Ontario | 60 (47.2%) | 30 (56.6%) | 171 (52.9%) | 18 (66.7%) |  |
| Quebec | 27 (21.3%) | 8 (15.1%) | 62 (19.2%) | 4 (14.8%) |  |
| Saskatchewan/  Manitoba | 0 (0.0%) | 0 (0.0%) | 1 (0.3%) | 0 (0.0%) |  |
| **Year of initiation of IFX treatment** | | | | | 0.0046 |
| 2015 | 21 (16.5%) | 3 (5.7%) | 67 (20.7%) | 1 (3.7%) |  |
| 2016 | 28 (22.0%) | 7 (13.2%) | 89 (27.6%) | 7 (25.9%) |  |
| 2017 | 40 (31.5%) | 20 (37.7%) | 87 (26.9%) | 7 (25.9%) |  |
| 2018 | 38 (29.9%) | 23 (43.4%) | 80 (24.8%) | 12 (44.4%) |  |
| **Time to first TDM (days)** | | | | | <0.0001 |
| Mean (SD) | 400.4 (322.02) | 266.3 (262.66) | 471.1 (368.36) | 289.8 (293.62) |  |
| **Year of first instance of TDM** | | | | | 0.8062 |
| 2015 | 0 (0.0%) | 0 (0.0%) | 0 (0.0%) | 0 (0.0%) |  |
| 2016 | 0 (0.0%) | 0 (0.0%) | 0 (0.0%) | 0 (0.0%) |  |
| 2017 | 46 (36.2%) | 15 (28.3%) | 119 (36.8%) | 9 (33.3%) |  |
| 2018 | 54 (42.5%) | 26 (49.1%) | 141 (43.7%) | 10 (37.0%) |  |
| 2019 | 27 (21.3%) | 12 (22.6%) | 63 (19.5%) | 8 (29.6%) |  |

TDM=therapeutic drug monitoring. P-value: categorical variables, chi-square test; numeric variables, ANOVA.

^†^Atlantic includes New Brunswick, Nova Scotia, Prince Edward Island, Newfoundland, and Labrador.

Dose optimization thresholds: High: A treatment interval decrease of ≥11 days (1.57 weeks) with a posterior interval of ≤35 days (5 weeks), and/or a dose level increase of ≥1.5 mg/kg with a posterior dose level of ≥9 mg/kg. Low: A treatment interval decrease of ≥11 days (1.57 weeks) with a posterior interval of ≤46 days (6.57 weeks), and/or dose level increase of ≥1.5 mg/kg with a posterior dose level of ≥7 mg/kg.

A=First TDM serum IFX concentration below threshold and no dose optimization within post-index period;

B=First TDM serum IFX concentration below threshold and dose optimization within post-index period;

C=First TDM serum IFX concentration at or above threshold and no dose optimization within post-index period;

D=First TDM serum IFX concentration at or above threshold and dose optimization within post-index period.

Suppl Table 8h: Subgroup Demographics and Baseline Characteristics of Patients with UC with a DO (Based on Low Threshold Criteria) Prior to their First Instance of TDM (Serum IFX Threshold: 3 μg/mL; Post-Index Period for Dose Optimization: +17 weeks; Post-Index Dose Optimization Threshold: High)

|  | **A (N=109)** | **B (N=71)** | **C (N=305)** | **D (N=45)** | **p-value** |
| --- | --- | --- | --- | --- | --- |
| **Age (years)** | | | | | 0.0087 |
| Mean (SD) | 45.8 (17.11) | 42.5 (15.95) | 41.4 (15.56) | 36.6 (15.58) |  |
| Missing | 0 | 0 | 0 | 1 |  |
| **Age group (years)** | | | | | 0.0293 |
| 18 to 64 | 89 (81.7%) | 62 (87.3%) | 277 (90.8%) | 42 (95.5%) |  |
| 65 to 90 | 20 (18.3%) | 9 (12.7%) | 28 (9.2%) | 2 (4.5%) |  |
| Missing | 0 | 0 | 0 | 1 |  |
| **Gender** | | | | | 0.6270 |
| Female | 47 (43.1%) | 36 (50.7%) | 145 (47.5%) | 24 (53.3%) |  |
| Male | 62 (56.9%) | 35 (49.3%) | 160 (52.5%) | 21 (46.7%) |  |
| **First recorded weight** | | | | | 0.2362 |
| Mean (SD) | 76.32 (18.304) | 71.95 (17.484) | 74.29 (18.016) | 70.88 (14.335) |  |
| Missing | 1 | 0 | 1 | 0 |  |
| **Province/region of treating physician** | | | | | 0.4491 |
| Alberta | 6 (5.5%) | 1 (1.4%) | 14 (4.6%) | 5 (11.1%) |  |
| Atlantic^†^ | 14 (12.8%) | 11 (15.5%) | 23 (7.5%) | 2 (4.4%) |  |
| British Columbia | 16 (14.7%) | 7 (9.9%) | 45 (14.8%) | 5 (11.1%) |  |
| Ontario | 52 (47.7%) | 38 (53.5%) | 163 (53.4%) | 26 (57.8%) |  |
| Quebec | 21 (19.3%) | 14 (19.7%) | 59 (19.3%) | 7 (15.6%) |  |
| Saskatchewan/  Manitoba | 0 (0.0%) | 0 (0.0%) | 1 (0.3%) | 0 (0.0%) |  |
| **Year of initiation of IFX treatment** | | | | | 0.0135 |
| 2015 | 17 (15.6%) | 7 (9.9%) | 63 (20.7%) | 5 (11.1%) |  |
| 2016 | 26 (23.9%) | 9 (12.7%) | 85 (27.9%) | 11 (24.4%) |  |
| 2017 | 34 (31.2%) | 26 (36.6%) | 83 (27.2%) | 11 (24.4%) |  |
| 2018 | 32 (29.4%) | 29 (40.8%) | 74 (24.3%) | 18 (40.0%) |  |
| **Time to first TDM (days)** | | | | | 0.0005 |
| Mean (SD) | 398.8 (314.52) | 302.7 (298.70) | 473.5 (369.83) | 345.9 (321.03) |  |
| **Year of first instance of TDM** | | | | | 0.8166 |
| 2015 | 0 (0.0%) | 0 (0.0%) | 0 (0.0%) | 0 (0.0%) |  |
| 2016 | 0 (0.0%) | 0 (0.0%) | 0 (0.0%) | 0 (0.0%) |  |
| 2017 | 40 (36.7%) | 21 (29.6%) | 113 (37.0%) | 15 (33.3%) |  |
| 2018 | 45 (41.3%) | 35 (49.3%) | 133 (43.6%) | 18 (40.0%) |  |
| 2019 | 24 (22.0%) | 15 (21.1%) | 59 (19.3%) | 12 (26.7%) |  |

TDM=therapeutic drug monitoring. P-value: categorical variables, chi-square test; numeric variables, ANOVA.

^†^Atlantic includes New Brunswick, Nova Scotia, Prince Edward Island, Newfoundland, and Labrador.

Dose optimization thresholds: High: A treatment interval decrease of ≥11 days (1.57 weeks) with a posterior interval of ≤35 days (5 weeks), and/or a dose level increase of ≥1.5 mg/kg with a posterior dose level of ≥9 mg/kg. Low: A treatment interval decrease of ≥11 days (1.57 weeks) with a posterior interval of ≤46 days (6.57 weeks), and/or dose level increase of ≥1.5 mg/kg with a posterior dose level of ≥7 mg/kg.

A=First TDM serum IFX concentration below threshold and no dose optimization within post-index period;

B=First TDM serum IFX concentration below threshold and dose optimization within post-index period;

C=First TDM serum IFX concentration at or above threshold and no dose optimization within post-index period;

D=First TDM serum IFX concentration at or above threshold and dose optimization within post-index period.

Suppl Table 8i: Subgroup Demographics and Baseline Characteristics of Patients with UC with a DO (Based on Low Threshold Criteria) Prior to their First Instance of TDM (Serum IFX Threshold: 5 μg/mL; Post-Index Period for Dose Optimization: +9 weeks; Post-Index Dose Optimization Threshold: High)

|  | **A (N=174)** | **B (N=59)** | **C (N=276)** | **D (N=21)** | **p-value** |
| --- | --- | --- | --- | --- | --- |
| **Age (years)** | | | | | 0.0537 |
| Mean (SD) | 44.6 (16.76) | 42.3 (16.39) | 40.4 (15.16) | 42.5 (19.35) |  |
| Missing | 0 | 0 | 0 | 1 |  |
| **Age group (years)** | | | | | 0.0670 |
| 18 to 64 | 146 (83.9%) | 52 (88.1%) | 254 (92.0%) | 18 (90.0%) |  |
| 65 to 90 | 28 (16.1%) | 7 (11.9%) | 22 (8.0%) | 2 (10.0%) |  |
| Missing | 0 | 0 | 0 | 1 |  |
| **Gender** | | | | | 0.1779 |
| Female | 71 (40.8%) | 29 (49.2%) | 142 (51.4%) | 10 (47.6%) |  |
| Male | 103 (59.2%) | 30 (50.8%) | 134 (48.6%) | 11 (52.4%) |  |
| **First recorded weight** | | | | | 0.2523 |
| Mean (SD) | 76.12 (17.671) | 72.38 (17.003) | 73.51 (18.027) | 70.24 (16.062) |  |
| Missing | 2 | 0 | 0 | 0 |  |
| **Province/region of treating physician** | | | | | 0.4546 |
| Alberta | 9 (5.2%) | 1 (1.7%) | 14 (5.1%) | 2 (9.5%) |  |
| Atlantic^†^ | 19 (10.9%) | 9 (15.3%) | 21 (7.6%) | 1 (4.8%) |  |
| British Columbia | 26 (14.9%) | 5 (8.5%) | 40 (14.5%) | 2 (9.5%) |  |
| Ontario | 79 (45.4%) | 36 (61.0%) | 152 (55.1%) | 12 (57.1%) |  |
| Quebec | 41 (23.6%) | 8 (13.6%) | 48 (17.4%) | 4 (19.0%) |  |
| Saskatchewan/  Manitoba | 0 (0.0%) | 0 (0.0%) | 1 (0.4%) | 0 (0.0%) |  |
| **Year of initiation of IFX treatment** | | | | | 0.0066 |
| 2015 | 37 (21.3%) | 3 (5.1%) | 51 (18.5%) | 1 (4.8%) |  |
| 2016 | 39 (22.4%) | 8 (13.6%) | 78 (28.3%) | 6 (28.6%) |  |
| 2017 | 48 (27.6%) | 21 (35.6%) | 79 (28.6%) | 6 (28.6%) |  |
| 2018 | 50 (28.7%) | 27 (45.8%) | 68 (24.6%) | 8 (38.1%) |  |
| **Time to first TDM (days)** | | | | | 0.0004 |
| Mean (SD) | 453.8 (359.30) | 256.9 (252.55) | 449.5 (356.13) | 322.8 (321.92) |  |
| **Year of first instance of TDM** | | | | | 0.5093 |
| 2015 | 0 (0.0%) | 0 (0.0%) | 0 (0.0%) | 0 (0.0%) |  |
| 2016 | 0 (0.0%) | 0 (0.0%) | 0 (0.0%) | 0 (0.0%) |  |
| 2017 | 61 (35.1%) | 16 (27.1%) | 104 (37.7%) | 8 (38.1%) |  |
| 2018 | 77 (44.3%) | 30 (50.8%) | 118 (42.8%) | 6 (28.6%) |  |
| 2019 | 36 (20.7%) | 13 (22.0%) | 54 (19.6%) | 7 (33.3%) |  |

TDM=therapeutic drug monitoring. P-value: categorical variables, chi-square test; numeric variables, ANOVA.

^†^Atlantic includes New Brunswick, Nova Scotia, Prince Edward Island, Newfoundland, and Labrador.

Dose optimization thresholds: High: A treatment interval decrease of ≥11 days (1.57 weeks) with a posterior interval of ≤35 days (5 weeks), and/or a dose level increase of ≥1.5 mg/kg with a posterior dose level of ≥9 mg/kg. Low: A treatment interval decrease of ≥11 days (1.57 weeks) with a posterior interval of ≤46 days (6.57 weeks), and/or dose level increase of ≥1.5 mg/kg with a posterior dose level of ≥7 mg/kg.

A=First TDM serum IFX concentration below threshold and no dose optimization within post-index period;

B=First TDM serum IFX concentration below threshold and dose optimization within post-index period;

C=First TDM serum IFX concentration at or above threshold and no dose optimization within post-index period;

D=First TDM serum IFX concentration at or above threshold and dose optimization within post-index period.

Suppl Table 8j: Subgroup Demographics and Baseline Characteristics of Patients with UC with a DO (Based on Low Threshold Criteria) Prior to their First Instance of TDM (Serum IFX Threshold: 5 μg/mL; Post-Index Period for Dose Optimization: +17 weeks; Post-Index Dose Optimization Threshold: High)

|  | **A (N=155)** | **B (N=78)** | **C (N=259)** | **D (N=38)** | **p-value** |
| --- | --- | --- | --- | --- | --- |
| **Age (years)** | | | | | 0.0129 |
| Mean (SD) | 45.0 (17.07) | 42.1 (15.76) | 41.1 (15.31) | 36.4 (16.03) |  |
| Missing | 0 | 0 | 0 | 1 |  |
| **Age group (years)** | | | | | 0.0453 |
| 18 to 64 | 129 (83.2%) | 69 (88.5%) | 237 (91.5%) | 35 (94.6%) |  |
| 65 to 90 | 26 (16.8%) | 9 (11.5%) | 22 (8.5%) | 2 (5.4%) |  |
| Missing | 0 | 0 | 0 | 1 |  |
| **Gender** | | | | | 0.1145 |
| Female | 62 (40.0%) | 38 (48.7%) | 130 (50.2%) | 22 (57.9%) |  |
| Male | 93 (60.0%) | 40 (51.3%) | 129 (49.8%) | 16 (42.1%) |  |
| **First recorded weight** | | | | | 0.1086 |
| Mean (SD) | 76.49 (17.763) | 72.56 (16.910) | 73.84 (18.245) | 69.43 (14.891) |  |
| Missing | 2 | 0 | 0 | 0 |  |
| **Province/region of treating physician** | | | | | 0.2996 |
| Alberta | 9 (5.8%) | 1 (1.3%) | 11 (4.2%) | 5 (13.2%) |  |
| Atlantic^†^ | 17 (11.0%) | 11 (14.1%) | 20 (7.7%) | 2 (5.3%) |  |
| British Columbia | 23 (14.8%) | 8 (10.3%) | 38 (14.7%) | 4 (10.5%) |  |
| Ontario | 71 (45.8%) | 44 (56.4%) | 144 (55.6%) | 20 (52.6%) |  |
| Quebec | 35 (22.6%) | 14 (17.9%) | 45 (17.4%) | 7 (18.4%) |  |
| Saskatchewan/  Manitoba | 0 (0.0%) | 0 (0.0%) | 1 (0.4%) | 0 (0.0%) |  |
| **Year of initiation of IFX treatment** | | | | | 0.0205 |
| 2015 | 32 (20.6%) | 8 (10.3%) | 48 (18.5%) | 4 (10.5%) |  |
| 2016 | 37 (23.9%) | 10 (12.8%) | 74 (28.6%) | 10 (26.3%) |  |
| 2017 | 42 (27.1%) | 27 (34.6%) | 75 (29.0%) | 10 (26.3%) |  |
| 2018 | 44 (28.4%) | 33 (42.3%) | 62 (23.9%) | 14 (36.8%) |  |
| **Time to first TDM (days)** | | | | | 0.0029 |
| Mean (SD) | 455.3 (356.84) | 301.9 (299.55) | 453.0 (358.18) | 355.6 (322.53) |  |
| **Year of first instance of TDM** | | | | | 0.5498 |
| 2015 | 0 (0.0%) | 0 (0.0%) | 0 (0.0%) | 0 (0.0%) |  |
| 2016 | 0 (0.0%) | 0 (0.0%) | 0 (0.0%) | 0 (0.0%) |  |
| 2017 | 55 (35.5%) | 22 (28.2%) | 98 (37.8%) | 14 (36.8%) |  |
| 2018 | 67 (43.2%) | 40 (51.3%) | 111 (42.9%) | 13 (34.2%) |  |
| 2019 | 33 (21.3%) | 16 (20.5%) | 50 (19.3%) | 11 (28.9%) |  |

TDM=therapeutic drug monitoring. P-value: categorical variables, chi-square test; numeric variables, ANOVA.

^†^Atlantic includes New Brunswick, Nova Scotia, Prince Edward Island, Newfoundland, and Labrador.

Dose optimization thresholds: High: A treatment interval decrease of ≥11 days (1.57 weeks) with a posterior interval of ≤35 days (5 weeks), and/or a dose level increase of ≥1.5 mg/kg with a posterior dose level of ≥9 mg/kg. Low: A treatment interval decrease of ≥11 days (1.57 weeks) with a posterior interval of ≤46 days (6.57 weeks), and/or dose level increase of ≥1.5 mg/kg with a posterior dose level of ≥7 mg/kg.

A=First TDM serum IFX concentration below threshold and no dose optimization within post-index period;

B=First TDM serum IFX concentration below threshold and dose optimization within post-index period;

C=First TDM serum IFX concentration at or above threshold and no dose optimization within post-index period;

D=First TDM serum IFX concentration at or above threshold and dose optimization within post-index period.

Suppl Table 8k: Subgroup Demographics and Baseline Characteristics of Patients with UC with a DO (Based on Low Threshold Criteria) Prior to their First Instance of TDM (Serum IFX Threshold: 10 μg/mL; Post-Index Period for Dose Optimization: +9 weeks; Post-Index Dose Optimization Threshold: High)

|  | **A (N=270)** | **B (N=75)** | **C (N=180)** | **D (N=5)** | **p-value** |
| --- | --- | --- | --- | --- | --- |
| **Age (years)** | | | | | 0.0392 |
| Mean (SD) | 43.4 (16.16) | 41.6 (16.95) | 39.9 (15.35) | 55.8 (15.04) |  |
| Missing | 0 | 0 | 0 | 1 |  |
| **Age group (years)** | | | | | 0.3722 |
| 18 to 64 | 235 (87.0%) | 67 (89.3%) | 165 (91.7%) | 3 (75.0%) |  |
| 65 to 90 | 35 (13.0%) | 8 (10.7%) | 15 (8.3%) | 1 (25.0%) |  |
| Missing | 0 | 0 | 0 | 1 |  |
| **Gender** | | | | | 0.4794 |
| Female | 120 (44.4%) | 37 (49.3%) | 93 (51.7%) | 2 (40.0%) |  |
| Male | 150 (55.6%) | 38 (50.7%) | 87 (48.3%) | 3 (60.0%) |  |
| **First recorded weight** | | | | | 0.3193 |
| Mean (SD) | 75.05 (18.266) | 71.22 (16.666) | 73.71 (17.401) | 80.71 (15.991) |  |
| Missing | 2 | 0 | 0 | 0 |  |
| **Province/region of treating physician** | | | | | 0.1876 |
| Alberta | 15 (5.6%) | 2 (2.7%) | 8 (4.4%) | 1 (20.0%) |  |
| Atlantic^†^ | 24 (8.9%) | 10 (13.3%) | 16 (8.9%) | 0 (0.0%) |  |
| British Columbia | 40 (14.8%) | 5 (6.7%) | 26 (14.4%) | 2 (40.0%) |  |
| Ontario | 129 (47.8%) | 47 (62.7%) | 102 (56.7%) | 1 (20.0%) |  |
| Quebec | 61 (22.6%) | 11 (14.7%) | 28 (15.6%) | 1 (20.0%) |  |
| Saskatchewan/  Manitoba | 1 (0.4%) | 0 (0.0%) | 0 (0.0%) | 0 (0.0%) |  |
| **Year of initiation of IFX treatment** | | | | | 0.0046 |
| 2015 | 60 (22.2%) | 4 (5.3%) | 28 (15.6%) | 0 (0.0%) |  |
| 2016 | 69 (25.6%) | 12 (16.0%) | 48 (26.7%) | 2 (40.0%) |  |
| 2017 | 70 (25.9%) | 26 (34.7%) | 57 (31.7%) | 1 (20.0%) |  |
| 2018 | 71 (26.3%) | 33 (44.0%) | 47 (26.1%) | 2 (40.0%) |  |
| **Time to first TDM (days)** | | | | | 0.0002 |
| Mean (SD) | 470.6 (367.41) | 274.1 (274.12) | 422.0 (339.62) | 276.6 (263.90) |  |
| **Year of first instance of TDM** | | | | | 0.8102 |
| 2015 | 0 (0.0%) | 0 (0.0%) | 0 (0.0%) | 0 (0.0%) |  |
| 2016 | 0 (0.0%) | 0 (0.0%) | 0 (0.0%) | 0 (0.0%) |  |
| 2017 | 101 (37.4%) | 22 (29.3%) | 64 (35.6%) | 2 (40.0%) |  |
| 2018 | 112 (41.5%) | 34 (45.3%) | 83 (46.1%) | 2 (40.0%) |  |
| 2019 | 57 (21.1%) | 19 (25.3%) | 33 (18.3%) | 1 (20.0%) |  |

TDM=therapeutic drug monitoring. P-value: categorical variables, chi-square test; numeric variables, ANOVA.

^†^Atlantic includes New Brunswick, Nova Scotia, Prince Edward Island, Newfoundland, and Labrador.

Dose optimization thresholds: High: A treatment interval decrease of ≥11 days (1.57 weeks) with a posterior interval of ≤35 days (5 weeks), and/or a dose level increase of ≥1.5 mg/kg with a posterior dose level of ≥9 mg/kg. Low: A treatment interval decrease of ≥11 days (1.57 weeks) with a posterior interval of ≤46 days (6.57 weeks), and/or dose level increase of ≥1.5 mg/kg with a posterior dose level of ≥7 mg/kg.

A=First TDM serum IFX concentration below threshold and no dose optimization within post-index period;

B=First TDM serum IFX concentration below threshold and dose optimization within post-index period;

C=First TDM serum IFX concentration at or above threshold and no dose optimization within post-index period;

D=First TDM serum IFX concentration at or above threshold and dose optimization within post-index period.

Suppl Table 8l: Subgroup Demographics and Baseline Characteristics of Patients with UC with a DO (Based on Low Threshold Criteria) Prior to their First Instance of TDM (Serum IFX Threshold: 10 μg/mL; Post-Index Period for Dose Optimization: +17 weeks; Post-Index Dose Optimization Threshold: High)

|  | **A (N=249)** | **B (N=96)** | **C (N=165)** | **D (N=20)** | **p-value** |
| --- | --- | --- | --- | --- | --- |
| **Age (years)** | | | | | 0.059 |
| Mean (SD) | 43.7 (16.33) | 41.3 (16.28) | 40.8 (15.59) | 35.1 (13.75) |  |
| Missing | 0 | 0 | 0 | 1 |  |
| **Age group (years)** | | | | | 0.4692 |
| 18 to 64 | 216 (86.7%) | 86 (89.6%) | 150 (90.9%) | 18 (94.7%) |  |
| 65 to 90 | 33 (13.3%) | 10 (10.4%) | 15 (9.1%) | 1 (5.3%) |  |
| Missing | 0 | 0 | 0 | 1 |  |
| **Gender** | | | | | 0.2659 |
| Female | 110 (44.2%) | 47 (49.0%) | 82 (49.7%) | 13 (65.0%) |  |
| Male | 139 (55.8%) | 49 (51.0%) | 83 (50.3%) | 7 (35.0%) |  |
| **First recorded weight** | | | | | 0.3004 |
| Mean (SD) | 75.33 (18.358) | 71.35 (16.702) | 74.07 (17.714) | 72.44 (14.422) |  |
| Missing | 2 | 0 | 0 | 0 |  |
| **Province/region of treating physician** | | | | | 0.0513 |
| Alberta | 15 (6.0%) | 2 (2.1%) | 5 (3.0%) | 4 (20.0%) |  |
| Atlantic^†^ | 22 (8.8%) | 12 (12.5%) | 15 (9.1%) | 1 (5.0%) |  |
| British Columbia | 37 (14.9%) | 8 (8.3%) | 24 (14.5%) | 4 (20.0%) |  |
| Ontario | 119 (47.8%) | 57 (59.4%) | 96 (58.2%) | 7 (35.0%) |  |
| Quebec | 55 (22.1%) | 17 (17.7%) | 25 (15.2%) | 4 (20.0%) |  |
| Saskatchewan/  Manitoba | 1 (0.4%) | 0 (0.0%) | 0 (0.0%) | 0 (0.0%) |  |
| **Year of initiation of IFX treatment** | | | | | 0.0078 |
| 2015 | 55 (22.1%) | 9 (9.4%) | 25 (15.2%) | 3 (15.0%) |  |
| 2016 | 67 (26.9%) | 14 (14.6%) | 44 (26.7%) | 6 (30.0%) |  |
| 2017 | 64 (25.7%) | 32 (33.3%) | 53 (32.1%) | 5 (25.0%) |  |
| 2018 | 63 (25.3%) | 41 (42.7%) | 43 (26.1%) | 6 (30.0%) |  |
| **Time to first TDM (days)** | | | | | 0.0008 |
| Mean (SD) | 475.2 (366.92) | 305.1 (303.39) | 421.6 (340.70) | 388.8 (322.13) |  |
| **Year of first instance of TDM** | | | | | 0.6910 |
| 2015 | 0 (0.0%) | 0 (0.0%) | 0 (0.0%) | 0 (0.0%) |  |
| 2016 | 0 (0.0%) | 0 (0.0%) | 0 (0.0%) | 0 (0.0%) |  |
| 2017 | 95 (38.2%) | 28 (29.2%) | 58 (35.2%) | 8 (40.0%) |  |
| 2018 | 102 (41.0%) | 44 (45.8%) | 76 (46.1%) | 9 (45.0%) |  |
| 2019 | 52 (20.9%) | 24 (25.0%) | 31 (18.8%) | 3 (15.0%) |  |

TDM=therapeutic drug monitoring. P-value: categorical variables, chi-square test; numeric variables, ANOVA.

^†^Atlantic includes New Brunswick, Nova Scotia, Prince Edward Island, Newfoundland, and Labrador.

Dose optimization thresholds: High: A treatment interval decrease of ≥11 days (1.57 weeks) with a posterior interval of ≤35 days (5 weeks), and/or a dose level increase of ≥1.5 mg/kg with a posterior dose level of ≥9 mg/kg. Low: A treatment interval decrease of ≥11 days (1.57 weeks) with a posterior interval of ≤46 days (6.57 weeks), and/or dose level increase of ≥1.5 mg/kg with a posterior dose level of ≥7 mg/kg.

A=First TDM serum IFX concentration below threshold and no dose optimization within post-index period;

B=First TDM serum IFX concentration below threshold and dose optimization within post-index period;

C=First TDM serum IFX concentration at or above threshold and no dose optimization within post-index period;

D=First TDM serum IFX concentration at or above threshold and dose optimization within post-index period.

Suppl Table 9a: Subgroup Demographics and Baseline Characteristics of Patients with UC with a DO (Based on High Threshold Criteria) Prior to their First Instance of TDM (Serum IFX Threshold: 3 μg/mL; Post-Index Period for Dose Optimization: +9 weeks; Post-Index Dose Optimization Threshold: Low)

|  | **A (N=68)** | **B (N=42)** | **C (N=239)** | **D (N=31)** | **p-value** |
| --- | --- | --- | --- | --- | --- |
| **Age (years)** | | | | | 0.0294 |
| Mean (SD) | 47.4 (17.53) | 43.6 (17.38) | 41.1 (16.03) | 39.2 (16.78) |  |
| **Age group (years)** | | | | | 0.0100 |
| 18 to 64 | 52 (76.5%) | 36 (85.7%) | 217 (90.8%) | 29 (93.5%) |  |
| 65 to 90 | 16 (23.5%) | 6 (14.3%) | 22 (9.2%) | 2 (6.5%) |  |
| **Gender** | | | | | 0.3508 |
| Female | 32 (47.1%) | 25 (59.5%) | 117 (49.0%) | 12 (38.7%) |  |
| Male | 36 (52.9%) | 17 (40.5%) | 122 (51.0%) | 19 (61.3%) |  |
| **First recorded weight** | | | | | 0.6750 |
| Mean (SD) | 73.64 (19.112) | 70.00 (16.176) | 73.50 (17.978) | 73.90 (13.815) |  |
| Missing | 0 | 0 | 1 | 0 |  |
| **Province/region of treating physician** | | | | | 0.1277 |
| Alberta | 3 (4.4%) | 0 (0.0%) | 14 (5.9%) | 3 (9.7%) |  |
| Atlantic^†^ | 8 (11.8%) | 8 (19.0%) | 18 (7.5%) | 1 (3.2%) |  |
| British Columbia | 10 (14.7%) | 3 (7.1%) | 38 (15.9%) | 1 (3.2%) |  |
| Ontario | 36 (52.9%) | 19 (45.2%) | 133 (55.6%) | 20 (64.5%) |  |
| Quebec | 11 (16.2%) | 12 (28.6%) | 35 (14.6%) | 6 (19.4%) |  |
| Saskatchewan/  Manitoba | 0 (0.0%) | 0 (0.0%) | 1 (0.4%) | 0 (0.0%) |  |
| **Year of initiation of IFX treatment** | | | | | 0.0385 |
| 2015 | 7 (10.3%) | 1 (2.4%) | 37 (15.5%) | 1 (3.2%) |  |
| 2016 | 13 (19.1%) | 6 (14.3%) | 65 (27.2%) | 6 (19.4%) |  |
| 2017 | 22 (32.4%) | 15 (35.7%) | 69 (28.9%) | 11 (35.5%) |  |
| 2018 | 26 (38.2%) | 20 (47.6%) | 68 (28.5%) | 13 (41.9%) |  |
| **Time to first TDM (days)** | | | | | 0.0009 |
| Mean (SD) | 318.2 (280.49) | 230.3 (208.90) | 414.1 (337.97) | 288.3 (319.48) |  |
| **Year of first instance of TDM** | | | | | 0.7808 |
| 2015 | 0 (0.0%) | 0 (0.0%) | 0 (0.0%) | 0 (0.0%) |  |
| 2016 | 0 (0.0%) | 0 (0.0%) | 0 (0.0%) | 0 (0.0%) |  |
| 2017 | 25 (36.8%) | 12 (28.6%) | 86 (36.0%) | 9 (29.0%) |  |
| 2018 | 31 (45.6%) | 19 (45.2%) | 107 (44.8%) | 13 (41.9%) |  |
| 2019 | 12 (17.6%) | 11 (26.2%) | 46 (19.2%) | 9 (29.0%) |  |

TDM=therapeutic drug monitoring. P-value: categorical variables, chi-square test; numeric variables, ANOVA.

^†^Atlantic includes New Brunswick, Nova Scotia, Prince Edward Island, Newfoundland, and Labrador.

Dose optimization thresholds: High: A treatment interval decrease of ≥11 days (1.57 weeks) with a posterior interval of ≤35 days (5 weeks), and/or a dose level increase of ≥1.5 mg/kg with a posterior dose level of ≥9 mg/kg. Low: A treatment interval decrease of ≥11 days (1.57 weeks) with a posterior interval of ≤46 days (6.57 weeks), and/or dose level increase of ≥1.5 mg/kg with a posterior dose level of ≥7 mg/kg.

A=First TDM serum IFX concentration below threshold and no dose optimization within post-index period;

B=First TDM serum IFX concentration below threshold and dose optimization within post-index period;

C=First TDM serum IFX concentration at or above threshold and no dose optimization within post-index period;

D=First TDM serum IFX concentration at or above threshold and dose optimization within post-index period.

Suppl Table 9b: Subgroup Demographics and Baseline Characteristics of Patients with UC with a DO (Based on High Threshold Criteria) Prior to their First Instance of TDM (Serum IFX Threshold: 3 μg/mL; Post-Index Period for Dose Optimization: +17 weeks; Post-Index Dose Optimization Threshold: Low)

|  | **A (N=59)** | **B (N=51)** | **C (N=212)** | **D (N=58)** | **p-value** |
| --- | --- | --- | --- | --- | --- |
| **Age (years)** | | | | | 0.0086 |
| Mean (SD) | 48.5 (17.55) | 43.0 (17.13) | 41.5 (15.73) | 38.7 (17.34) |  |
| **Age group (years)** | | | | | 0.0052 |
| 18 to 64 | 44 (74.6%) | 44 (86.3%) | 194 (91.5%) | 52 (89.7%) |  |
| 65 to 90 | 15 (25.4%) | 7 (13.7%) | 18 (8.5%) | 6 (10.3%) |  |
| **Gender** | | | | | 0.4964 |
| Female | 27 (45.8%) | 30 (58.8%) | 101 (47.6%) | 28 (48.3%) |  |
| Male | 32 (54.2%) | 21 (41.2%) | 111 (52.4%) | 30 (51.7%) |  |
| **First recorded weight** | | | | | 0.4453 |
| Mean (SD) | 74.41 (19.161) | 69.75 (16.528) | 73.88 (18.084) | 72.29 (15.357) |  |
| Missing | 0 | 0 | 0 | 1 |  |
| **Province/region of treating physician** | | | | | 0.0545 |
| Alberta | 3 (5.1%) | 0 (0.0%) | 10 (4.7%) | 7 (12.1%) |  |
| Atlantic^†^ | 7 (11.9%) | 9 (17.6%) | 16 (7.5%) | 3 (5.2%) |  |
| British Columbia | 9 (15.3%) | 4 (7.8%) | 35 (16.5%) | 4 (6.9%) |  |
| Ontario | 31 (52.5%) | 24 (47.1%) | 120 (56.6%) | 33 (56.9%) |  |
| Quebec | 9 (15.3%) | 14 (27.5%) | 30 (14.2%) | 11 (19.0%) |  |
| Saskatchewan/  Manitoba | 0 (0.0%) | 0 (0.0%) | 1 (0.5%) | 0 (0.0%) |  |
| **Year of initiation of IFX treatment** | | | | | 0.0846 |
| 2015 | 6 (10.2%) | 2 (3.9%) | 32 (15.1%) | 6 (10.3%) |  |
| 2016 | 12 (20.3%) | 7 (13.7%) | 56 (26.4%) | 15 (25.9%) |  |
| 2017 | 19 (32.2%) | 18 (35.3%) | 66 (31.1%) | 14 (24.1%) |  |
| 2018 | 22 (37.3%) | 24 (47.1%) | 58 (27.4%) | 23 (39.7%) |  |
| **Time to first TDM (days)** | | | | | 0.0027 |
| Mean (SD) | 330.0 (281.03) | 232.2 (219.93) | 411.7 (337.87) | 355.7 (336.41) |  |
| **Year of first instance of TDM** | | | | | 0.8164 |
| 2015 | 0 (0.0%) | 0 (0.0%) | 0 (0.0%) | 0 (0.0%) |  |
| 2016 | 0 (0.0%) | 0 (0.0%) | 0 (0.0%) | 0 (0.0%) |  |
| 2017 | 21 (35.6%) | 16 (31.4%) | 77 (36.3%) | 18 (31.0%) |  |
| 2018 | 27 (45.8%) | 23 (45.1%) | 96 (45.3%) | 24 (41.4%) |  |
| 2019 | 11 (18.6%) | 12 (23.5%) | 39 (18.4%) | 16 (27.6%) |  |

TDM=therapeutic drug monitoring. P-value: categorical variables, chi-square test; numeric variables, ANOVA.

^†^Atlantic includes New Brunswick, Nova Scotia, Prince Edward Island, Newfoundland, and Labrador.

Dose optimization thresholds: High: A treatment interval decrease of ≥11 days (1.57 weeks) with a posterior interval of ≤35 days (5 weeks), and/or a dose level increase of ≥1.5 mg/kg with a posterior dose level of ≥9 mg/kg. Low: A treatment interval decrease of ≥11 days (1.57 weeks) with a posterior interval of ≤46 days (6.57 weeks), and/or dose level increase of ≥1.5 mg/kg with a posterior dose level of ≥7 mg/kg.

A=First TDM serum IFX concentration below threshold and no dose optimization within post-index period;

B=First TDM serum IFX concentration below threshold and dose optimization within post-index period;

C=First TDM serum IFX concentration at or above threshold and no dose optimization within post-index period;

D=First TDM serum IFX concentration at or above threshold and dose optimization within post-index period.

Suppl Table 9c: Subgroup Demographics and Baseline Characteristics of Patients with UC with a DO (Based on High Threshold Criteria) Prior to their First Instance of TDM (Serum IFX Threshold: 5 μg/mL; Post-Index Period for Dose Optimization: +9 weeks; Post-Index Dose Optimization Threshold: Low)

|  | **A (N=96)** | **B (N=52)** | **C (N=211)** | **D (N=21)** | **p-value** |
| --- | --- | --- | --- | --- | --- |
| **Age (years)** | | | | | 0.0229 |
| Mean (SD) | 46.7 (17.50) | 41.1 (16.87) | 40.5 (15.77) | 43.2 (18.14) |  |
| **Age group (years)** | | | | | 0.0228 |
| 18 to 64 | 76 (79.2%) | 46 (88.5%) | 193 (91.5%) | 19 (90.5%) |  |
| 65 to 90 | 20 (20.8%) | 6 (11.5%) | 18 (8.5%) | 2 (9.5%) |  |
| **Gender** | | | | | 0.5496 |
| Female | 42 (43.8%) | 28 (53.8%) | 107 (50.7%) | 9 (42.9%) |  |
| Male | 54 (56.3%) | 24 (46.2%) | 104 (49.3%) | 12 (57.1%) |  |
| **First recorded weight** | | | | | 0.8084 |
| Mean (SD) | 73.98 (17.983) | 71.03 (15.286) | 73.33 (18.342) | 73.19 (15.393) |  |
| Missing | 1 | 0 | 0 | 0 |  |
| **Province/region of treating physician** | | | | | 0.3536 |
| Alberta | 5 (5.2%) | 1 (1.9%) | 12 (5.7%) | 2 (9.5%) |  |
| Atlantic^†^ | 10 (10.4%) | 8 (15.4%) | 16 (7.6%) | 1 (4.8%) |  |
| British Columbia | 18 (18.8%) | 3 (5.8%) | 30 (14.2%) | 1 (4.8%) |  |
| Ontario | 48 (50.0%) | 26 (50.0%) | 121 (57.3%) | 13 (61.9%) |  |
| Quebec | 15 (15.6%) | 14 (26.9%) | 31 (14.7%) | 4 (19.0%) |  |
| Saskatchewan/  Manitoba | 0 (0.0%) | 0 (0.0%) | 1 (0.5%) | 0 (0.0%) |  |
| **Year of initiation of IFX treatment** | | | | | 0.0537 |
| 2015 | 15 (15.6%) | 2 (3.8%) | 29 (13.7%) | 0 (0.0%) |  |
| 2016 | 20 (20.8%) | 8 (15.4%) | 58 (27.5%) | 4 (19.0%) |  |
| 2017 | 27 (28.1%) | 17 (32.7%) | 64 (30.3%) | 9 (42.9%) |  |
| 2018 | 34 (35.4%) | 25 (48.1%) | 60 (28.4%) | 8 (38.1%) |  |
| **Time to first TDM (days)** | | | | | 0.0115 |
| Mean (SD) | 388.4 (335.46) | 251.9 (262.77) | 394.9 (325.51) | 262.6 (263.44) |  |
| **Year of first instance of TDM** | | | | | 0.4598 |
| 2015 | 0 (0.0%) | 0 (0.0%) | 0 (0.0%) | 0 (0.0%) |  |
| 2016 | 0 (0.0%) | 0 (0.0%) | 0 (0.0%) | 0 (0.0%) |  |
| 2017 | 34 (35.4%) | 14 (26.9%) | 77 (36.5%) | 7 (33.3%) |  |
| 2018 | 47 (49.0%) | 25 (48.1%) | 91 (43.1%) | 7 (33.3%) |  |
| 2019 | 15 (15.6%) | 13 (25.0%) | 43 (20.4%) | 7 (33.3%) |  |

TDM=therapeutic drug monitoring. P-value: categorical variables, chi-square test; numeric variables, ANOVA.

^†^Atlantic includes New Brunswick, Nova Scotia, Prince Edward Island, Newfoundland, and Labrador.

Dose optimization thresholds: High: A treatment interval decrease of ≥11 days (1.57 weeks) with a posterior interval of ≤35 days (5 weeks), and/or a dose level increase of ≥1.5 mg/kg with a posterior dose level of ≥9 mg/kg. Low: A treatment interval decrease of ≥11 days (1.57 weeks) with a posterior interval of ≤46 days (6.57 weeks), and/or dose level increase of ≥1.5 mg/kg with a posterior dose level of ≥7 mg/kg.

A=First TDM serum IFX concentration below threshold and no dose optimization within post-index period;

B=First TDM serum IFX concentration below threshold and dose optimization within post-index period;

C=First TDM serum IFX concentration at or above threshold and no dose optimization within post-index period;

D=First TDM serum IFX concentration at or above threshold and dose optimization within post-index period.

Suppl Table 9d: Subgroup Demographics and Baseline Characteristics of Patients with UC with a DO (Based on High Threshold Criteria) Prior to their First Instance of TDM (Serum IFX Threshold: 5 μg/mL; Post-Index Period for Dose Optimization: +17 weeks; Post-Index Dose Optimization Threshold: Low)

|  | **A (N=82)** | **B (N=66)** | **C (N=189)** | **D (N=43)** | **p-value** |
| --- | --- | --- | --- | --- | --- |
| **Age (years)** | | | | | 0.0133 |
| Mean (SD) | 47.6 (17.30) | 41.3 (17.09) | 41.0 (15.58) | 39.7 (17.77) |  |
| **Age group (years)** | | | | | 0.0143 |
| 18 to 64 | 64 (78.0%) | 58 (87.9%) | 174 (92.1%) | 38 (88.4%) |  |
| 65 to 90 | 18 (22.0%) | 8 (12.1%) | 15 (7.9%) | 5 (11.6%) |  |
| **Gender** | | | | | 0.5544 |
| Female | 35 (42.7%) | 35 (53.0%) | 93 (49.2%) | 23 (53.5%) |  |
| Male | 47 (57.3%) | 31 (47.0%) | 96 (50.8%) | 20 (46.5%) |  |
| **First recorded weight** | | | | | 0.5390 |
| Mean (SD) | 74.47 (17.827) | 70.99 (16.020) | 73.79 (18.529) | 71.25 (15.897) |  |
| Missing | 0 | 1 | 0 | 0 |  |
| **Province/region of treating physician** | | | | | 0.2582 |
| Alberta | 4 (4.9%) | 2 (3.0%) | 9 (4.8%) | 5 (11.6%) |  |
| Atlantic^†^ | 9 (11.0%) | 9 (13.6%) | 14 (7.4%) | 3 (7.0%) |  |
| British Columbia | 15 (18.3%) | 6 (9.1%) | 29 (15.3%) | 2 (4.7%) |  |
| Ontario | 42 (51.2%) | 32 (48.5%) | 109 (57.7%) | 25 (58.1%) |  |
| Quebec | 12 (14.6%) | 17 (25.8%) | 27 (14.3%) | 8 (18.6%) |  |
| Saskatchewan/  Manitoba | 0 (0.0%) | 0 (0.0%) | 1 (0.5%) | 0 (0.0%) |  |
| **Year of initiation of IFX treatment** | | | | | 0.1788 |
| 2015 | 13 (15.9%) | 4 (6.1%) | 25 (13.2%) | 4 (9.3%) |  |
| 2016 | 17 (20.7%) | 11 (16.7%) | 51 (27.0%) | 11 (25.6%) |  |
| 2017 | 23 (28.0%) | 21 (31.8%) | 62 (32.8%) | 11 (25.6%) |  |
| 2018 | 29 (35.4%) | 30 (45.5%) | 51 (27.0%) | 17 (39.5%) |  |
| **Time to first TDM (days)** | | | | | 0.0389 |
| Mean (SD) | 397.8 (335.56) | 269.1 (280.51) | 392.3 (324.97) | 342.0 (309.79) |  |
| **Year of first instance of TDM** | | | | | 0.5418 |
| 2015 | 0 (0.0%) | 0 (0.0%) | 0 (0.0%) | 0 (0.0%) |  |
| 2016 | 0 (0.0%) | 0 (0.0%) | 0 (0.0%) | 0 (0.0%) |  |
| 2017 | 28 (34.1%) | 20 (30.3%) | 70 (37.0%) | 14 (32.6%) |  |
| 2018 | 41 (50.0%) | 31 (47.0%) | 82 (43.4%) | 16 (37.2%) |  |
| 2019 | 13 (15.9%) | 15 (22.7%) | 37 (19.6%) | 13 (30.2%) |  |

TDM=therapeutic drug monitoring. P-value: categorical variables, chi-square test; numeric variables, ANOVA.

^†^Atlantic includes New Brunswick, Nova Scotia, Prince Edward Island, Newfoundland, and Labrador.

Dose optimization thresholds: High: A treatment interval decrease of ≥11 days (1.57 weeks) with a posterior interval of ≤35 days (5 weeks), and/or a dose level increase of ≥1.5 mg/kg with a posterior dose level of ≥9 mg/kg. Low: A treatment interval decrease of ≥11 days (1.57 weeks) with a posterior interval of ≤46 days (6.57 weeks), and/or dose level increase of ≥1.5 mg/kg with a posterior dose level of ≥7 mg/kg.

A=First TDM serum IFX concentration below threshold and no dose optimization within post-index period;

B=First TDM serum IFX concentration below threshold and dose optimization within post-index period;

C=First TDM serum IFX concentration at or above threshold and no dose optimization within post-index period;

D=First TDM serum IFX concentration at or above threshold and dose optimization within post-index period.

Suppl Table 9e: Subgroup Demographics and Baseline Characteristics of Patients with UC with a DO (Based on High Threshold Criteria) Prior to their First Instance of TDM (Serum IFX Threshold: 10 μg/mL; Post-Index Period for Dose Optimization: +9 weeks; Post-Index Dose Optimization Threshold: Low)

|  | **A (N=157)** | **B (N=68)** | **C (N=150)** | **D (N=5)** | **p-value** |
| --- | --- | --- | --- | --- | --- |
| **Age (years)** | | | | | 0.0330 |
| Mean (SD) | 44.8 (16.92) | 41.0 (17.10) | 40.0 (15.85) | 52.2 (15.71) |  |
| **Age group (years)** | | | | | 0.2288 |
| 18 to 64 | 132 (84.1%) | 61 (89.7%) | 137 (91.3%) | 4 (80.0%) |  |
| 65 to 90 | 25 (15.9%) | 7 (10.3%) | 13 (8.7%) | 1 (20.0%) |  |
| **Gender** | | | | | 0.4461 |
| Female | 73 (46.5%) | 36 (52.9%) | 76 (50.7%) | 1 (20.0%) |  |
| Male | 84 (53.5%) | 32 (47.1%) | 74 (49.3%) | 4 (80.0%) |  |
| **First recorded weight** | | | | | 0.4191 |
| Mean (SD) | 73.54 (18.623) | 70.83 (15.147) | 73.51 (17.820) | 82.89 (13.003) |  |
| Missing | 1 | 0 | 0 | 0 |  |
| **Province/region of treating physician** | | | | | 0.2756 |
| Alberta | 10 (6.4%) | 2 (2.9%) | 7 (4.7%) | 1 (20.0%) |  |
| Atlantic^†^ | 13 (8.3%) | 9 (13.2%) | 13 (8.7%) | 0 (0.0%) |  |
| British Columbia | 26 (16.6%) | 3 (4.4%) | 22 (14.7%) | 1 (20.0%) |  |
| Ontario | 82 (52.2%) | 38 (55.9%) | 87 (58.0%) | 1 (20.0%) |  |
| Quebec | 25 (15.9%) | 16 (23.5%) | 21 (14.0%) | 2 (40.0%) |  |
| Saskatchewan/  Manitoba | 1 (0.6%) | 0 (0.0%) | 0 (0.0%) | 0 (0.0%) |  |
| **Year of initiation of IFX treatment** | | | | | 0.0797 |
| 2015 | 25 (15.9%) | 2 (2.9%) | 19 (12.7%) | 0 (0.0%) |  |
| 2016 | 40 (25.5%) | 11 (16.2%) | 38 (25.3%) | 1 (20.0%) |  |
| 2017 | 41 (26.1%) | 24 (35.3%) | 50 (33.3%) | 2 (40.0%) |  |
| 2018 | 51 (32.5%) | 31 (45.6%) | 43 (28.7%) | 2 (40.0%) |  |
| **Time to first TDM (days)** | | | | | 0.0102 |
| Mean (SD) | 402.0 (331.06) | 256.9 (268.38) | 383.3 (325.83) | 228.4 (142.85) |  |
| **Year of first instance of TDM** | | | | | 0.7084 |
| 2015 | 0 (0.0%) | 0 (0.0%) | 0 (0.0%) | 0 (0.0%) |  |
| 2016 | 0 (0.0%) | 0 (0.0%) | 0 (0.0%) | 0 (0.0%) |  |
| 2017 | 59 (37.6%) | 19 (27.9%) | 52 (34.7%) | 2 (40.0%) |  |
| 2018 | 68 (43.3%) | 30 (44.1%) | 70 (46.7%) | 2 (40.0%) |  |
| 2019 | 30 (19.1%) | 19 (27.9%) | 28 (18.7%) | 1 (20.0%) |  |

TDM=therapeutic drug monitoring. P-value: categorical variables, chi-square test; numeric variables, ANOVA.

^†^Atlantic includes New Brunswick, Nova Scotia, Prince Edward Island, Newfoundland, and Labrador.

Dose optimization thresholds: High: A treatment interval decrease of ≥11 days (1.57 weeks) with a posterior interval of ≤35 days (5 weeks), and/or a dose level increase of ≥1.5 mg/kg with a posterior dose level of ≥9 mg/kg. Low: A treatment interval decrease of ≥11 days (1.57 weeks) with a posterior interval of ≤46 days (6.57 weeks), and/or dose level increase of ≥1.5 mg/kg with a posterior dose level of ≥7 mg/kg.

A=First TDM serum IFX concentration below threshold and no dose optimization within post-index period;

B=First TDM serum IFX concentration below threshold and dose optimization within post-index period;

C=First TDM serum IFX concentration at or above threshold and no dose optimization within post-index period;

D=First TDM serum IFX concentration at or above threshold and dose optimization within post-index period.

Suppl Table 9f: Subgroup Demographics and Baseline Characteristics of Patients with UC with a DO (Based on High Threshold Criteria) Prior to their First Instance of TDM (Serum IFX Threshold: 10 μg/mL; Post-Index Period for Dose Optimization: +17 weeks; Post-Index Dose Optimization Threshold: Low)

|  | **A (N=134)** | **B (N=91)** | **C (N=137)** | **D (N=18)** | **p-value** |
| --- | --- | --- | --- | --- | --- |
| **Age (years)** | | | | | 0.0474 |
| Mean (SD) | 45.6 (16.74) | 40.9 (17.15) | 40.5 (15.65) | 39.7 (18.51) |  |
| **Age group (years)** | | | | | 0.1762 |
| 18 to 64 | 112 (83.6%) | 81 (89.0%) | 126 (92.0%) | 15 (83.3%) |  |
| 65 to 90 | 22 (16.4%) | 10 (11.0%) | 11 (8.0%) | 3 (16.7%) |  |
| **Gender** | | | | | 0.6893 |
| Female | 61 (45.5%) | 48 (52.7%) | 67 (48.9%) | 10 (55.6%) |  |
| Male | 73 (54.5%) | 43 (47.3%) | 70 (51.1%) | 8 (44.4%) |  |
| **First recorded weight** | | | | | 0.5079 |
| Mean (SD) | 74.06 (18.560) | 70.72 (16.097) | 73.93 (18.089) | 72.99 (15.148) |  |
| Missing | 0 | 1 | 0 | 0 |  |
| **Province/region of treating physician** | | | | | 0.0456 |
| Alberta | 9 (6.7%) | 3 (3.3%) | 4 (2.9%) | 4 (22.2%) |  |
| Atlantic^†^ | 11 (8.2%) | 11 (12.1%) | 12 (8.8%) | 1 (5.6%) |  |
| British Columbia | 23 (17.2%) | 6 (6.6%) | 21 (15.3%) | 2 (11.1%) |  |
| Ontario | 70 (52.2%) | 50 (54.9%) | 81 (59.1%) | 7 (38.9%) |  |
| Quebec | 20 (14.9%) | 21 (23.1%) | 19 (13.9%) | 4 (22.2%) |  |
| Saskatchewan/  Manitoba | 1 (0.7%) | 0 (0.0%) | 0 (0.0%) | 0 (0.0%) |  |
| **Year of initiation of IFX treatment** | | | | | 0.0744 |
| 2015 | 22 (16.4%) | 5 (5.5%) | 16 (11.7%) | 3 (16.7%) |  |
| 2016 | 35 (26.1%) | 16 (17.6%) | 33 (24.1%) | 6 (33.3%) |  |
| 2017 | 37 (27.6%) | 28 (30.8%) | 48 (35.0%) | 4 (22.2%) |  |
| 2018 | 40 (29.9%) | 42 (46.2%) | 40 (29.2%) | 5 (27.8%) |  |
| **Time to first TDM (days)** | | | | | 0.0075 |
| Mean (SD) | 416.6 (332.12) | 272.1 (281.27) | 371.8 (322.78) | 428.4 (325.03) |  |
| **Year of first instance of TDM** | | | | | 0.4272 |
| 2015 | 0 (0.0%) | 0 (0.0%) | 0 (0.0%) | 0 (0.0%) |  |
| 2016 | 0 (0.0%) | 0 (0.0%) | 0 (0.0%) | 0 (0.0%) |  |
| 2017 | 51 (38.1%) | 27 (29.7%) | 47 (34.3%) | 7 (38.9%) |  |
| 2018 | 60 (44.8%) | 38 (41.8%) | 63 (46.0%) | 9 (50.0%) |  |
| 2019 | 23 (17.2%) | 26 (28.6%) | 27 (19.7%) | 2 (11.1%) |  |

TDM=therapeutic drug monitoring. P-value: categorical variables, chi-square test; numeric variables, ANOVA.

^†^Atlantic includes New Brunswick, Nova Scotia, Prince Edward Island, Newfoundland, and Labrador.

Dose optimization thresholds: High: A treatment interval decrease of ≥11 days (1.57 weeks) with a posterior interval of ≤35 days (5 weeks), and/or a dose level increase of ≥1.5 mg/kg with a posterior dose level of ≥9 mg/kg. Low: A treatment interval decrease of ≥11 days (1.57 weeks) with a posterior interval of ≤46 days (6.57 weeks), and/or dose level increase of ≥1.5 mg/kg with a posterior dose level of ≥7 mg/kg.

A=First TDM serum IFX concentration below threshold and no dose optimization within post-index period;

B=First TDM serum IFX concentration below threshold and dose optimization within post-index period;

C=First TDM serum IFX concentration at or above threshold and no dose optimization within post-index period;

D=First TDM serum IFX concentration at or above threshold and dose optimization within post-index period.

Suppl Table 9g: Subgroup Demographics and Baseline Characteristics of Patients with UC with a DO (Based on High Threshold Criteria) Prior to their First Instance of TDM (Serum IFX Threshold: 3 μg/mL; Post-Index Period for Dose Optimization: +9 weeks; Post-Index Dose Optimization Threshold: High)

|  | **A (N=78)** | **B (N=32)** | **C (N=247)** | **D (N=23)** | **p-value** |
| --- | --- | --- | --- | --- | --- |
| **Age (years)** | | | | | 0.0399 |
| Mean (SD) | 47.0 (17.69) | 43.4 (17.02) | 40.9 (15.94) | 40.7 (18.06) |  |
| **Age group (years)** | | | | | 0.0095 |
| 18 to 64 | 60 (76.9%) | 28 (87.5%) | 225 (91.1%) | 21 (91.3%) |  |
| 65 to 90 | 18 (23.1%) | 4 (12.5%) | 22 (8.9%) | 2 (8.7%) |  |
| **Gender** | | | | | 0.3444 |
| Female | 37 (47.4%) | 20 (62.5%) | 120 (48.6%) | 9 (39.1%) |  |
| Male | 41 (52.6%) | 12 (37.5%) | 127 (51.4%) | 14 (60.9%) |  |
| **First recorded weight** | | | | | 0.7672 |
| Mean (SD) | 73.17 (18.769) | 70.01 (16.260) | 73.55 (17.770) | 73.47 (15.020) |  |
| Missing | 0 | 0 | 1 | 0 |  |
| **Province/region of treating physician** | | | | | 0.3878 |
| Alberta | 3 (3.8%) | 0 (0.0%) | 15 (6.1%) | 2 (8.7%) |  |
| Atlantic^†^ | 10 (12.8%) | 6 (18.8%) | 18 (7.3%) | 1 (4.3%) |  |
| British Columbia | 11 (14.1%) | 2 (6.3%) | 38 (15.4%) | 1 (4.3%) |  |
| Ontario | 37 (47.4%) | 18 (56.3%) | 137 (55.5%) | 16 (69.6%) |  |
| Quebec | 17 (21.8%) | 6 (18.8%) | 38 (15.4%) | 3 (13.0%) |  |
| Saskatchewan/  Manitoba | 0 (0.0%) | 0 (0.0%) | 1 (0.4%) | 0 (0.0%) |  |
| **Year of initiation of IFX treatment** | | | | | 0.0179 |
| 2015 | 7 (9.0%) | 1 (3.1%) | 38 (15.4%) | 0 (0.0%) |  |
| 2016 | 15 (19.2%) | 4 (12.5%) | 67 (27.1%) | 4 (17.4%) |  |
| 2017 | 26 (33.3%) | 11 (34.4%) | 73 (29.6%) | 7 (30.4%) |  |
| 2018 | 30 (38.5%) | 16 (50.0%) | 69 (27.9%) | 12 (52.2%) |  |
| **Time to first TDM (days)** | | | | | 0.0003 |
| Mean (SD) | 313.6 (277.93) | 214.0 (187.32) | 414.8 (340.83) | 237.3 (256.06) |  |
| **Year of first instance of TDM** | | | | | 0.6755 |
| 2015 | 0 (0.0%) | 0 (0.0%) | 0 (0.0%) | 0 (0.0%) |  |
| 2016 | 0 (0.0%) | 0 (0.0%) | 0 (0.0%) | 0 (0.0%) |  |
| 2017 | 28 (35.9%) | 9 (28.1%) | 89 (36.0%) | 6 (26.1%) |  |
| 2018 | 34 (43.6%) | 16 (50.0%) | 111 (44.9%) | 9 (39.1%) |  |
| 2019 | 16 (20.5%) | 7 (21.9%) | 47 (19.0%) | 8 (34.8%) |  |

TDM=therapeutic drug monitoring. P-value: categorical variables, chi-square test; numeric variables, ANOVA.

^†^Atlantic includes New Brunswick, Nova Scotia, Prince Edward Island, Newfoundland, and Labrador.

Dose optimization thresholds: High: A treatment interval decrease of ≥11 days (1.57 weeks) with a posterior interval of ≤35 days (5 weeks), and/or a dose level increase of ≥1.5 mg/kg with a posterior dose level of ≥9 mg/kg. Low: A treatment interval decrease of ≥11 days (1.57 weeks) with a posterior interval of ≤46 days (6.57 weeks), and/or dose level increase of ≥1.5 mg/kg with a posterior dose level of ≥7 mg/kg.

A=First TDM serum IFX concentration below threshold and no dose optimization within post-index period;

B=First TDM serum IFX concentration below threshold and dose optimization within post-index period;

C=First TDM serum IFX concentration at or above threshold and no dose optimization within post-index period;

D=First TDM serum IFX concentration at or above threshold and dose optimization within post-index period.

Suppl Table 9h: Subgroup Demographics and Baseline Characteristics of Patients with UC with a DO (Based on High Threshold Criteria) Prior to their First Instance of TDM (Serum IFX Threshold: 3 μg/mL; Post-Index Period for Dose Optimization: +17 weeks; Post-Index Dose Optimization Threshold: High)

|  | **A (N=67)** | **B (N=43)** | **C (N=233)** | **D (N=37)** | **p-value** |
| --- | --- | --- | --- | --- | --- |
| **Age (years)** | | | | | 0.0031 |
| Mean (SD) | 48.5 (17.89) | 42.0 (16.29) | 41.5 (16.02) | 36.8 (16.20) |  |
| **Age group (years)** | | | | | 0.0026 |
| 18 to 64 | 50 (74.6%) | 38 (88.4%) | 211 (90.6%) | 35 (94.6%) |  |
| 65 to 90 | 17 (25.4%) | 5 (11.6%) | 22 (9.4%) | 2 (5.4%) |  |
| **Gender** | | | | | 0.4436 |
| Female | 31 (46.3%) | 26 (60.5%) | 112 (48.1%) | 17 (45.9%) |  |
| Male | 36 (53.7%) | 17 (39.5%) | 121 (51.9%) | 20 (54.1%) |  |
| **First recorded weight** | | | | | 0.4975 |
| Mean (SD) | 74.03 (18.821) | 69.47 (16.633) | 73.74 (17.945) | 72.28 (14.789) |  |
| Missing | 0 | 0 | 1 | 0 |  |
| **Province/region of treating physician** | | | | | 0.2514 |
| Alberta | 3 (4.5%) | 0 (0.0%) | 12 (5.2%) | 5 (13.5%) |  |
| Atlantic^†^ | 8 (11.9%) | 8 (18.6%) | 17 (7.3%) | 2 (5.4%) |  |
| British Columbia | 9 (13.4%) | 4 (9.3%) | 36 (15.5%) | 3 (8.1%) |  |
| Ontario | 33 (49.3%) | 22 (51.2%) | 131 (56.2%) | 22 (59.5%) |  |
| Quebec | 14 (20.9%) | 9 (20.9%) | 36 (15.5%) | 5 (13.5%) |  |
| Saskatchewan/  Manitoba | 0 (0.0%) | 0 (0.0%) | 1 (0.4%) | 0 (0.0%) |  |
| **Year of initiation of IFX treatment** | | | | | 0.0580 |
| 2015 | 6 (9.0%) | 2 (4.7%) | 35 (15.0%) | 3 (8.1%) |  |
| 2016 | 13 (19.4%) | 6 (14.0%) | 63 (27.0%) | 8 (21.6%) |  |
| 2017 | 23 (34.3%) | 14 (32.6%) | 71 (30.5%) | 9 (24.3%) |  |
| 2018 | 25 (37.3%) | 21 (48.8%) | 64 (27.5%) | 17 (45.9%) |  |
| **Time to first TDM (days)** | | | | | 0.0025 |
| Mean (SD) | 314.2 (270.27) | 238.6 (233.51) | 412.4 (340.21) | 319.9 (314.25) |  |
| **Year of first instance of TDM** | | | | | 0.6862 |
| 2015 | 0 (0.0%) | 0 (0.0%) | 0 (0.0%) | 0 (0.0%) |  |
| 2016 | 0 (0.0%) | 0 (0.0%) | 0 (0.0%) | 0 (0.0%) |  |
| 2017 | 25 (37.3%) | 12 (27.9%) | 85 (36.5%) | 10 (27.0%) |  |
| 2018 | 28 (41.8%) | 22 (51.2%) | 104 (44.6%) | 16 (43.2%) |  |
| 2019 | 14 (20.9%) | 9 (20.9%) | 44 (18.9%) | 11 (29.7%) |  |

TDM=therapeutic drug monitoring. P-value: categorical variables, chi-square test; numeric variables, ANOVA.

^†^Atlantic includes New Brunswick, Nova Scotia, Prince Edward Island, Newfoundland, and Labrador.

Dose optimization thresholds: High: A treatment interval decrease of ≥11 days (1.57 weeks) with a posterior interval of ≤35 days (5 weeks), and/or a dose level increase of ≥1.5 mg/kg with a posterior dose level of ≥9 mg/kg. Low: A treatment interval decrease of ≥11 days (1.57 weeks) with a posterior interval of ≤46 days (6.57 weeks), and/or dose level increase of ≥1.5 mg/kg with a posterior dose level of ≥7 mg/kg.

A=First TDM serum IFX concentration below threshold and no dose optimization within post-index period;

B=First TDM serum IFX concentration below threshold and dose optimization within post-index period;

C=First TDM serum IFX concentration at or above threshold and no dose optimization within post-index period;

D=First TDM serum IFX concentration at or above threshold and dose optimization within post-index period.

Suppl Table 9i: Subgroup Demographics and Baseline Characteristics of Patients with UC with a DO (Based on High Threshold Criteria) Prior to their First Instance of TDM (Serum IFX Threshold: 5 μg/mL; Post-Index Period for Dose Optimization: +9 weeks; Post-Index Dose Optimization Threshold: High)

|  | **A (N=110)** | **B (N=38)** | **C (N=215)** | **D (N=17)** | **p-value** |
| --- | --- | --- | --- | --- | --- |
| **Age (years)** | | | | | 0.0687 |
| Mean (SD) | 45.8 (17.73) | 41.9 (16.43) | 40.6 (15.68) | 43.2 (19.75) |  |
| **Age group (years)** | | | | | 0.0250 |
| 18 to 64 | 88 (80.0%) | 34 (89.5%) | 197 (91.6%) | 15 (88.2%) |  |
| 65 to 90 | 22 (20.0%) | 4 (10.5%) | 18 (8.4%) | 2 (11.8%) |  |
| **Gender** | | | | | 0.3715 |
| Female | 48 (43.6%) | 22 (57.9%) | 109 (50.7%) | 7 (41.2%) |  |
| Male | 62 (56.4%) | 16 (42.1%) | 106 (49.3%) | 10 (58.8%) |  |
| **First recorded weight** | | | | | 0.8948 |
| Mean (SD) | 73.49 (17.637) | 71.34 (15.492) | 73.44 (18.202) | 71.70 (16.653) |  |
| Missing | 1 | 0 | 0 | 0 |  |
| **Province/region of treating physician** | | | | | 0.4346 |
| Alberta | 6 (5.5%) | 0 (0.0%) | 12 (5.6%) | 2 (11.8%) |  |
| Atlantic^†^ | 12 (10.9%) | 6 (15.8%) | 16 (7.4%) | 1 (5.9%) |  |
| British Columbia | 19 (17.3%) | 2 (5.3%) | 30 (14.0%) | 1 (5.9%) |  |
| Ontario | 50 (45.5%) | 24 (63.2%) | 124 (57.7%) | 10 (58.8%) |  |
| Quebec | 23 (20.9%) | 6 (15.8%) | 32 (14.9%) | 3 (17.6%) |  |
| Saskatchewan/  Manitoba | 0 (0.0%) | 0 (0.0%) | 1 (0.5%) | 0 (0.0%) |  |
| **Year of initiation of IFX treatment** | | | | | 0.0503 |
| 2015 | 16 (14.5%) | 1 (2.6%) | 29 (13.5%) | 0 (0.0%) |  |
| 2016 | 23 (20.9%) | 5 (13.2%) | 59 (27.4%) | 3 (17.6%) |  |
| 2017 | 32 (29.1%) | 12 (31.6%) | 67 (31.2%) | 6 (35.3%) |  |
| 2018 | 39 (35.5%) | 20 (52.6%) | 60 (27.9%) | 8 (47.1%) |  |
| **Time to first TDM (days)** | | | | | 0.0042 |
| Mean (SD) | 386.3 (342.40) | 207.8 (177.21) | 392.7 (323.16) | 259.5 (289.84) |  |
| **Year of first instance of TDM** | | | | | 0.3484 |
| 2015 | 0 (0.0%) | 0 (0.0%) | 0 (0.0%) | 0 (0.0%) |  |
| 2016 | 0 (0.0%) | 0 (0.0%) | 0 (0.0%) | 0 (0.0%) |  |
| 2017 | 38 (34.5%) | 10 (26.3%) | 79 (36.7%) | 5 (29.4%) |  |
| 2018 | 52 (47.3%) | 20 (52.6%) | 93 (43.3%) | 5 (29.4%) |  |
| 2019 | 20 (18.2%) | 8 (21.1%) | 43 (20.0%) | 7 (41.2%) |  |

TDM=therapeutic drug monitoring. P-value: categorical variables, chi-square test; numeric variables, ANOVA.

^†^Atlantic includes New Brunswick, Nova Scotia, Prince Edward Island, Newfoundland, and Labrador.

Dose optimization thresholds: High: A treatment interval decrease of ≥11 days (1.57 weeks) with a posterior interval of ≤35 days (5 weeks), and/or a dose level increase of ≥1.5 mg/kg with a posterior dose level of ≥9 mg/kg. Low: A treatment interval decrease of ≥11 days (1.57 weeks) with a posterior interval of ≤46 days (6.57 weeks), and/or dose level increase of ≥1.5 mg/kg with a posterior dose level of ≥7 mg/kg.

A=First TDM serum IFX concentration below threshold and no dose optimization within post-index period;

B=First TDM serum IFX concentration below threshold and dose optimization within post-index period;

C=First TDM serum IFX concentration at or above threshold and no dose optimization within post-index period;

D=First TDM serum IFX concentration at or above threshold and dose optimization within post-index period.

Suppl Table 9j: Subgroup Demographics and Baseline Characteristics of Patients with UC with a DO (Based on High Threshold Criteria) Prior to their First Instance of TDM (Serum IFX Threshold: 5 μg/mL; Post-Index Period for Dose Optimization: +17 weeks; Post-Index Dose Optimization Threshold: High)

|  | **A (N=98)** | **B (N=50)** | **C (N=202)** | **D (N=30)** | **p-value** |
| --- | --- | --- | --- | --- | --- |
| **Age (years)** | | | | | 0.0146 |
| Mean (SD) | 46.5 (17.99) | 41.4 (15.93) | 41.4 (15.79) | 36.6 (16.88) |  |
| **Age group (years)** | | | | | 0.0118 |
| 18 to 64 | 77 (78.6%) | 45 (90.0%) | 184 (91.1%) | 28 (93.3%) |  |
| 65 to 90 | 21 (21.4%) | 5 (10.0%) | 18 (8.9%) | 2 (6.7%) |  |
| **Gender** | | | | | 0.4658 |
| Female | 42 (42.9%) | 28 (56.0%) | 101 (50.0%) | 15 (50.0%) |  |
| Male | 56 (57.1%) | 22 (44.0%) | 101 (50.0%) | 15 (50.0%) |  |
| **First recorded weight** | | | | | 0.5959 |
| Mean (SD) | 74.05 (17.615) | 70.76 (15.948) | 73.69 (18.390) | 70.78 (15.745) |  |
| Missing | 1 | 0 | 0 | 0 |  |
| **Province/region of treating physician** | | | | | 0.1781 |
| Alberta | 6 (6.1%) | 0 (0.0%) | 9 (4.5%) | 5 (16.7%) |  |
| Atlantic^†^ | 10 (10.2%) | 8 (16.0%) | 15 (7.4%) | 2 (6.7%) |  |
| British Columbia | 16 (16.3%) | 5 (10.0%) | 29 (14.4%) | 2 (6.7%) |  |
| Ontario | 46 (46.9%) | 28 (56.0%) | 118 (58.4%) | 16 (53.3%) |  |
| Quebec | 20 (20.4%) | 9 (18.0%) | 30 (14.9%) | 5 (16.7%) |  |
| Saskatchewan/  Manitoba | 0 (0.0%) | 0 (0.0%) | 1 (0.5%) | 0 (0.0%) |  |
| **Year of initiation of IFX treatment** | | | | | 0.1262 |
| 2015 | 14 (14.3%) | 3 (6.0%) | 27 (13.4%) | 2 (6.7%) |  |
| 2016 | 21 (21.4%) | 7 (14.0%) | 55 (27.2%) | 7 (23.3%) |  |
| 2017 | 29 (29.6%) | 15 (30.0%) | 65 (32.2%) | 8 (26.7%) |  |
| 2018 | 34 (34.7%) | 25 (50.0%) | 55 (27.2%) | 13 (43.3%) |  |
| **Time to first TDM (days)** | | | | | 0.0264 |
| Mean (SD) | 388.4 (339.80) | 246.3 (246.26) | 391.4 (323.04) | 326.0 (315.46) |  |
| **Year of first instance of TDM** | | | | | 0.4365 |
| 2015 | 0 (0.0%) | 0 (0.0%) | 0 (0.0%) | 0 (0.0%) |  |
| 2016 | 0 (0.0%) | 0 (0.0%) | 0 (0.0%) | 0 (0.0%) |  |
| 2017 | 35 (35.7%) | 13 (26.0%) | 75 (37.1%) | 9 (30.0%) |  |
| 2018 | 45 (45.9%) | 27 (54.0%) | 87 (43.1%) | 11 (36.7%) |  |
| 2019 | 18 (18.4%) | 10 (20.0%) | 40 (19.8%) | 10 (33.3%) |  |

TDM=therapeutic drug monitoring. P-value: categorical variables, chi-square test; numeric variables, ANOVA.

^†^Atlantic includes New Brunswick, Nova Scotia, Prince Edward Island, Newfoundland, and Labrador.

Dose optimization thresholds: High: A treatment interval decrease of ≥11 days (1.57 weeks) with a posterior interval of ≤35 days (5 weeks), and/or a dose level increase of ≥1.5 mg/kg with a posterior dose level of ≥9 mg/kg. Low: A treatment interval decrease of ≥11 days (1.57 weeks) with a posterior interval of ≤46 days (6.57 weeks), and/or dose level increase of ≥1.5 mg/kg with a posterior dose level of ≥7 mg/kg.

A=First TDM serum IFX concentration below threshold and no dose optimization within post-index period;

B=First TDM serum IFX concentration below threshold and dose optimization within post-index period;

C=First TDM serum IFX concentration at or above threshold and no dose optimization within post-index period;

D=First TDM serum IFX concentration at or above threshold and dose optimization within post-index period.

Suppl Table 9k: Subgroup Demographics and Baseline Characteristics of Patients with UC with a DO (Based on High Threshold Criteria) Prior to their First Instance of TDM (Serum IFX Threshold: 10 μg/mL; Post-Index Period for Dose Optimization: +9 weeks; Post-Index Dose Optimization Threshold: High)

|  | **A (N=173)** | **B (N=52)** | **C (N=152)** | **D (N=3)** | **p-value** |
| --- | --- | --- | --- | --- | --- |
| **Age (years)** | | | | | 0.0138 |
| Mean (SD) | 44.4 (17.00) | 41.1 (17.05) | 40.0 (15.77) | 62.7 (7.23) |  |
| **Age group (years)** | | | | | 0.1466 |
| 18 to 64 | 146 (84.4%) | 47 (90.4%) | 139 (91.4%) | 2 (66.7%) |  |
| 65 to 90 | 27 (15.6%) | 5 (9.6%) | 13 (8.6%) | 1 (33.3%) |  |
| **Gender** | | | | | 0.7596 |
| Female | 81 (46.8%) | 28 (53.8%) | 76 (50.0%) | 1 (33.3%) |  |
| Male | 92 (53.2%) | 24 (46.2%) | 76 (50.0%) | 2 (66.7%) |  |
| **First recorded weight** | | | | | 0.4069 |
| Mean (SD) | 73.36 (18.280) | 70.59 (15.357) | 73.57 (17.708) | 86.48 (16.956) |  |
| Missing | 1 | 0 | 0 | 0 |  |
| **Province/region of treating physician** | | | | | 0.2068 |
| Alberta | 11 (6.4%) | 1 (1.9%) | 7 (4.6%) | 1 (33.3%) |  |
| Atlantic^†^ | 15 (8.7%) | 7 (13.5%) | 13 (8.6%) | 0 (0.0%) |  |
| British Columbia | 27 (15.6%) | 2 (3.8%) | 22 (14.5%) | 1 (33.3%) |  |
| Ontario | 86 (49.7%) | 34 (65.4%) | 88 (57.9%) | 0 (0.0%) |  |
| Quebec | 33 (19.1%) | 8 (15.4%) | 22 (14.5%) | 1 (33.3%) |  |
| Saskatchewan/  Manitoba | 1 (0.6%) | 0 (0.0%) | 0 (0.0%) | 0 (0.0%) |  |
| **Year of initiation of IFX treatment** | | | | | 0.0644 |
| 2015 | 26 (15.0%) | 1 (1.9%) | 19 (12.5%) | 0 (0.0%) |  |
| 2016 | 43 (24.9%) | 8 (15.4%) | 39 (25.7%) | 0 (0.0%) |  |
| 2017 | 48 (27.7%) | 17 (32.7%) | 51 (33.6%) | 1 (33.3%) |  |
| 2018 | 56 (32.4%) | 26 (50.0%) | 43 (28.3%) | 2 (66.7%) |  |
| **Time to first TDM (days)** | | | | | 0.0042 |
| Mean (SD) | 397.2 (334.78) | 228.3 (221.69) | 382.9 (323.86) | 144.3 (74.61) |  |
| **Year of first instance of TDM** | | | | | 0.7867 |
| 2015 | 0 (0.0%) | 0 (0.0%) | 0 (0.0%) | 0 (0.0%) |  |
| 2016 | 0 (0.0%) | 0 (0.0%) | 0 (0.0%) | 0 (0.0%) |  |
| 2017 | 64 (37.0%) | 14 (26.9%) | 53 (34.9%) | 1 (33.3%) |  |
| 2018 | 74 (42.8%) | 24 (46.2%) | 71 (46.7%) | 1 (33.3%) |  |
| 2019 | 35 (20.2%) | 14 (26.9%) | 28 (18.4%) | 1 (33.3%) |  |

TDM=therapeutic drug monitoring. P-value: categorical variables, chi-square test; numeric variables, ANOVA.

^†^Atlantic includes New Brunswick, Nova Scotia, Prince Edward Island, Newfoundland, and Labrador.

Dose optimization thresholds: High: A treatment interval decrease of ≥11 days (1.57 weeks) with a posterior interval of ≤35 days (5 weeks), and/or a dose level increase of ≥1.5 mg/kg with a posterior dose level of ≥9 mg/kg. Low: A treatment interval decrease of ≥11 days (1.57 weeks) with a posterior interval of ≤46 days (6.57 weeks), and/or dose level increase of ≥1.5 mg/kg with a posterior dose level of ≥7 mg/kg.

A=First TDM serum IFX concentration below threshold and no dose optimization within post-index period;

B=First TDM serum IFX concentration below threshold and dose optimization within post-index period;

C=First TDM serum IFX concentration at or above threshold and no dose optimization within post-index period;

D=First TDM serum IFX concentration at or above threshold and dose optimization within post-index period.

Suppl Table 9l: Subgroup Demographics and Baseline Characteristics of Patients with UC with a DO (Based on High Threshold Criteria) Prior to their First Instance of TDM (Serum IFX Threshold: 10 μg/mL; Post-Index Period for Dose Optimization: +17 weeks; Post-Index Dose Optimization Threshold: High)

|  | **A (N=159)** | **B (N=66)** | **C (N=141)** | **D (N=14)** | **p-value** |
| --- | --- | --- | --- | --- | --- |
| **Age (years)** | | | | | 0.0395 |
| Mean (SD) | 45.0 (17.14) | 40.5 (16.45) | 40.9 (15.92) | 35.4 (15.85) |  |
| **Age group (years)** | | | | | 0.1963 |
| 18 to 64 | 133 (83.6%) | 60 (90.9%) | 128 (90.8%) | 13 (92.9%) |  |
| 65 to 90 | 26 (16.4%) | 6 (9.1%) | 13 (9.2%) | 1 (7.1%) |  |
| **Gender** | | | | | 0.7566 |
| Female | 74 (46.5%) | 35 (53.0%) | 69 (48.9%) | 8 (57.1%) |  |
| Male | 85 (53.5%) | 31 (47.0%) | 72 (51.1%) | 6 (42.9%) |  |
| **First recorded weight** | | | | | 0.4396 |
| Mean (SD) | 73.88 (18.301) | 69.94 (15.772) | 73.73 (17.965) | 74.70 (15.741) |  |
| Missing | 1 | 0 | 0 | 0 |  |
| **Province/region of treating physician** | | | | | 0.0191 |
| Alberta | 11 (6.9%) | 1 (1.5%) | 4 (2.8%) | 4 (28.6%) |  |
| Atlantic^†^ | 13 (8.2%) | 9 (13.6%) | 12 (8.5%) | 1 (7.1%) |  |
| British Columbia | 24 (15.1%) | 5 (7.6%) | 21 (14.9%) | 2 (14.3%) |  |
| Ontario | 80 (50.3%) | 40 (60.6%) | 84 (59.6%) | 4 (28.6%) |  |
| Quebec | 30 (18.9%) | 11 (16.7%) | 20 (14.2%) | 3 (21.4%) |  |
| Saskatchewan/  Manitoba | 1 (0.6%) | 0 (0.0%) | 0 (0.0%) | 0 (0.0%) |  |
| **Year of initiation of IFX treatment** | | | | | 0.0861 |
| 2015 | 24 (15.1%) | 3 (4.5%) | 17 (12.1%) | 2 (14.3%) |  |
| 2016 | 41 (25.8%) | 10 (15.2%) | 35 (24.8%) | 4 (28.6%) |  |
| 2017 | 45 (28.3%) | 20 (30.3%) | 49 (34.8%) | 3 (21.4%) |  |
| 2018 | 49 (30.8%) | 33 (50.0%) | 40 (28.4%) | 5 (35.7%) |  |
| **Time to first TDM (days)** | | | | | 0.0135 |
| Mean (SD) | 402.2 (333.61) | 252.0 (256.80) | 377.2 (322.32) | 390.1 (336.27) |  |
| **Year of first instance of TDM** | | | | | 0.6042 |
| 2015 | 0 (0.0%) | 0 (0.0%) | 0 (0.0%) | 0 (0.0%) |  |
| 2016 | 0 (0.0%) | 0 (0.0%) | 0 (0.0%) | 0 (0.0%) |  |
| 2017 | 61 (38.4%) | 17 (25.8%) | 49 (34.8%) | 5 (35.7%) |  |
| 2018 | 67 (42.1%) | 31 (47.0%) | 65 (46.1%) | 7 (50.0%) |  |
| 2019 | 31 (19.5%) | 18 (27.3%) | 27 (19.1%) | 2 (14.3%) |  |

TDM=therapeutic drug monitoring. P-value: categorical variables, chi-square test; numeric variables, ANOVA.

^†^Atlantic includes New Brunswick, Nova Scotia, Prince Edward Island, Newfoundland, and Labrador.

Dose optimization thresholds: High: A treatment interval decrease of ≥11 days (1.57 weeks) with a posterior interval of ≤35 days (5 weeks), and/or a dose level increase of ≥1.5 mg/kg with a posterior dose level of ≥9 mg/kg. Low: A treatment interval decrease of ≥11 days (1.57 weeks) with a posterior interval of ≤46 days (6.57 weeks), and/or dose level increase of ≥1.5 mg/kg with a posterior dose level of ≥7 mg/kg.

A=First TDM serum IFX concentration below threshold and no dose optimization within post-index period;

B=First TDM serum IFX concentration below threshold and dose optimization within post-index period;

C=First TDM serum IFX concentration at or above threshold and no dose optimization within post-index period;

D=First TDM serum IFX concentration at or above threshold and dose optimization within post-index period.

Suppl Table 10a: Overall Study Population Analysis Time-Dependent Cox Proportional Hazards Models

| **Model** | **Main factor: Interaction term(s)** | | **Purpose** |
| --- | --- | --- | --- |
| 1 | Province/Region  * TDM vs No TDM  (time-dependent covariate) |  | Main model |
| 2 | Province/Region  * TDM vs No TDM  (time-dependent covariate) | TDM vs No TDM  (time-dependent covariate)  *  Log (time to TDM) | To test the proportional hazards assumption based on model 1. |
| 3 | None |  | To allow for the possibility that the interaction term in model 1 was insignificant |
| 4 |  | TDM vs No TDM  (time-dependent covariate)  *  Log (time to TDM) | To allow for the possibility that the interaction factor in model 1 was insignificant and the proportional hazards assumption was not met |
|  | **Stratification factor** | | |
| All | Year of treatment initiation | | |
|  | **Main factor** | | |
| All | TDM vs No TDM (time-dependent covariate) | | |
|  | **Covariates** | | |
| All | Age group (18 to 65 vs. >65 to 90)  Gender (male vs. female)  First recorded weight (quartiles)  Province/region of treating physician | | |

TDM=therapeutic drug monitoring.

Suppl Table 10b: Subgroup Analysis Time-Dependent Cox Proportional Hazards Models

| **Model** | **Main factor: Interaction term(s)** | | | **Purpose** |
| --- | --- | --- | --- | --- |
| 1 | Serum IFX concentration (above or below a threshold value)  * Dose optimization within post-TDM period of 9/17 weeks  (yes or no) |  | | Main model |
| 2 | None |  | | To allow for the possibility that the interaction term in model 1 was insignificant |
| 3 | Serum IFX concentration (above or below a threshold value)  *  Dose optimization within post-TDM period of 9/17 weeks  (yes or no) | Dose optimization within post-TDM period of 9/17 weeks  (yes or no)  *  Log (time to dose optimization within post-TDM period of 9/17 weeks) | | To test the proportional hazards assumption based on model 1. |
| 4 |  | Dose optimization within post-TDM period of 9/17 weeks  (yes or no)  *  Log (time to dose optimization within post-TDM period of 9/17 weeks) | | To allow for the possibility that the interaction factor in model 1 was insignificant and the proportional hazards assumption was not met |
|  | **Main factors** | | | |
| All | Serum IFX concentration  (above or below a threshold value) | | Dose optimization within post-TDM period of 9/17 weeks (yes or no)  (time-dependent covariate) | |
|  | **Covariates** | | | |
| All | Log (time from first IFX treatment to a first TDM with a recorded TDM result)  Age group (18 to 65 vs. >65 to 90)  Gender (male vs. female)  First recorded weight (quartiles)  Province/region of treating physician  Year of initiation of IFX treatment | | | |

IFX=infliximab; TDM=therapeutic drug monitoring.

For models 3 and 4, when the proportional hazards assumption was violated, comparisons were evaluated at two time points for determining a post-TDM dose optimization: at 28 days (4 weeks) and at 42 days (6 weeks). In relation to the 9-week post-TDM period (approximately one cycle of treatment based on a treatment interval of every 8 weeks), 4 weeks represents a shorter, commonly observed interval, and 6 weeks represents the mid-point between 4 weeks and 8 weeks.

Suppl Table 11a: Subgroup Analysis Time-Dependent Cox Proportional Hazards Model for Patients with CD with no DO (Based on Low Threshold Criteria) Prior to their First Instance of TDM (Serum IFX Threshold: 3 μg/mL; Post-Index Period for Dose Optimization: +9 weeks; Post-Index Dose Optimization Threshold: Low)

| **Interaction factor (model 1)** | | | **p-value** |
| --- | --- | --- | --- |
| Serum IFX concentration (below vs. above threshold) * Dose optimization within post-index period (y vs. n) | | | **0.0016** |
| **Class Level** | **Reference Level** | **HR (95% CI)** | **p-value** |
| ***Main factor contrasts*** | | | |
| B: First TDM serum IFX <3 μg/mL;  Post-Index low-threshold DO  within 9 wk | A: First TDM serum IFX <3 μg/mL;  No post-Index low-threshold DO  within 9 wk | 0.36 (0.26, 0.50) | **<0.0001** |
| C: First TDM serum IFX ≥3 μg/mL;  No post-Index low-threshold DO  within 9 wk | A: First TDM serum IFX <3 μg/mL;  No post-Index low-threshold DO  within 9 wk | 0.23 (0.18, 0.30) | **<0.0001** |
| C: First TDM serum IFX ≥3 μg/mL;  No post-Index low-threshold DO  within 9 wk | B: First TDM serum IFX <3 μg/mL;  Post-Index low-threshold DO  within 9 wk | 0.63 (0.45, 0.88) | **0.0070** |
| ***Covariates*** | | | |
| log(days to TDM), per unit | 0 | 0.57 (0.45, 0.73) | **<0.0001** |
| <65 | ≥65 | 0.80 (0.57, 1.11) | 0.1745 |
| Female | Male | 1.18 (0.94, 1.48) | 0.1655 |
| Q1 (<62 kg) | Q4 (≥86 kg) | 0.80 (0.58, 1.09) | 0.1552 |
| Q2 (62 to <73 kg) | Q4 (≥86 kg) | 0.77 (0.56, 1.07) | 0.1186 |
| Q3 (73 to <86 kg) | Q4 (≥86 kg) | 0.95 (0.70, 1.28) | 0.7233 |
| Alberta | Ontario | 1.61 (0.90, 2.86) | 0.1084 |
| Atlantic^†^ | Ontario | 1.09 (0.81, 1.47) | 0.5809 |
| British Columbia | Ontario | 1.25 (0.87, 1.79) | 0.2281 |
| Quebec | Ontario | 1.18 (0.88, 1.59) | 0.2735 |
| Saskatchewan/Manitoba | Ontario | 2.25 (0.55, 9.20) | 0.2588 |
| 2015 | 2018 | 2.23 (1.18, 4.22) | **0.0134** |
| 2016 | 2018 | 1.88 (1.22, 2.88) | **0.0042** |
| 2017 | 2018 | 1.12 (0.84, 1.50) | 0.4410 |

CI=confidence interval; IFX=infliximab; TDM=therapeutic drug monitoring; wk=weeks.

^†^Atlantic includes New Brunswick, Nova Scotia, Prince Edward Island, Newfoundland, and Labrador.

Dose optimization thresholds: High: A treatment interval decrease of ≥11 days (1.57 weeks) with a posterior interval of ≤35 days (5 weeks), and/or a dose level increase of ≥1.5 mg/kg with a posterior dose level of ≥9 mg/kg. Low: A treatment interval decrease of ≥11 days (1.57 weeks) with a posterior interval of ≤46 days (6.57 weeks), and/or dose level increase of ≥1.5 mg/kg with a posterior dose level of ≥7 mg/kg.

Hazard ratio and 95% CI are from time-dependent Cox proportional hazards model 1 (Suppl Table 10b) with main factors of serum infliximab concentration (below vs. above threshold), a time-dependent covariate modelling dose optimization state (yes vs. no) within the post-index period and their interaction, and covariates as shown in the table

A hazard ratio of <1 indicates an advantage for the class level over the reference level. HR (95% CI) is shown in bold when p<0.05.

Suppl Table 11b: Subgroup Analysis Time-Dependent Cox Proportional Hazards Model for Patients with CD with no DO (Based on Low Threshold Criteria) Prior to their First Instance of TDM (Serum IFX Threshold: 3 μg/mL; Post-Index Period for Dose Optimization: +17 weeks; Post-Index Dose Optimization Threshold: Low)

| **Interaction factor (model 1)** | | | **p-value** |
| --- | --- | --- | --- |
| Serum IFX concentration (below vs. above threshold) * Dose optimization within post-index period (y vs. n) | | | **<0.0001** |
| **Class Level** | **Reference Level** | **HR (95% CI)** | **p-value** |
| ***Main factor contrasts*** | | | |
| B: First TDM serum IFX <3 μg/mL;  Post-Index low-threshold DO  within 17 wk | A: First TDM serum IFX <3 μg/mL;  No post-Index low-threshold DO  within 17 wk | 0.39 (0.29, 0.53) | **<0.0001** |
| C: First TDM serum IFX ≥3 μg/mL;  No post-Index low-threshold DO  within 17 wk | A: First TDM serum IFX <3 μg/mL;  No post-Index low-threshold DO  within 17 wk | 0.21 (0.16, 0.28) | **<0.0001** |
| C: First TDM serum IFX ≥3 μg/mL;  No post-Index low-threshold DO  within 17 wk | B: First TDM serum IFX <3 μg/mL;  Post-Index low-threshold DO  within 17 wk | 0.53 (0.39, 0.73) | **<0.0001** |
| ***Covariates*** | | | |
| log(days to TDM), per unit | 0 | 0.57 (0.45, 0.73) | **<0.0001** |
| <65 | ≥65 | 0.80 (0.58, 1.12) | 0.1955 |
| Female | Male | 1.17 (0.93, 1.48) | 0.1721 |
| Q1 (<62 kg) | Q4 (≥86 kg) | 0.79 (0.58, 1.09) | 0.1480 |
| Q2 (62 to <73 kg) | Q4 (≥86 kg) | 0.77 (0.56, 1.06) | 0.1072 |
| Q3 (73 to <86 kg) | Q4 (≥86 kg) | 0.94 (0.69, 1.27) | 0.6717 |
| Alberta | Ontario | 1.60 (0.90, 2.85) | 0.1127 |
| Atlantic^†^ | Ontario | 1.08 (0.80, 1.46) | 0.6145 |
| British Columbia | Ontario | 1.26 (0.88, 1.80) | 0.2124 |
| Quebec | Ontario | 1.17 (0.87, 1.57) | 0.3115 |
| Saskatchewan/Manitoba | Ontario | 2.32 (0.57, 9.50) | 0.2413 |
| 2015 | 2018 | 2.22 (1.17, 4.18) | **0.0142** |
| 2016 | 2018 | 1.85 (1.20, 2.85) | **0.0050** |
| 2017 | 2018 | 1.12 (0.84, 1.50) | 0.4424 |

CI=confidence interval; IFX=infliximab; TDM=therapeutic drug monitoring; wk=weeks.

^†^Atlantic includes New Brunswick, Nova Scotia, Prince Edward Island, Newfoundland, and Labrador.

Dose optimization thresholds: High: A treatment interval decrease of ≥11 days (1.57 weeks) with a posterior interval of ≤35 days (5 weeks), and/or a dose level increase of ≥1.5 mg/kg with a posterior dose level of ≥9 mg/kg. Low: A treatment interval decrease of ≥11 days (1.57 weeks) with a posterior interval of ≤46 days (6.57 weeks), and/or dose level increase of ≥1.5 mg/kg with a posterior dose level of ≥7 mg/kg.

Hazard ratio and 95% CI are from time-dependent Cox proportional hazards model 1 (Suppl Table 10b) with main factors of serum infliximab concentration (below vs. above threshold), a time-dependent covariate modelling dose optimization state (yes vs. no) within the post-index period and their interaction, and covariates as shown in the table

A hazard ratio of <1 indicates an advantage for the class level over the reference level. HR (95% CI) is shown in bold when p<0.05.

Suppl Table 11c: Subgroup Analysis Time-Dependent Cox Proportional Hazards Model for Patients with CD with no DO (Based on Low Threshold Criteria) Prior to their First Instance of TDM (Serum IFX Threshold: 5 μg/mL; Post-Index Period for Dose Optimization: +9 weeks; Post-Index Dose Optimization Threshold: Low)

| **Interaction factors (model 4)** | | | **p-value** |
| --- | --- | --- | --- |
| Dose optimization within post-index period (y vs. n) * Log time to dose optimization within post-index period | | | 0.0657 |
| **Class Level** | **Reference Level** | **HR (95% CI)** | **p-value** |
| ***Main factor contrasts (D.I. at 4 weeks)*** | | | |
| B: First TDM serum IFX <5 μg/mL;  Post-Index low-threshold DO  within 9 wk | A: First TDM serum IFX <5 μg/mL;  No post-Index low-threshold DO  within 9 wk | 0.62 (0.46, 0.84) | **0.0017** |
| C: First TDM serum IFX ≥5 μg/mL;  No post-Index low-threshold DO  within 9 wk | A: First TDM serum IFX <5 μg/mL;  No post-Index low-threshold DO  within 9 wk | 0.37 (0.29, 0.48) | **<0.0001** |
| C: First TDM serum IFX ≥5 μg/mL;  No post-Index low-threshold DO  within 9 wk | B: First TDM serum IFX <5 μg/mL;  Post-Index low-threshold DO  within 9 wk | 0.60 (0.42, 0.85) | **0.0038** |
| ***Main factor contrasts (D.I. at 6 weeks)*** | | | |
| B: First TDM serum IFX <5 μg/mL;  Post-Index low-threshold DO  within 9 wk | A: First TDM serum IFX <5 μg/mL;  No post-Index low-threshold DO  within 9 wk | 0.54 (0.40, 0.72) | **<0.0001** |
| C: First TDM serum IFX ≥5 μg/mL;  No post-Index low-threshold DO  within 9 wk | A: First TDM serum IFX <5 μg/mL;  No post-Index low-threshold DO  within 9 wk | 0.37 (0.29, 0.48) | **<0.0001** |
| C: First TDM serum IFX ≥5 μg/mL;  No post-Index low-threshold DO  within 9 wk | B: First TDM serum IFX <5 μg/mL;  Post-Index low-threshold DO  within 9 wk | 0.69 (0.49, 0.97) | **0.0307** |
| ***Covariates*** | | | |
| log(days to TDM), per unit | 0 | 0.62 (0.49, 0.79) | **<0.0001** |
| <65 | ≥65 | 0.77 (0.56, 1.07) | 0.1240 |
| Female | Male | 1.18 (0.94, 1.49) | 0.1497 |
| Q1 (<62 kg) | Q4 (≥86 kg) | 0.85 (0.62, 1.16) | 0.3135 |
| Q2 (62 to <73 kg) | Q4 (≥86 kg) | 0.75 (0.54, 1.03) | 0.0742 |
| Q3 (73 to <86 kg) | Q4 (≥86 kg) | 0.87 (0.64, 1.18) | 0.3703 |
| Alberta | Ontario | 1.55 (0.87, 2.76) | 0.1341 |
| Atlantic^†^ | Ontario | 1.11 (0.82, 1.50) | 0.5107 |
| British Columbia | Ontario | 1.20 (0.84, 1.72) | 0.3164 |
| Quebec | Ontario | 1.18 (0.88, 1.60) | 0.2714 |
| Saskatchewan/Manitoba | Ontario | 1.49 (0.37, 6.08) | 0.5772 |
| 2015 | 2018 | 1.94 (1.03, 3.64) | **0.0403** |
| 2016 | 2018 | 1.74 (1.13, 2.67) | **0.0118** |
| 2017 | 2018 | 1.17 (0.88, 1.57) | 0.2775 |

CI=confidence interval; IFX=infliximab; TDM=therapeutic drug monitoring; wk=weeks.

^†^Atlantic includes New Brunswick, Nova Scotia, Prince Edward Island, Newfoundland, and Labrador.

Dose optimization thresholds: High: A treatment interval decrease of ≥11 days (1.57 weeks) with a posterior interval of ≤35 days (5 weeks), and/or a dose level increase of ≥1.5 mg/kg with a posterior dose level of ≥9 mg/kg. Low: A treatment interval decrease of ≥11 days (1.57 weeks) with a posterior interval of ≤46 days (6.57 weeks), and/or dose level increase of ≥1.5 mg/kg with a posterior dose level of ≥7 mg/kg.

Hazard ratio and 95% CI are from time-dependent Cox proportional hazards model 4 (Suppl Table 10b) with main factors of serum infliximab concentration (below vs. above threshold), a time-dependent covariate modelling dose optimization state (yes vs. no) within the post-index period, the interaction between the time-dependent covariate and log time to dose optimization within the post-index period, and covariates as shown in the table.

A hazard ratio of <1 indicates an advantage for the class level over the reference level. HR (95% CI) is shown in bold when p<0.05.

Suppl Table 11d: Subgroup Analysis Time-Dependent Cox Proportional Hazards Model for Patients with CD with no DO (Based on Low Threshold Criteria) Prior to their First Instance of TDM (Serum IFX Threshold: 5 μg/mL; Post-Index Period for Dose Optimization: +17 weeks; Post-Index Dose Optimization Threshold: Low)

| **Interaction factor (model 1)** | | | **p-value** |
| --- | --- | --- | --- |
| Serum IFX concentration (below vs. above threshold) * Dose optimization within post-index period (y vs. n) | | | **0.0003** |
| **Class Level** | **Reference Level** | **HR (95% CI)** | **p-value** |
| ***Main factor contrasts*** | | | |
| B: First TDM serum IFX <5 μg/mL;  Post-Index low-threshold DO  within 17 wk | A: First TDM serum IFX <5 μg/mL;  No post-Index low-threshold DO  within 17 wk | 0.57 (0.43, 0.75) | **<0.0001** |
| C: First TDM serum IFX ≥5 μg/mL;  No post-Index low-threshold DO  within 17 wk | A: First TDM serum IFX <5 μg/mL;  No post-Index low-threshold DO  within 17 wk | 0.32 (0.24, 0.43) | **<0.0001** |
| C: First TDM serum IFX ≥5 μg/mL;  No post-Index low-threshold DO  within 17 wk | B: First TDM serum IFX <5 μg/mL;  Post-Index low-threshold DO  within 17 wk | 0.57 (0.41, 0.79) | **0.0008** |
| ***Covariates*** | | | |
| log(days to TDM), per unit | 0 | 0.61 (0.48, 0.78) | **<0.0001** |
| <65 | ≥65 | 0.77 (0.55, 1.06) | 0.1131 |
| Female | Male | 1.19 (0.95, 1.50) | 0.1341 |
| Q1 (<62 kg) | Q4 (≥86 kg) | 0.85 (0.62, 1.16) | 0.3109 |
| Q2 (62 to <73 kg) | Q4 (≥86 kg) | 0.75 (0.54, 1.03) | 0.0738 |
| Q3 (73 to <86 kg) | Q4 (≥86 kg) | 0.86 (0.63, 1.16) | 0.3183 |
| Alberta | Ontario | 1.51 (0.85, 2.68) | 0.1632 |
| Atlantic^†^ | Ontario | 1.12 (0.83, 1.51) | 0.4763 |
| British Columbia | Ontario | 1.25 (0.87, 1.79) | 0.2263 |
| Quebec | Ontario | 1.15 (0.85, 1.55) | 0.3607 |
| Saskatchewan/Manitoba | Ontario | 1.50 (0.37, 6.13) | 0.5712 |
| 2015 | 2018 | 2.02 (1.08, 3.80) | **0.0287** |
| 2016 | 2018 | 1.78 (1.15, 2.73) | **0.0089** |
| 2017 | 2018 | 1.17 (0.87, 1.56) | 0.3001 |

CI=confidence interval; IFX=infliximab; TDM=therapeutic drug monitoring; wk=weeks.

^†^Atlantic includes New Brunswick, Nova Scotia, Prince Edward Island, Newfoundland, and Labrador.

Dose optimization thresholds: High: A treatment interval decrease of ≥11 days (1.57 weeks) with a posterior interval of ≤35 days (5 weeks), and/or a dose level increase of ≥1.5 mg/kg with a posterior dose level of ≥9 mg/kg. Low: A treatment interval decrease of ≥11 days (1.57 weeks) with a posterior interval of ≤46 days (6.57 weeks), and/or dose level increase of ≥1.5 mg/kg with a posterior dose level of ≥7 mg/kg.

Hazard ratio and 95% CI are from time-dependent Cox proportional hazards model 1 (Suppl Table 10b) with main factors of serum infliximab concentration (below vs. above threshold), a time-dependent covariate modelling dose optimization state (yes vs. no) within the post-index period and their interaction, and covariates as shown in the table

A hazard ratio of <1 indicates an advantage for the class level over the reference level. HR (95% CI) is shown in bold when p<0.05.

Suppl Table 11e: Subgroup Analysis Time-Dependent Cox Proportional Hazards Model for Patients with CD with no DO (Based on Low Threshold Criteria) Prior to their First Instance of TDM (Serum IFX Threshold: 10 μg/mL; Post-Index Period for Dose Optimization: +9 weeks; Post-Index Dose Optimization Threshold: Low)

| **Class Level** | **Reference Level** | **HR (95% CI)** | **p-value** |
| --- | --- | --- | --- |
| ***Main factor contrasts*** | | | |
| B: First TDM serum IFX <10 μg/mL;  Post-Index low-threshold DO  within 9 wk | A: First TDM serum IFX <10 μg/mL; No post-Index low-threshold DO  within 9 wk | 0.71 (0.54, 0.94) | **0.0179** |
| C: First TDM serum IFX ≥10 μg/mL;  No post-Index low-threshold DO  within 9 wk | A: First TDM serum IFX <10 μg/mL; No post-Index low-threshold DO  within 9 wk | 0.56 (0.40, 0.78) | **0.0007** |
| C: First TDM serum IFX ≥10 μg/mL;  No post-Index low-threshold DO  within 9 wk | B: First TDM serum IFX <10 μg/mL; Post-Index low-threshold DO  within 9 wk | 0.78 (0.52, 1.16) | 0.2220 |
| ***Covariates*** | | | |
| log(days to TDM), per unit | 0 | 0.63 (0.50, 0.81) | **0.0002** |
| <65 | ≥65 | 0.71 (0.52, 0.99) | **0.0439** |
| Female | Male | 1.16 (0.92, 1.46) | 0.2141 |
| Q1 (<62 kg) | Q4 (≥86 kg) | 0.86 (0.63, 1.18) | 0.3410 |
| Q2 (62 to <73 kg) | Q4 (≥86 kg) | 0.76 (0.55, 1.04) | 0.0891 |
| Q3 (73 to <86 kg) | Q4 (≥86 kg) | 0.88 (0.65, 1.19) | 0.4106 |
| Alberta | Ontario | 1.57 (0.88, 2.79) | 0.1268 |
| Atlantic^†^ | Ontario | 1.17 (0.87, 1.58) | 0.3051 |
| British Columbia | Ontario | 1.23 (0.86, 1.76) | 0.2635 |
| Quebec | Ontario | 1.29 (0.96, 1.74) | 0.0929 |
| Saskatchewan/Manitoba | Ontario | 1.58 (0.39, 6.42) | 0.5229 |
| 2015 | 2018 | 1.80 (0.96, 3.39) | 0.0674 |
| 2016 | 2018 | 1.83 (1.19, 2.82) | **0.0057** |
| 2017 | 2018 | 1.16 (0.87, 1.54) | 0.3246 |

CI=confidence interval; IFX=infliximab; TDM=therapeutic drug monitoring; wk=weeks.

^†^Atlantic includes New Brunswick, Nova Scotia, Prince Edward Island, Newfoundland, and Labrador.

Dose optimization thresholds: High: A treatment interval decrease of ≥11 days (1.57 weeks) with a posterior interval of ≤35 days (5 weeks), and/or a dose level increase of ≥1.5 mg/kg with a posterior dose level of ≥9 mg/kg. Low: A treatment interval decrease of ≥11 days (1.57 weeks) with a posterior interval of ≤46 days (6.57 weeks), and/or dose level increase of ≥1.5 mg/kg with a posterior dose level of ≥7 mg/kg.

Hazard ratio and 95% CI are from time-dependent Cox proportional hazards model 2 (Suppl Table 10b) with main factors of serum infliximab concentration (below vs. above threshold) and a time-dependent covariate modelling dose optimization state (yes vs. no) within the post-index period, and covariates as shown in the table.

A hazard ratio of <1 indicates an advantage for the class level over the reference level. HR (95% CI) is shown in bold when p<0.05.

Suppl Table 11f: Subgroup Analysis Time-Dependent Cox Proportional Hazards Model for Patients with CD with no DO (Based on Low Threshold Criteria) Prior to their First Instance of TDM (Serum IFX Threshold: 10 μg/mL; Post-Index Period for Dose Optimization: +17 weeks; Post-Index Dose Optimization Threshold: Low)

| **Class Level** | **Reference Level** | **HR (95% CI)** | **p-value** |
| --- | --- | --- | --- |
| ***Main factor contrasts*** | | | |
| B: First TDM serum IFX <10 μg/mL;  Post-Index low-threshold DO  within 17 wk | A: First TDM serum IFX <10 μg/mL; No post-Index low-threshold DO  within 17 wk | 0.86 (0.66, 1.11) | 0.2424 |
| C: First TDM serum IFX ≥10 μg/mL;  No post-Index low-threshold DO  within 17 wk | A: First TDM serum IFX <10 μg/mL; No post-Index low-threshold DO  within 17 wk | 0.58 (0.41, 0.81) | **0.0015** |
| C: First TDM serum IFX ≥10 μg/mL;  No post-Index low-threshold DO  within 17 wk | B: First TDM serum IFX <10 μg/mL; Post-Index low-threshold DO  within 17 wk | 0.67 (0.46, 0.98) | **0.0401** |
| ***Covariates*** | | | |
| log(days to TDM), per unit | 0 | 0.64 (0.50, 0.81) | **0.0002** |
| <65 | ≥65 | 0.71 (0.51, 0.99) | **0.0420** |
| Female | Male | 1.16 (0.92, 1.46) | 0.2130 |
| Q1 (<62 kg) | Q4 (≥86 kg) | 0.85 (0.62, 1.16) | 0.3080 |
| Q2 (62 to <73 kg) | Q4 (≥86 kg) | 0.75 (0.55, 1.04) | 0.0855 |
| Q3 (73 to <86 kg) | Q4 (≥86 kg) | 0.88 (0.65, 1.19) | 0.4024 |
| Alberta | Ontario | 1.58 (0.89, 2.81) | 0.1200 |
| Atlantic^†^ | Ontario | 1.19 (0.88, 1.61) | 0.2557 |
| British Columbia | Ontario | 1.24 (0.87, 1.78) | 0.2373 |
| Quebec | Ontario | 1.28 (0.95, 1.73) | 0.1008 |
| Saskatchewan/Manitoba | Ontario | 1.63 (0.40, 6.61) | 0.4975 |
| 2015 | 2018 | 1.83 (0.97, 3.45) | 0.0600 |
| 2016 | 2018 | 1.84 (1.20, 2.84) | **0.0053** |
| 2017 | 2018 | 1.16 (0.87, 1.55) | 0.3165 |

CI=confidence interval; IFX=infliximab; TDM=therapeutic drug monitoring; wk=weeks.

^†^Atlantic includes New Brunswick, Nova Scotia, Prince Edward Island, Newfoundland, and Labrador.

Dose optimization thresholds: High: A treatment interval decrease of ≥11 days (1.57 weeks) with a posterior interval of ≤35 days (5 weeks), and/or a dose level increase of ≥1.5 mg/kg with a posterior dose level of ≥9 mg/kg. Low: A treatment interval decrease of ≥11 days (1.57 weeks) with a posterior interval of ≤46 days (6.57 weeks), and/or dose level increase of ≥1.5 mg/kg with a posterior dose level of ≥7 mg/kg.

Hazard ratio and 95% CI are from time-dependent Cox proportional hazards model 2 (Suppl Table 10b) with main factors of serum infliximab concentration (below vs. above threshold) and a time-dependent covariate modelling dose optimization state (yes vs. no) within the post-index period, and covariates as shown in the table.

A hazard ratio of <1 indicates an advantage for the class level over the reference level. HR (95% CI) is shown in bold when p<0.05.

Suppl Table 11g: Subgroup Analysis Time-Dependent Cox Proportional Hazards Model for Patients with CD with no DO (Based on Low Threshold Criteria) Prior to their First Instance of TDM (Serum IFX Threshold: 3 μg/mL; Post-Index Period for Dose Optimization: +9 weeks; Post-Index Dose Optimization Threshold: High)

| **Class Level** | **Reference Level** | **HR (95% CI)** | **p-value** |
| --- | --- | --- | --- |
| ***Main factor contrasts*** | | | |
| B: First TDM serum IFX <3 μg/mL;  Post-Index high-threshold DO  within 9 wk | A: First TDM serum IFX <3 μg/mL;  No post-Index high-threshold DO  within 9 wk | 0.61 (0.44, 0.85) | **0.0035** |
| C: First TDM serum IFX ≥3 μg/mL;  No post-Index high-threshold DO  within 9 wk | A: First TDM serum IFX <3 μg/mL;  No post-Index high-threshold DO  within 9 wk | 0.30 (0.24, 0.39) | **<0.0001** |
| C: First TDM serum IFX ≥3 μg/mL;  No post-Index high-threshold DO  within 9 wk | B: First TDM serum IFX <3 μg/mL;  Post-Index high-threshold DO  within 9 wk | 0.50 (0.35, 0.70) | **<0.0001** |
| ***Covariates*** | | | |
| log(days to TDM), per unit | 0 | 0.60 (0.47, 0.76) | **<0.0001** |
| <65 | ≥65 | 0.80 (0.57, 1.11) | 0.1749 |
| Female | Male | 1.17 (0.93, 1.48) | 0.1728 |
| Q1 (<62 kg) | Q4 (≥86 kg) | 0.77 (0.56, 1.05) | 0.1019 |
| Q2 (62 to <73 kg) | Q4 (≥86 kg) | 0.75 (0.55, 1.04) | 0.0818 |
| Q3 (73 to <86 kg) | Q4 (≥86 kg) | 0.91 (0.67, 1.23) | 0.5309 |
| Alberta | Ontario | 1.75 (0.98, 3.11) | 0.0575 |
| Atlantic^†^ | Ontario | 1.16 (0.86, 1.57) | 0.3296 |
| British Columbia | Ontario | 1.28 (0.90, 1.84) | 0.1730 |
| Quebec | Ontario | 1.23 (0.91, 1.65) | 0.1778 |
| Saskatchewan/Manitoba | Ontario | 2.32 (0.57, 9.46) | 0.2418 |
| 2015 | 2018 | 2.21 (1.18, 4.16) | **0.0138** |
| 2016 | 2018 | 1.85 (1.21, 2.84) | **0.0047** |
| 2017 | 2018 | 1.14 (0.85, 1.52) | 0.3835 |

CI=confidence interval; IFX=infliximab; TDM=therapeutic drug monitoring; wk=weeks.

^†^Atlantic includes New Brunswick, Nova Scotia, Prince Edward Island, Newfoundland, and Labrador.

Dose optimization thresholds: High: A treatment interval decrease of ≥11 days (1.57 weeks) with a posterior interval of ≤35 days (5 weeks), and/or a dose level increase of ≥1.5 mg/kg with a posterior dose level of ≥9 mg/kg. Low: A treatment interval decrease of ≥11 days (1.57 weeks) with a posterior interval of ≤46 days (6.57 weeks), and/or dose level increase of ≥1.5 mg/kg with a posterior dose level of ≥7 mg/kg.

Hazard ratio and 95% CI are from time-dependent Cox proportional hazards model 2 (Suppl Table 10b) with main factors of serum infliximab concentration (below vs. above threshold) and a time-dependent covariate modelling dose optimization state (yes vs. no) within the post-index period, and covariates as shown in the table.

A hazard ratio of <1 indicates an advantage for the class level over the reference level. HR (95% CI) is shown in bold when p<0.05.

Suppl Table 11h: Subgroup Analysis Time-Dependent Cox Proportional Hazards Model for Patients with CD with no DO (Based on Low Threshold Criteria) Prior to their First Instance of TDM (Serum IFX Threshold: 3 μg/mL; Post-Index Period for Dose Optimization: +17 weeks; Post-Index Dose Optimization Threshold: High)

| **Interaction factor (model 1)** | | | **p-value** |
| --- | --- | --- | --- |
| Serum IFX concentration (below vs. above threshold) * Dose optimization within post-index period (y vs. n) | | | **0.0167** |
| **Class Level** | **Reference Level** | **HR (95% CI)** | **p-value** |
| ***Main factor contrasts*** | | | |
| B: First TDM serum IFX <3 μg/mL;  Post-Index high-threshold DO  within 17 wk | A: First TDM serum IFX <3 μg/mL;  No post-Index high-threshold DO  within 17 wk | 0.63 (0.46, 0.87) | **0.0052** |
| C: First TDM serum IFX ≥3 μg/mL;  No post-Index high-threshold DO  within 17 wk | A: First TDM serum IFX <3 μg/mL;  No post-Index high-threshold DO  within 17 wk | 0.29 (0.22, 0.37) | **<0.0001** |
| C: First TDM serum IFX ≥3 μg/mL;  No post-Index high-threshold DO  within 17 wk | B: First TDM serum IFX <3 μg/mL;  Post-Index high-threshold DO within 17 wk | 0.45 (0.32, 0.63) | **<0.0001** |
| ***Covariates*** | | | |
| log(days to TDM), per unit | 0 | 0.59 (0.46, 0.75) | **<0.0001** |
| <65 | ≥65 | 0.79 (0.57, 1.10) | 0.1682 |
| Female | Male | 1.17 (0.93, 1.47) | 0.1749 |
| Q1 (<62 kg) | Q4 (≥86 kg) | 0.77 (0.56, 1.05) | 0.1001 |
| Q2 (62 to <73 kg) | Q4 (≥86 kg) | 0.75 (0.54, 1.03) | 0.0745 |
| Q3 (73 to <86 kg) | Q4 (≥86 kg) | 0.91 (0.67, 1.23) | 0.5341 |
| Alberta | Ontario | 1.74 (0.98, 3.10) | 0.0593 |
| Atlantic^†^ | Ontario | 1.16 (0.86, 1.57) | 0.3208 |
| British Columbia | Ontario | 1.28 (0.89, 1.83) | 0.1845 |
| Quebec | Ontario | 1.21 (0.90, 1.63) | 0.2092 |
| Saskatchewan/Manitoba | Ontario | 2.38 (0.58, 9.74) | 0.2265 |
| 2015 | 2018 | 2.29 (1.22, 4.32) | **0.0102** |
| 2016 | 2018 | 1.91 (1.25, 2.93) | **0.0030** |
| 2017 | 2018 | 1.15 (0.86, 1.53) | 0.3449 |

CI=confidence interval; IFX=infliximab; TDM=therapeutic drug monitoring; wk=weeks.

^†^Atlantic includes New Brunswick, Nova Scotia, Prince Edward Island, Newfoundland, and Labrador.

Dose optimization thresholds: High: A treatment interval decrease of ≥11 days (1.57 weeks) with a posterior interval of ≤35 days (5 weeks), and/or a dose level increase of ≥1.5 mg/kg with a posterior dose level of ≥9 mg/kg. Low: A treatment interval decrease of ≥11 days (1.57 weeks) with a posterior interval of ≤46 days (6.57 weeks), and/or dose level increase of ≥1.5 mg/kg with a posterior dose level of ≥7 mg/kg.

Hazard ratio and 95% CI are from time-dependent Cox proportional hazards model 1 (Suppl Table 10b) with main factors of serum infliximab concentration (below vs. above threshold), a time-dependent covariate modelling dose optimization state (yes vs. no) within the post-index period and their interaction, and covariates as shown in the table

A hazard ratio of <1 indicates an advantage for the class level over the reference level. HR (95% CI) is shown in bold when p<0.05.

Suppl Table 11i: Subgroup Analysis Time-Dependent Cox Proportional Hazards Model for Patients with CD with no DO (Based on Low Threshold Criteria) Prior to their First Instance of TDM (Serum IFX Threshold: 5 μg/mL; Post-Index Period for Dose Optimization: +9 weeks; Post-Index Dose Optimization Threshold: High)

| **Interaction factors (model 4)** | | | **p-value** |
| --- | --- | --- | --- |
| Dose optimization within post-index period (y vs. n) * Log time to dose optimization within post-index period | | | 0.0912 |
| **Class Level** | **Reference Level** | **HR (95% CI)** | **p-value** |
| ***Main factor contrasts (D.I. at 4 weeks)*** | | | |
| B: First TDM serum IFX <5 μg/mL;  Post-Index high-threshold DO  within 9 wk | A: First TDM serum IFX <5 μg/mL;  No post-Index high-threshold DO  within 9 wk | 0.83 (0.60, 1.15) | 0.2595 |
| C: First TDM serum IFX ≥5 μg/mL;  No post-Index high-threshold DO  within 9 wk | A: First TDM serum IFX <5 μg/mL;  No post-Index high-threshold DO  within 9 wk | 0.42 (0.32, 0.54) | **<0.0001** |
| C: First TDM serum IFX ≥5 μg/mL;  No post-Index high-threshold DO  within 9 wk | B: First TDM serum IFX <5 μg/mL;  Post-Index high-threshold DO  within 9 wk | 0.50 (0.34, 0.73) | **0.0003** |
| ***Main factor contrasts (D.I. at 6 weeks)*** | | | |
| B: First TDM serum IFX <5 μg/mL;  Post-Index high-threshold DO  within 9 wk | A: First TDM serum IFX <5 μg/mL;  No post-Index high-threshold DO  within 9 wk | 0.72 (0.51, 1.01) | 0.0574 |
| C: First TDM serum IFX ≥5 μg/mL;  No post-Index high-threshold DO  within 9 wk | A: First TDM serum IFX <5 μg/mL;  No post-Index high-threshold DO  within 9 wk | 0.42 (0.32, 0.54) | **<0.0001** |
| C: First TDM serum IFX ≥5 μg/mL;  No post-Index high-threshold DO  within 9 wk | B: First TDM serum IFX <5 μg/mL;  Post-Index high-threshold DO  within 9 wk | 0.58 (0.39, 0.85) | **0.0049** |
| ***Covariates*** | | | |
| log(days to TDM), per unit | 0 | 0.63 (0.49, 0.80) | **0.0001** |
| <65 | ≥65 | 0.77 (0.55, 1.06) | 0.1121 |
| Female | Male | 1.18 (0.94, 1.49) | 0.1508 |
| Q1 (<62 kg) | Q4 (≥86 kg) | 0.83 (0.61, 1.14) | 0.2467 |
| Q2 (62 to <73 kg) | Q4 (≥86 kg) | 0.74 (0.54, 1.02) | 0.0666 |
| Q3 (73 to <86 kg) | Q4 (≥86 kg) | 0.86 (0.63, 1.16) | 0.3158 |
| Alberta | Ontario | 1.65 (0.93, 2.93) | 0.0896 |
| Atlantic^†^ | Ontario | 1.16 (0.86, 1.57) | 0.3311 |
| British Columbia | Ontario | 1.23 (0.86, 1.77) | 0.2510 |
| Quebec | Ontario | 1.18 (0.88, 1.59) | 0.2768 |
| Saskatchewan/Manitoba | Ontario | 1.63 (0.40, 6.64) | 0.4953 |
| 2015 | 2018 | 2.01 (1.07, 3.77) | **0.0306** |
| 2016 | 2018 | 1.79 (1.17, 2.75) | **0.0077** |
| 2017 | 2018 | 1.18 (0.88, 1.57) | 0.2631 |

CI=confidence interval; IFX=infliximab; TDM=therapeutic drug monitoring; wk=weeks.

^†^Atlantic includes New Brunswick, Nova Scotia, Prince Edward Island, Newfoundland, and Labrador.

Dose optimization thresholds: High: A treatment interval decrease of ≥11 days (1.57 weeks) with a posterior interval of ≤35 days (5 weeks), and/or a dose level increase of ≥1.5 mg/kg with a posterior dose level of ≥9 mg/kg. Low: A treatment interval decrease of ≥11 days (1.57 weeks) with a posterior interval of ≤46 days (6.57 weeks), and/or dose level increase of ≥1.5 mg/kg with a posterior dose level of ≥7 mg/kg.
[truncated: 361,165 more chars]
